# Supplementary figures and images for: Pooled genome-wide CRISPR activation screening for rapamycin resistance genes in Drosophila cells (part 1 of 2)
Source: eLife. 2023 Apr 20;12:e85542. doi: 10.7554/eLife.85542 (PMC10118385; doi:10.7554/eLife.85542)

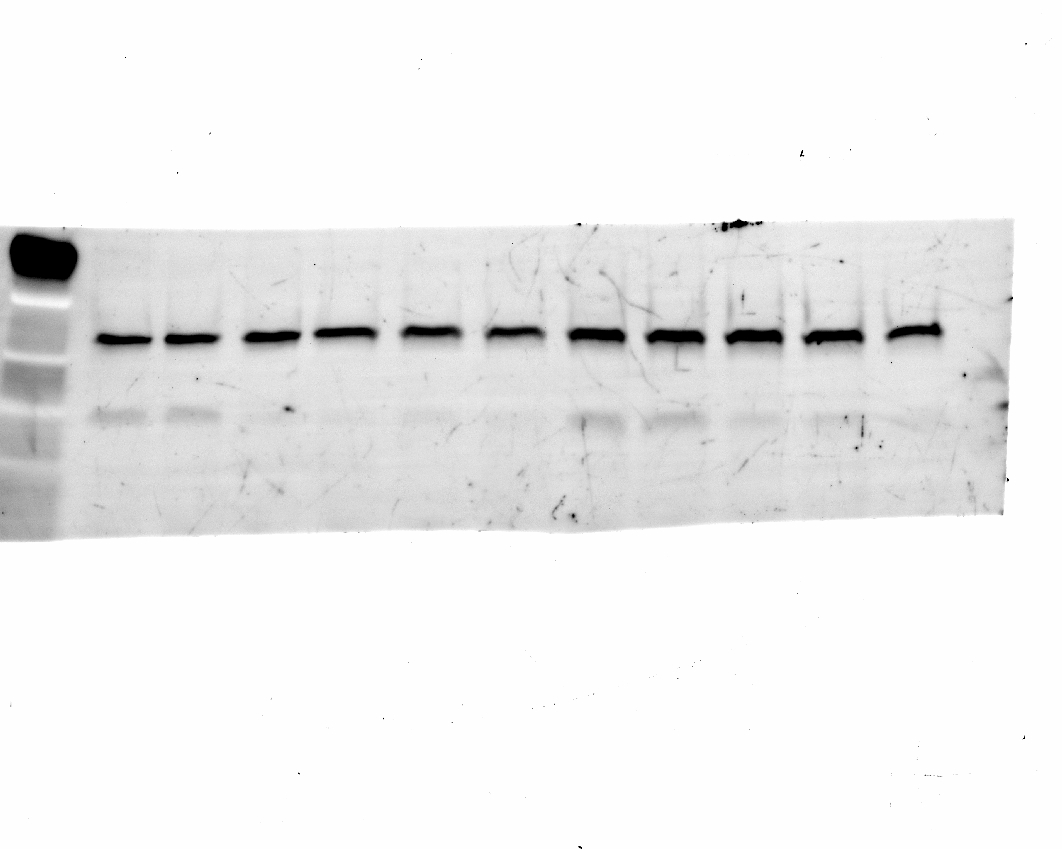

Supplement: Figure 3—source data 1. [file elife-85542-fig3-data1.zip › Figure 3 source data/Figure 3A/Figure 3A-actin raw data.jpg]

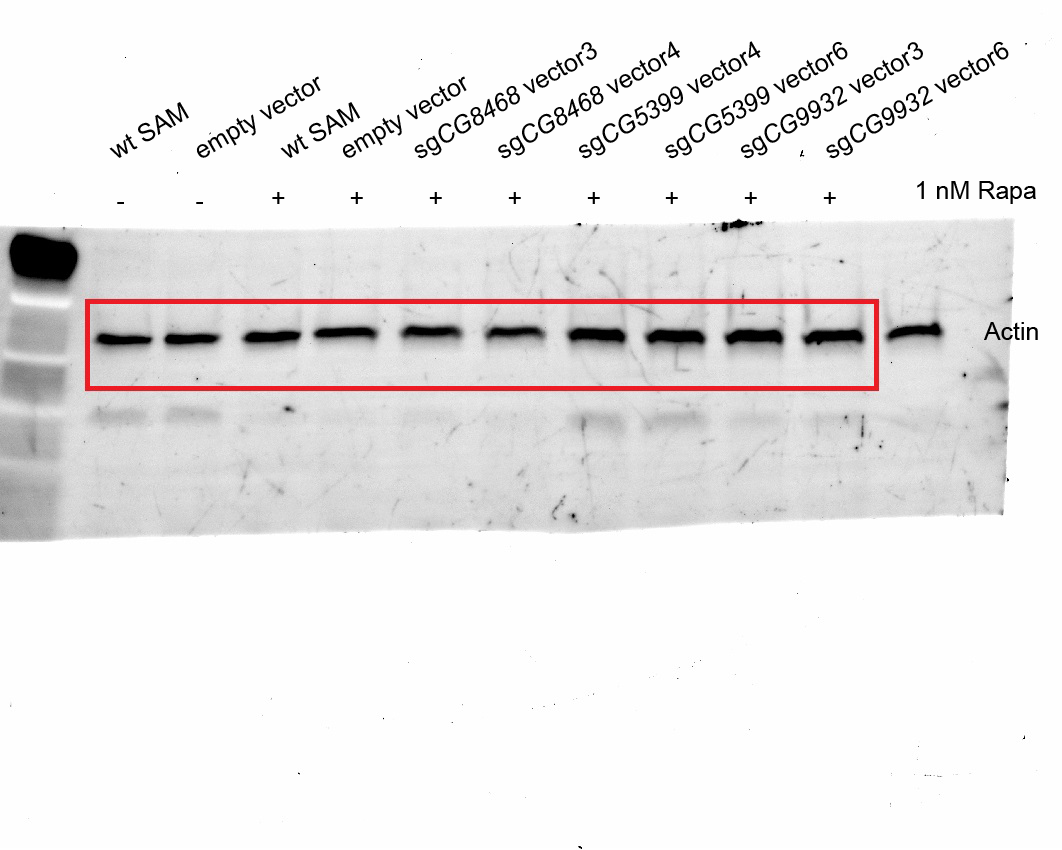

Supplement: Figure 3—source data 1. [file elife-85542-fig3-data1.zip › Figure 3 source data/Figure 3A/Figure 3A-actin.tif]

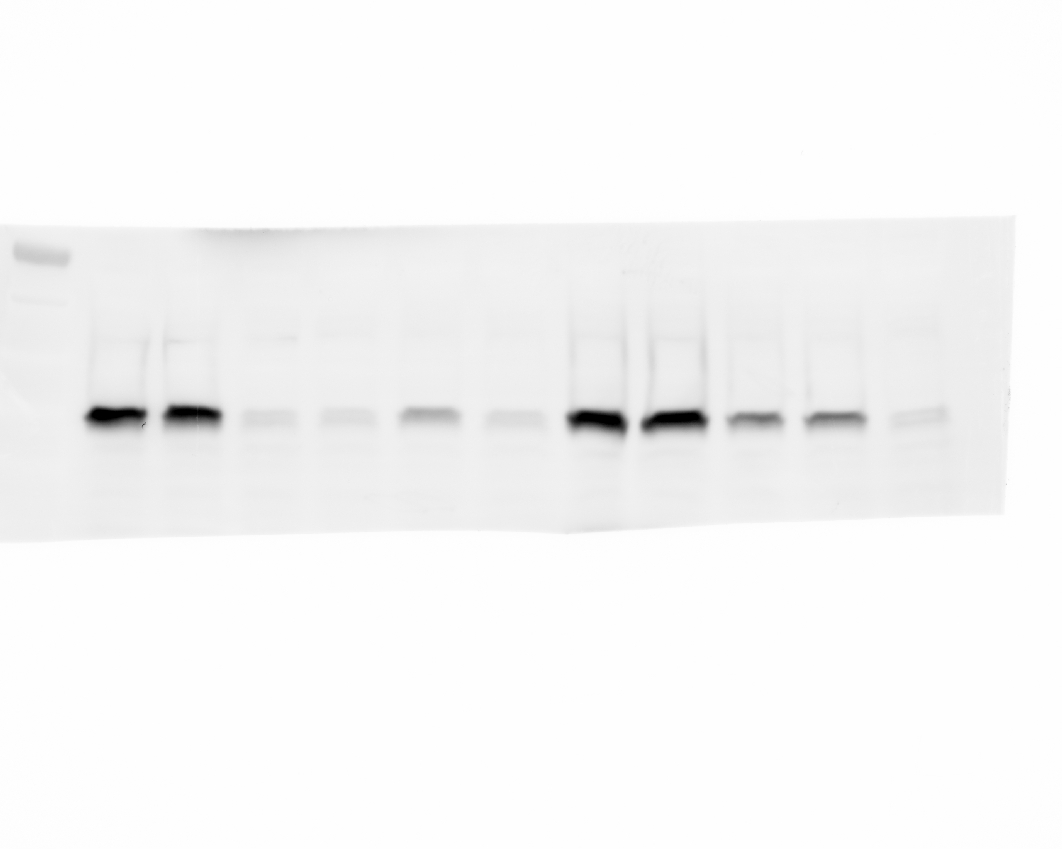

Supplement: Figure 3—source data 1. [file elife-85542-fig3-data1.zip › Figure 3 source data/Figure 3A/Figure 3A-pS6 raw data.jpg]

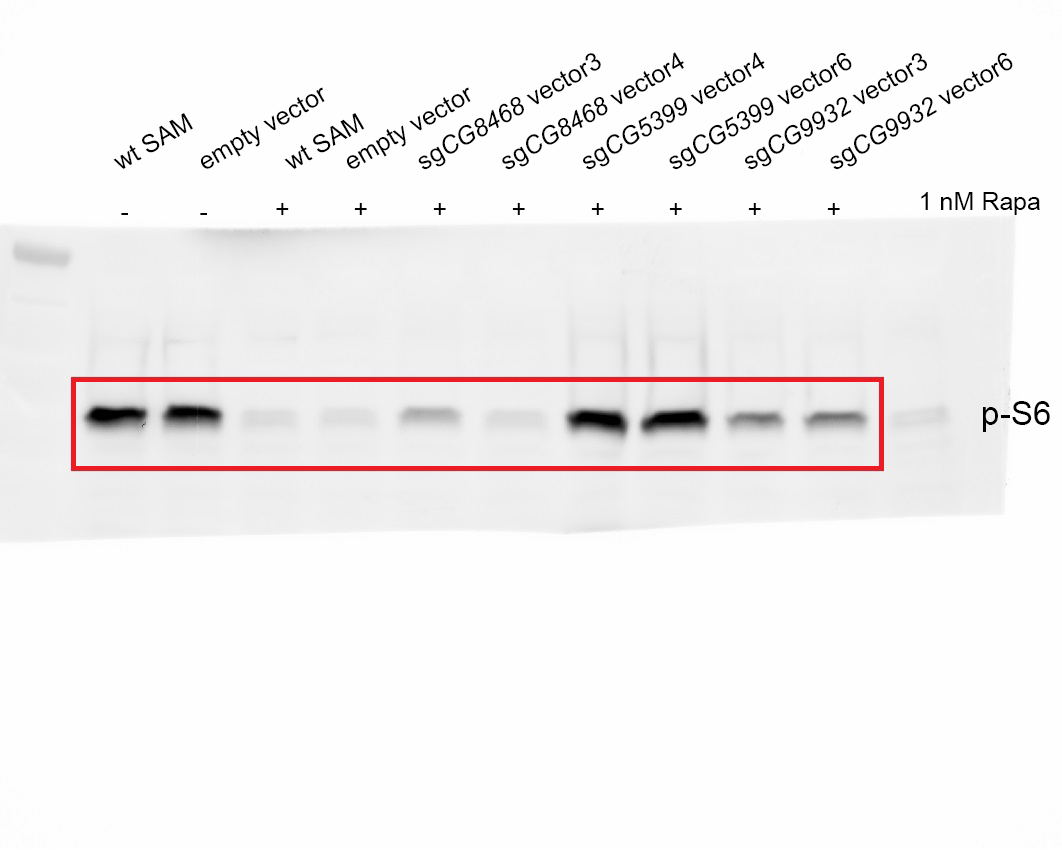

Supplement: Figure 3—source data 1. [file elife-85542-fig3-data1.zip › Figure 3 source data/Figure 3A/Figure 3A-pS6.tif]

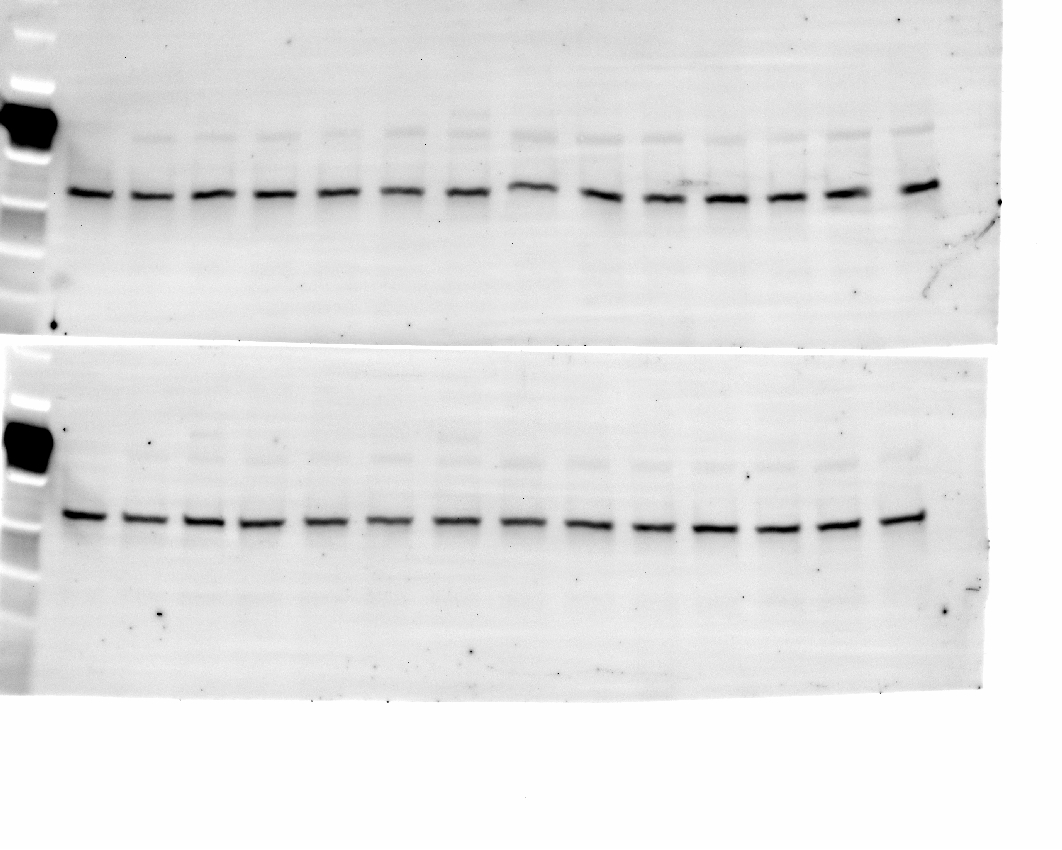

Supplement: Figure 3—source data 1. [file elife-85542-fig3-data1.zip › Figure 3 source data/Figure 3B/Figure 3B-actin raw data.jpg]

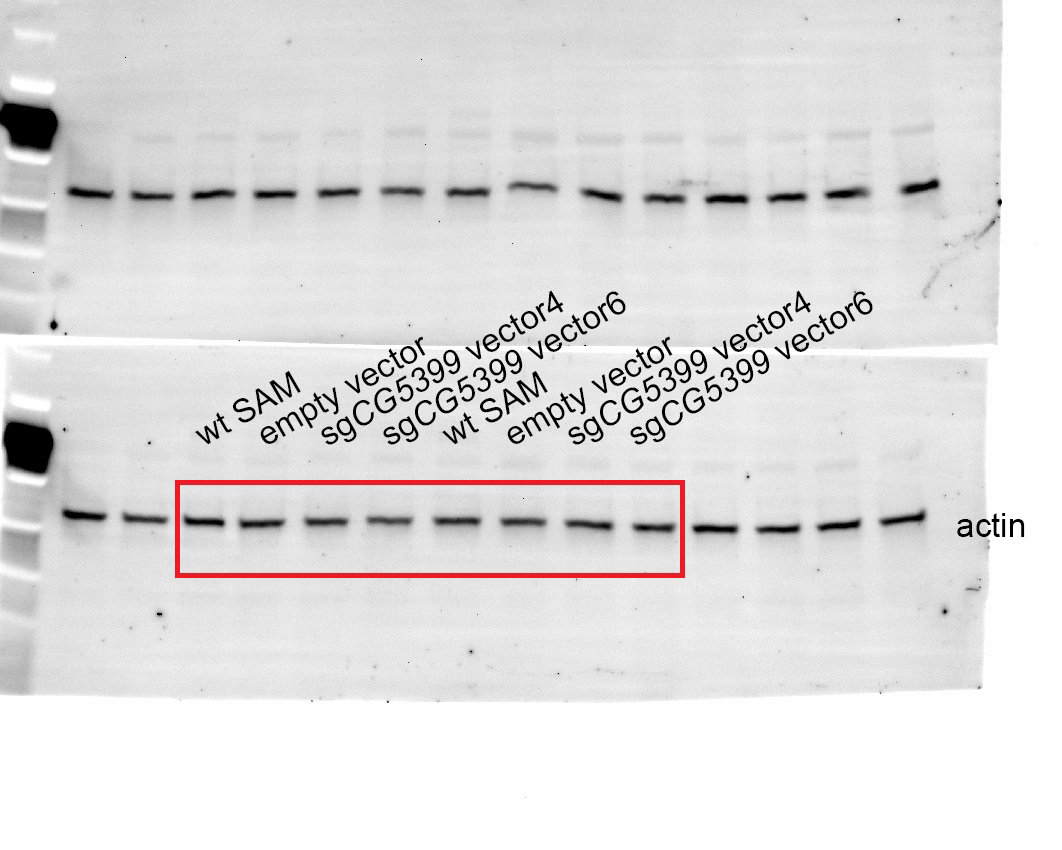

Supplement: Figure 3—source data 1. [file elife-85542-fig3-data1.zip › Figure 3 source data/Figure 3B/Figure 3B-actin.tif]

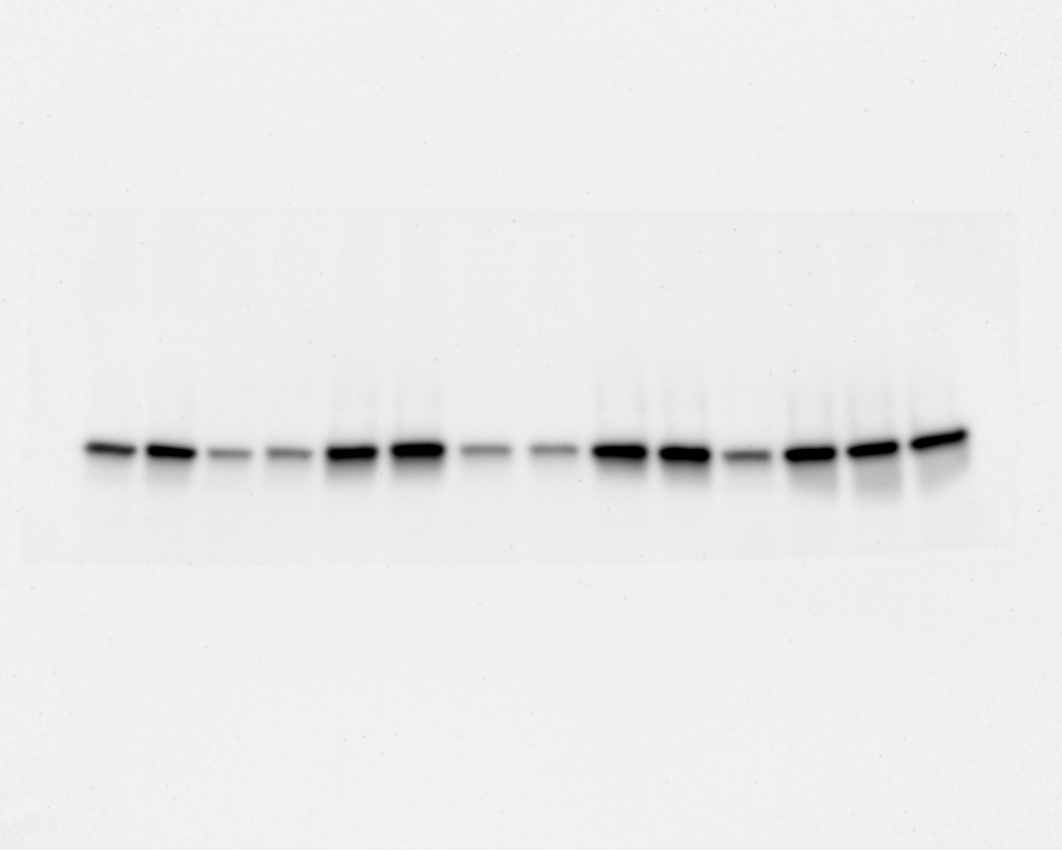

Supplement: Figure 3—source data 1. [file elife-85542-fig3-data1.zip › Figure 3 source data/Figure 3B/Figure 3B-pS6 raw data.jpg]

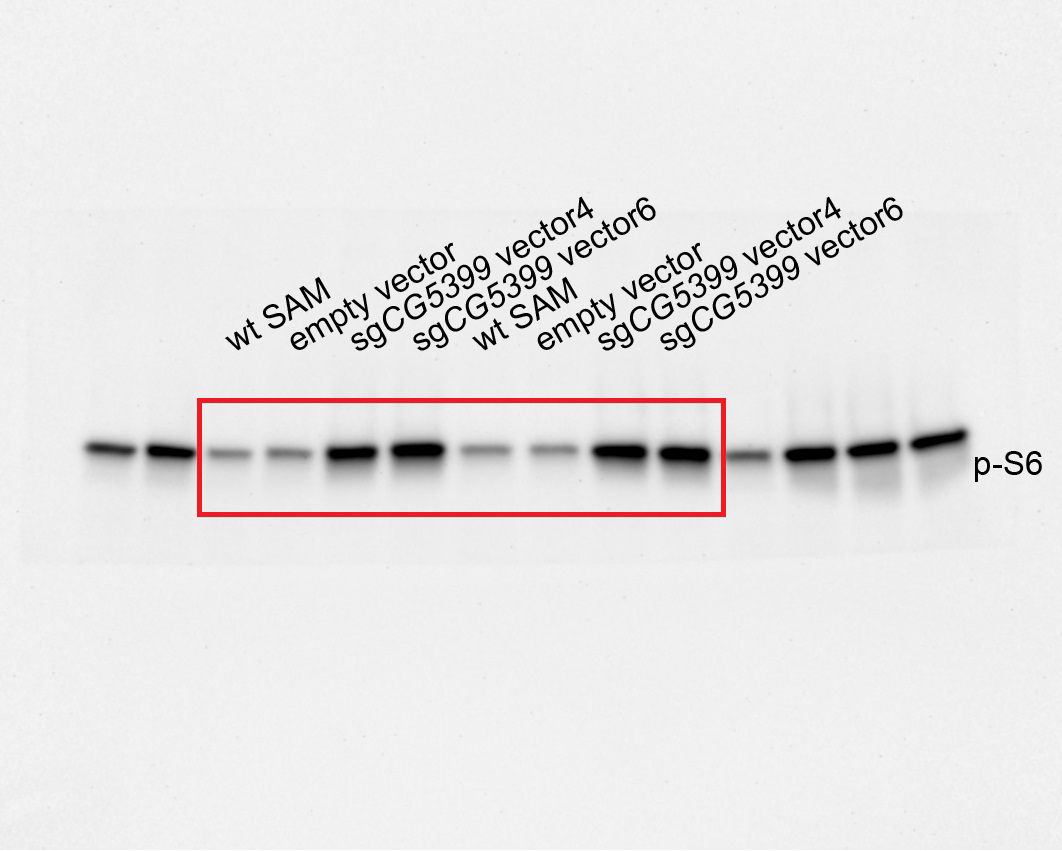

Supplement: Figure 3—source data 1. [file elife-85542-fig3-data1.zip › Figure 3 source data/Figure 3B/Figure 3B-pS6.tif]

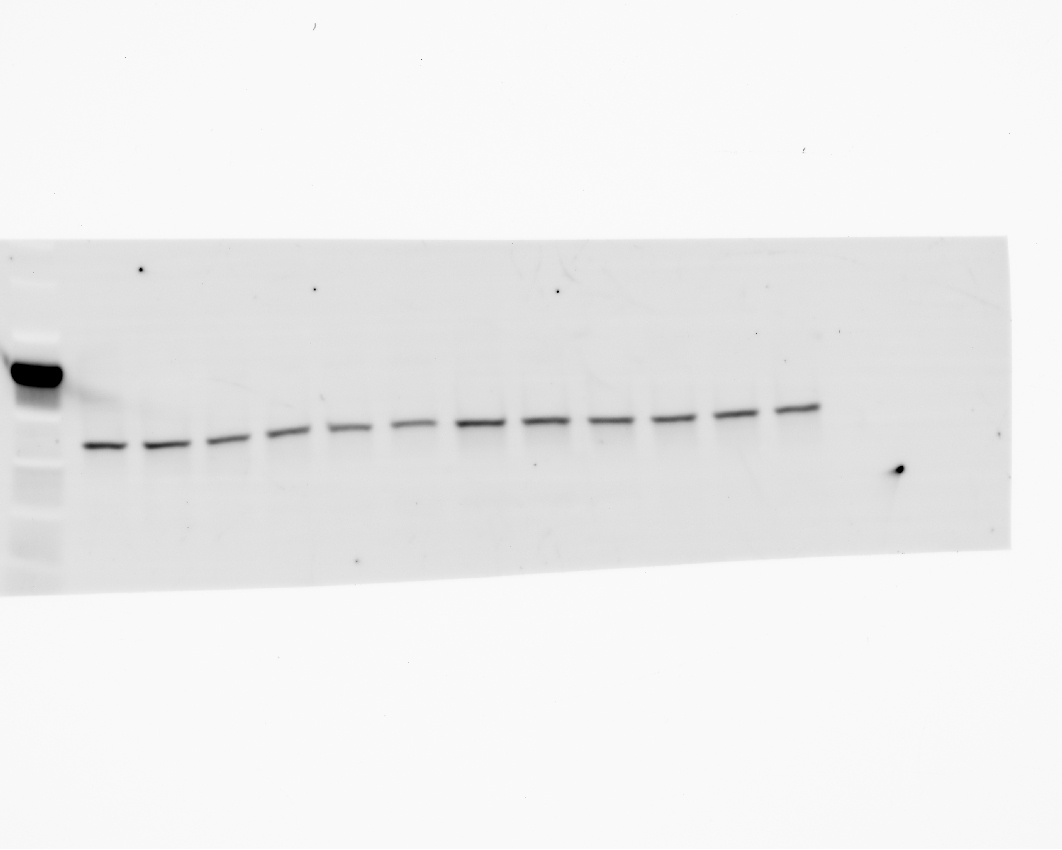

Supplement: Figure 3—source data 1. [file elife-85542-fig3-data1.zip › Figure 3 source data/Figure 3C/Figure 3C-actin for pAkt blot raw data.jpg]

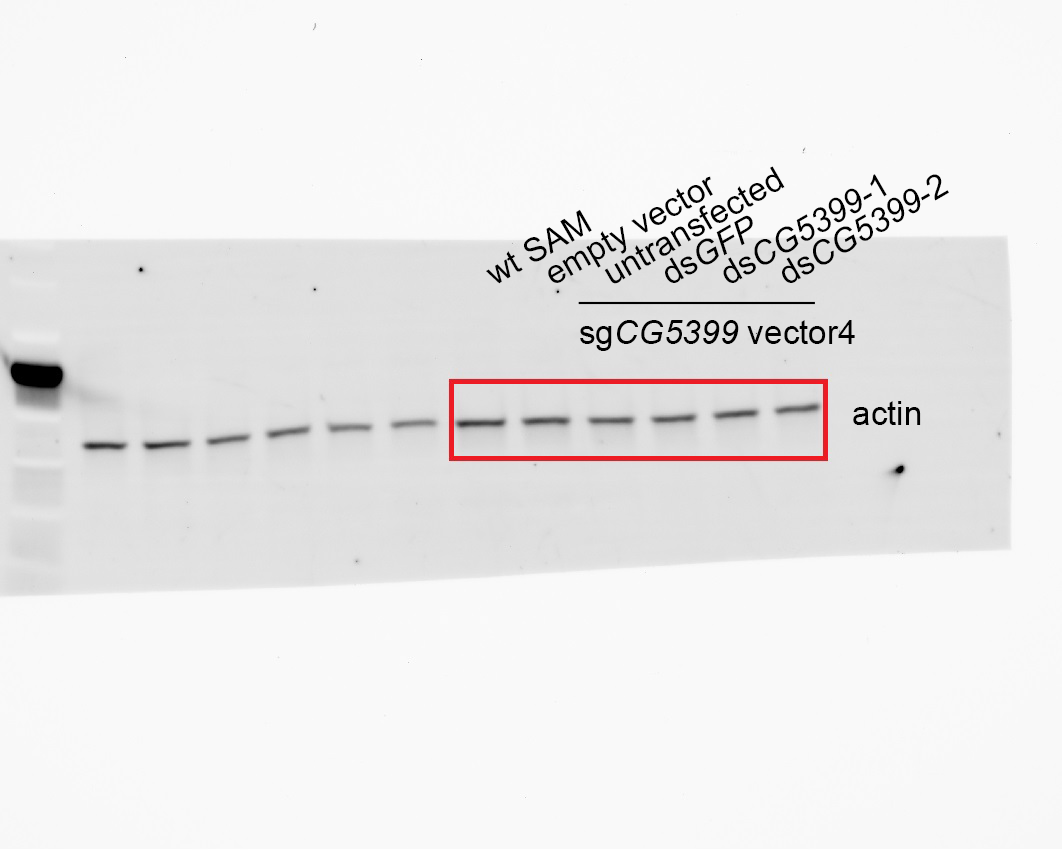

Supplement: Figure 3—source data 1. [file elife-85542-fig3-data1.zip › Figure 3 source data/Figure 3C/Figure 3C-actin for pAkt blot.tif]

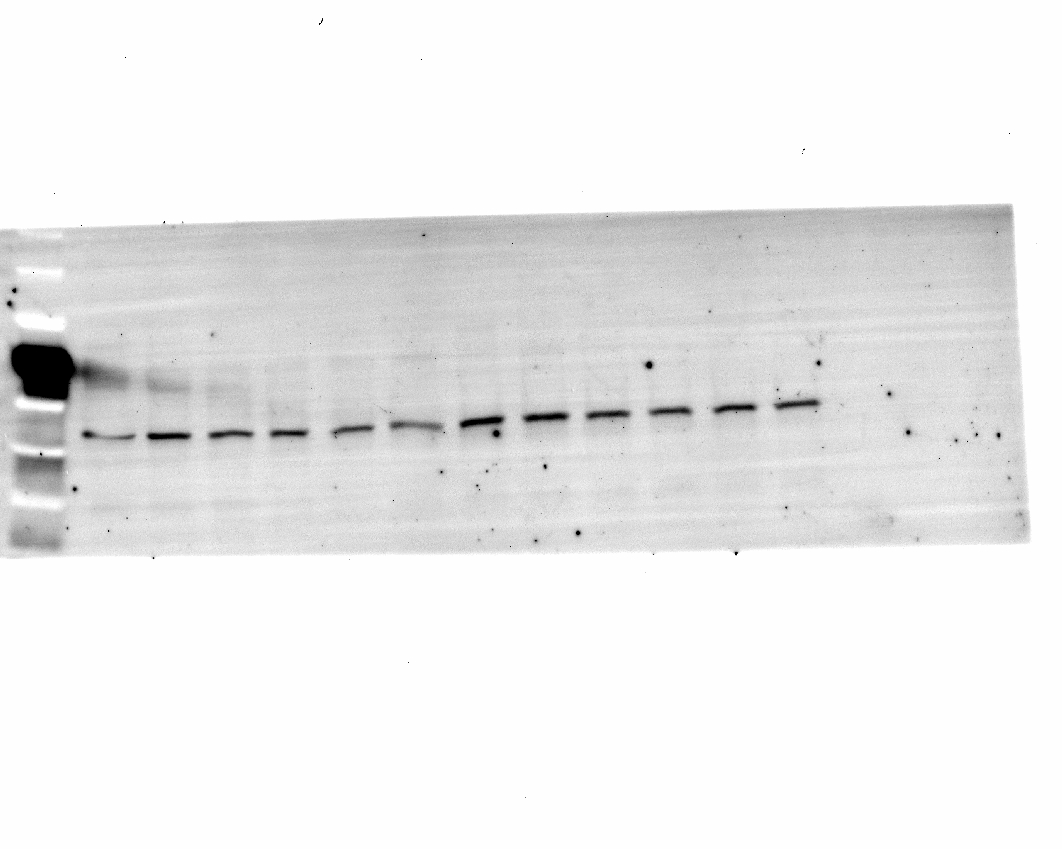

Supplement: Figure 3—source data 1. [file elife-85542-fig3-data1.zip › Figure 3 source data/Figure 3C/Figure 3C-actin for pS6 blot raw data.jpg]

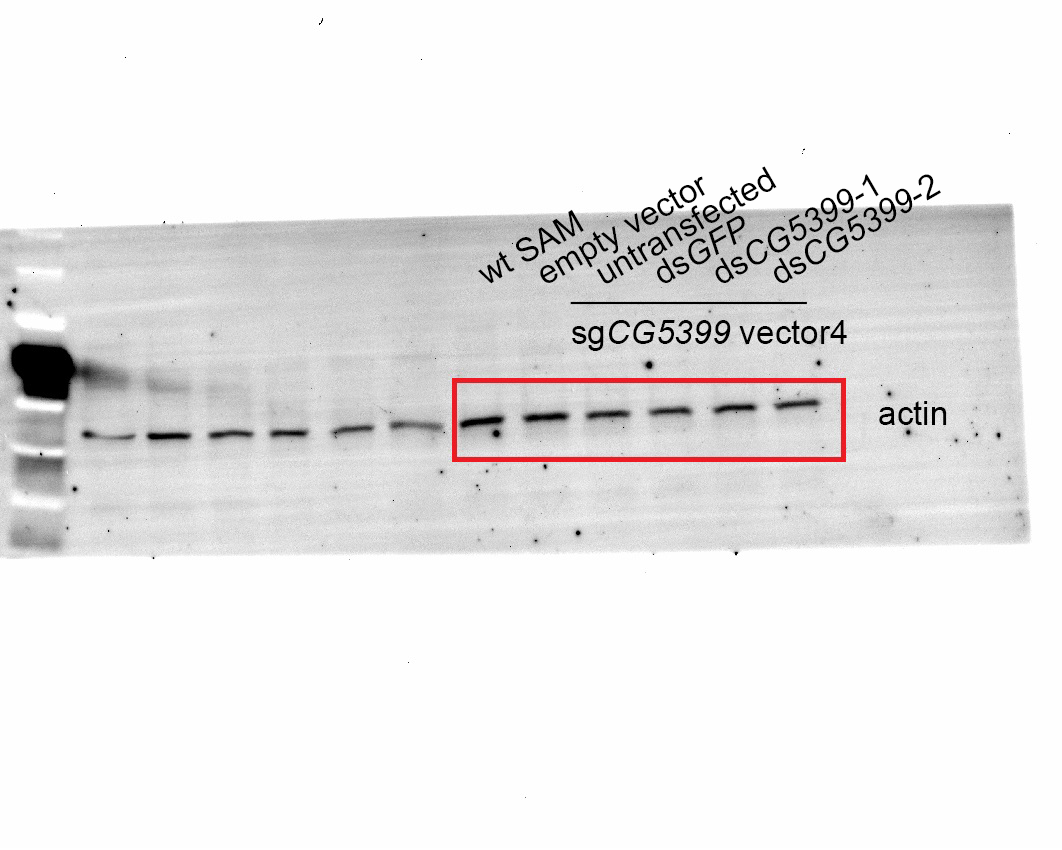

Supplement: Figure 3—source data 1. [file elife-85542-fig3-data1.zip › Figure 3 source data/Figure 3C/Figure 3C-actin for pS6 blot.tif]

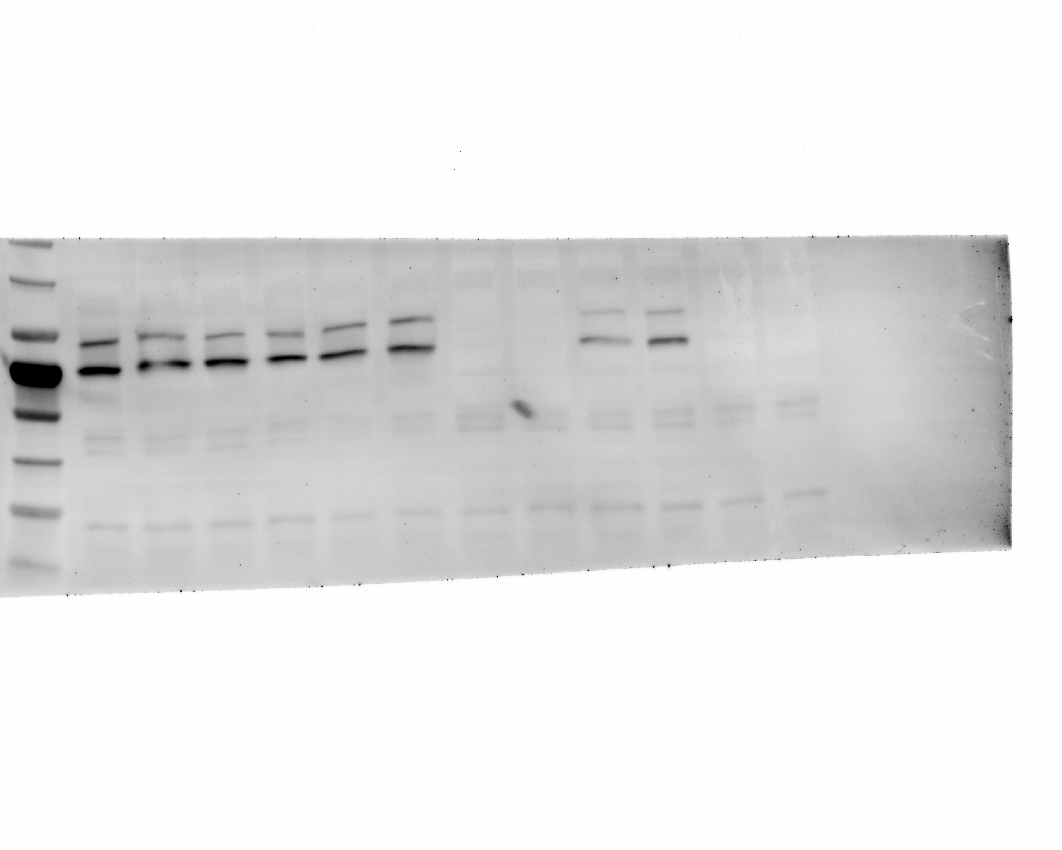

Supplement: Figure 3—source data 1. [file elife-85542-fig3-data1.zip › Figure 3 source data/Figure 3C/Figure 3C-pAkt raw data.jpg]

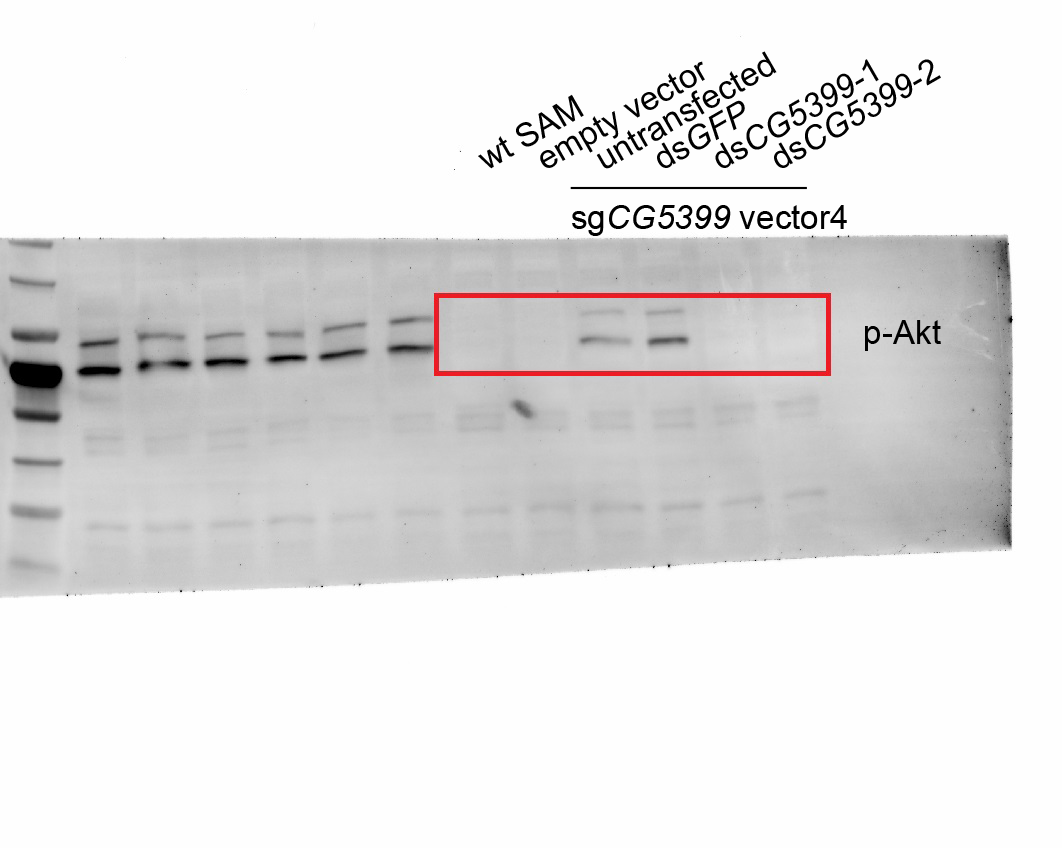

Supplement: Figure 3—source data 1. [file elife-85542-fig3-data1.zip › Figure 3 source data/Figure 3C/Figure 3C-pAkt.tif]

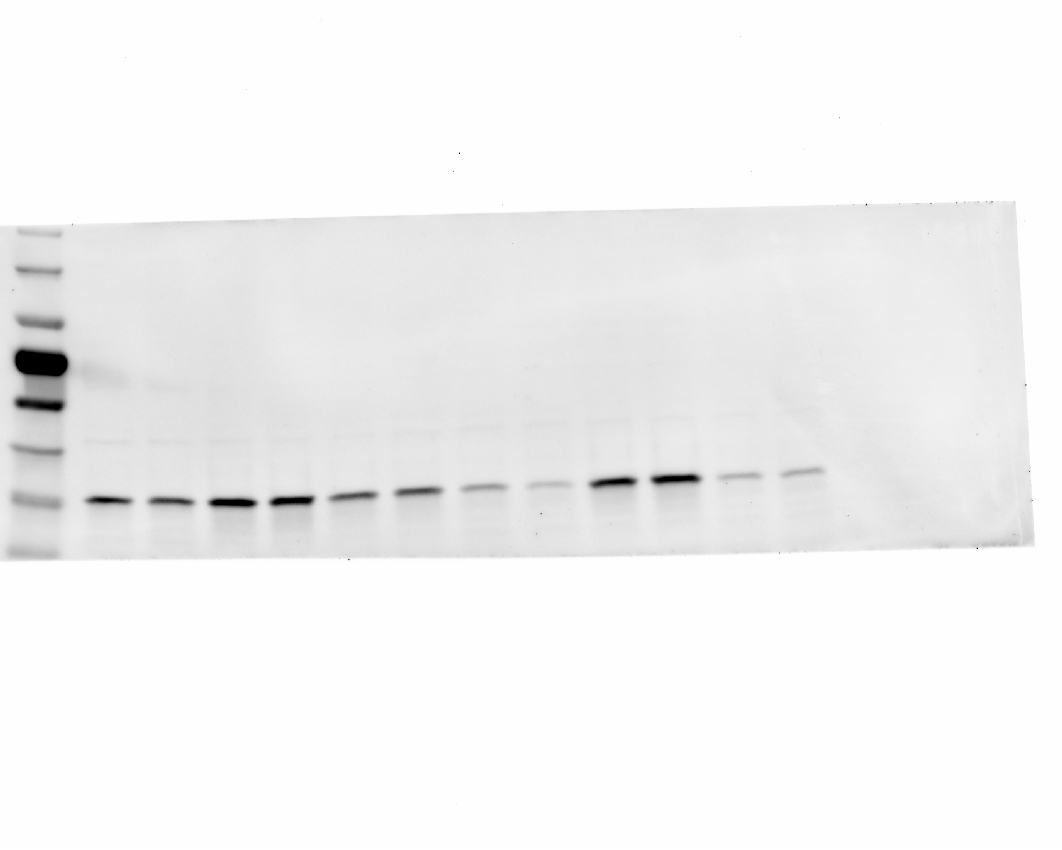

Supplement: Figure 3—source data 1. [file elife-85542-fig3-data1.zip › Figure 3 source data/Figure 3C/Figure 3C-pS6 raw data.jpg]

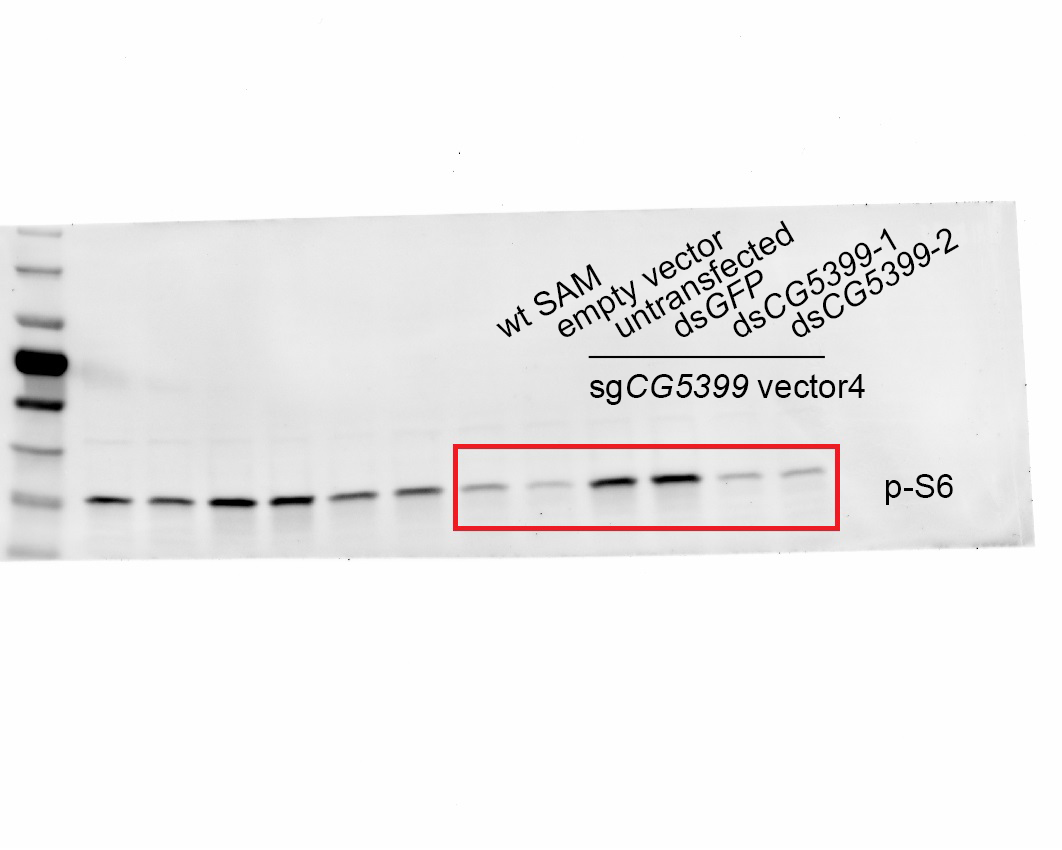

Supplement: Figure 3—source data 1. [file elife-85542-fig3-data1.zip › Figure 3 source data/Figure 3C/Figure 3C-pS6.tif]

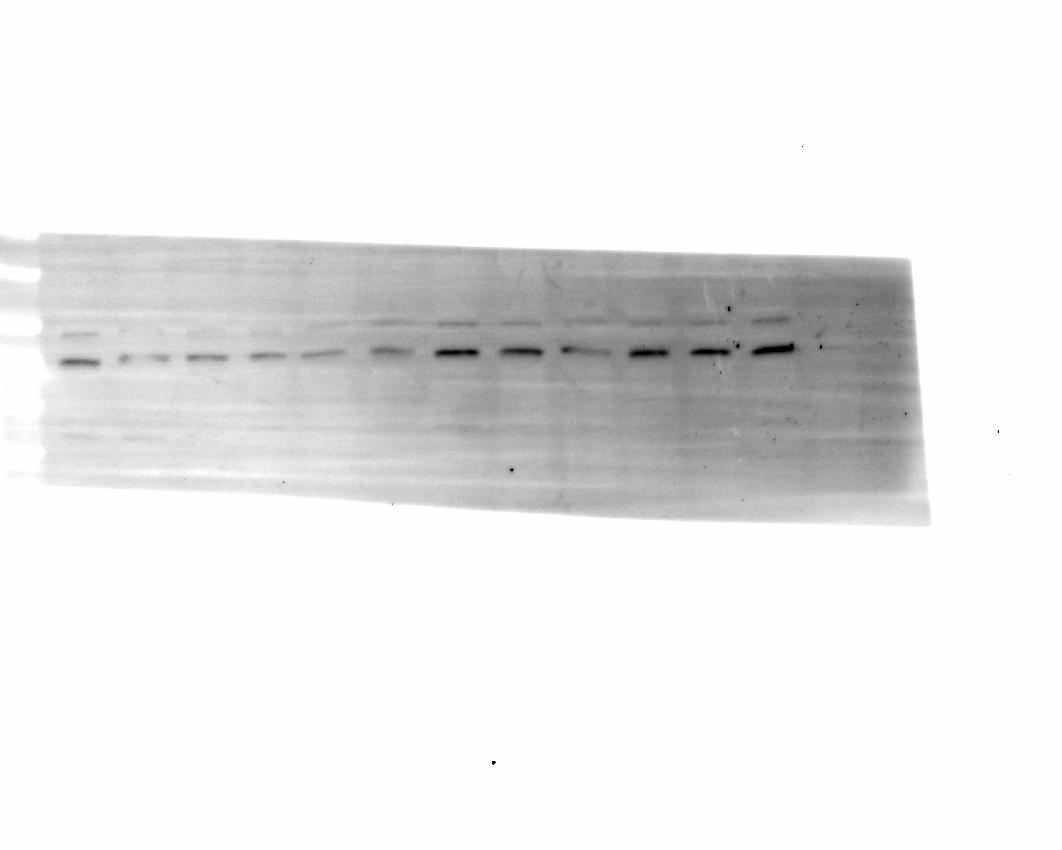

Supplement: Figure 3—source data 1. [file elife-85542-fig3-data1.zip › Figure 3 source data/Figure 3C/Figure 3C-total Akt raw data.tif]

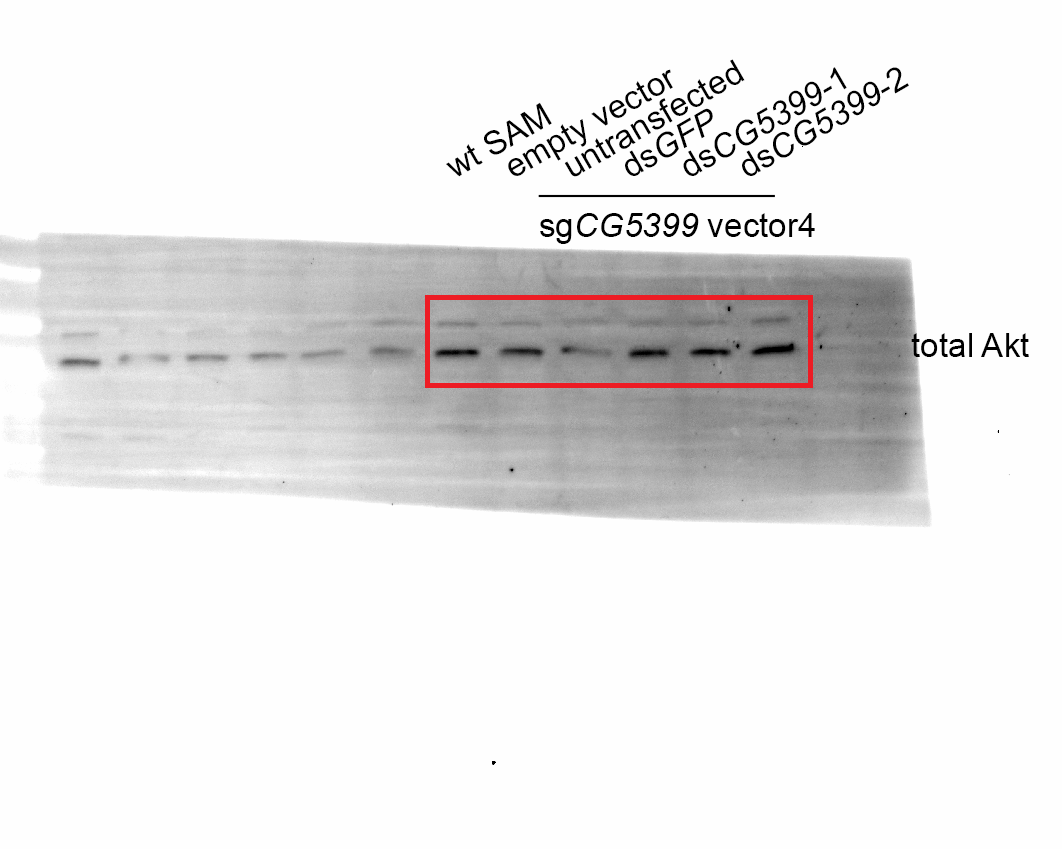

Supplement: Figure 3—source data 1. [file elife-85542-fig3-data1.zip › Figure 3 source data/Figure 3C/Figure 3C-total Akt.tif]

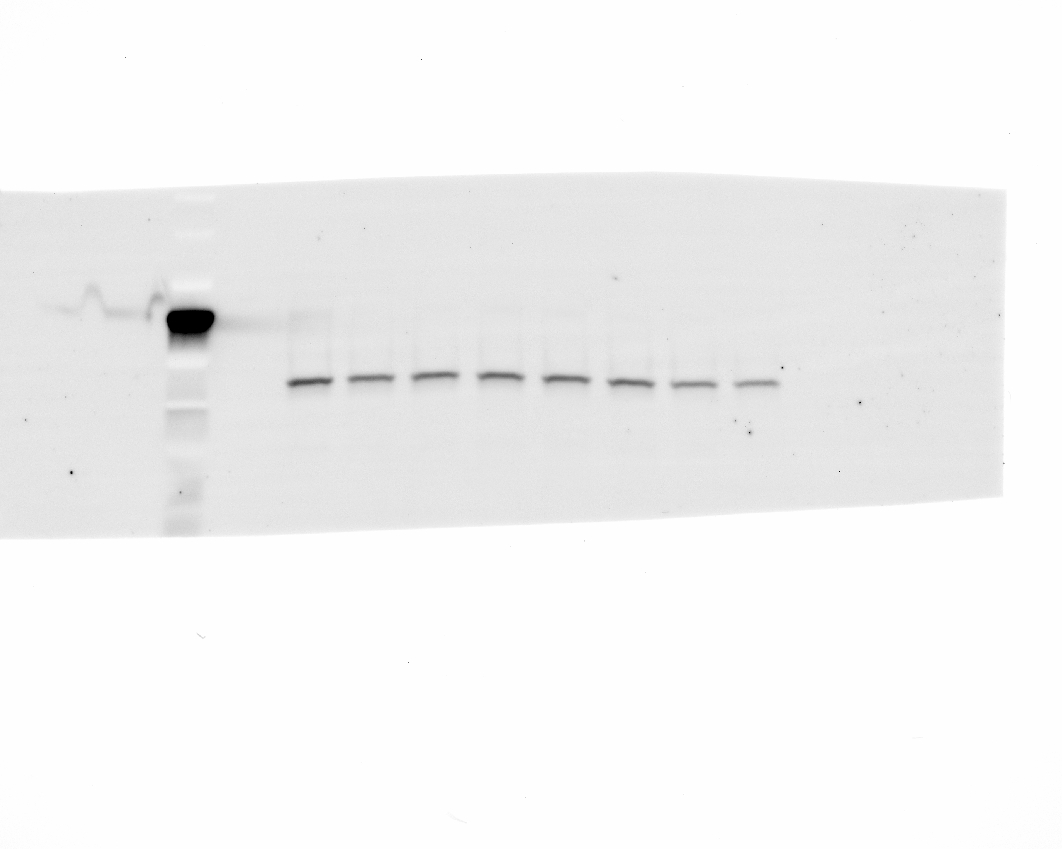

Supplement: Figure 3—source data 1. [file elife-85542-fig3-data1.zip › Figure 3 source data/Figure 3D/Figure 3D-actin for pAkt blot raw data.jpg]

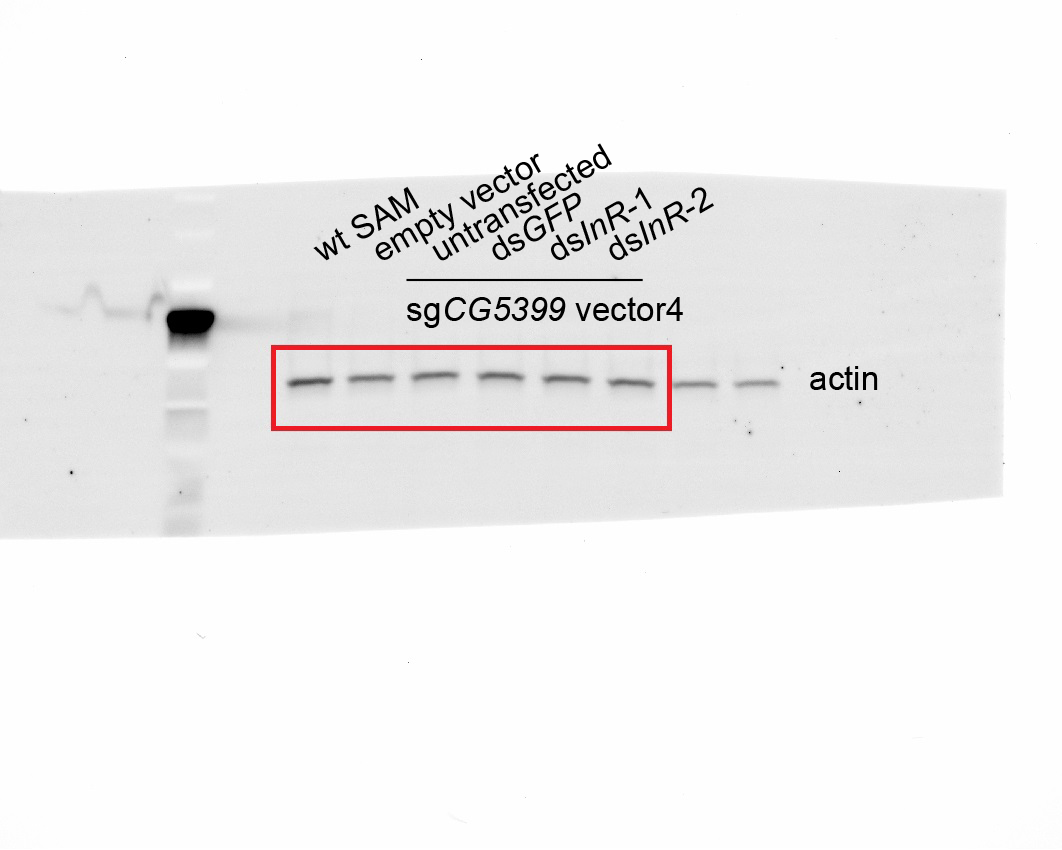

Supplement: Figure 3—source data 1. [file elife-85542-fig3-data1.zip › Figure 3 source data/Figure 3D/Figure 3D-actin for pAkt blot.tif]

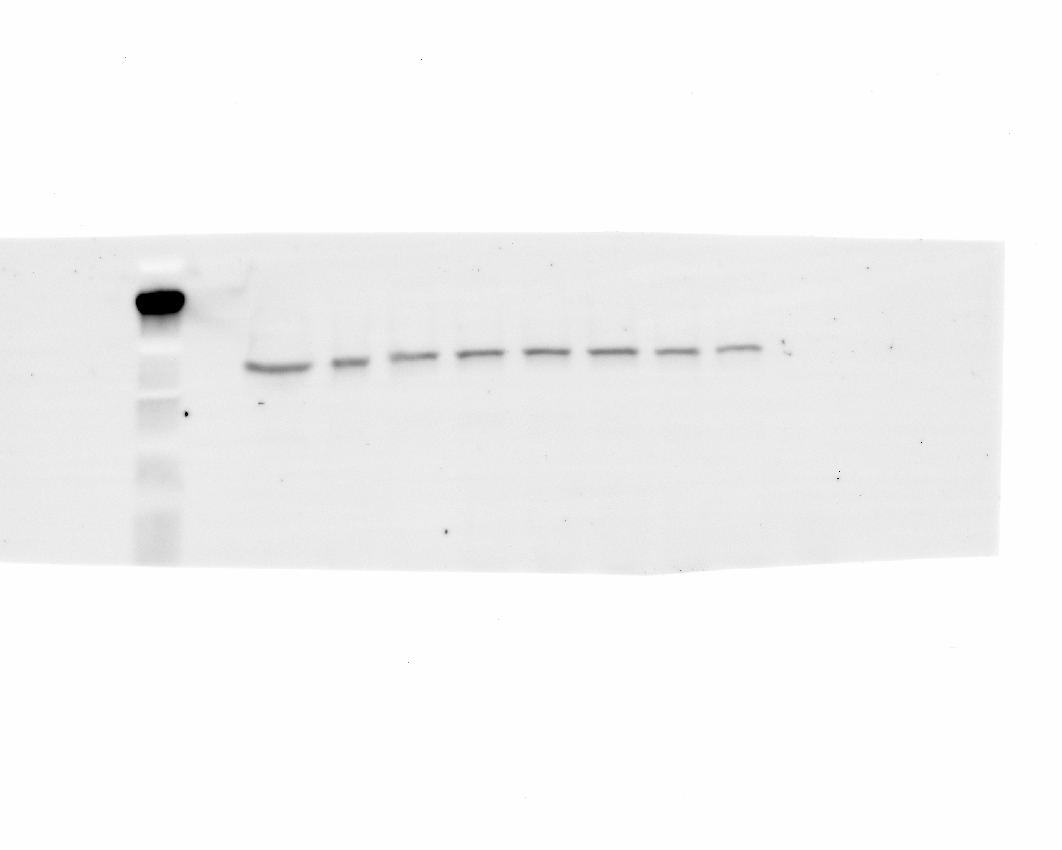

Supplement: Figure 3—source data 1. [file elife-85542-fig3-data1.zip › Figure 3 source data/Figure 3D/Figure 3D-actin for pS6 blot raw data.jpg]

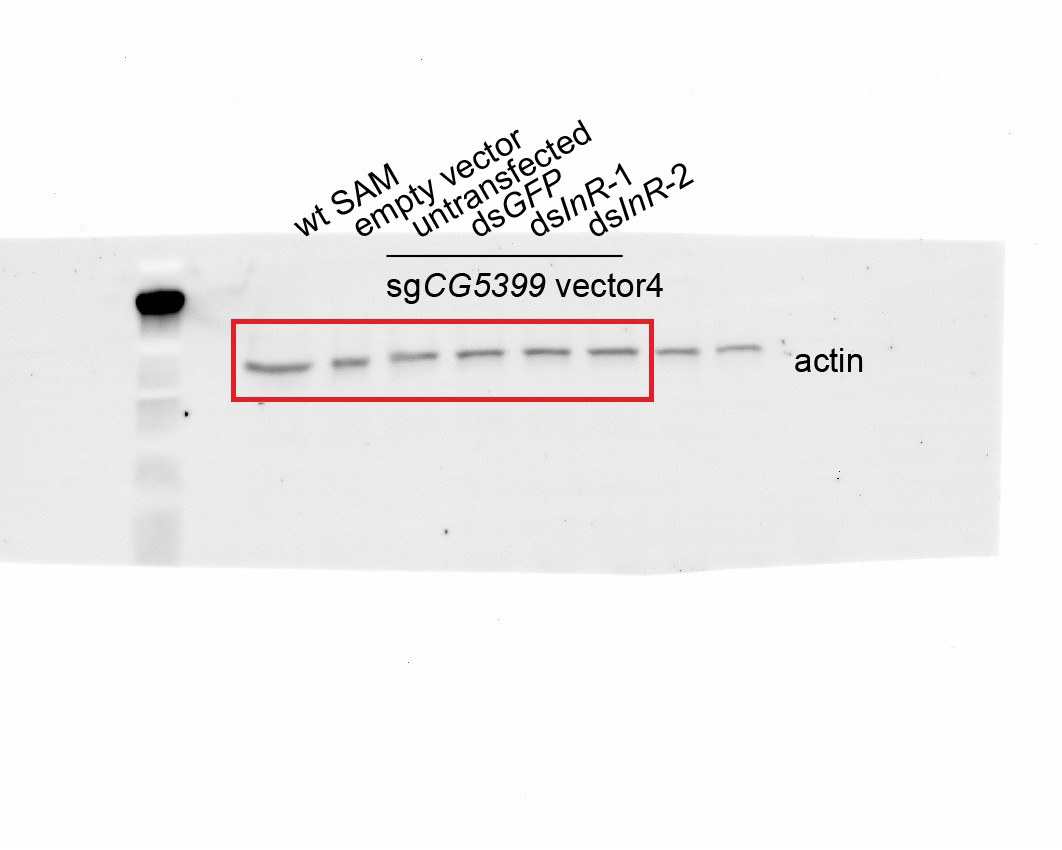

Supplement: Figure 3—source data 1. [file elife-85542-fig3-data1.zip › Figure 3 source data/Figure 3D/Figure 3D-actin for pS6 blot.tif]

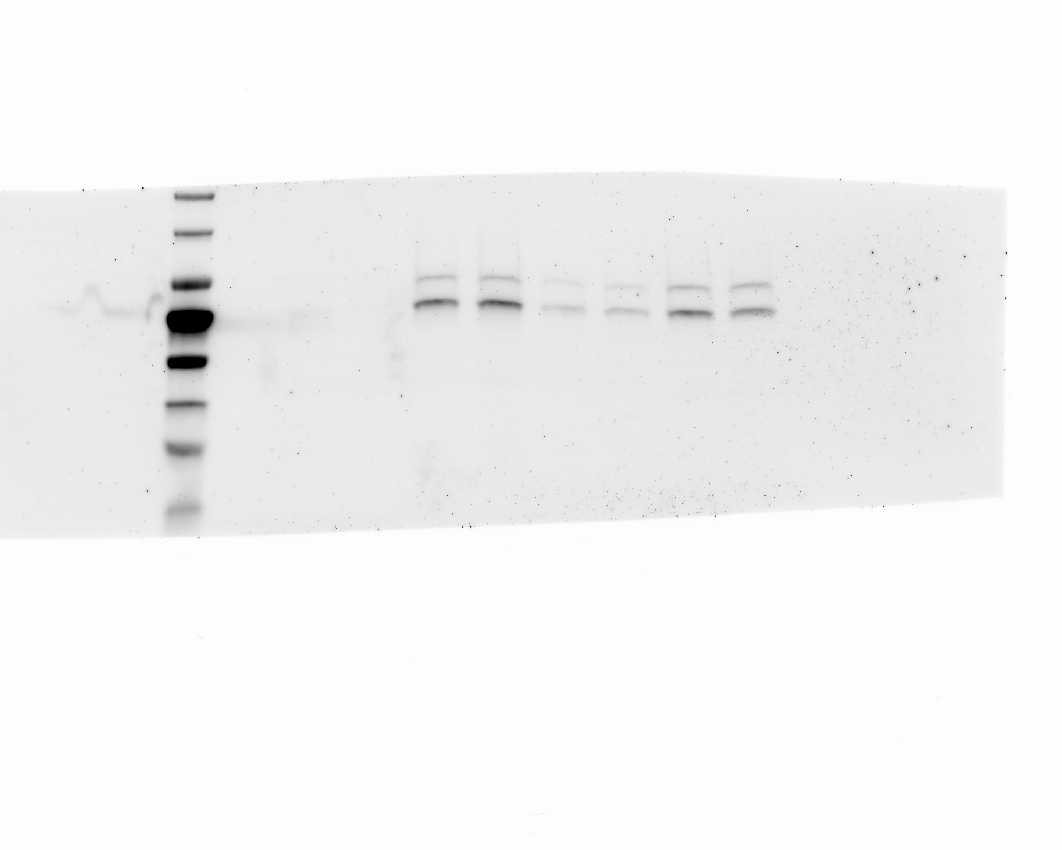

Supplement: Figure 3—source data 1. [file elife-85542-fig3-data1.zip › Figure 3 source data/Figure 3D/Figure 3D-pAkt raw data.jpg]

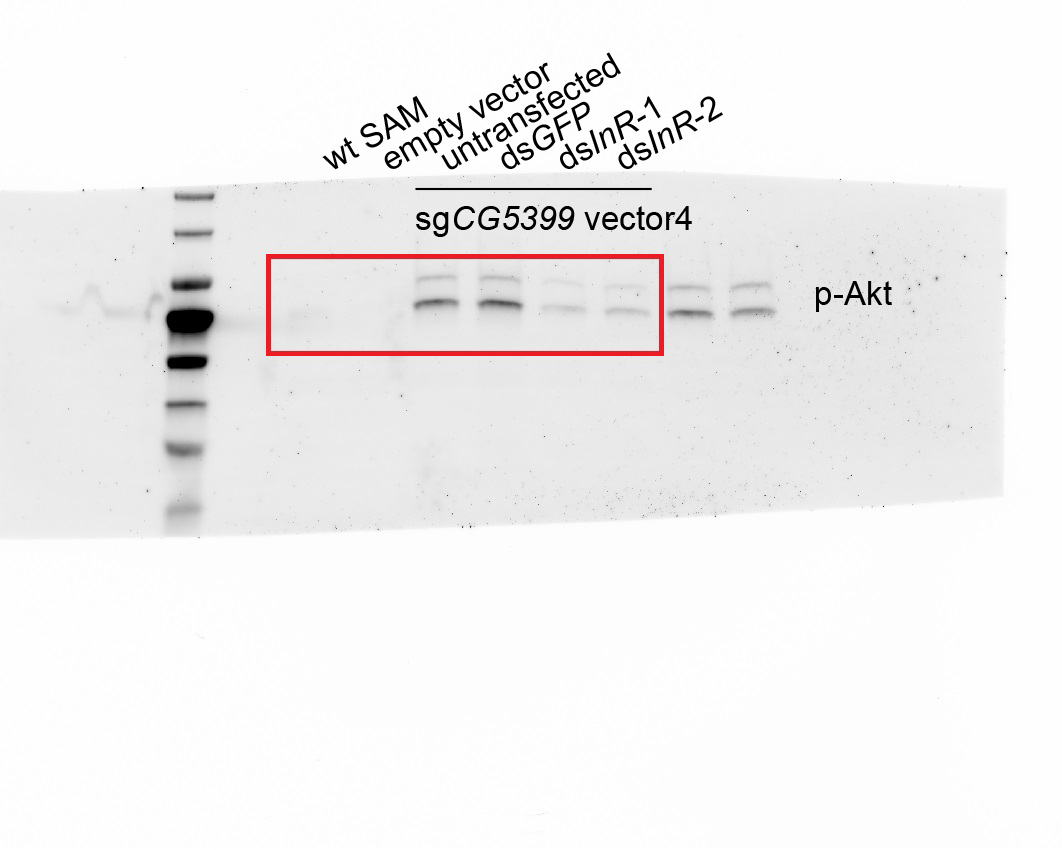

Supplement: Figure 3—source data 1. [file elife-85542-fig3-data1.zip › Figure 3 source data/Figure 3D/Figure 3D-pAkt.tif]

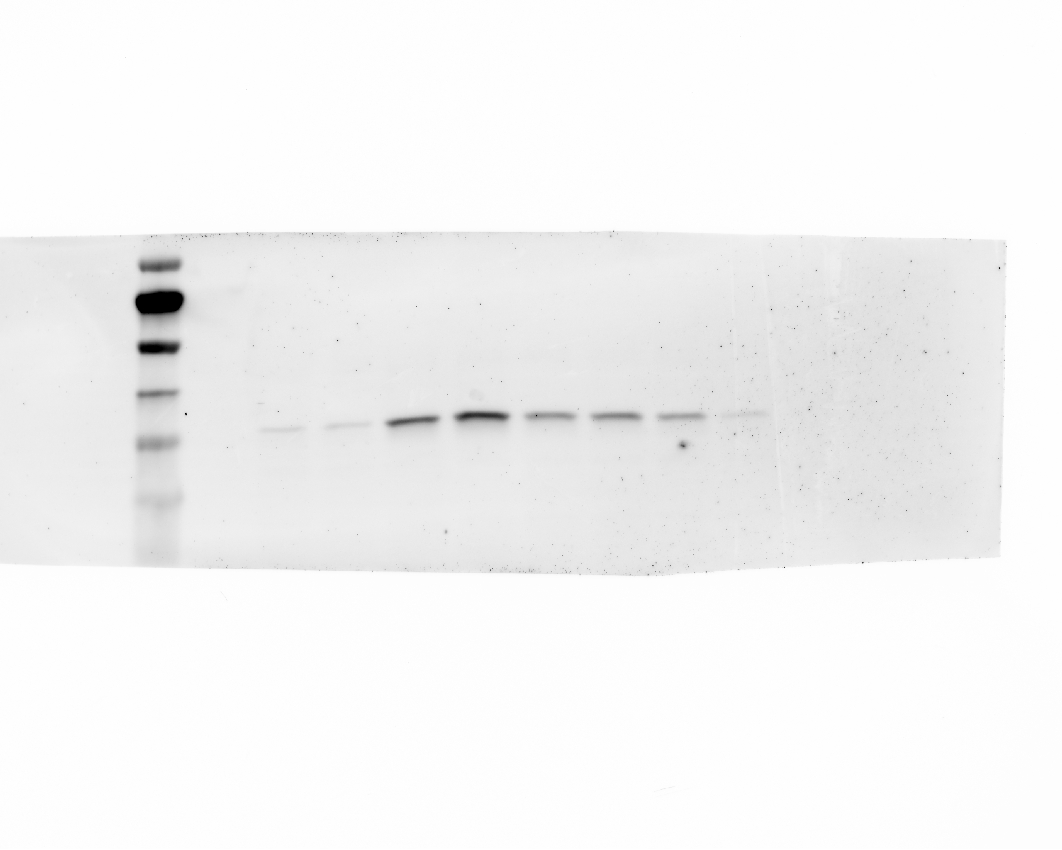

Supplement: Figure 3—source data 1. [file elife-85542-fig3-data1.zip › Figure 3 source data/Figure 3D/Figure 3D-pS6 raw data.jpg]

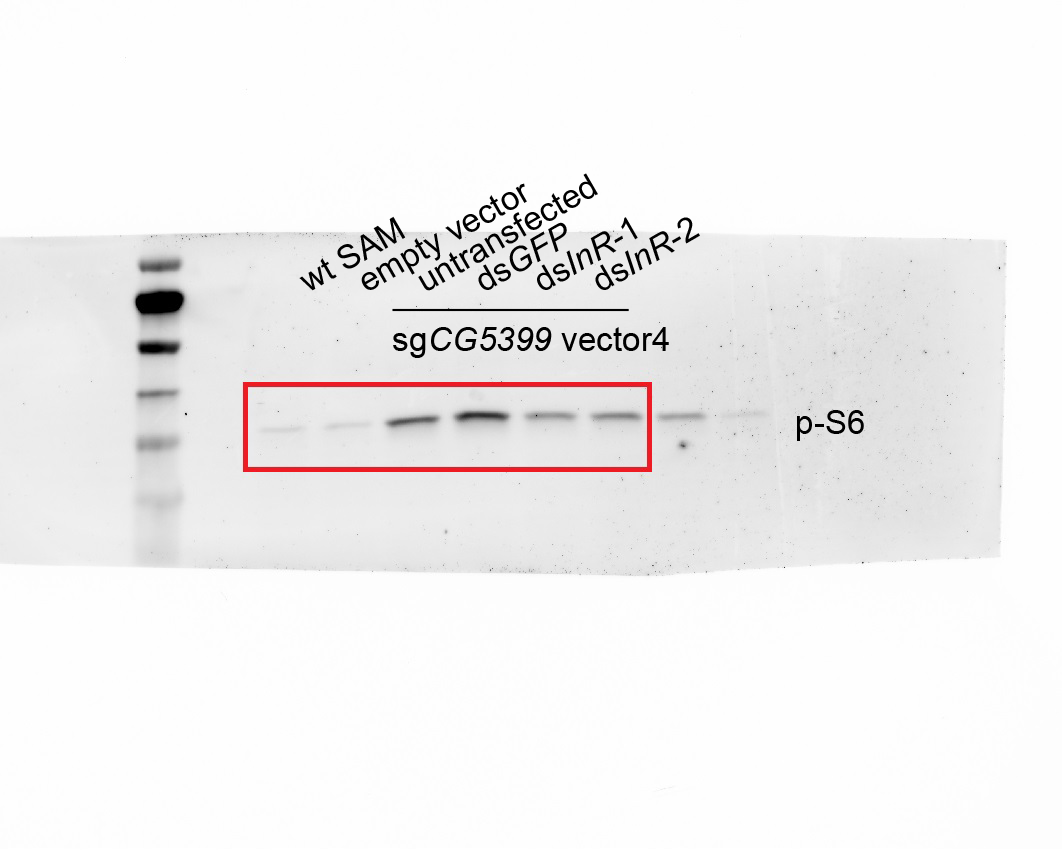

Supplement: Figure 3—source data 1. [file elife-85542-fig3-data1.zip › Figure 3 source data/Figure 3D/Figure 3D-pS6.tif]

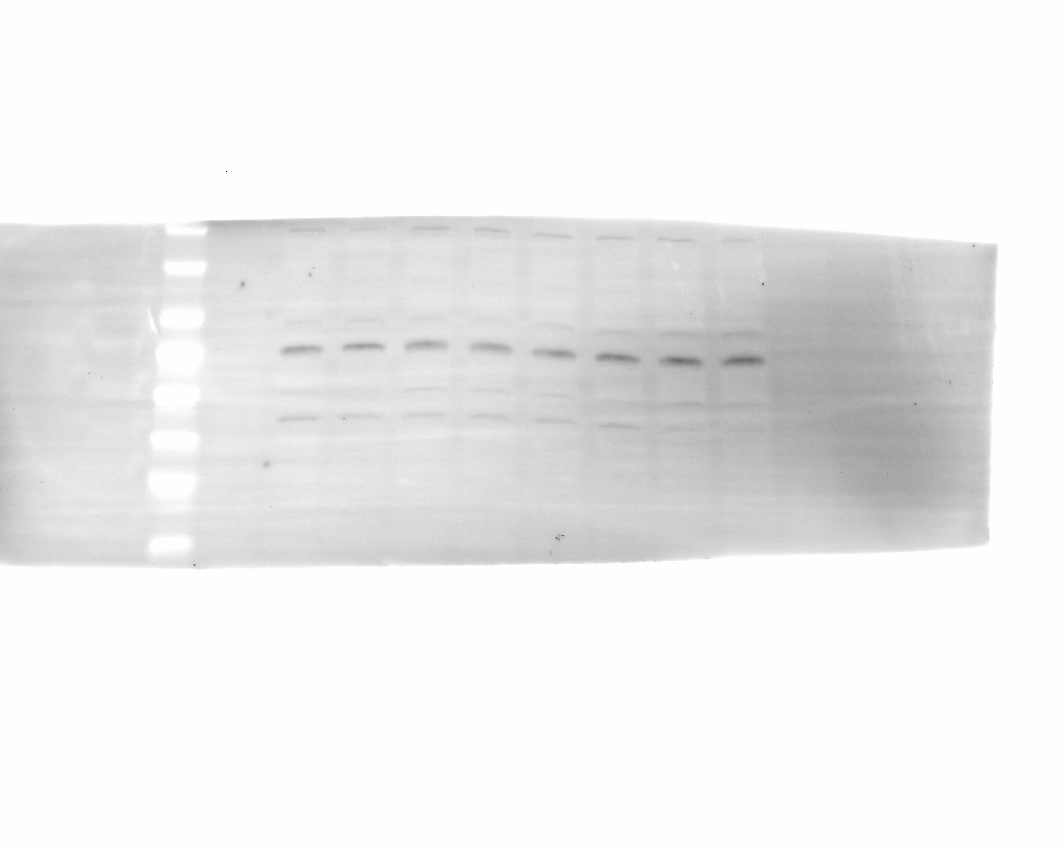

Supplement: Figure 3—source data 1. [file elife-85542-fig3-data1.zip › Figure 3 source data/Figure 3D/Figure 3D-total Akt raw data.jpg]

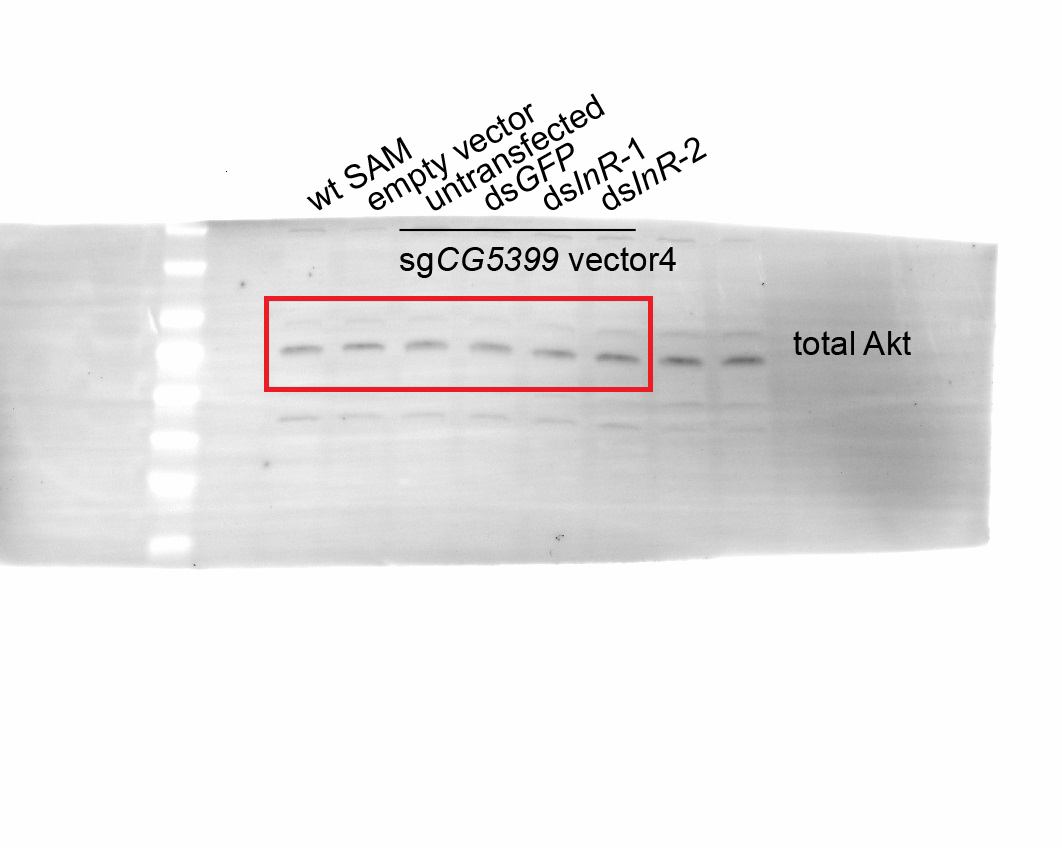

Supplement: Figure 3—source data 1. [file elife-85542-fig3-data1.zip › Figure 3 source data/Figure 3D/Figure 3D-total Akt.tif]

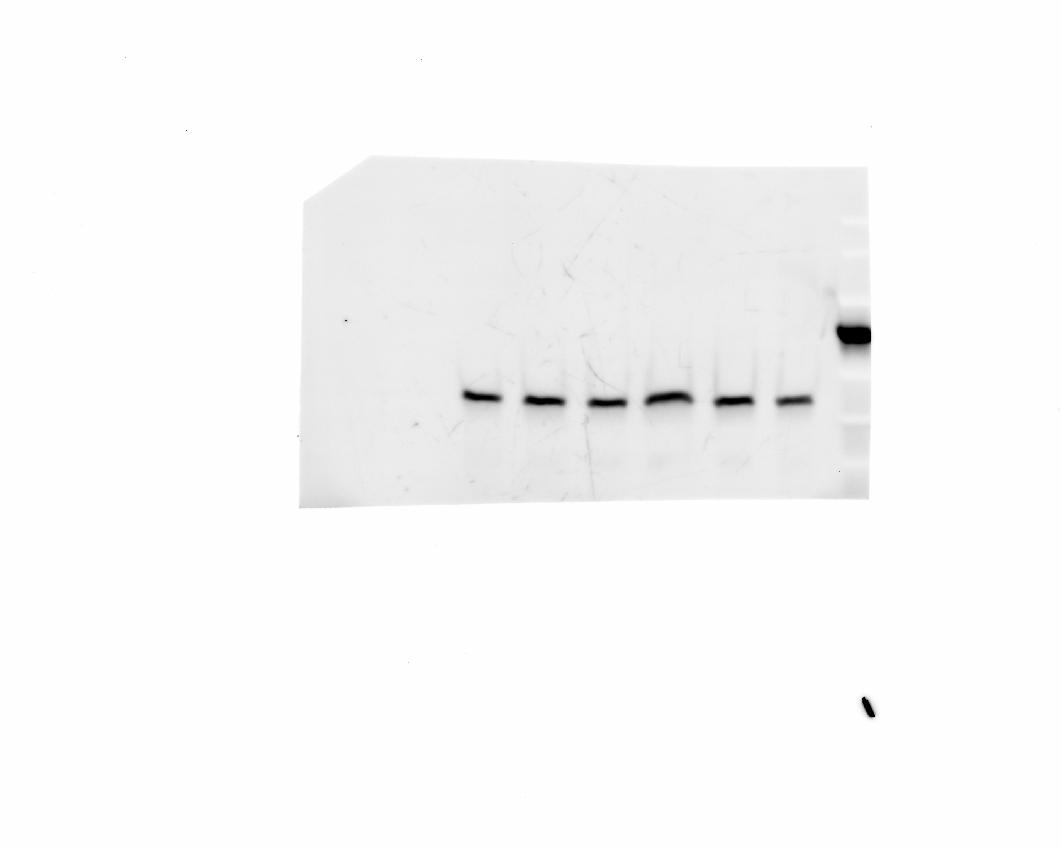

Supplement: Figure 3—source data 1. [file elife-85542-fig3-data1.zip › Figure 3 source data/Figure 3E/Figure 3E-actin for pAkt blot raw data.jpg]

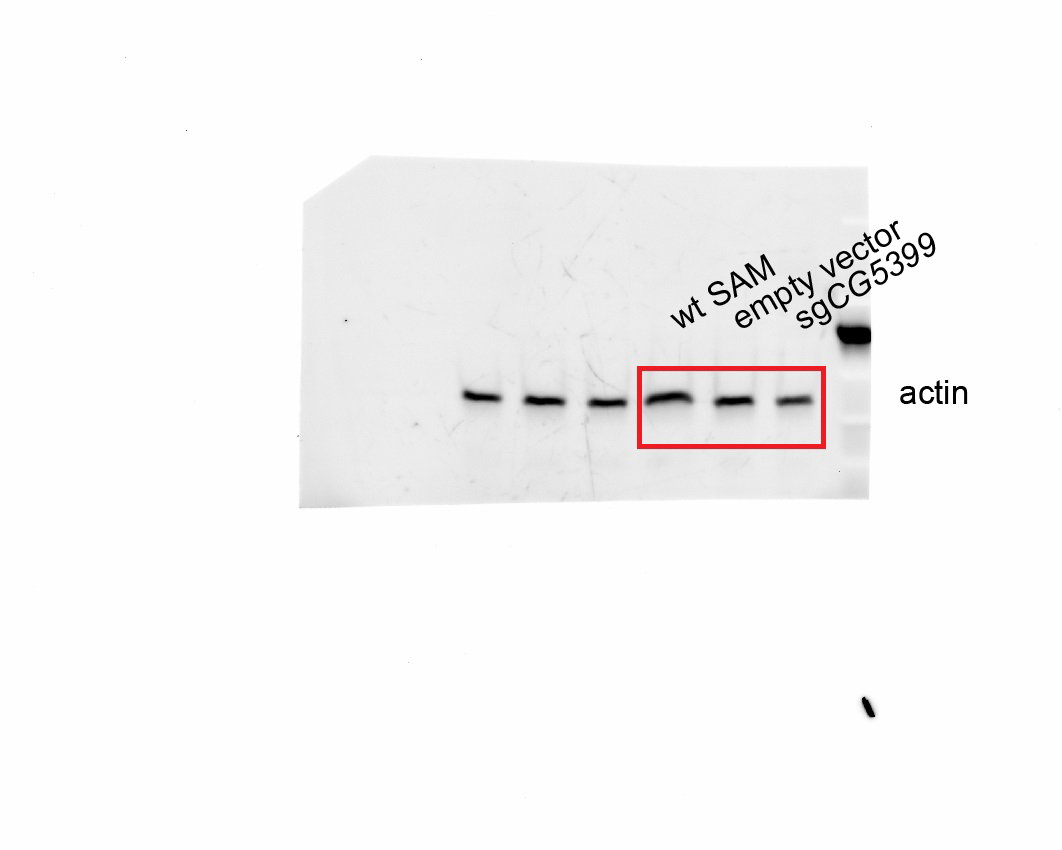

Supplement: Figure 3—source data 1. [file elife-85542-fig3-data1.zip › Figure 3 source data/Figure 3E/Figure 3E-actin for pAkt blot.tif]

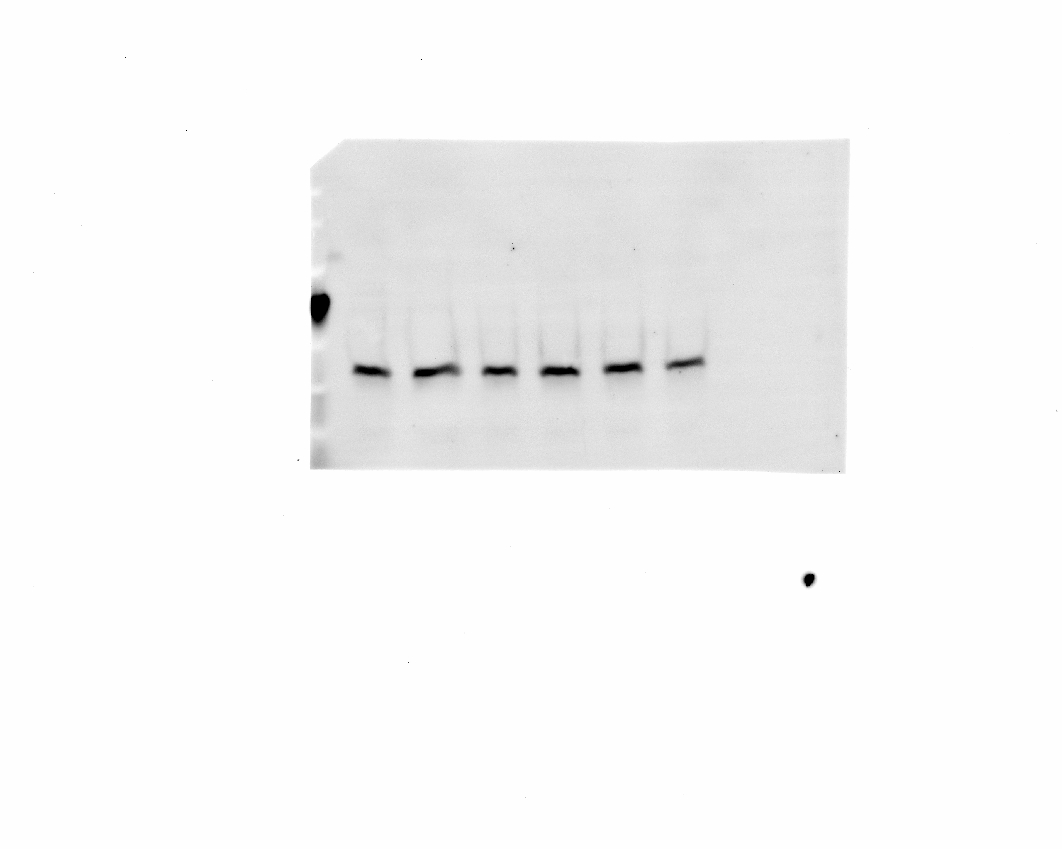

Supplement: Figure 3—source data 1. [file elife-85542-fig3-data1.zip › Figure 3 source data/Figure 3E/Figure 3E-actin for pInR blot raw data.jpg]

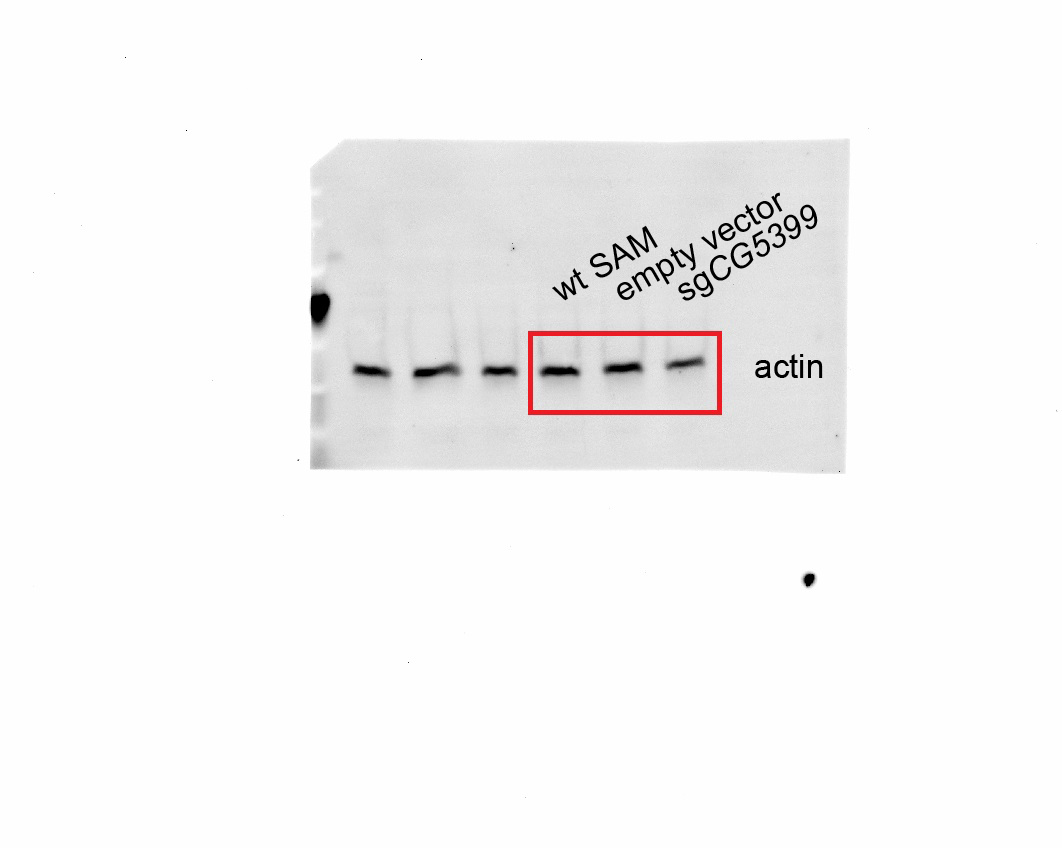

Supplement: Figure 3—source data 1. [file elife-85542-fig3-data1.zip › Figure 3 source data/Figure 3E/Figure 3E-actin for pInR blot.tif]

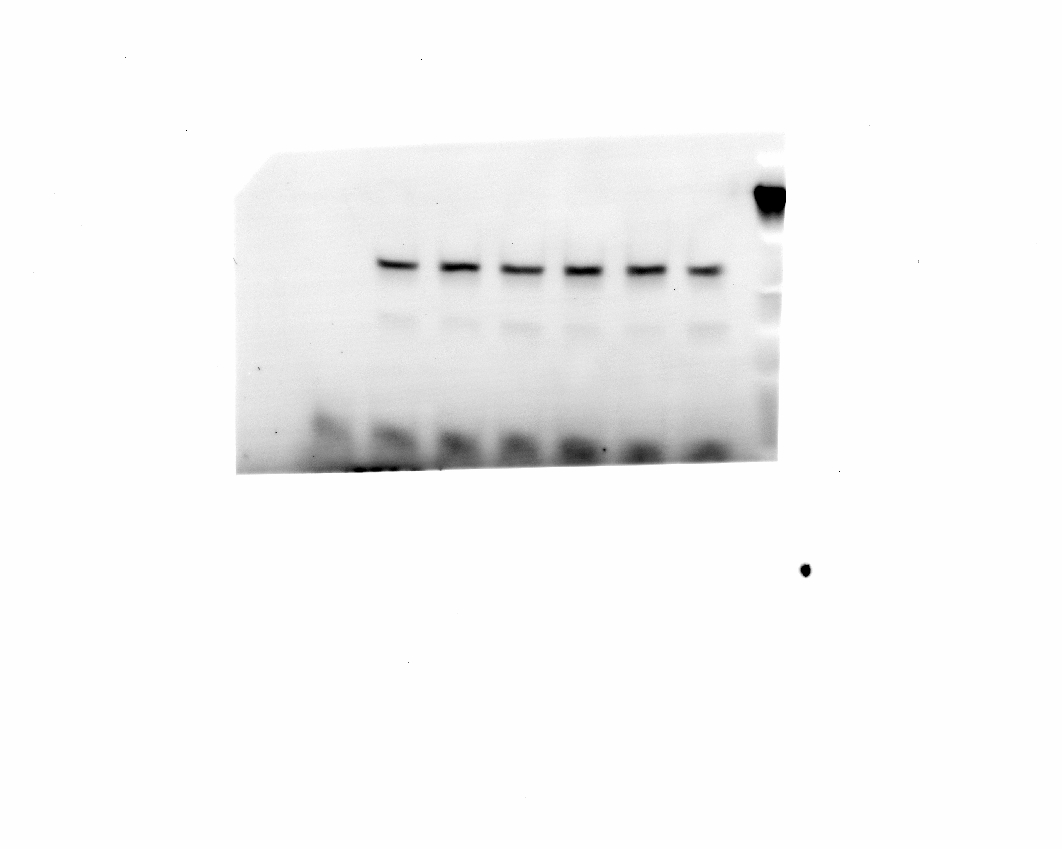

Supplement: Figure 3—source data 1. [file elife-85542-fig3-data1.zip › Figure 3 source data/Figure 3E/Figure 3E-actin for pS6 blot raw data.jpg]

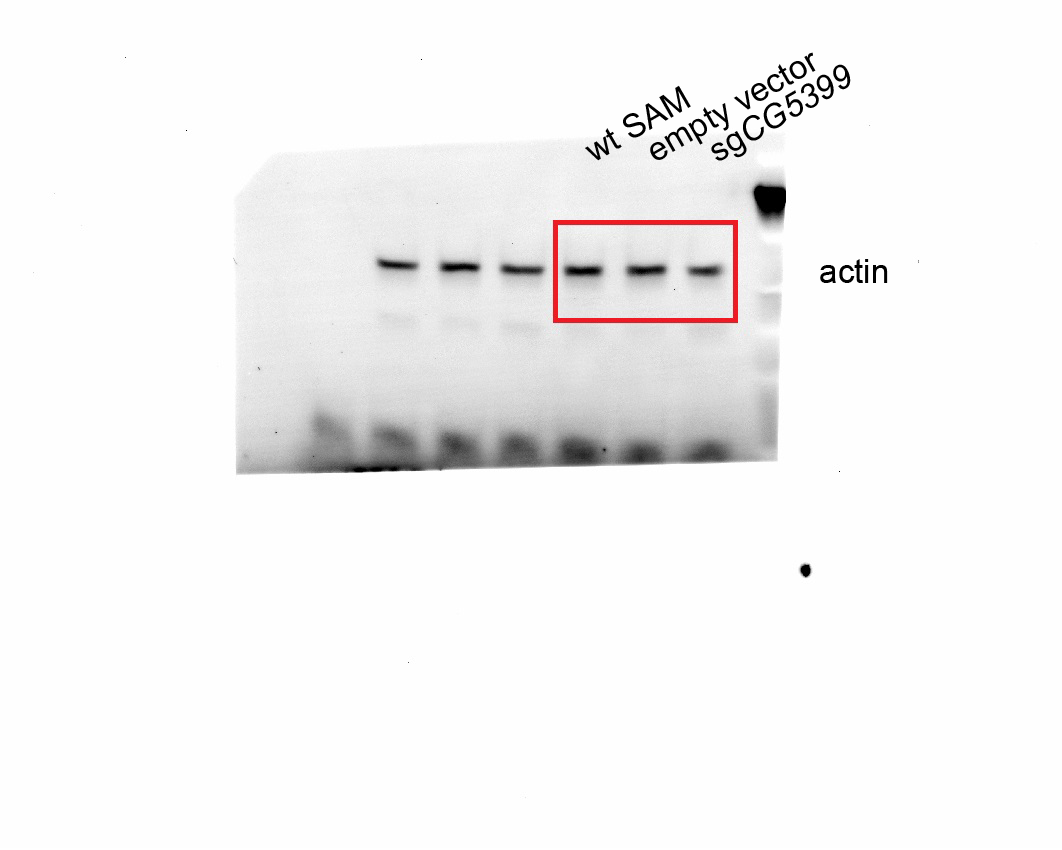

Supplement: Figure 3—source data 1. [file elife-85542-fig3-data1.zip › Figure 3 source data/Figure 3E/Figure 3E-actin for pS6 blot.tif]

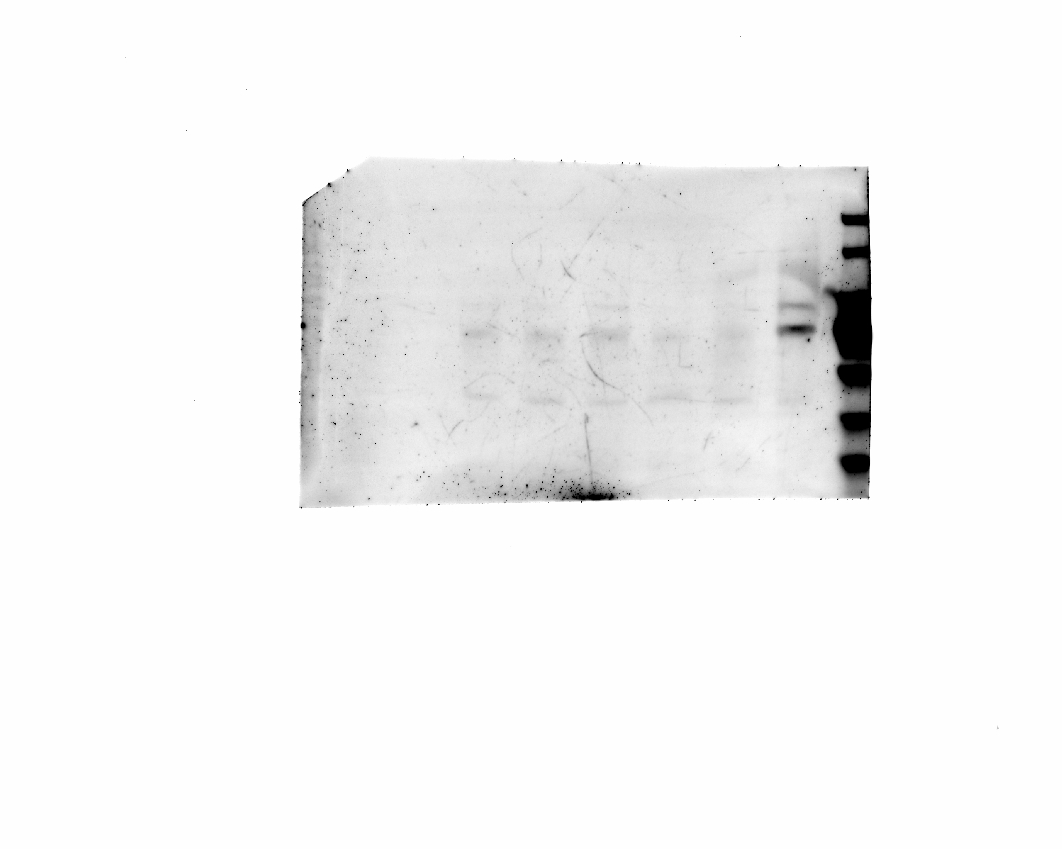

Supplement: Figure 3—source data 1. [file elife-85542-fig3-data1.zip › Figure 3 source data/Figure 3E/Figure 3E-pAkt raw data.jpg]

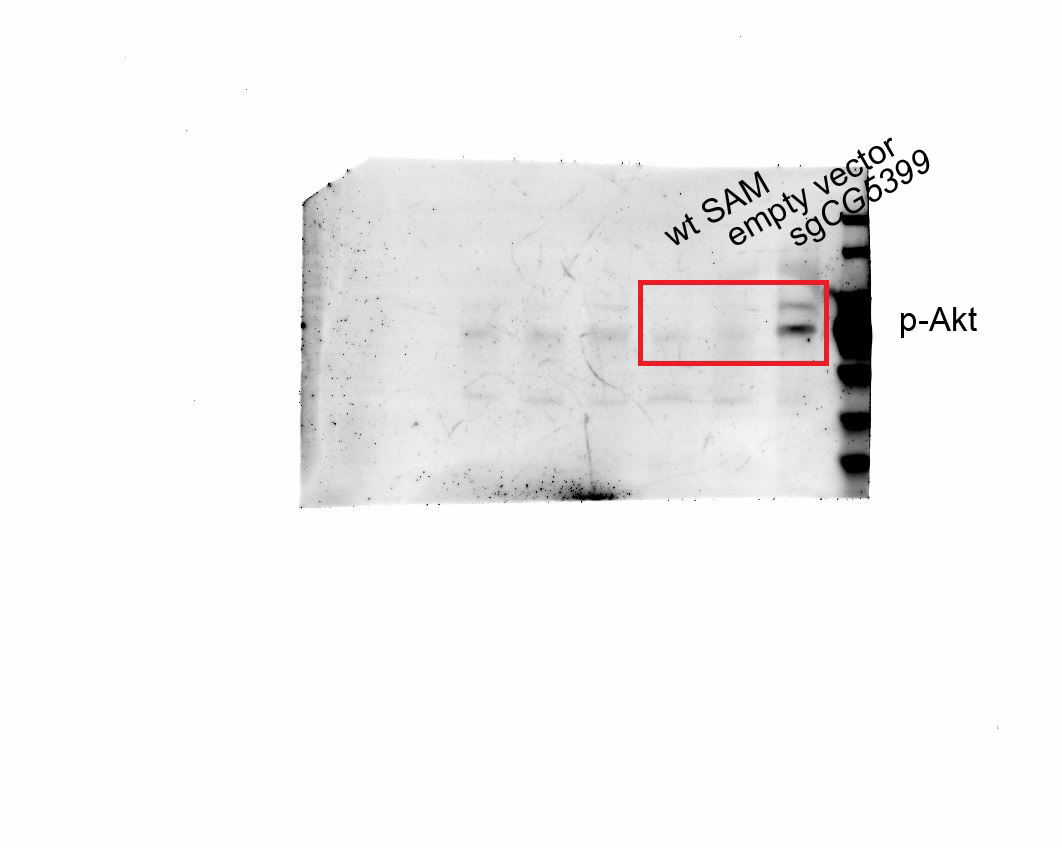

Supplement: Figure 3—source data 1. [file elife-85542-fig3-data1.zip › Figure 3 source data/Figure 3E/Figure 3E-pAkt.tif]

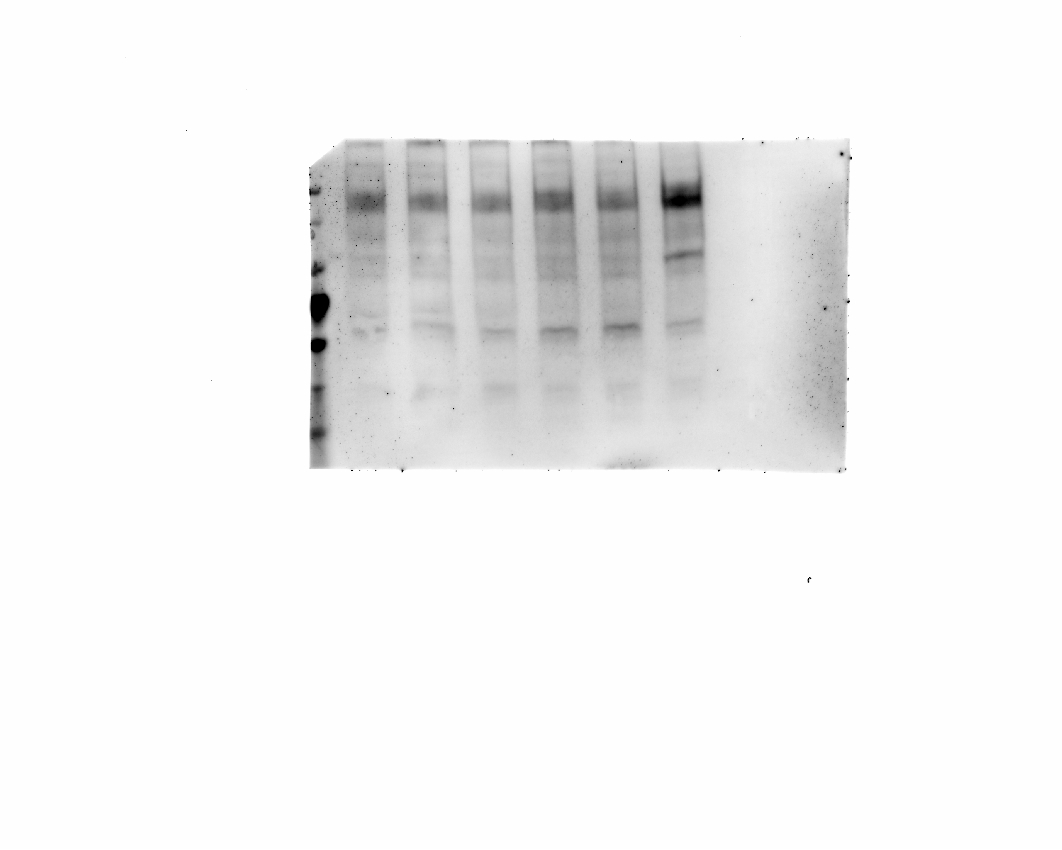

Supplement: Figure 3—source data 1. [file elife-85542-fig3-data1.zip › Figure 3 source data/Figure 3E/Figure 3E-pInR raw data.jpg]

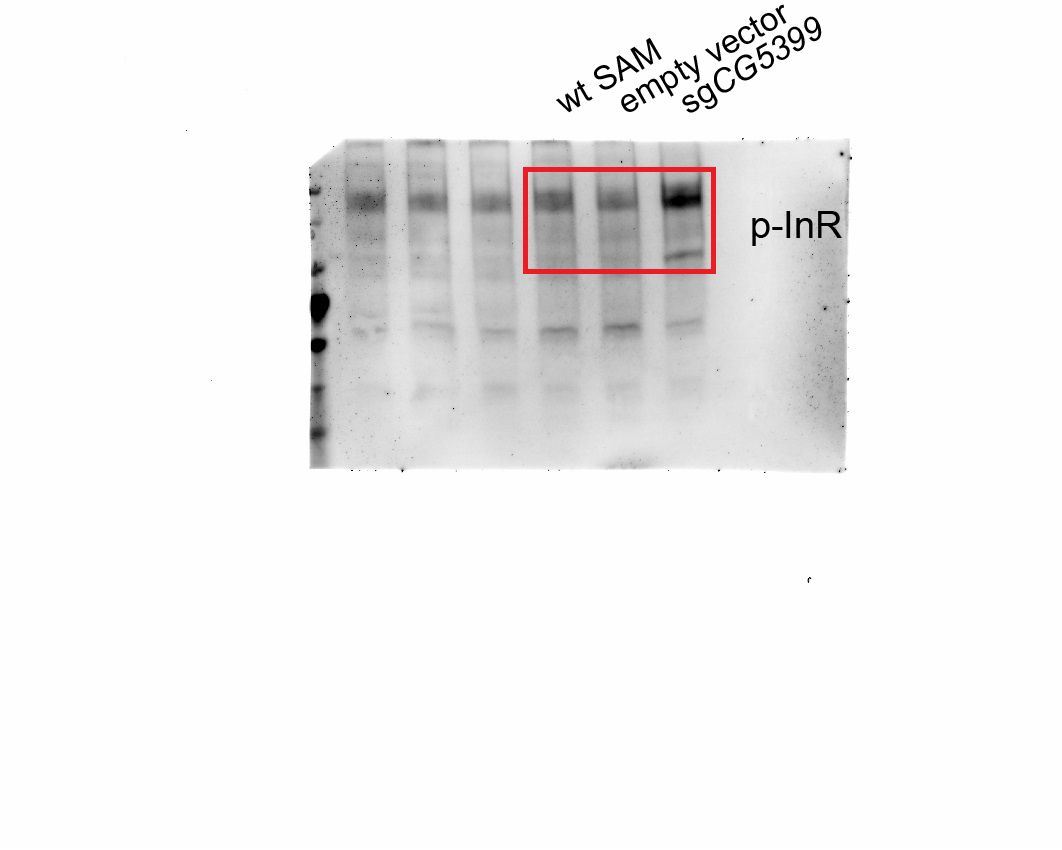

Supplement: Figure 3—source data 1. [file elife-85542-fig3-data1.zip › Figure 3 source data/Figure 3E/Figure 3E-pInR.tif]

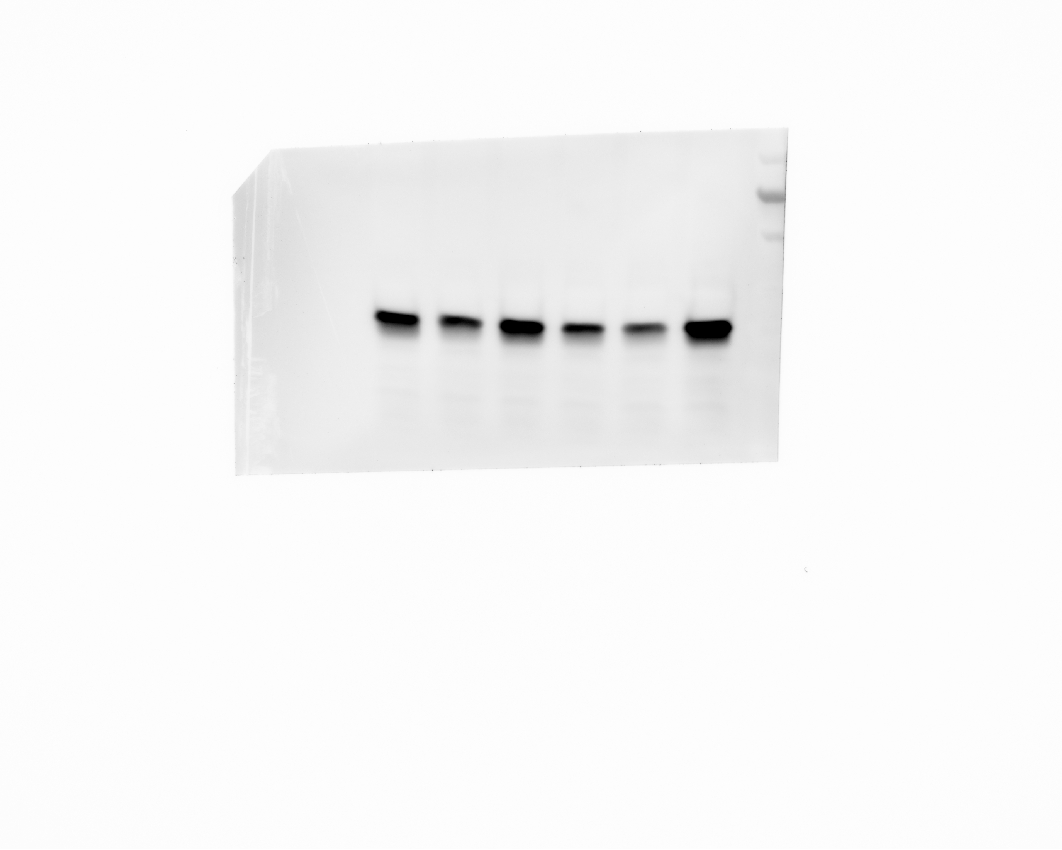

Supplement: Figure 3—source data 1. [file elife-85542-fig3-data1.zip › Figure 3 source data/Figure 3E/Figure 3E-pS6 raw data.jpg]

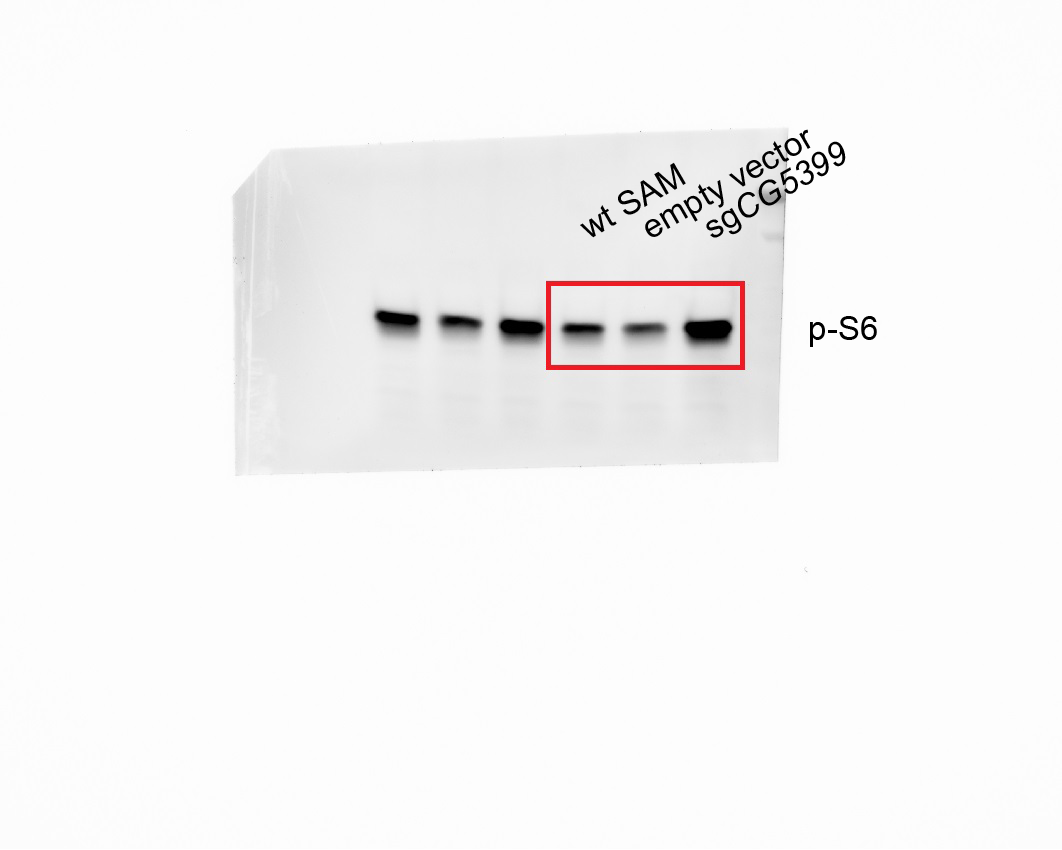

Supplement: Figure 3—source data 1. [file elife-85542-fig3-data1.zip › Figure 3 source data/Figure 3E/Figure 3E-pS6.tif]

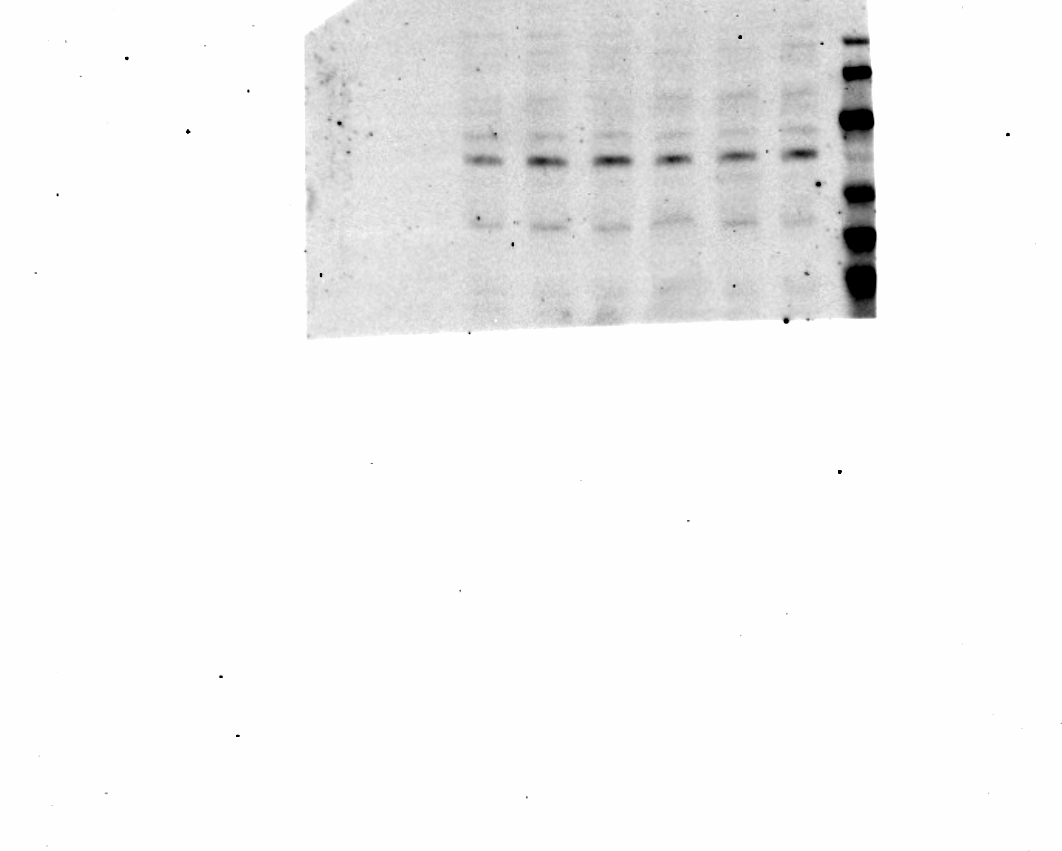

Supplement: Figure 3—source data 1. [file elife-85542-fig3-data1.zip › Figure 3 source data/Figure 3E/Figure 3E-total Akt raw data.jpg]

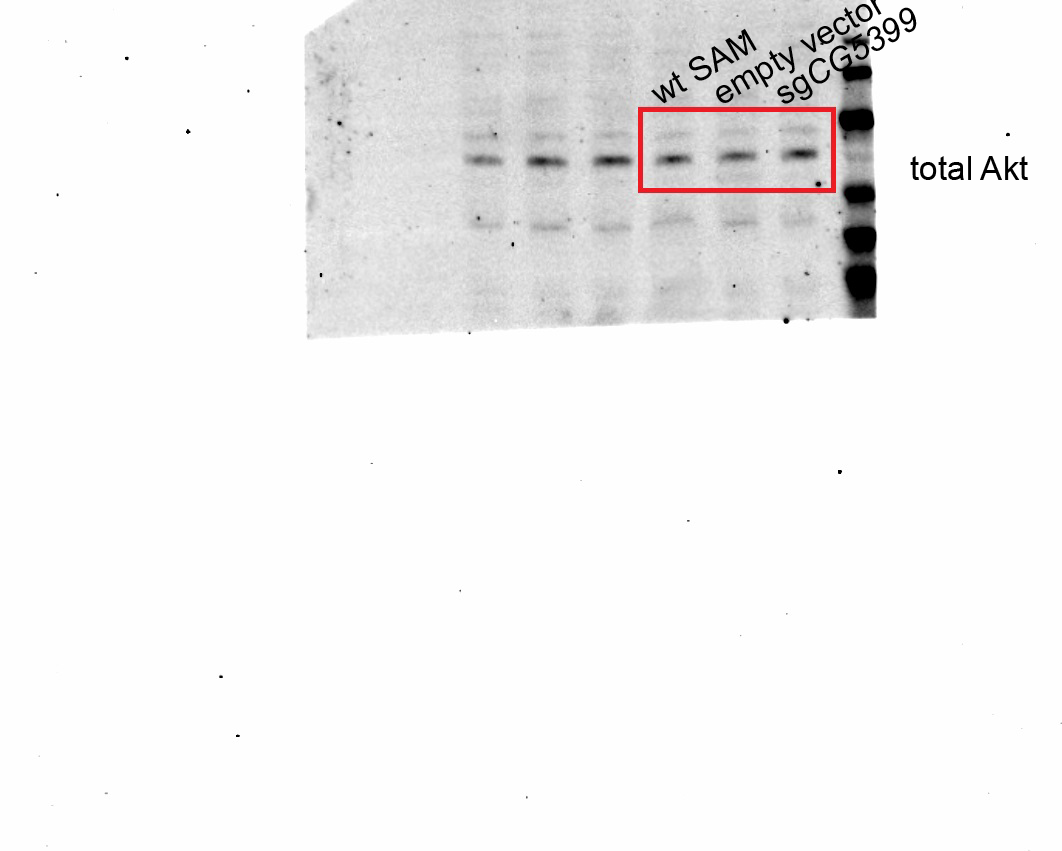

Supplement: Figure 3—source data 1. [file elife-85542-fig3-data1.zip › Figure 3 source data/Figure 3E/Figure 3E-total Akt.tif]

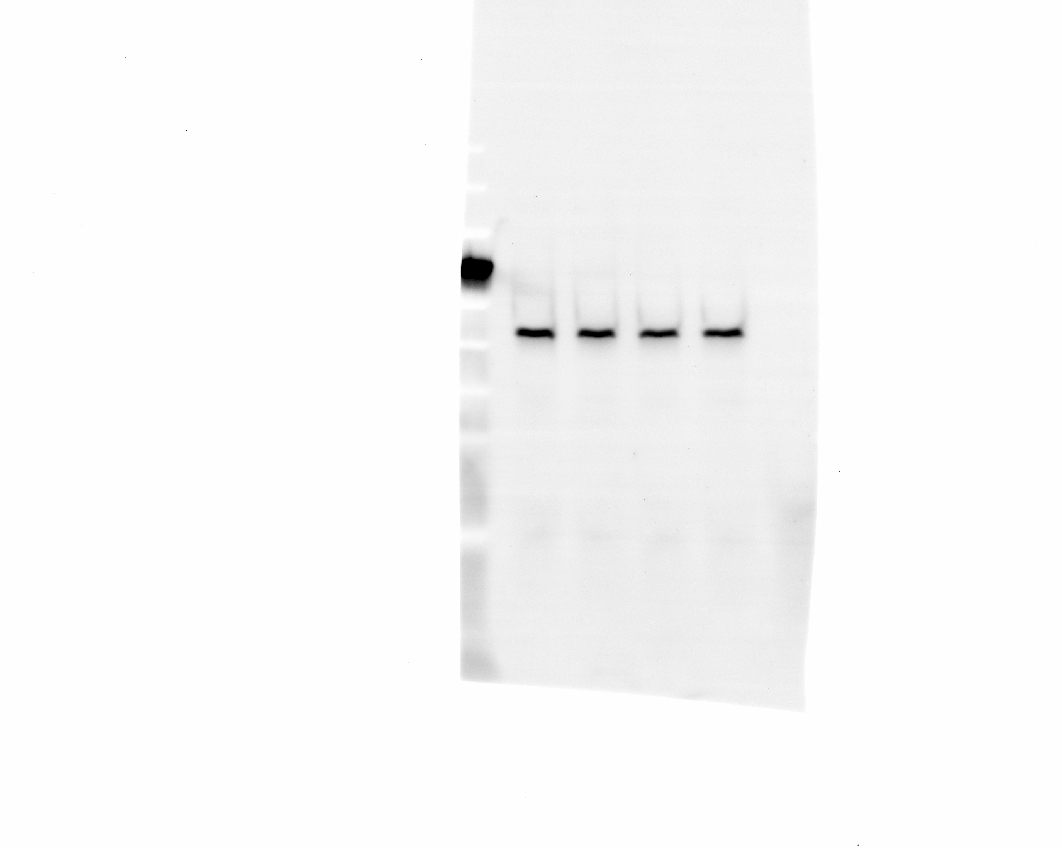

Supplement: Figure 3—source data 1. [file elife-85542-fig3-data1.zip › Figure 3 source data/Figure 3F/Figure 3F-actin for pAkt blot raw data.jpg]

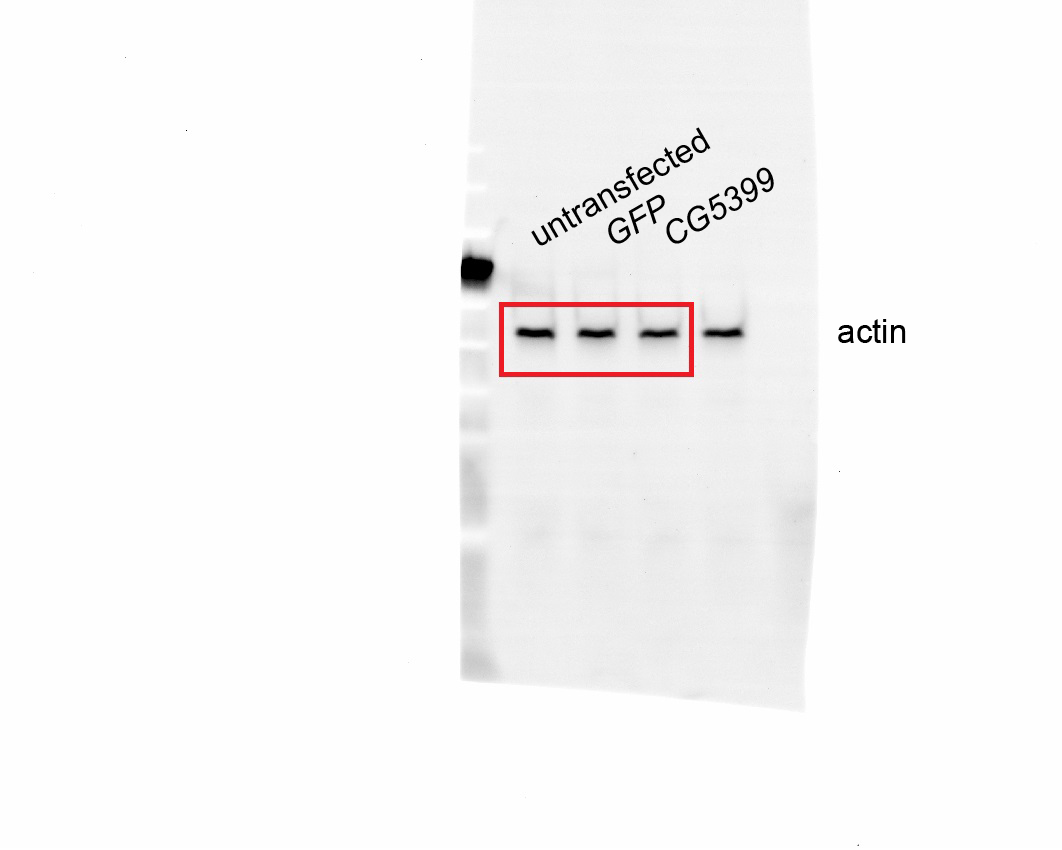

Supplement: Figure 3—source data 1. [file elife-85542-fig3-data1.zip › Figure 3 source data/Figure 3F/Figure 3F-actin for pAkt blot.tif]

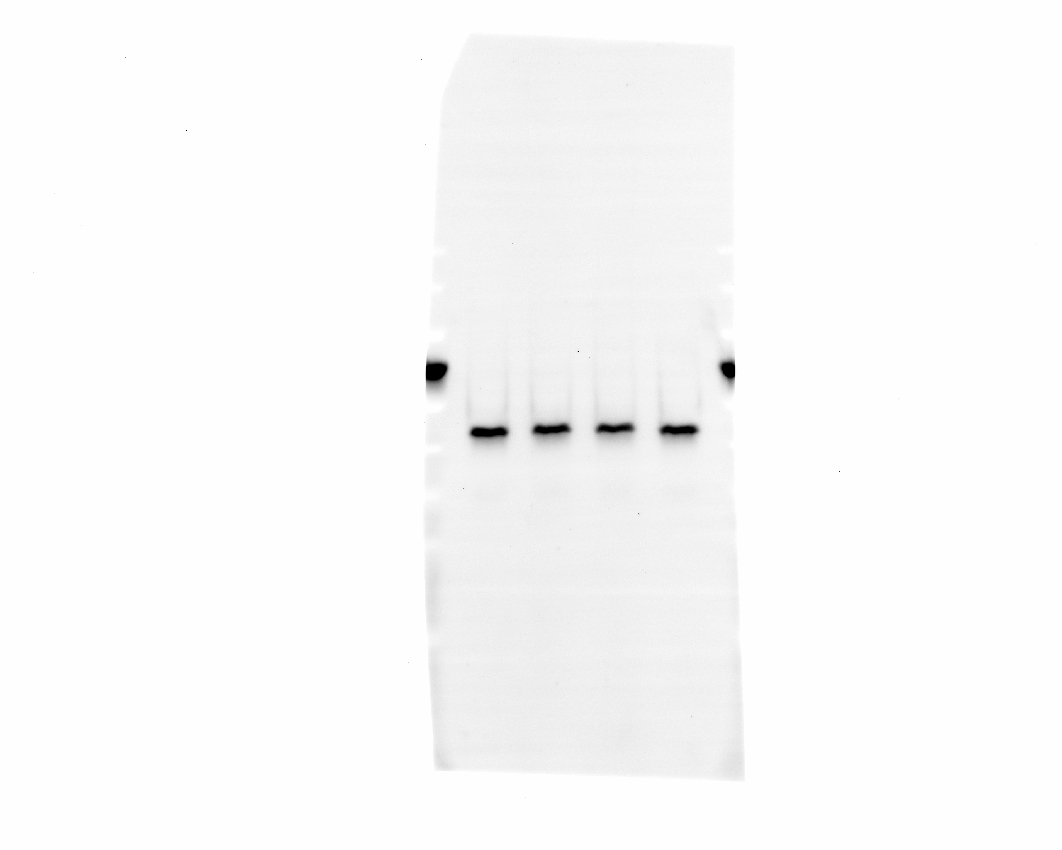

Supplement: Figure 3—source data 1. [file elife-85542-fig3-data1.zip › Figure 3 source data/Figure 3F/Figure 3F-actin for pInR blot raw data.jpg]

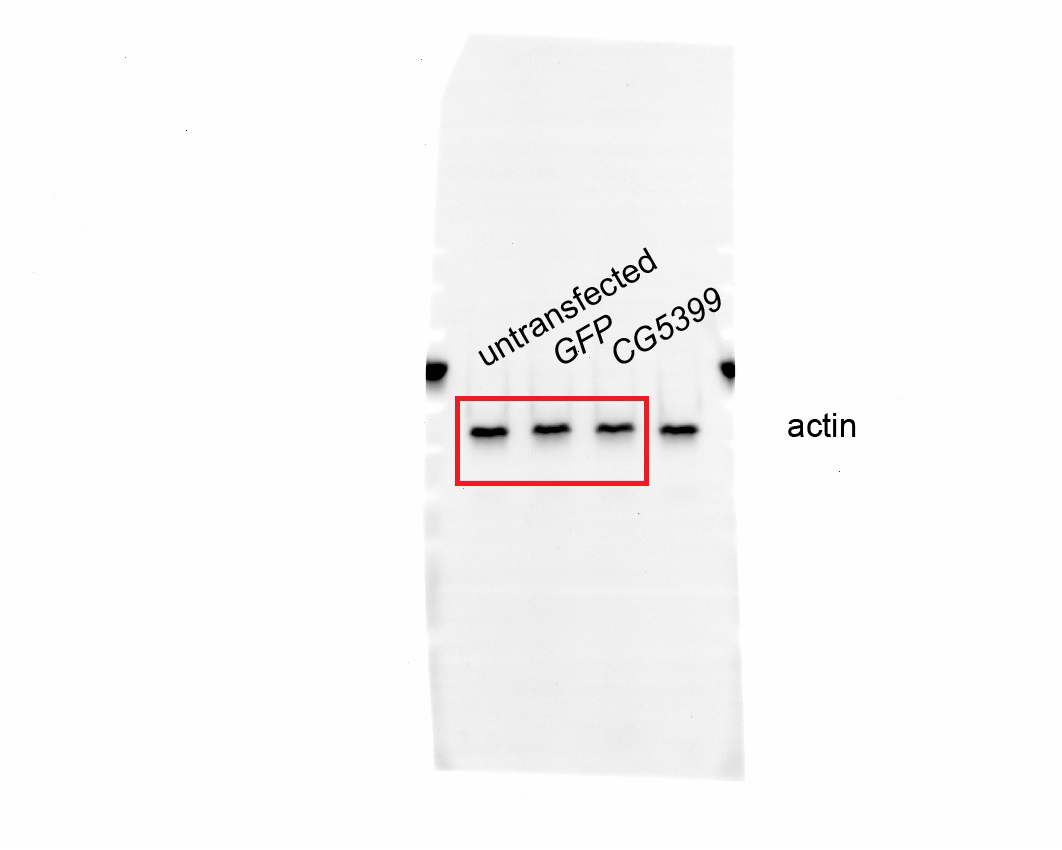

Supplement: Figure 3—source data 1. [file elife-85542-fig3-data1.zip › Figure 3 source data/Figure 3F/Figure 3F-actin for pInR blot.tif]

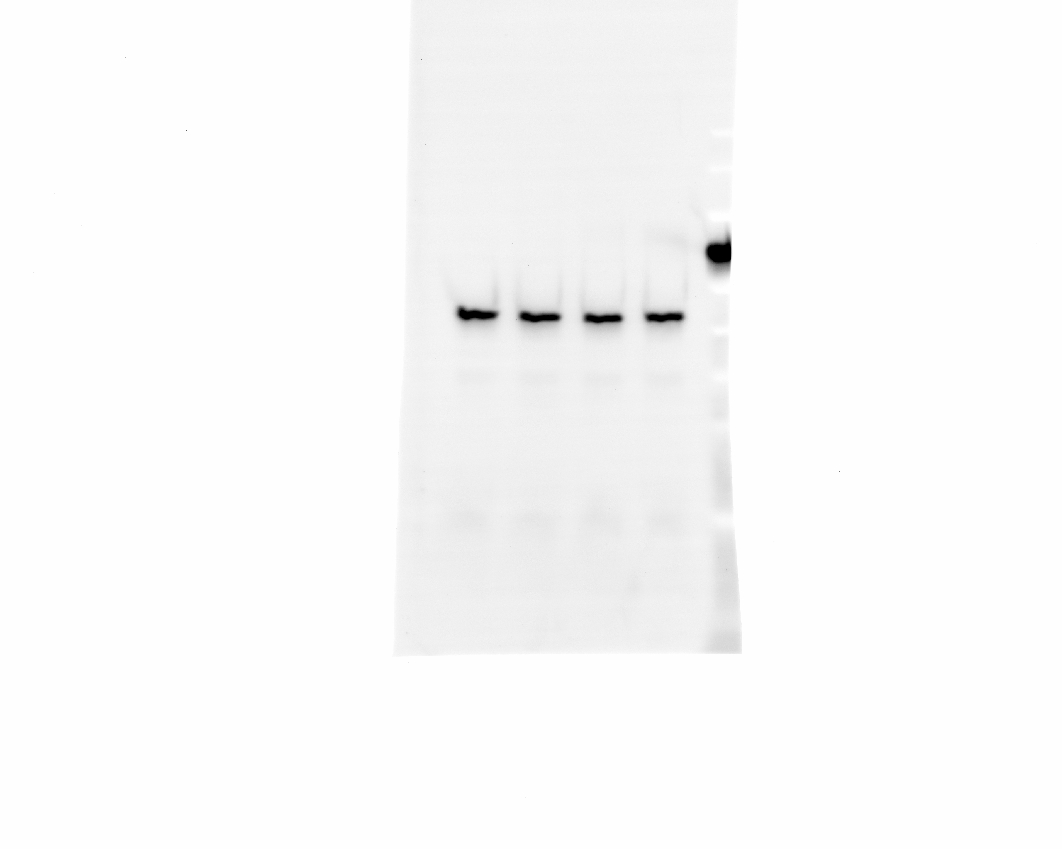

Supplement: Figure 3—source data 1. [file elife-85542-fig3-data1.zip › Figure 3 source data/Figure 3F/Figure 3F-actin for pS6 blot raw data.jpg]

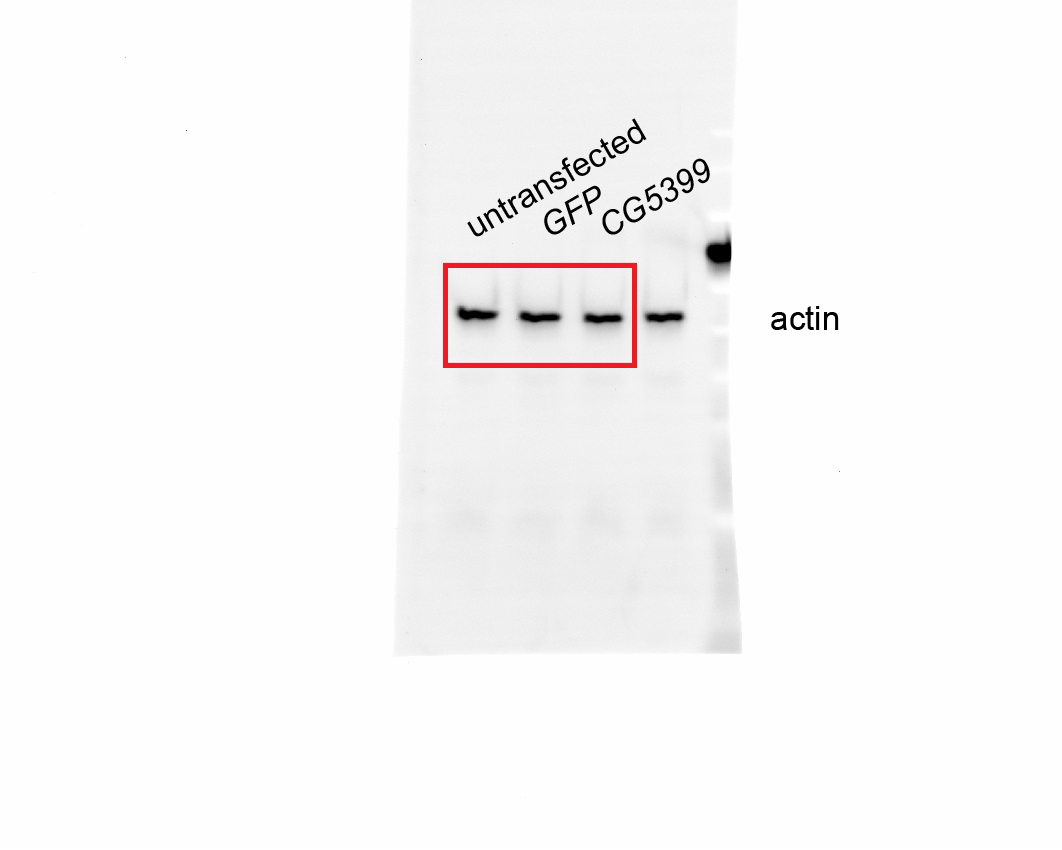

Supplement: Figure 3—source data 1. [file elife-85542-fig3-data1.zip › Figure 3 source data/Figure 3F/Figure 3F-actin for pS6 blot.tif]

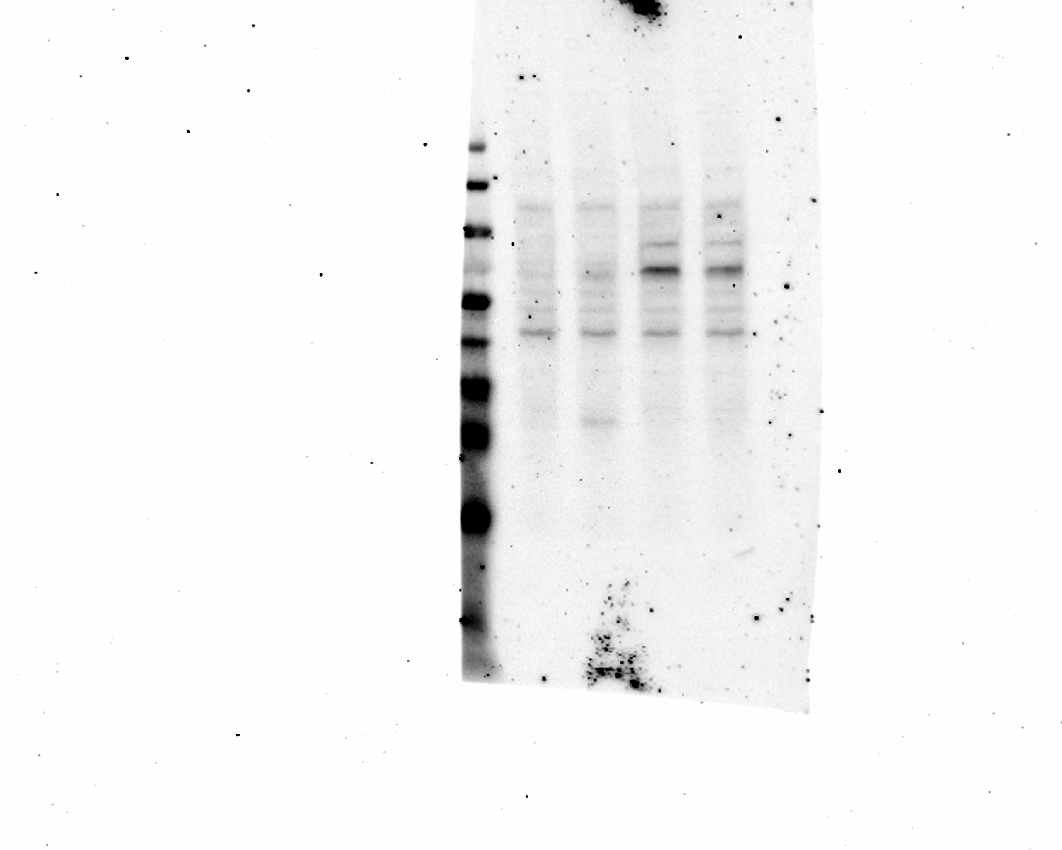

Supplement: Figure 3—source data 1. [file elife-85542-fig3-data1.zip › Figure 3 source data/Figure 3F/Figure 3F-pAkt raw data.jpg]

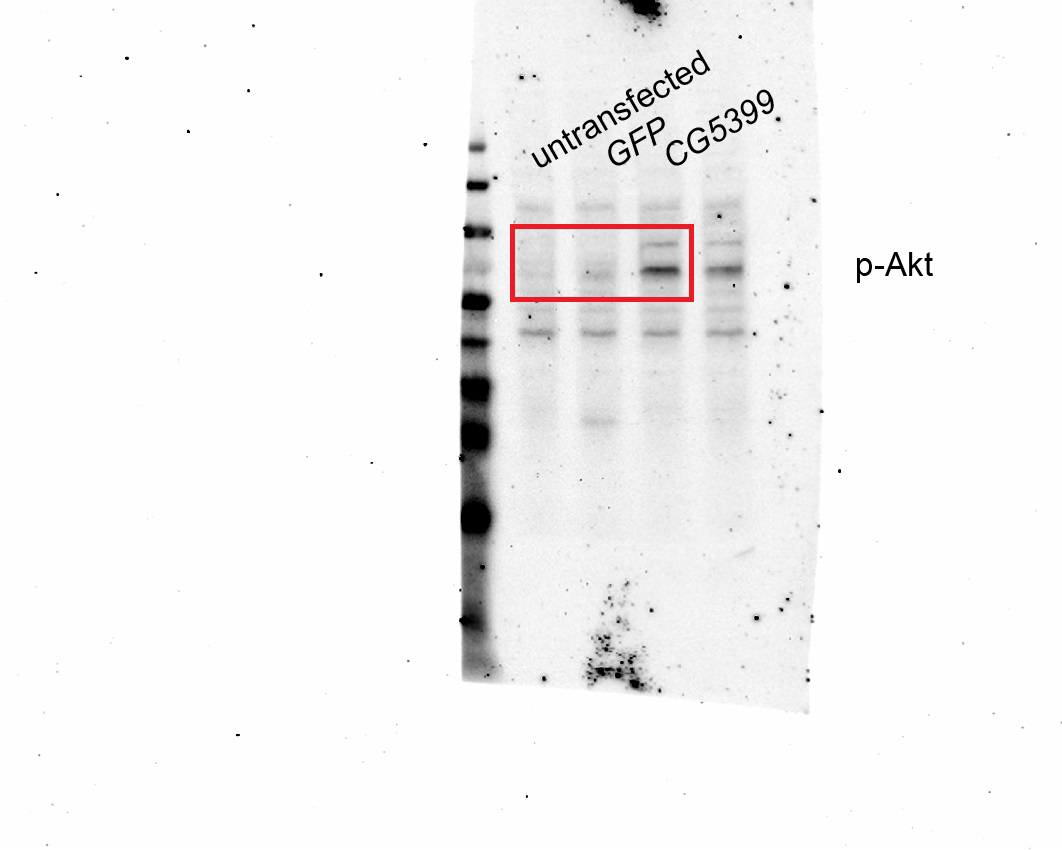

Supplement: Figure 3—source data 1. [file elife-85542-fig3-data1.zip › Figure 3 source data/Figure 3F/Figure 3F-pAkt.tif]

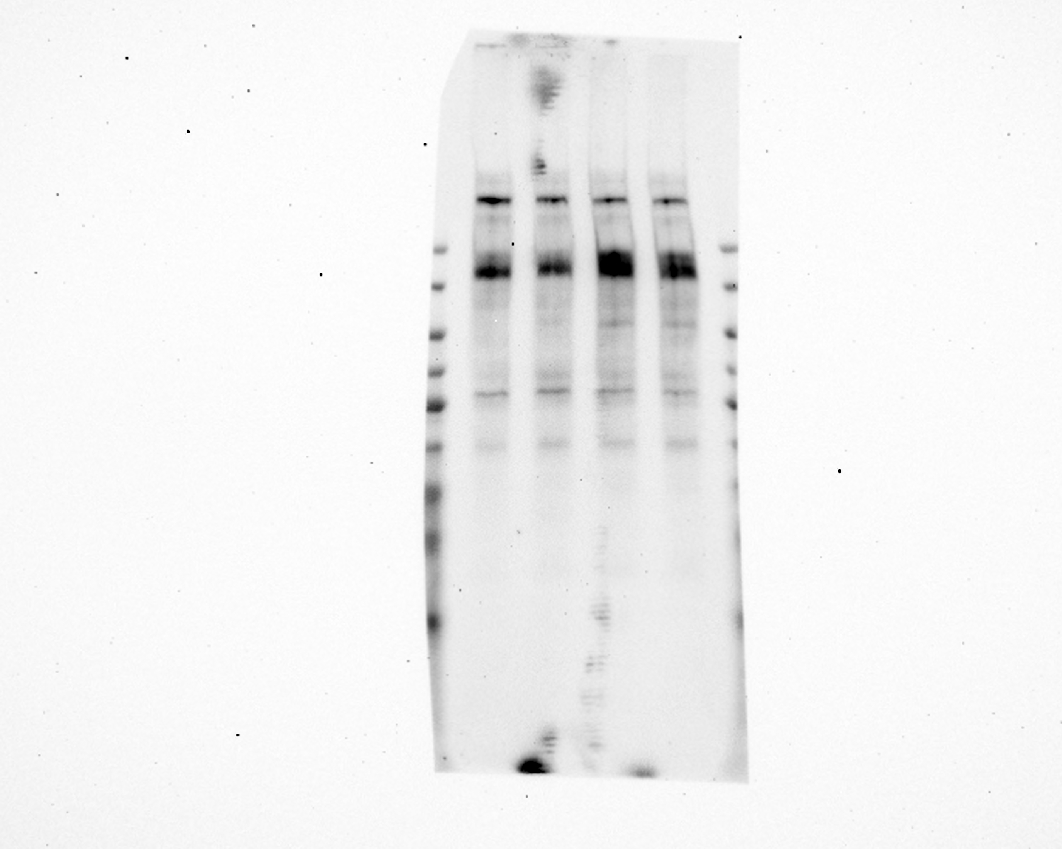

Supplement: Figure 3—source data 1. [file elife-85542-fig3-data1.zip › Figure 3 source data/Figure 3F/Figure 3F-pInR raw data.jpg]

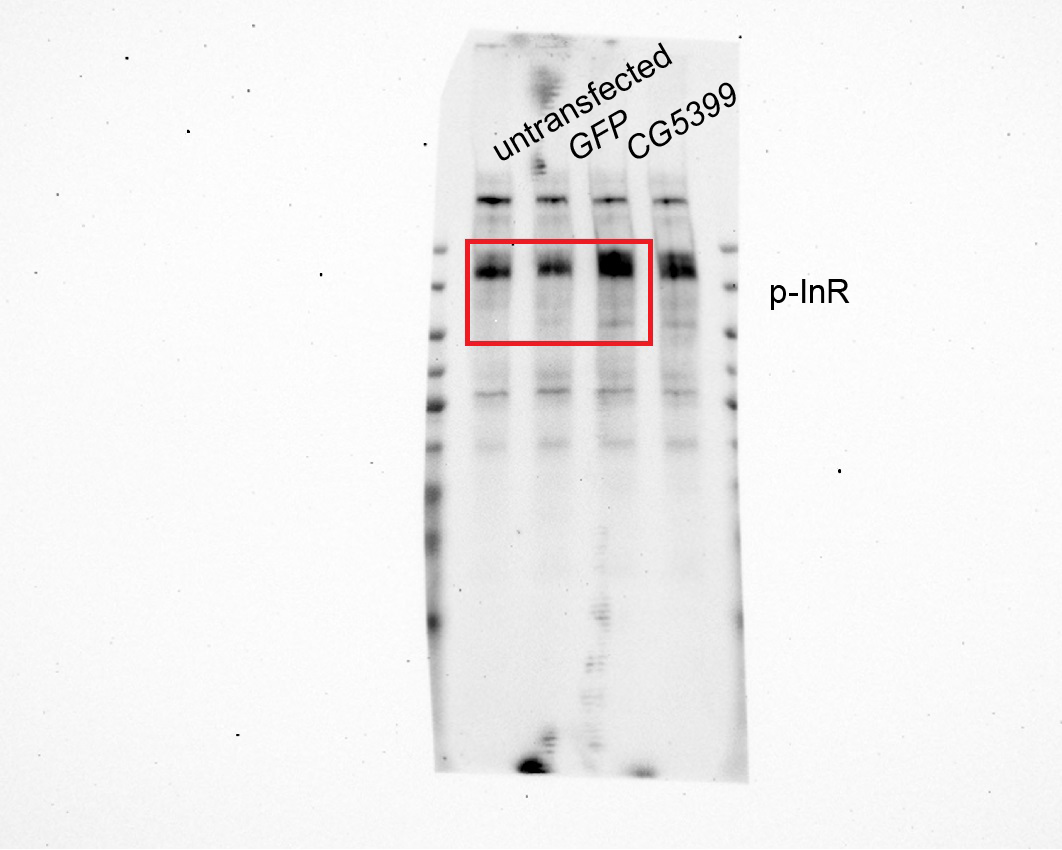

Supplement: Figure 3—source data 1. [file elife-85542-fig3-data1.zip › Figure 3 source data/Figure 3F/Figure 3F-pInR.tif]

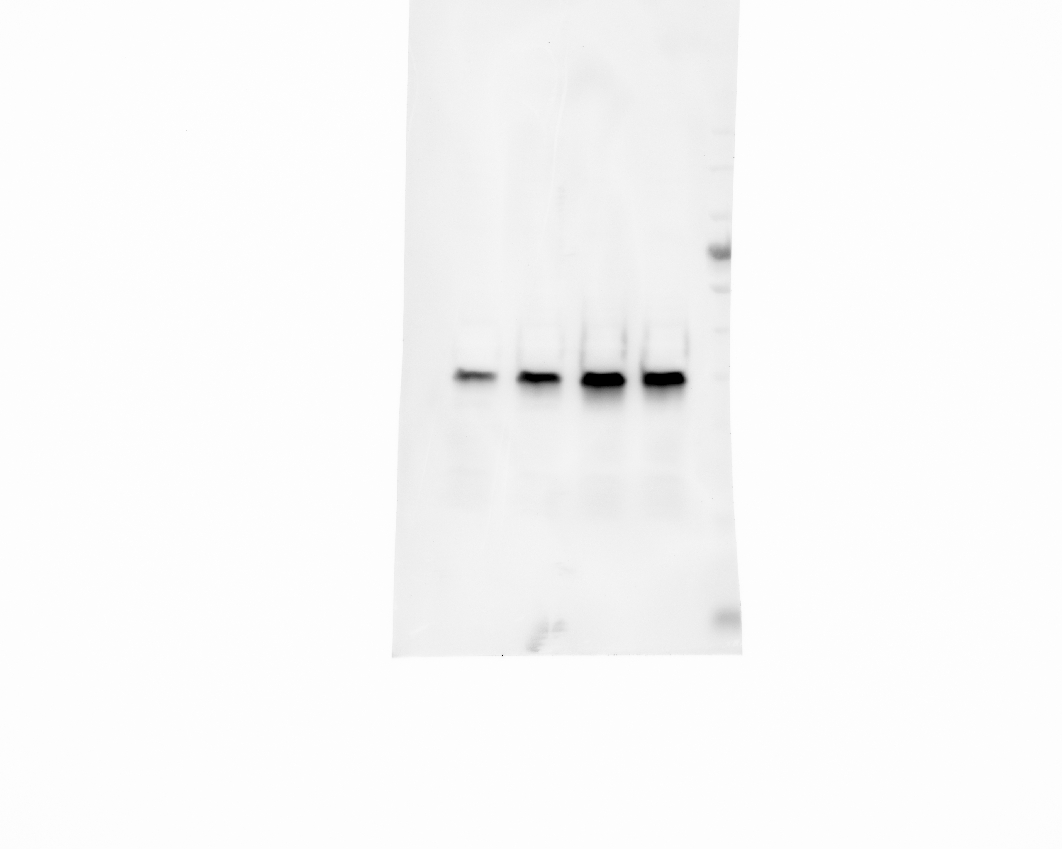

Supplement: Figure 3—source data 1. [file elife-85542-fig3-data1.zip › Figure 3 source data/Figure 3F/Figure 3F-pS6 raw data.jpg]

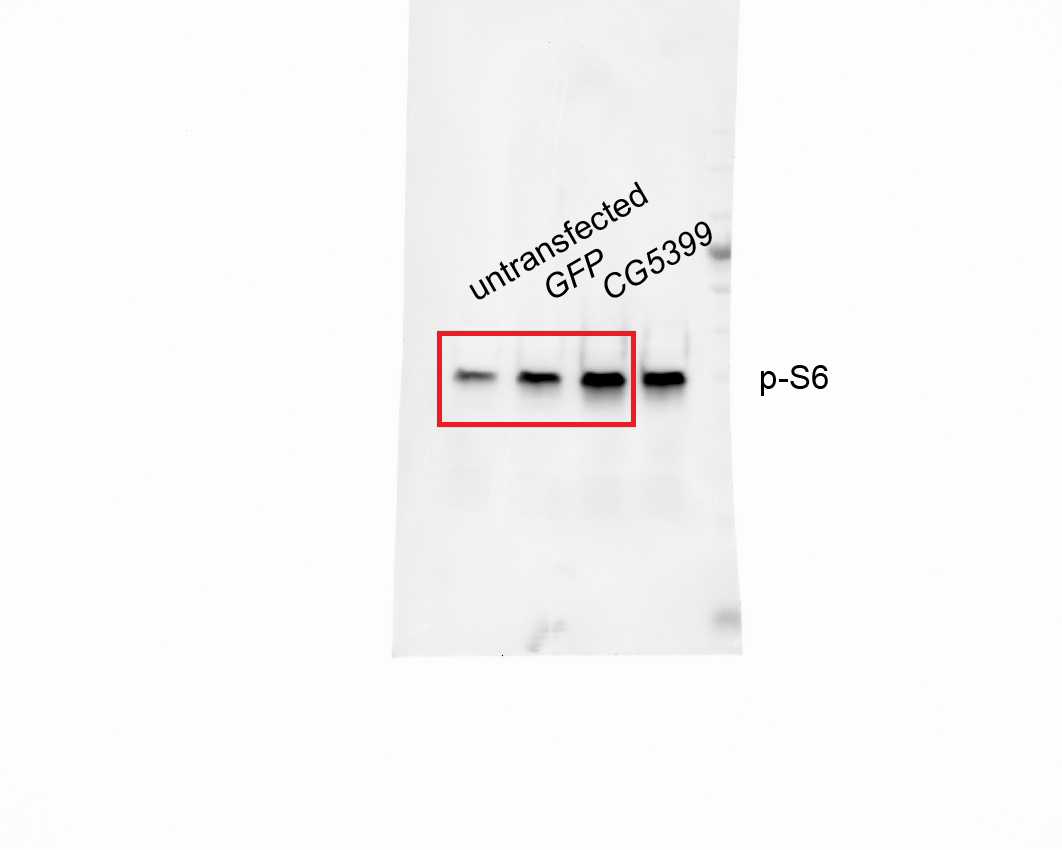

Supplement: Figure 3—source data 1. [file elife-85542-fig3-data1.zip › Figure 3 source data/Figure 3F/Figure 3F-pS6.tif]

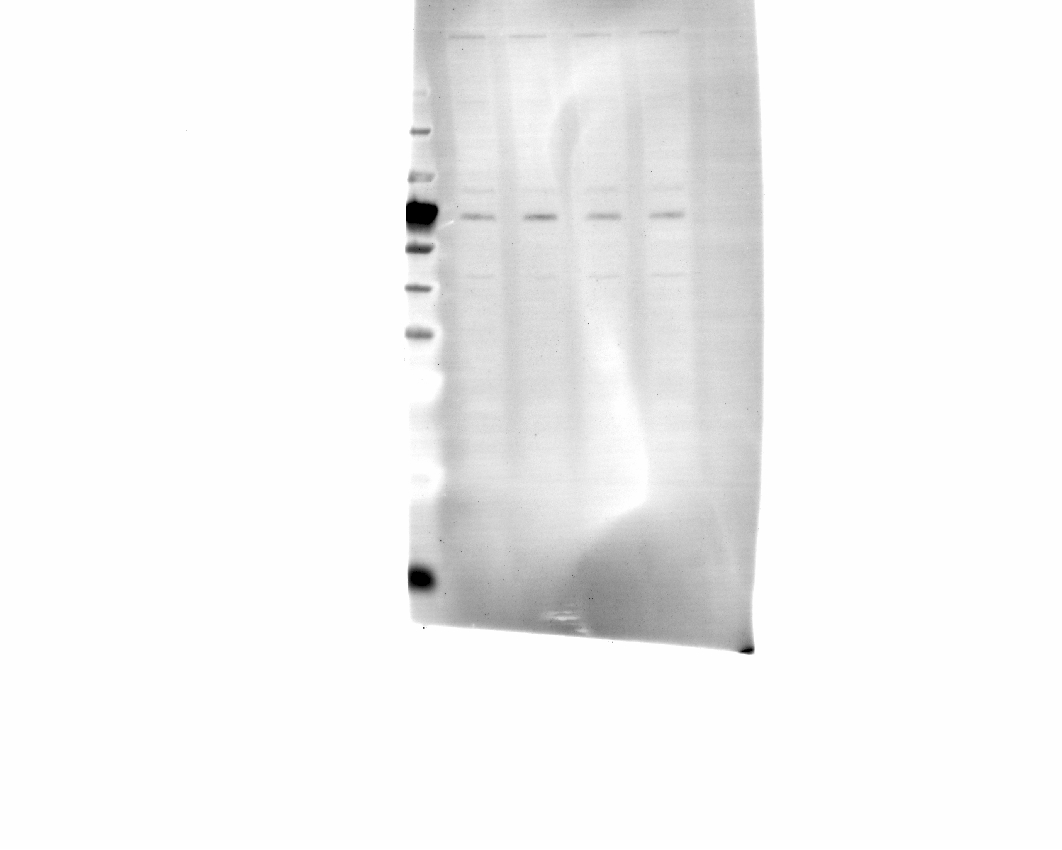

Supplement: Figure 3—source data 1. [file elife-85542-fig3-data1.zip › Figure 3 source data/Figure 3F/Figure 3F-total Akt raw data.jpg]

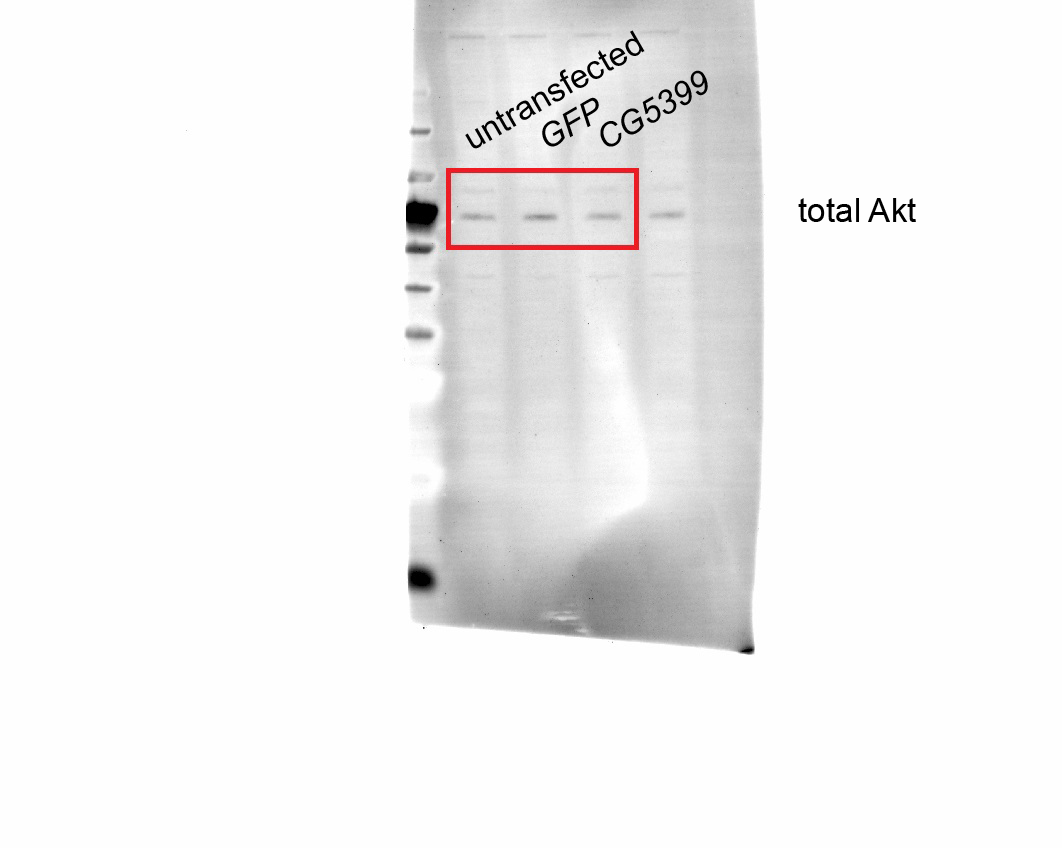

Supplement: Figure 3—source data 1. [file elife-85542-fig3-data1.zip › Figure 3 source data/Figure 3F/Figure 3F-total Akt.tif]

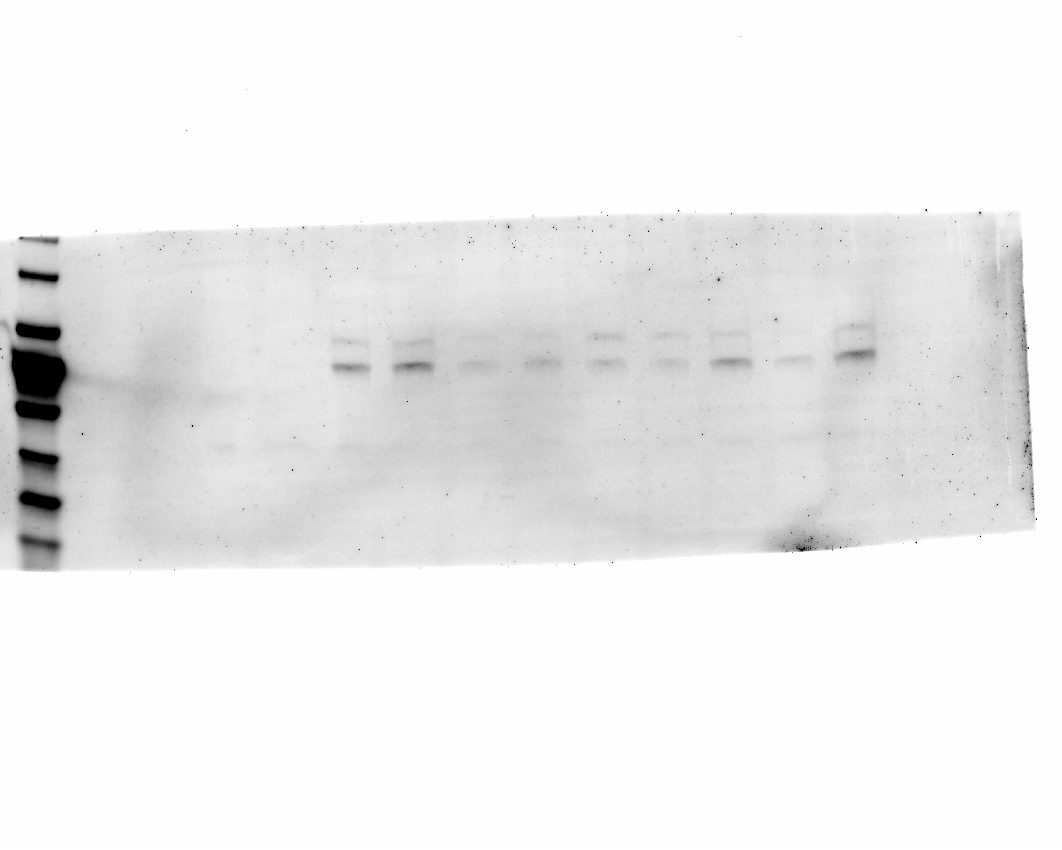

Supplement: Figure 3—figure supplement 1—source data 1. [file elife-85542-fig3-figsupp1-data1.zip › Figure 3-figure supplement 1 source data/Figure 3-figure supplement1A/S4A-pAkt raw data.jpg]

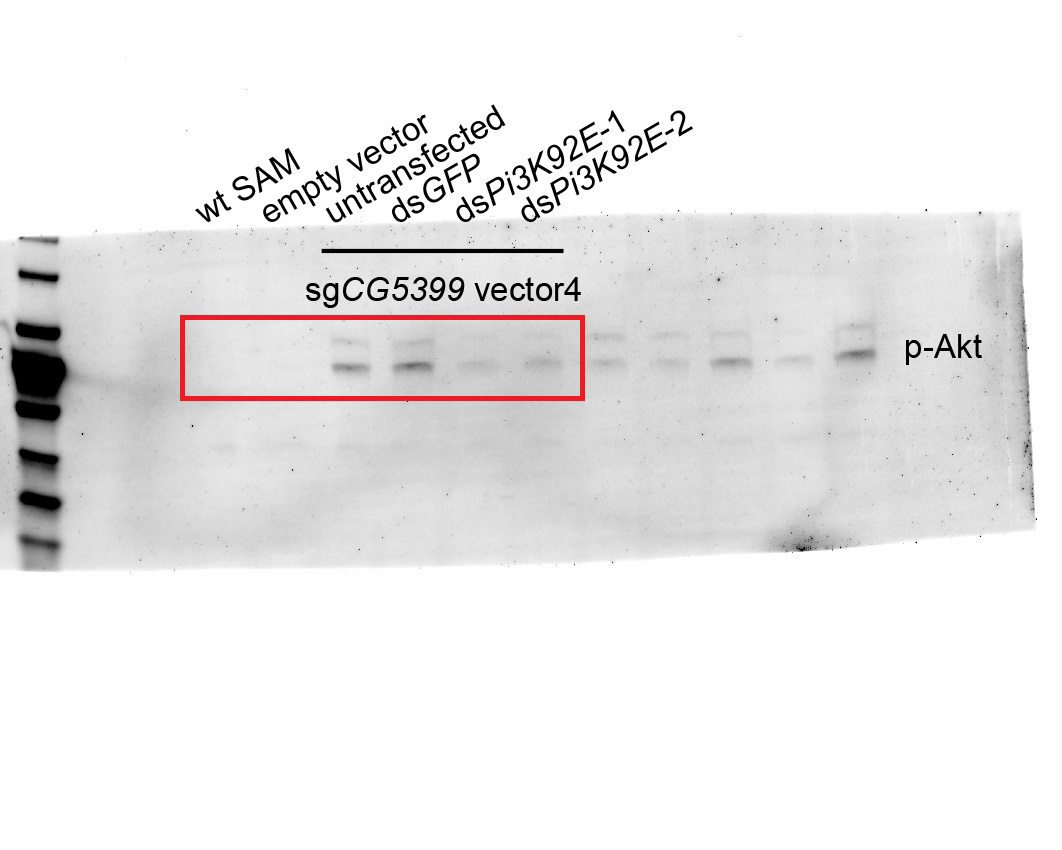

Supplement: Figure 3—figure supplement 1—source data 1. [file elife-85542-fig3-figsupp1-data1.zip › Figure 3-figure supplement 1 source data/Figure 3-figure supplement1A/S4A-pAkt.tif]

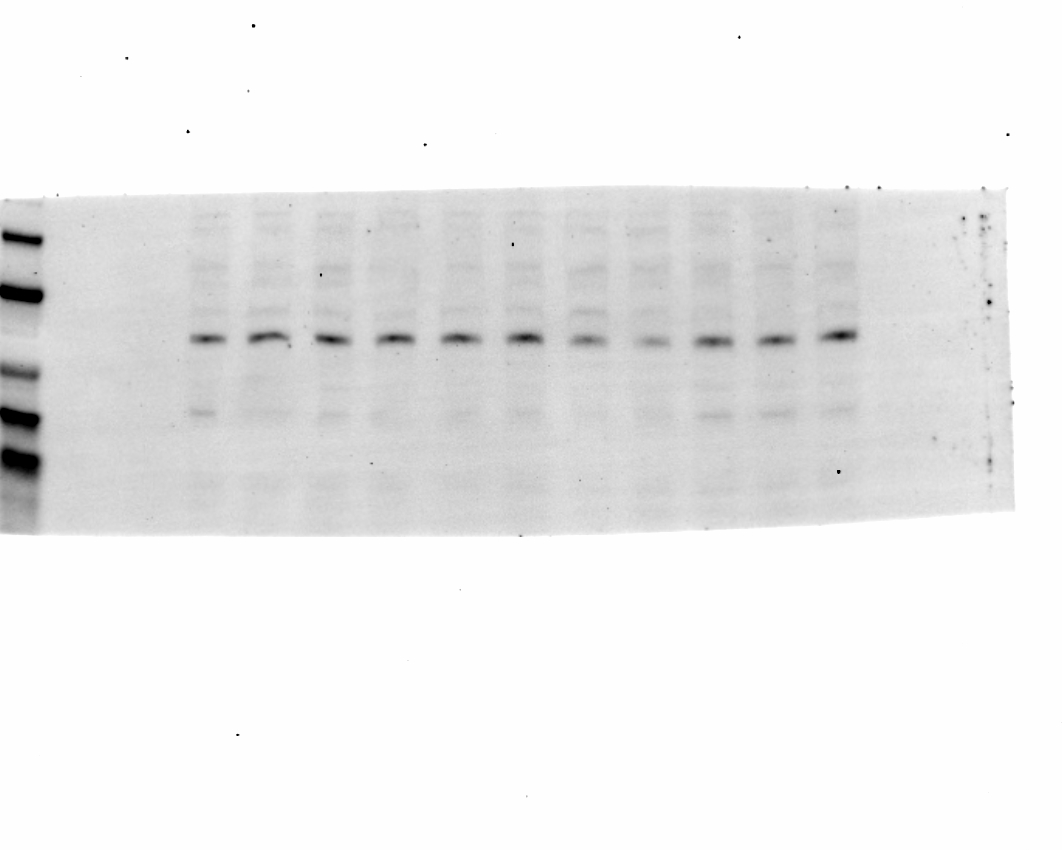

Supplement: Figure 3—figure supplement 1—source data 1. [file elife-85542-fig3-figsupp1-data1.zip › Figure 3-figure supplement 1 source data/Figure 3-figure supplement1A/S4A-total Akt raw data.jpg]

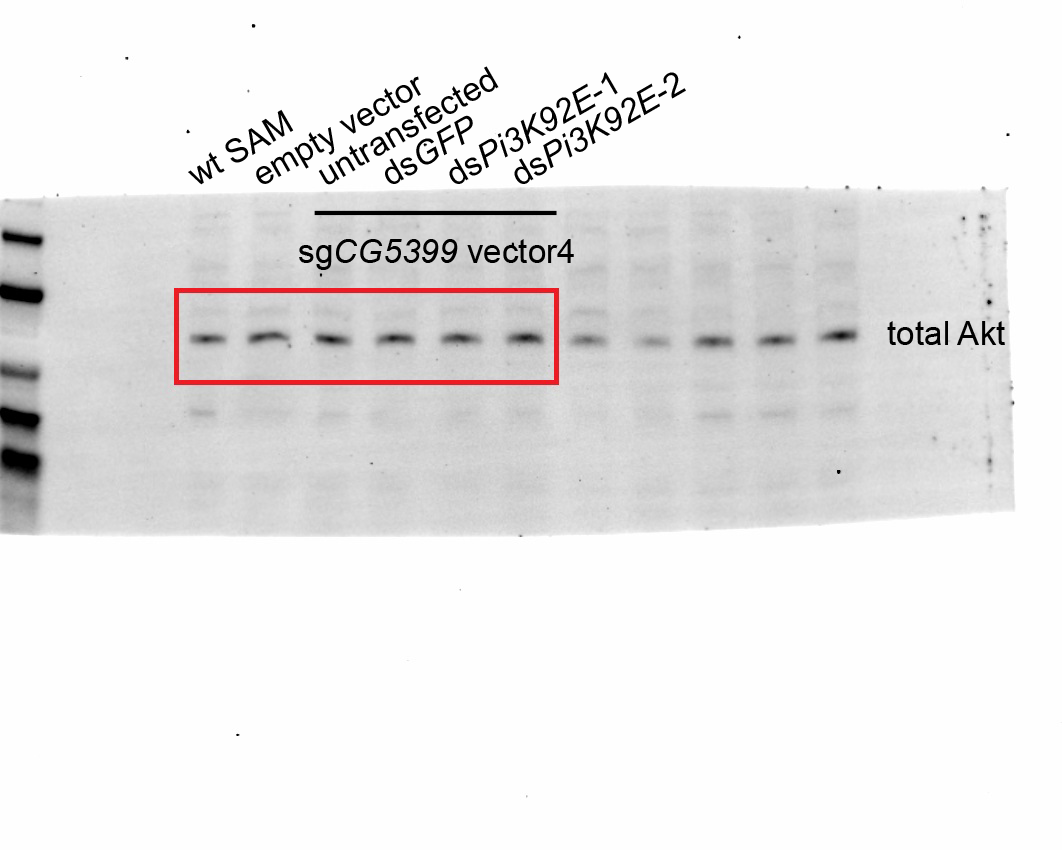

Supplement: Figure 3—figure supplement 1—source data 1. [file elife-85542-fig3-figsupp1-data1.zip › Figure 3-figure supplement 1 source data/Figure 3-figure supplement1A/S4A-total Akt.tif]

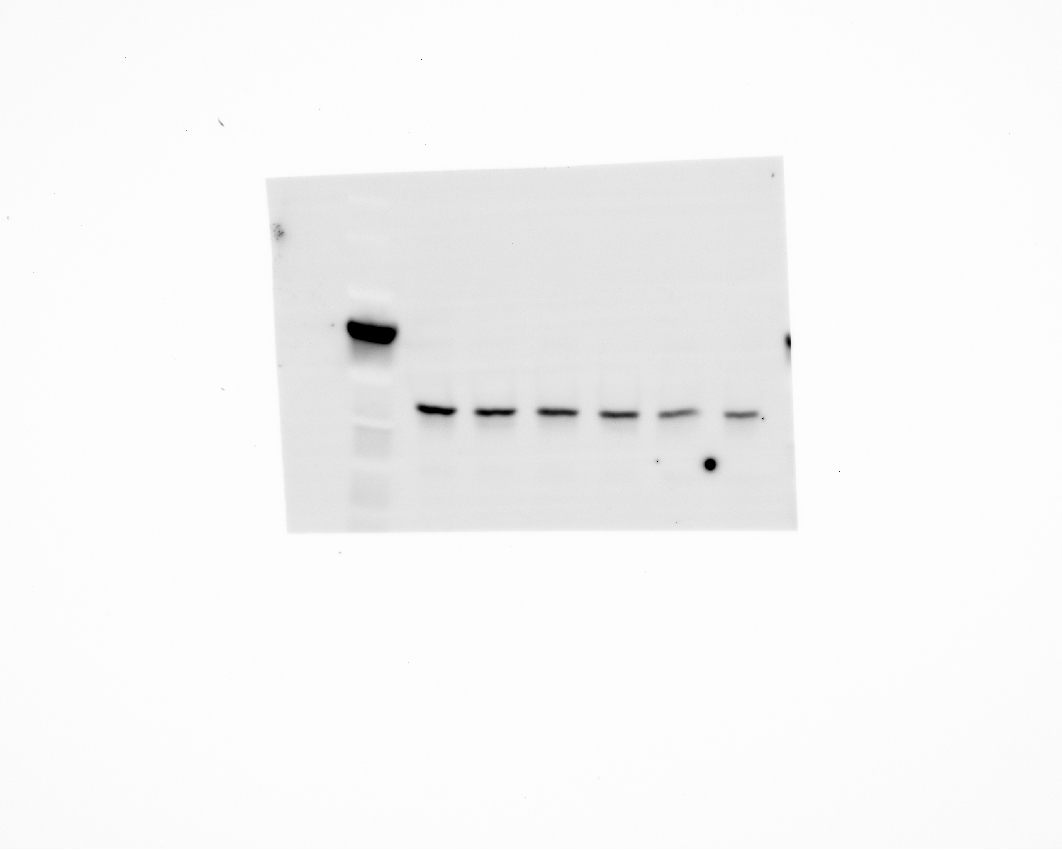

Supplement: Figure 3—figure supplement 1—source data 1. [file elife-85542-fig3-figsupp1-data1.zip › Figure 3-figure supplement 1 source data/Figure 3-figure supplement1B/S4B-actin for pAkt blot raw data.jpg]

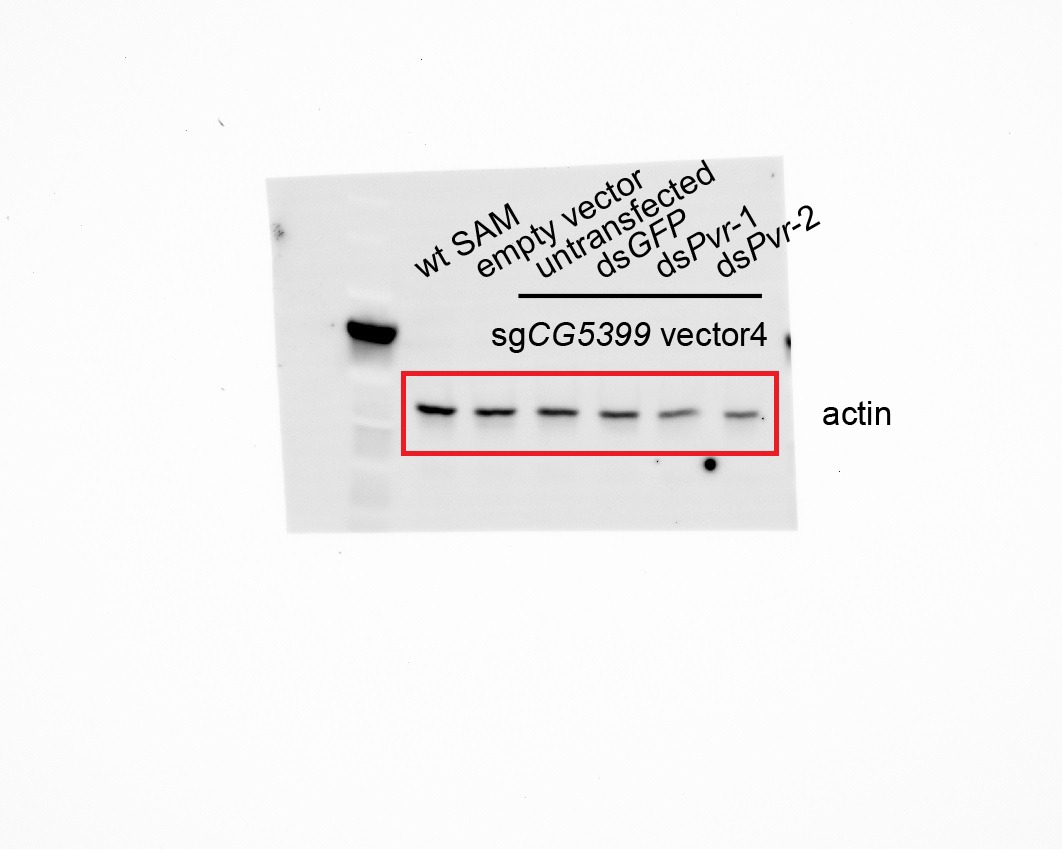

Supplement: Figure 3—figure supplement 1—source data 1. [file elife-85542-fig3-figsupp1-data1.zip › Figure 3-figure supplement 1 source data/Figure 3-figure supplement1B/S4B-actin for pAkt blot.tif]

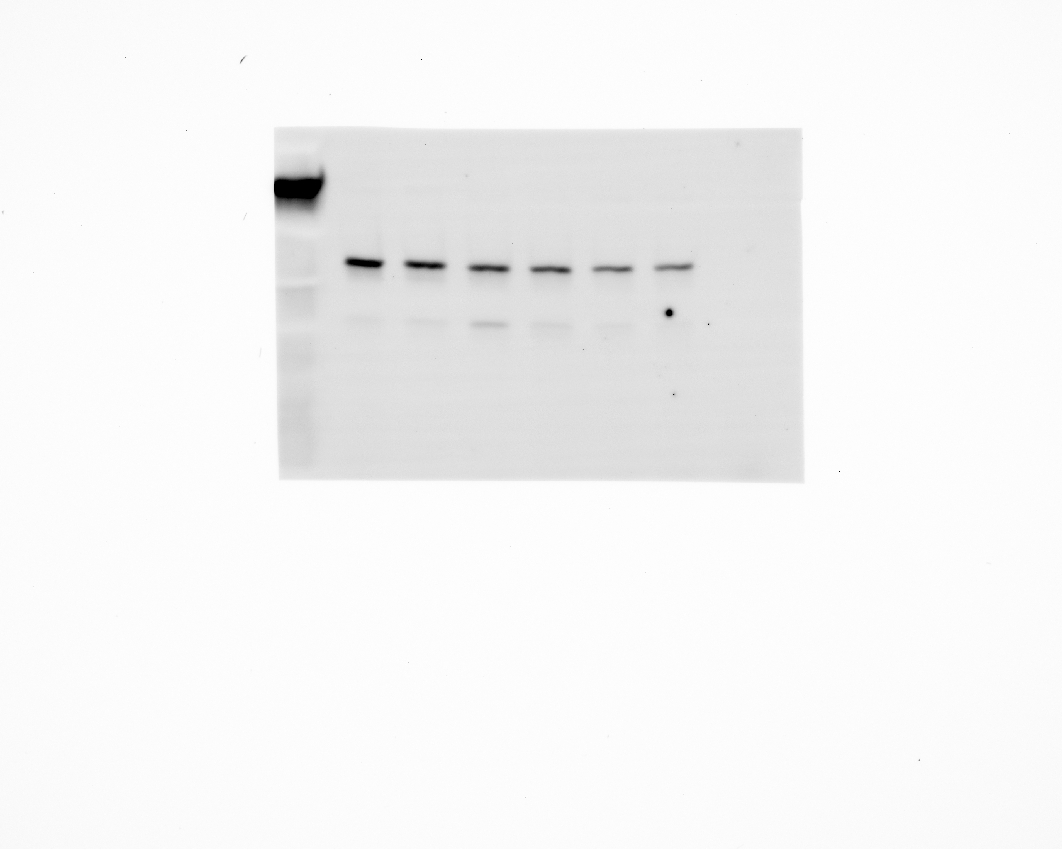

Supplement: Figure 3—figure supplement 1—source data 1. [file elife-85542-fig3-figsupp1-data1.zip › Figure 3-figure supplement 1 source data/Figure 3-figure supplement1B/S4B-actin for pS6 blot raw data.jpg]

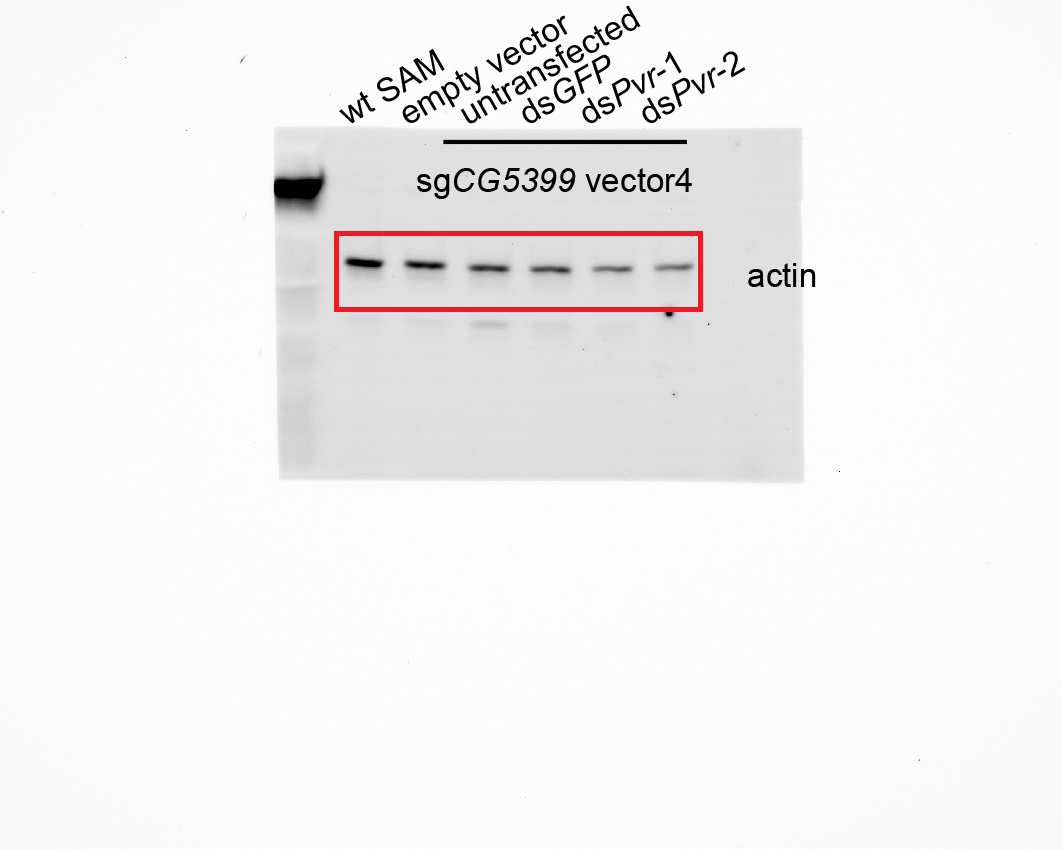

Supplement: Figure 3—figure supplement 1—source data 1. [file elife-85542-fig3-figsupp1-data1.zip › Figure 3-figure supplement 1 source data/Figure 3-figure supplement1B/S4B-actin for pS6 blot.tif]

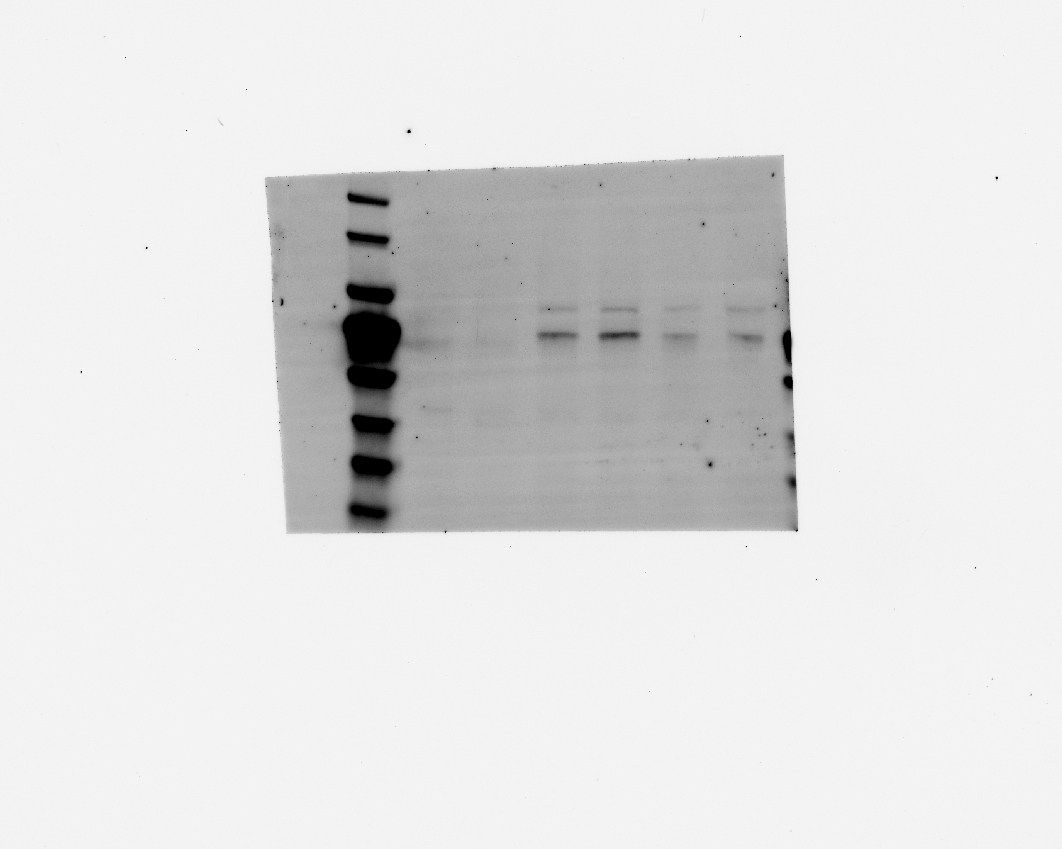

Supplement: Figure 3—figure supplement 1—source data 1. [file elife-85542-fig3-figsupp1-data1.zip › Figure 3-figure supplement 1 source data/Figure 3-figure supplement1B/S4B-pAkt raw data.jpg]

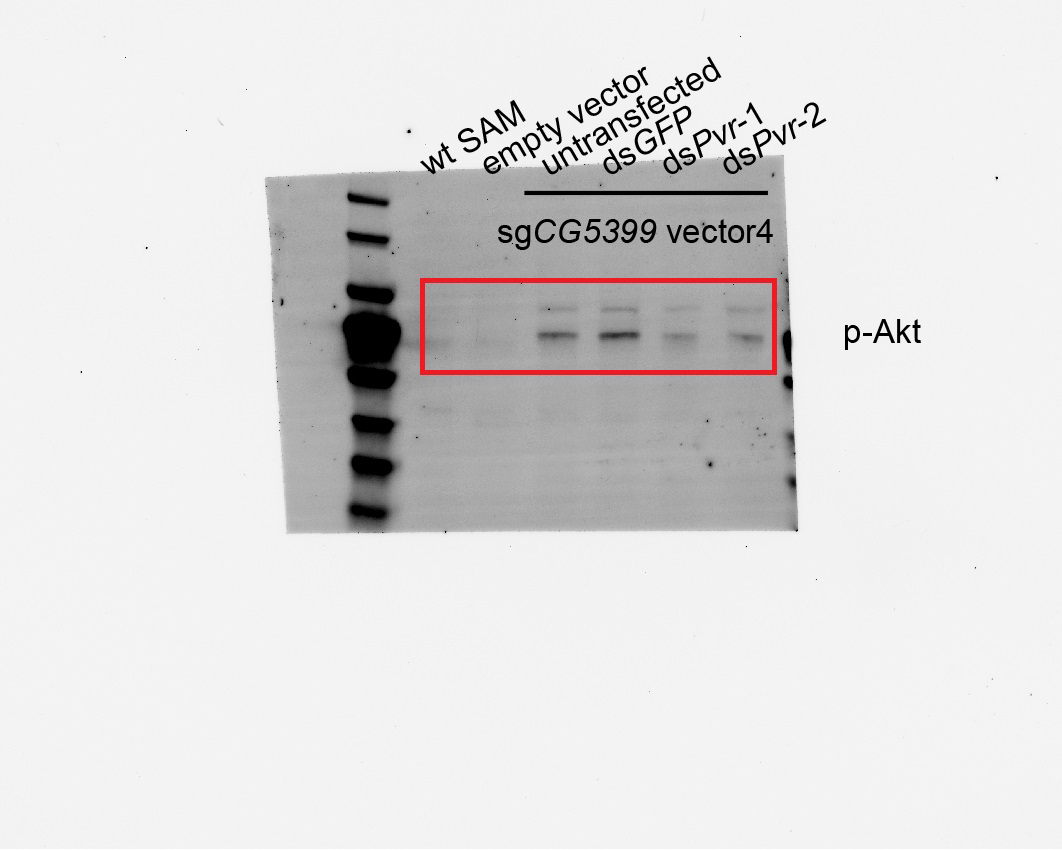

Supplement: Figure 3—figure supplement 1—source data 1. [file elife-85542-fig3-figsupp1-data1.zip › Figure 3-figure supplement 1 source data/Figure 3-figure supplement1B/S4B-pAkt.tif]

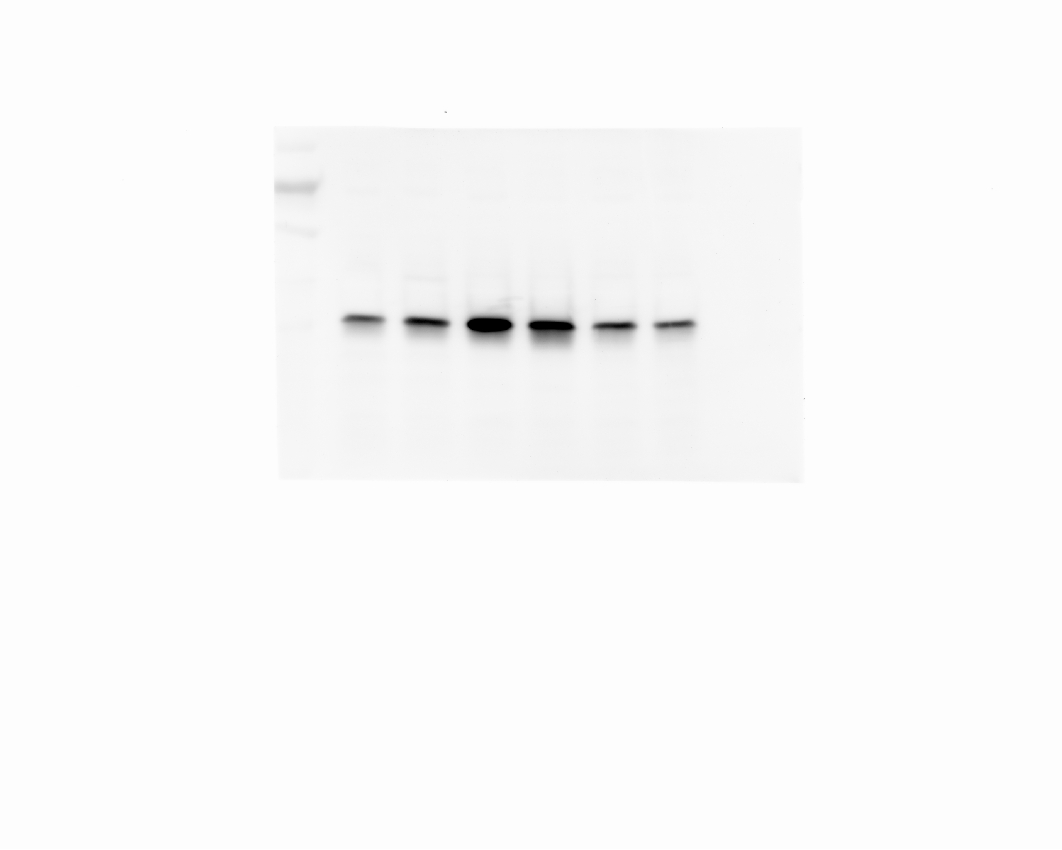

Supplement: Figure 3—figure supplement 1—source data 1. [file elife-85542-fig3-figsupp1-data1.zip › Figure 3-figure supplement 1 source data/Figure 3-figure supplement1B/S4B-pS6 raw data.jpg]

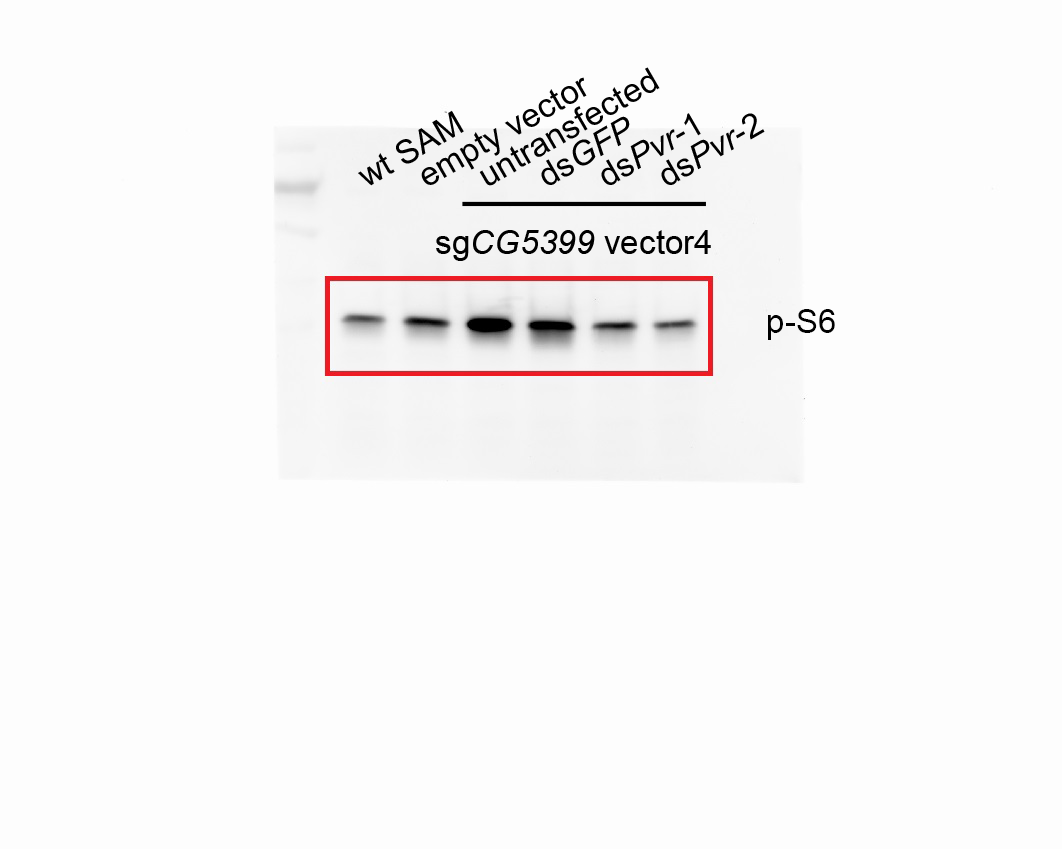

Supplement: Figure 3—figure supplement 1—source data 1. [file elife-85542-fig3-figsupp1-data1.zip › Figure 3-figure supplement 1 source data/Figure 3-figure supplement1B/S4B-pS6.tif]

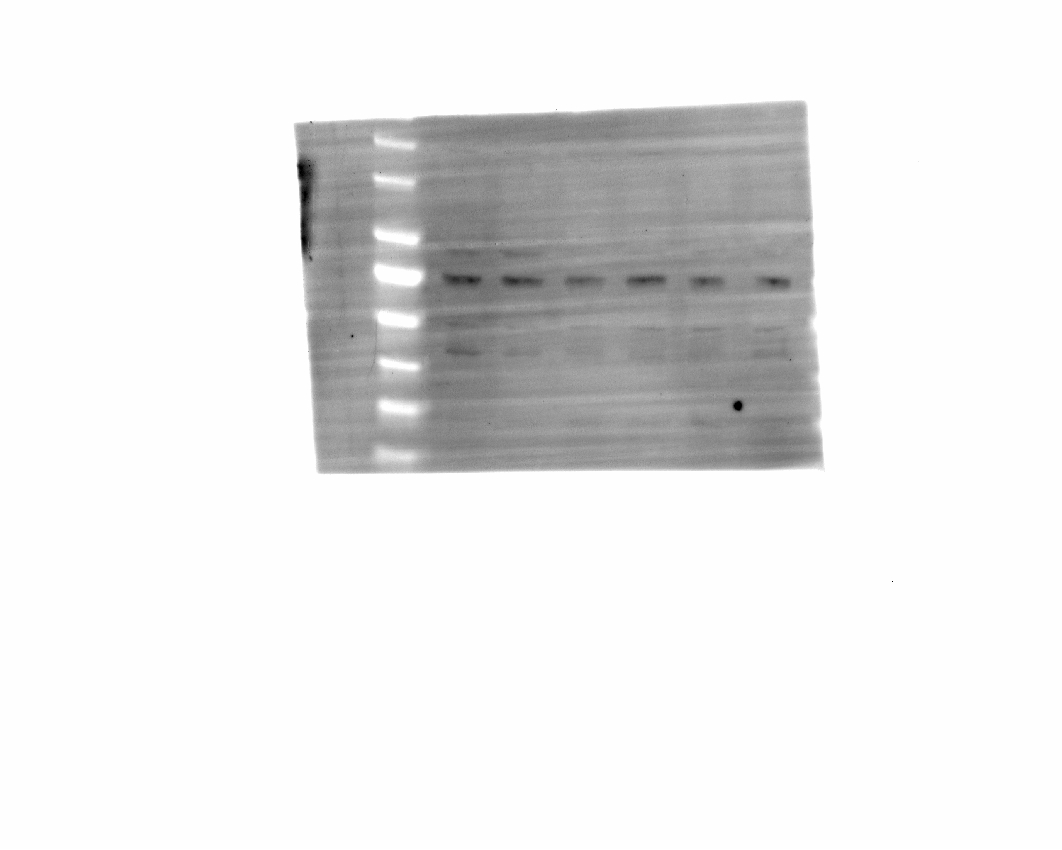

Supplement: Figure 3—figure supplement 1—source data 1. [file elife-85542-fig3-figsupp1-data1.zip › Figure 3-figure supplement 1 source data/Figure 3-figure supplement1B/S4B-total Akt raw data.jpg]

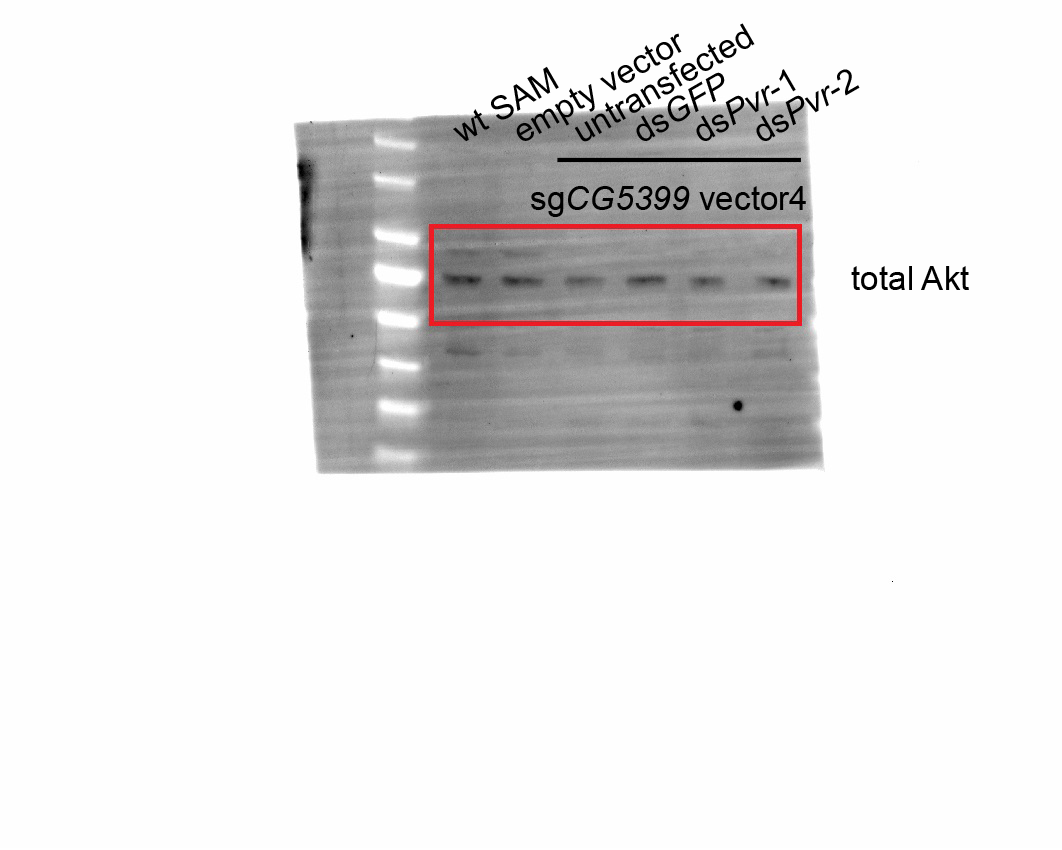

Supplement: Figure 3—figure supplement 1—source data 1. [file elife-85542-fig3-figsupp1-data1.zip › Figure 3-figure supplement 1 source data/Figure 3-figure supplement1B/S4B-total Akt.tif]

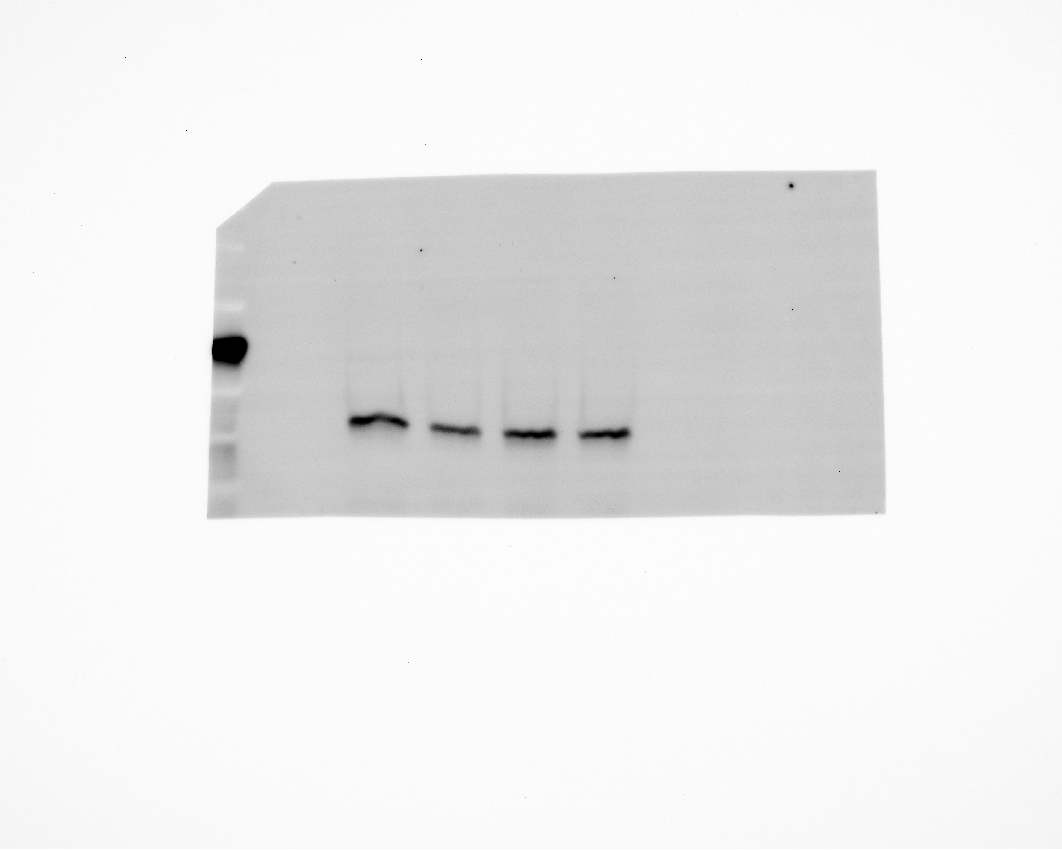

Supplement: Figure 3—figure supplement 1—source data 1. [file elife-85542-fig3-figsupp1-data1.zip › Figure 3-figure supplement 1 source data/Figure 3-figure supplement1C/S4C-actin for pAkt blot raw data.jpg]

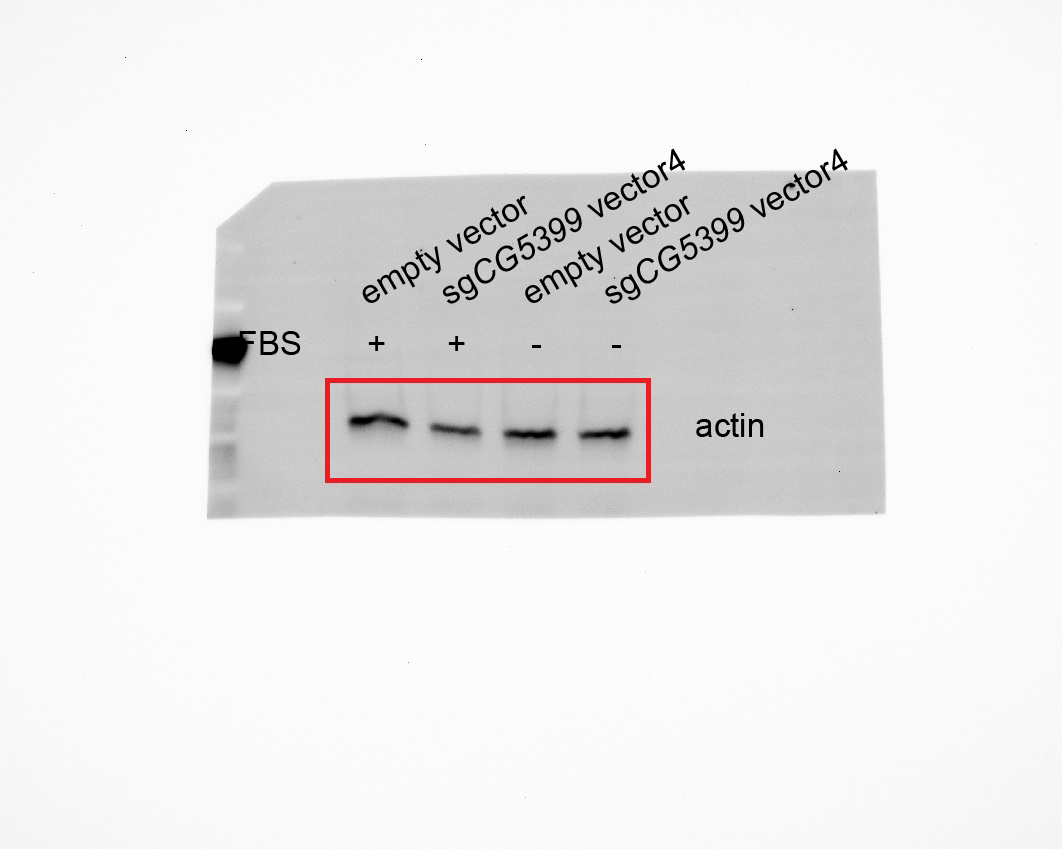

Supplement: Figure 3—figure supplement 1—source data 1. [file elife-85542-fig3-figsupp1-data1.zip › Figure 3-figure supplement 1 source data/Figure 3-figure supplement1C/S4C-actin for pAkt blot.tif]

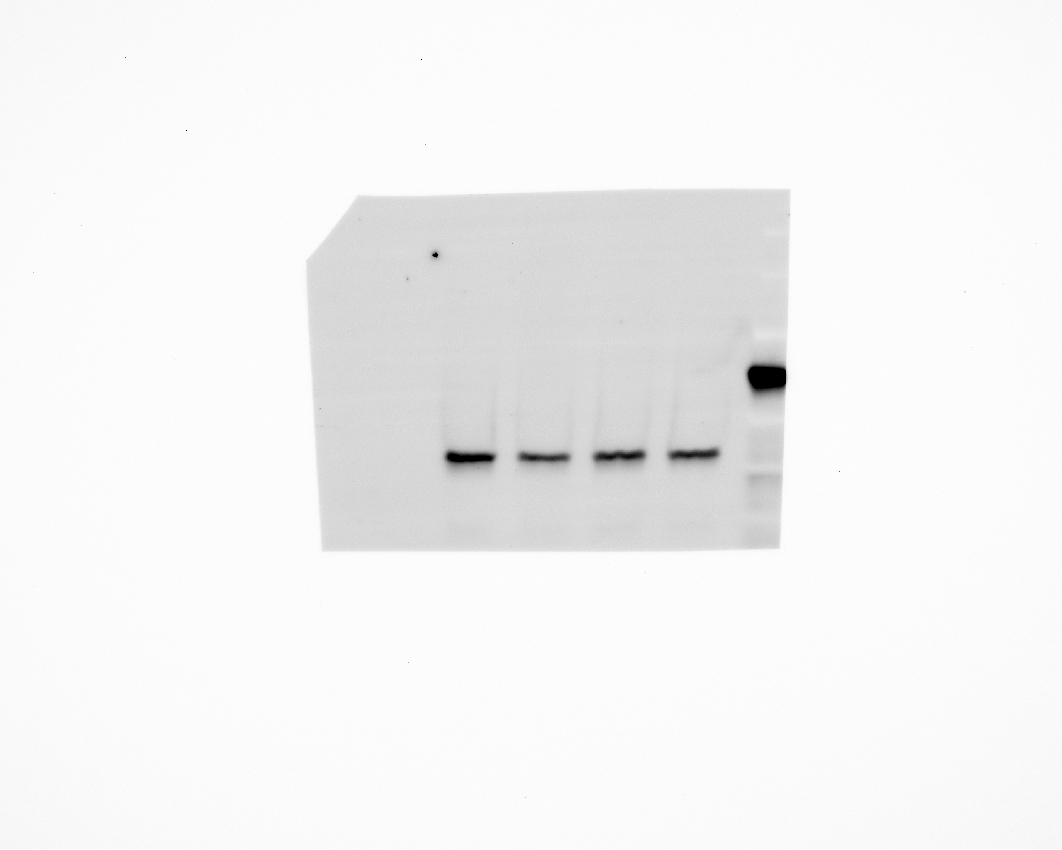

Supplement: Figure 3—figure supplement 1—source data 1. [file elife-85542-fig3-figsupp1-data1.zip › Figure 3-figure supplement 1 source data/Figure 3-figure supplement1C/S4C-actin for pInR blot raw data.jpg]

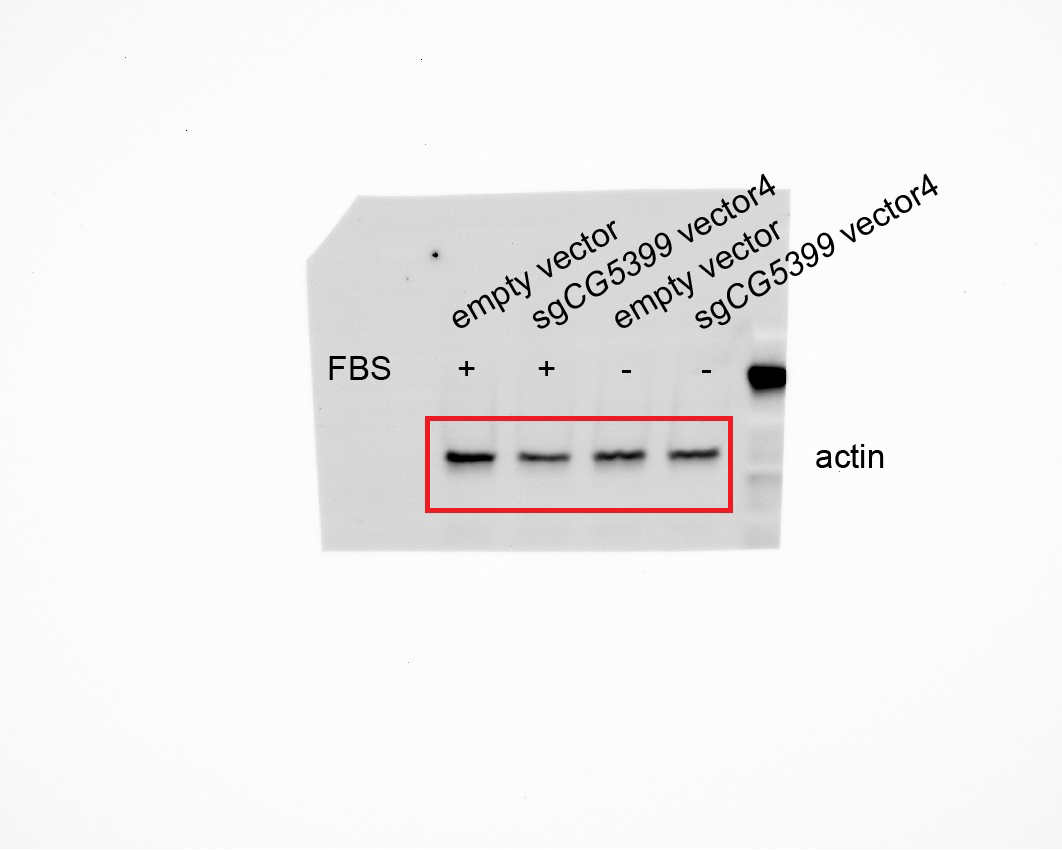

Supplement: Figure 3—figure supplement 1—source data 1. [file elife-85542-fig3-figsupp1-data1.zip › Figure 3-figure supplement 1 source data/Figure 3-figure supplement1C/S4C-actin for pInR blot.tif]

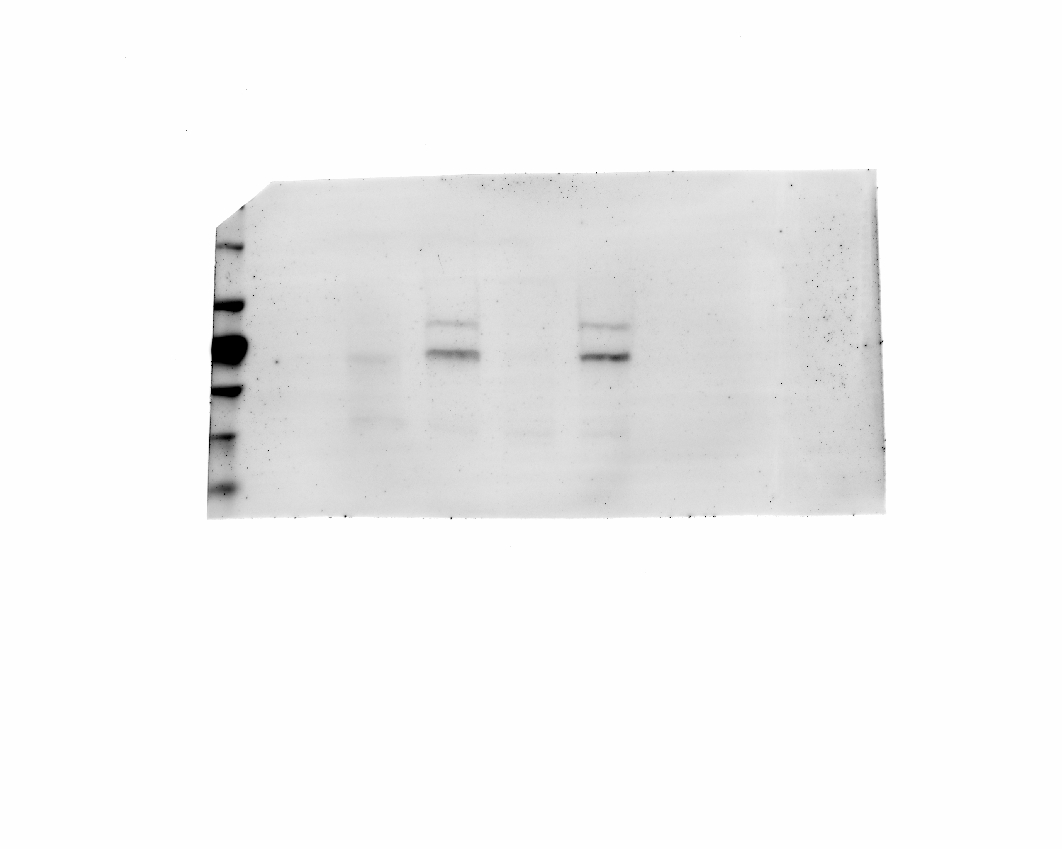

Supplement: Figure 3—figure supplement 1—source data 1. [file elife-85542-fig3-figsupp1-data1.zip › Figure 3-figure supplement 1 source data/Figure 3-figure supplement1C/S4C-pAkt raw data.jpg]

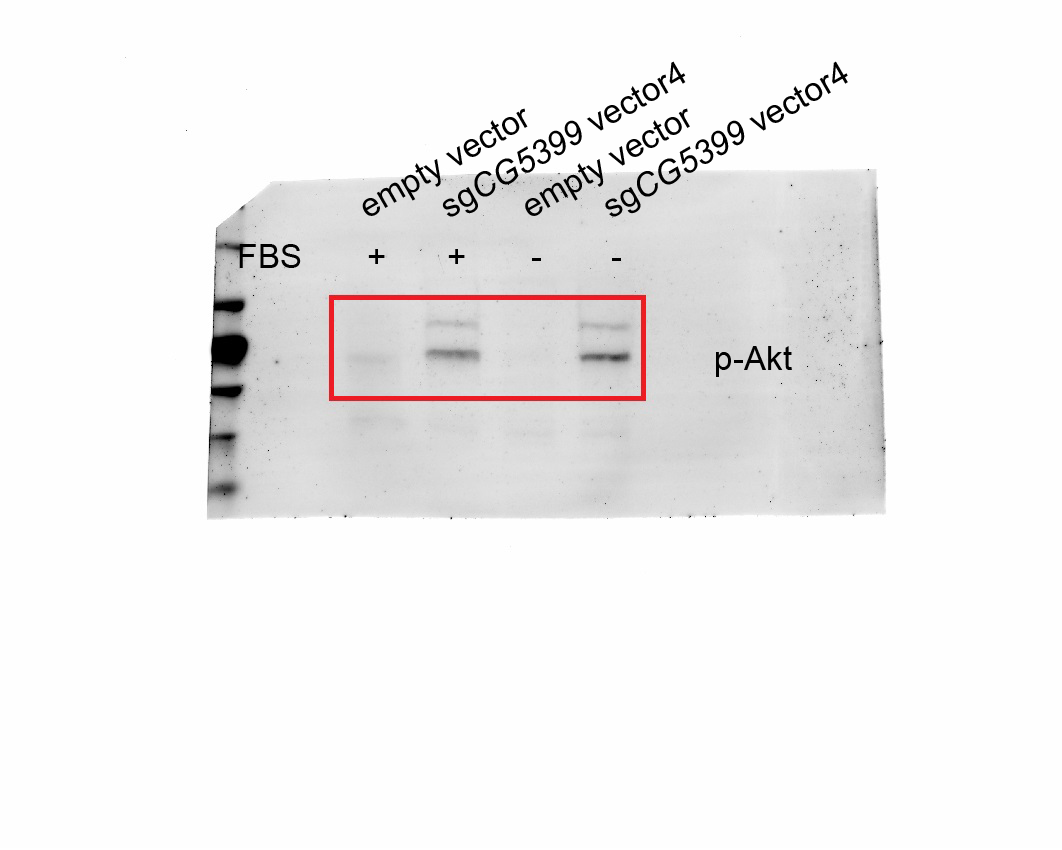

Supplement: Figure 3—figure supplement 1—source data 1. [file elife-85542-fig3-figsupp1-data1.zip › Figure 3-figure supplement 1 source data/Figure 3-figure supplement1C/S4C-pAkt.tif]

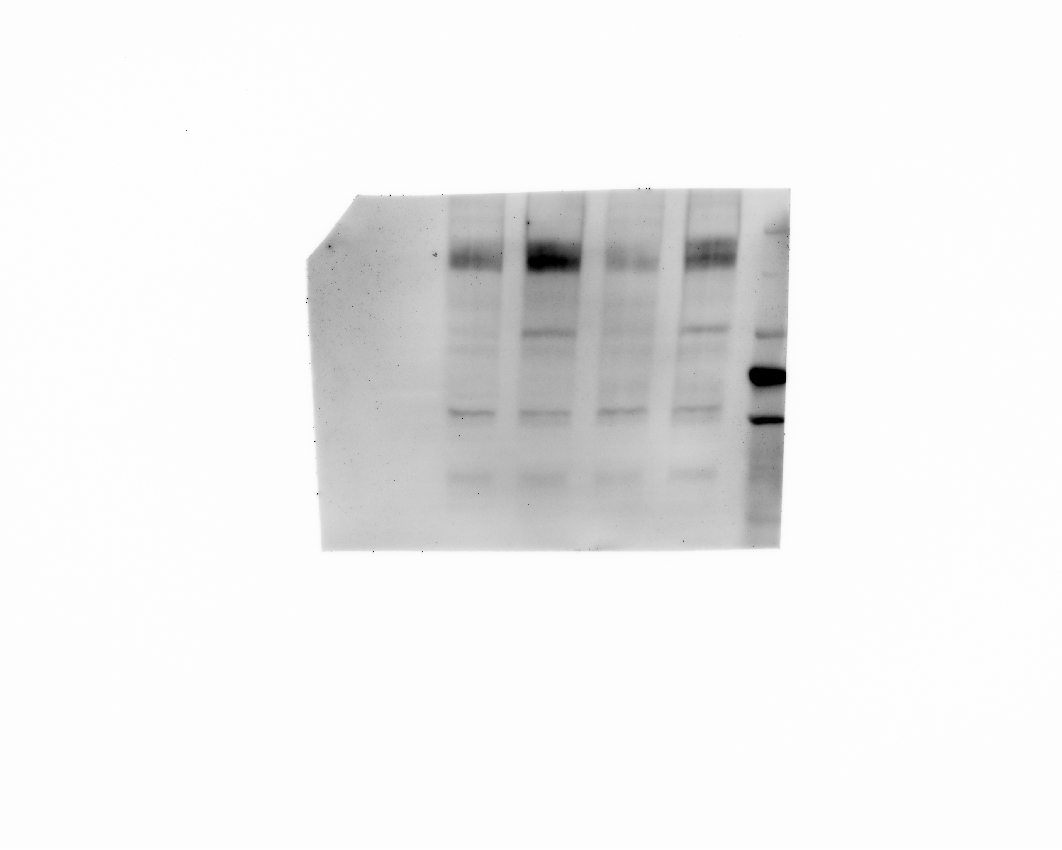

Supplement: Figure 3—figure supplement 1—source data 1. [file elife-85542-fig3-figsupp1-data1.zip › Figure 3-figure supplement 1 source data/Figure 3-figure supplement1C/S4C-pInR raw data.jpg]

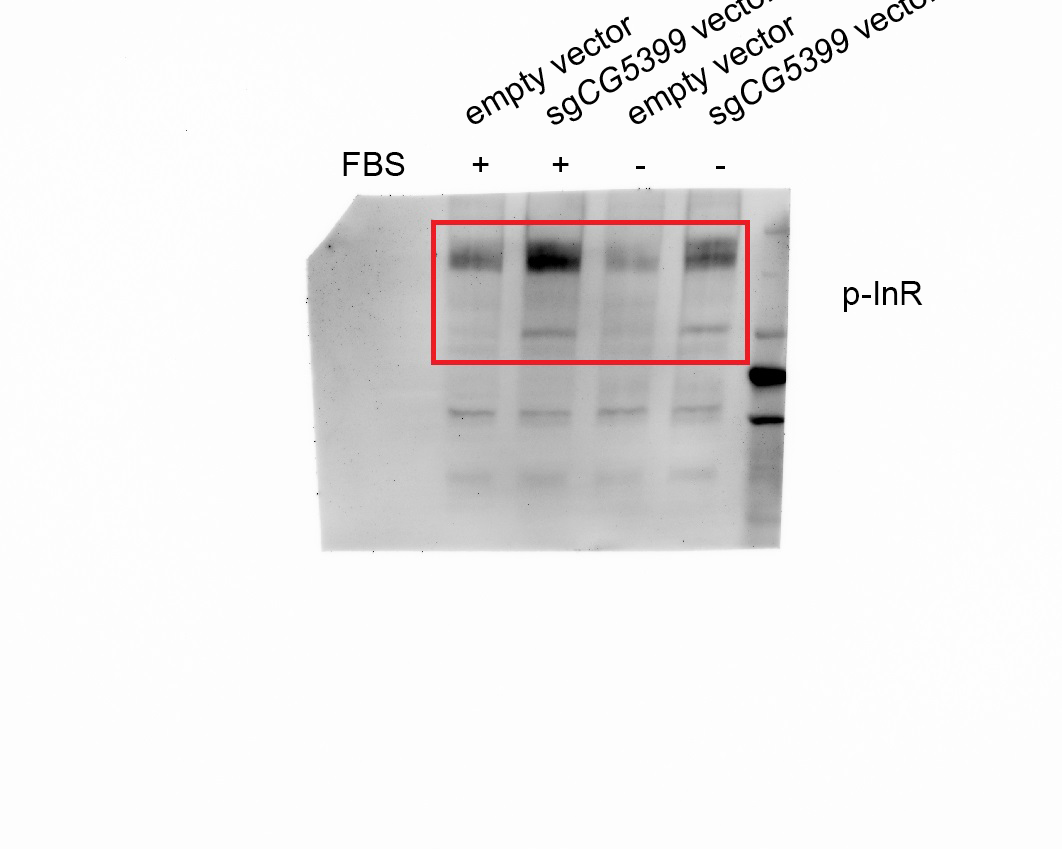

Supplement: Figure 3—figure supplement 1—source data 1. [file elife-85542-fig3-figsupp1-data1.zip › Figure 3-figure supplement 1 source data/Figure 3-figure supplement1C/S4C-pInR.tif]

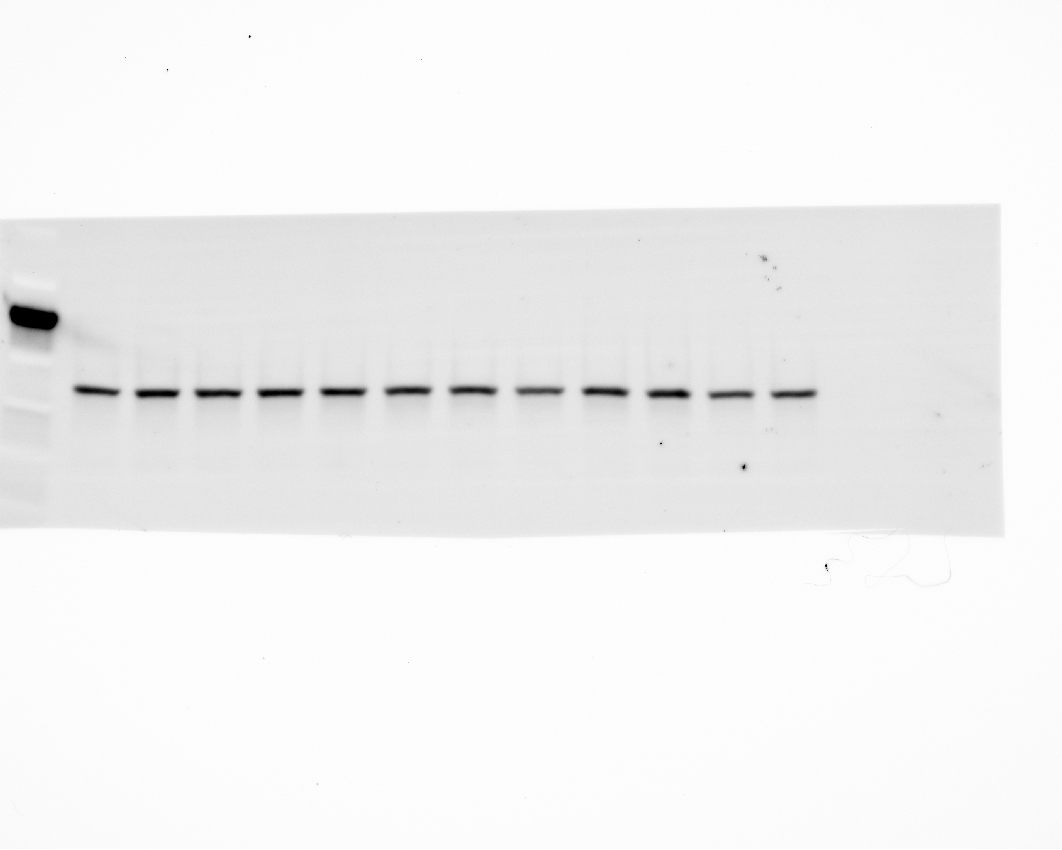

Supplement: Figure 4—source data 1. [file elife-85542-fig4-data1.zip › Figure 4 source data/Figure 4A/Figure 4A-actin for pAkt blot raw data.jpg]

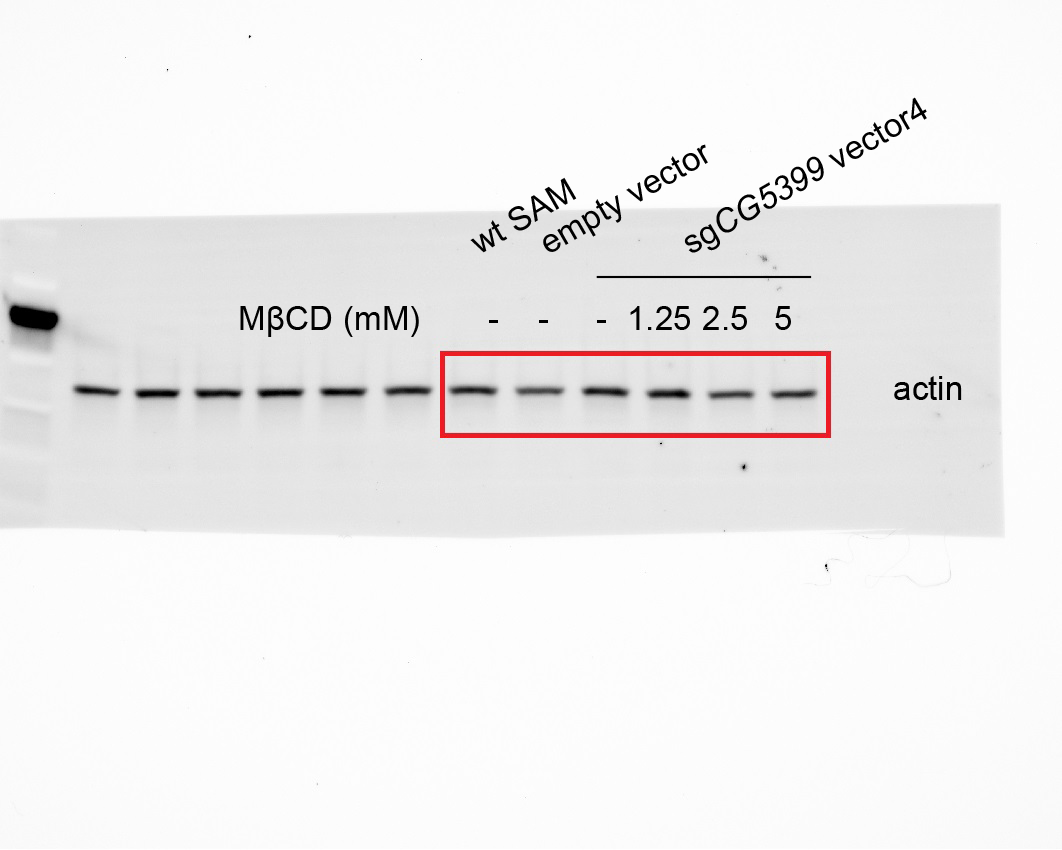

Supplement: Figure 4—source data 1. [file elife-85542-fig4-data1.zip › Figure 4 source data/Figure 4A/Figure 4A-actin for pAkt blot.tif]

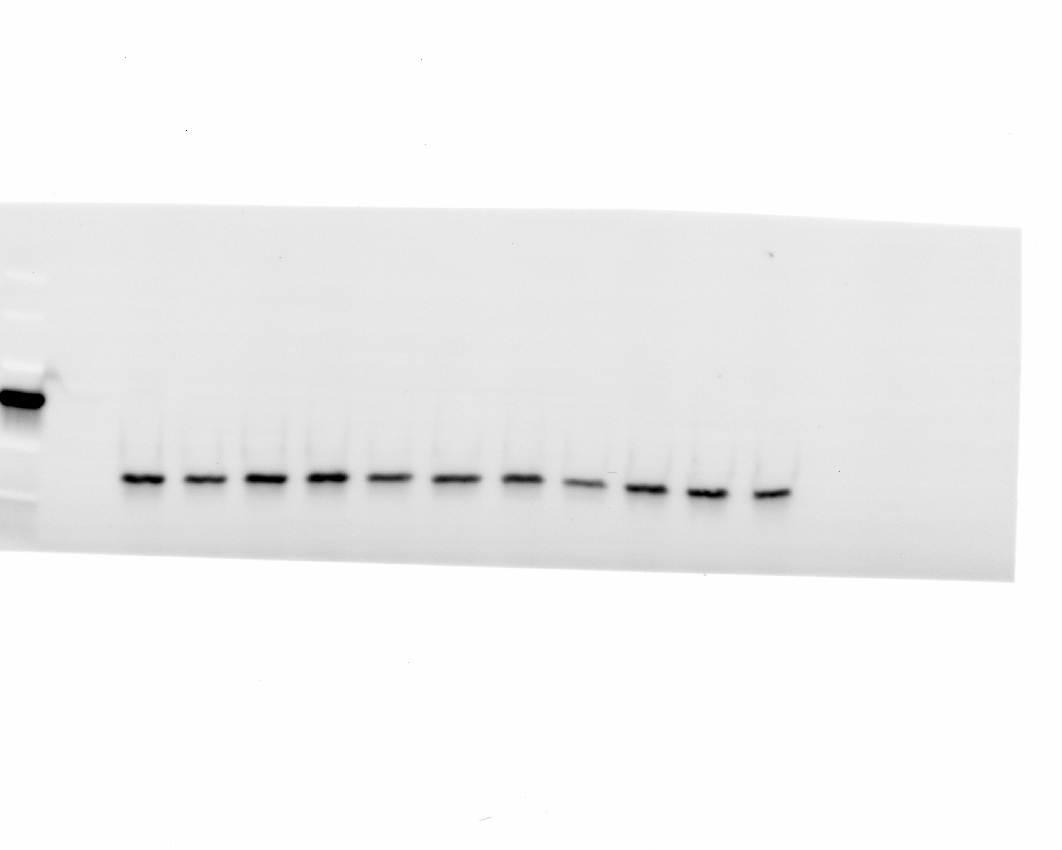

Supplement: Figure 4—source data 1. [file elife-85542-fig4-data1.zip › Figure 4 source data/Figure 4A/Figure 4A-actin for pInR blot raw data.jpg]

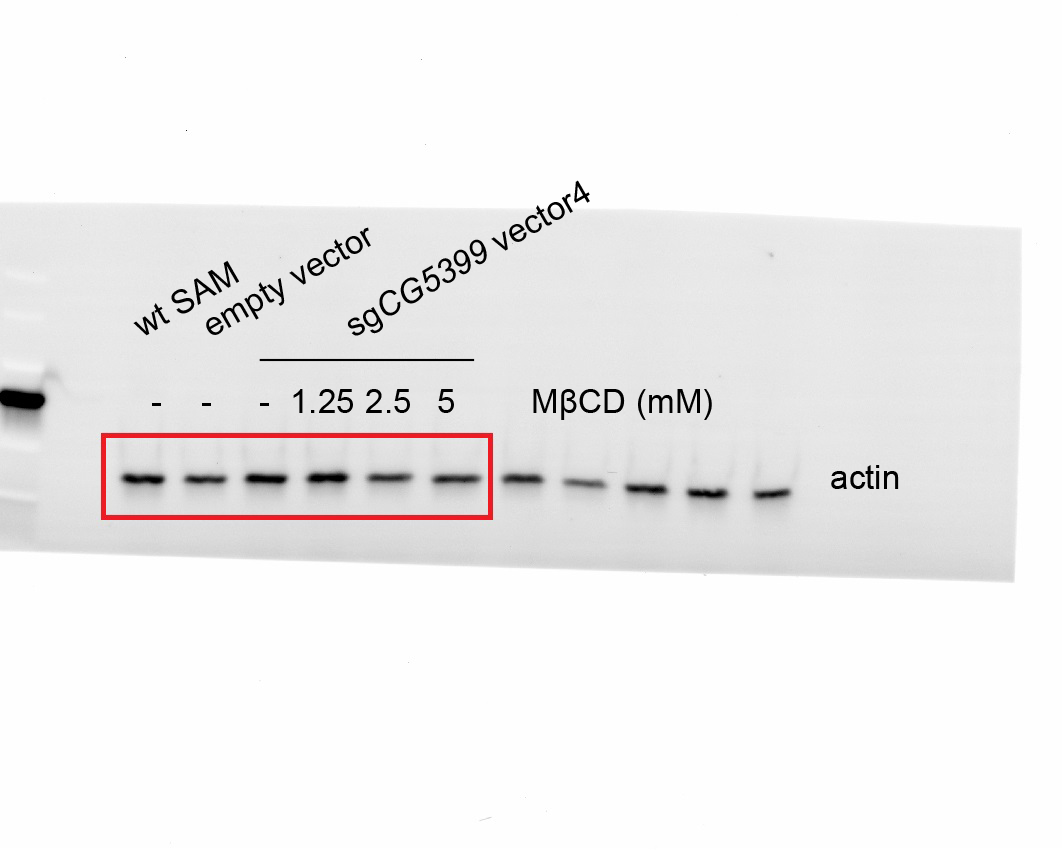

Supplement: Figure 4—source data 1. [file elife-85542-fig4-data1.zip › Figure 4 source data/Figure 4A/Figure 4A-actin for pInR blot.tif]

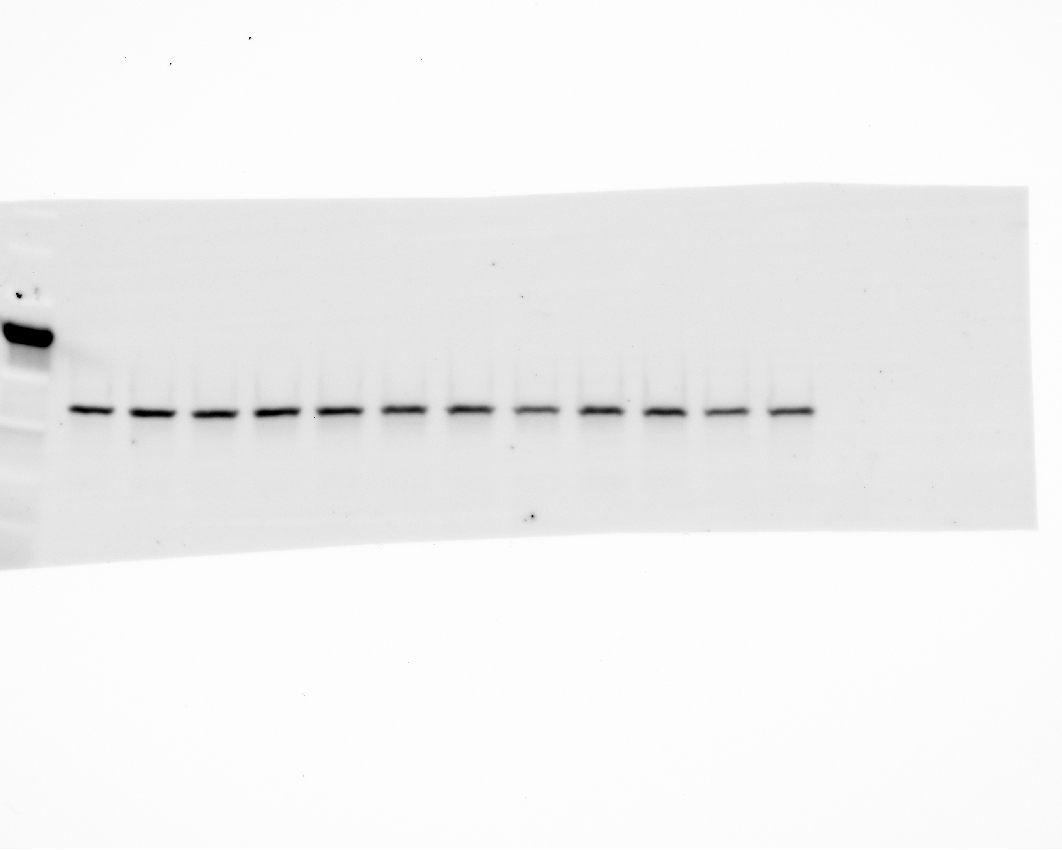

Supplement: Figure 4—source data 1. [file elife-85542-fig4-data1.zip › Figure 4 source data/Figure 4A/Figure 4A-actin for pS6 blot raw data.jpg]

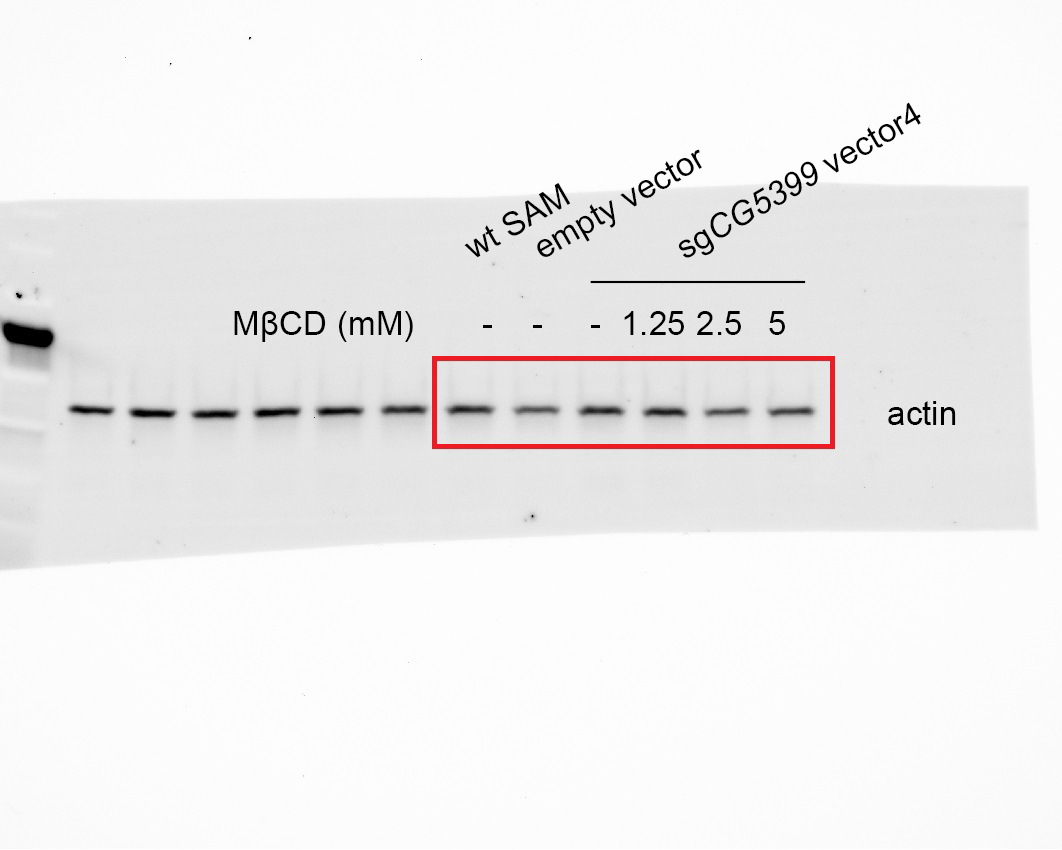

Supplement: Figure 4—source data 1. [file elife-85542-fig4-data1.zip › Figure 4 source data/Figure 4A/Figure 4A-actin for pS6 blot.tif]

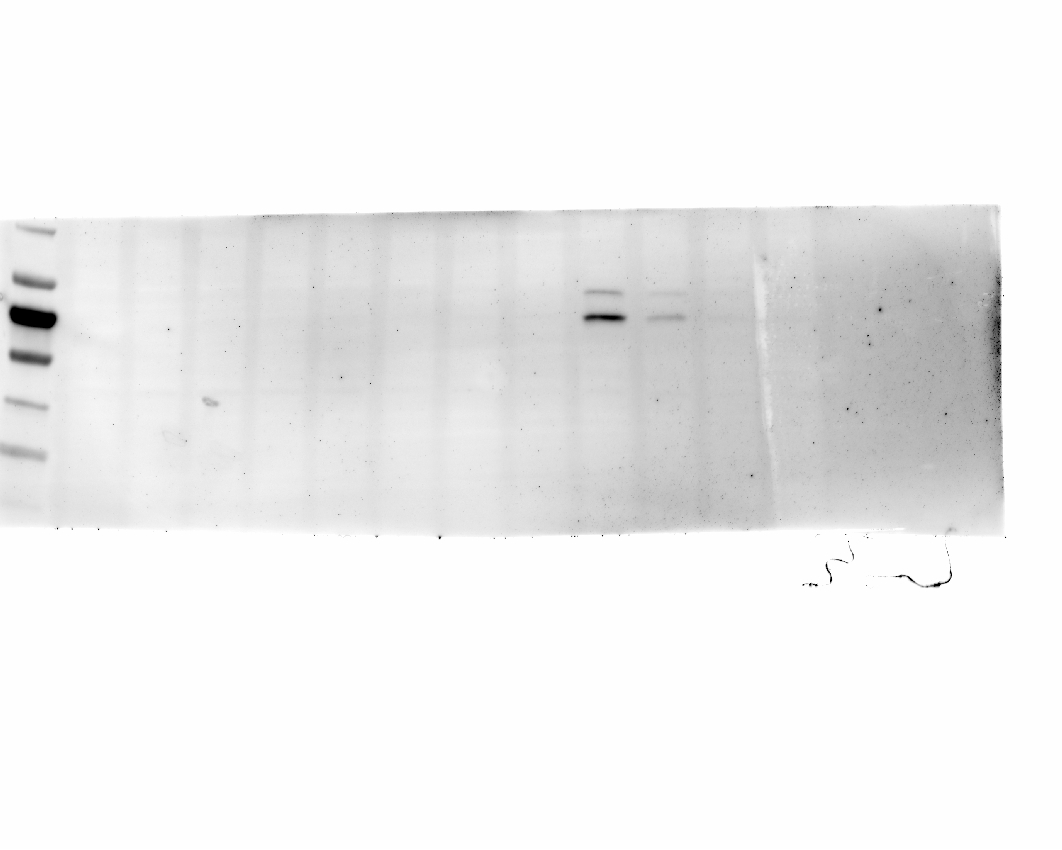

Supplement: Figure 4—source data 1. [file elife-85542-fig4-data1.zip › Figure 4 source data/Figure 4A/Figure 4A-pAkt raw data.jpg]

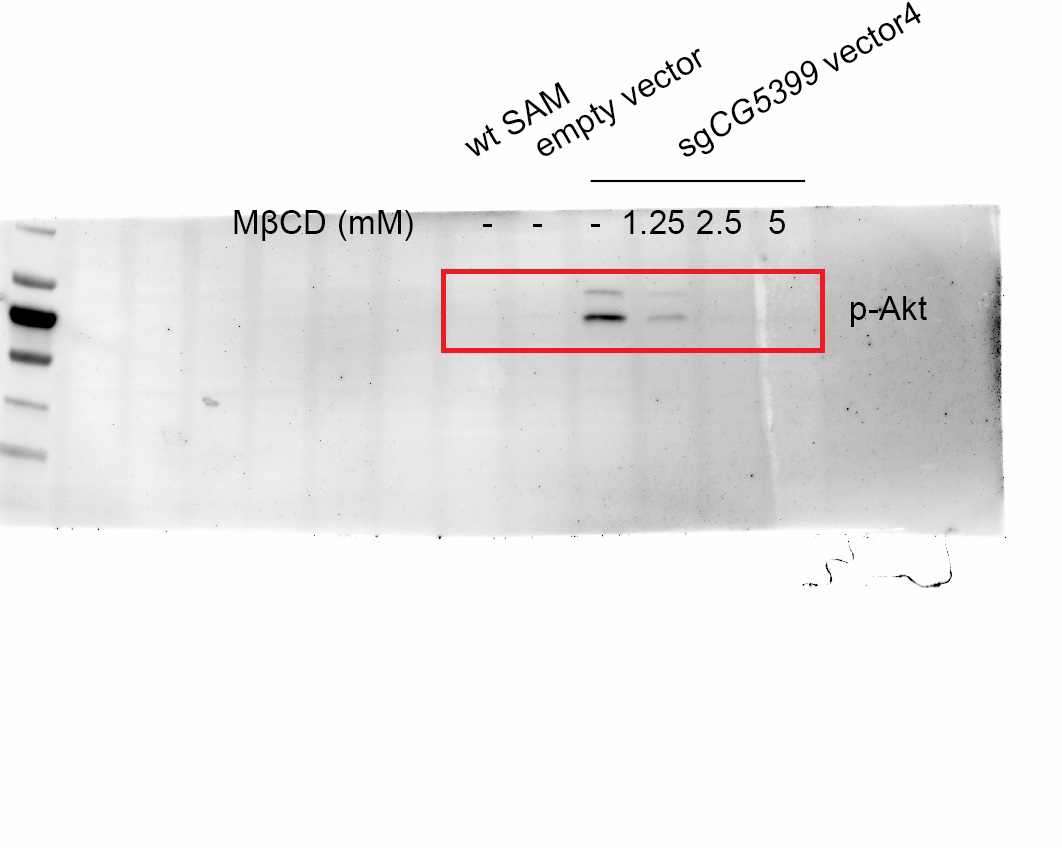

Supplement: Figure 4—source data 1. [file elife-85542-fig4-data1.zip › Figure 4 source data/Figure 4A/Figure 4A-pAkt.tif]

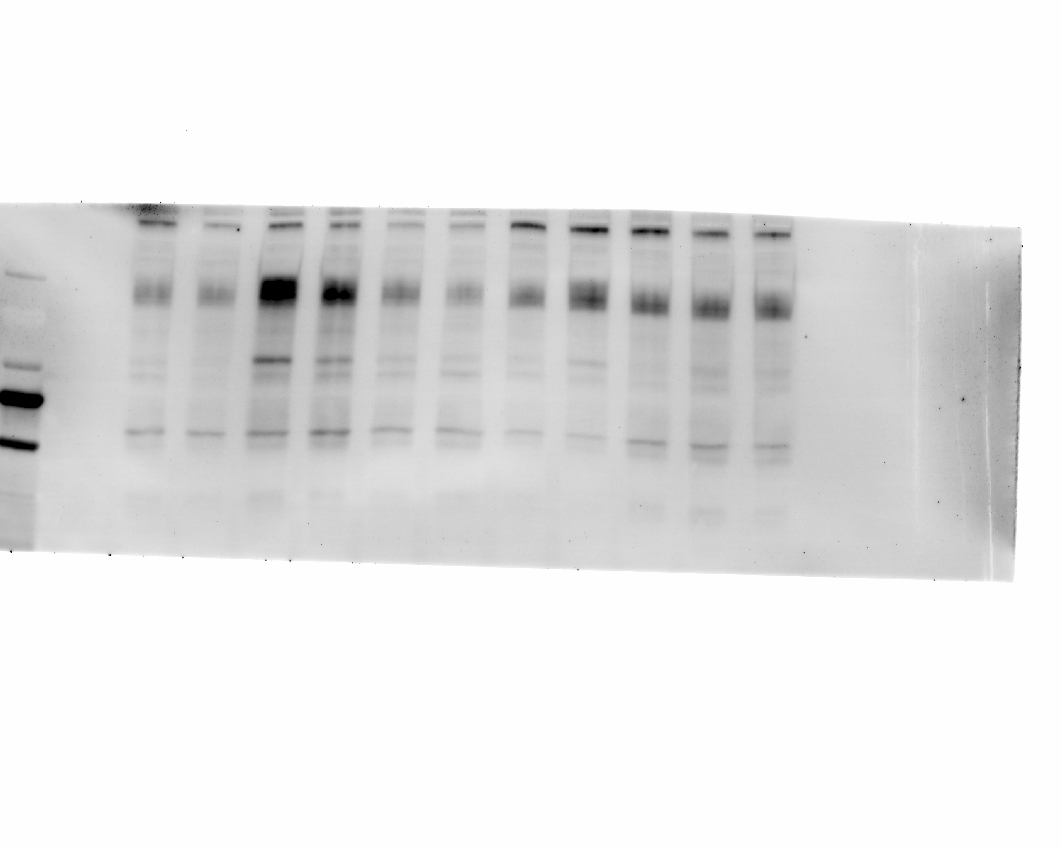

Supplement: Figure 4—source data 1. [file elife-85542-fig4-data1.zip › Figure 4 source data/Figure 4A/Figure 4A-pInR raw data.jpg]

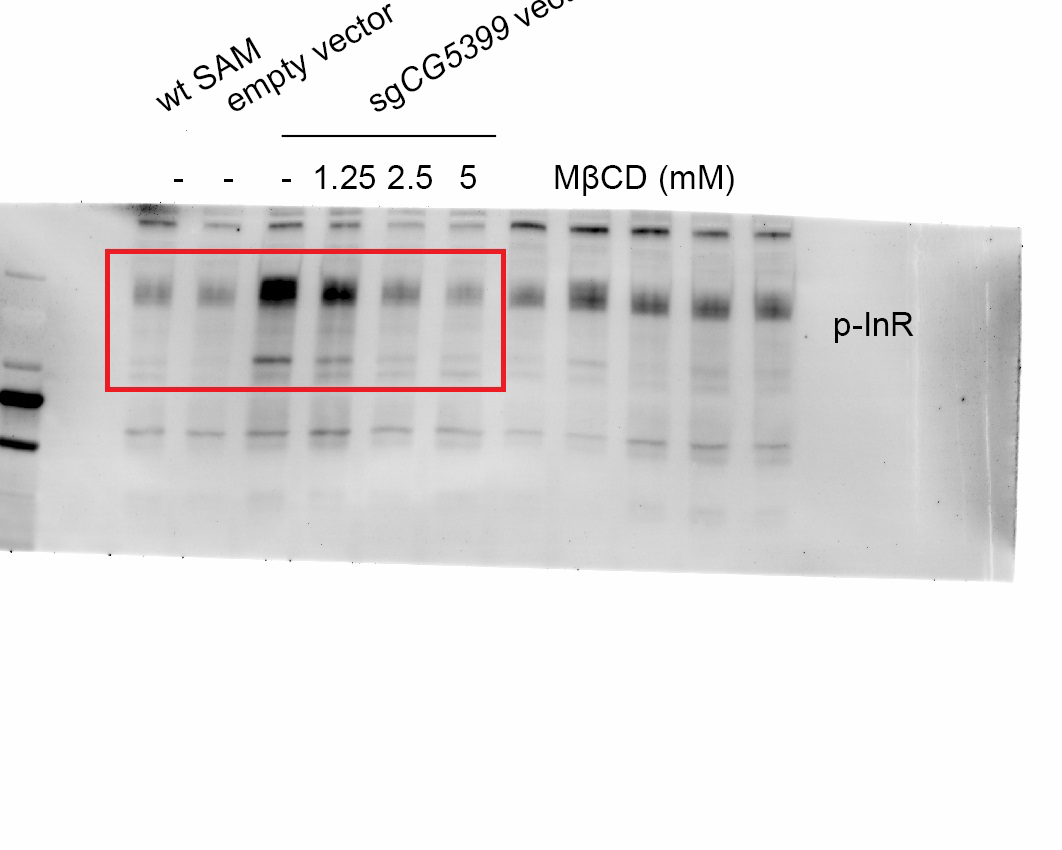

Supplement: Figure 4—source data 1. [file elife-85542-fig4-data1.zip › Figure 4 source data/Figure 4A/Figure 4A-pInR.tif]

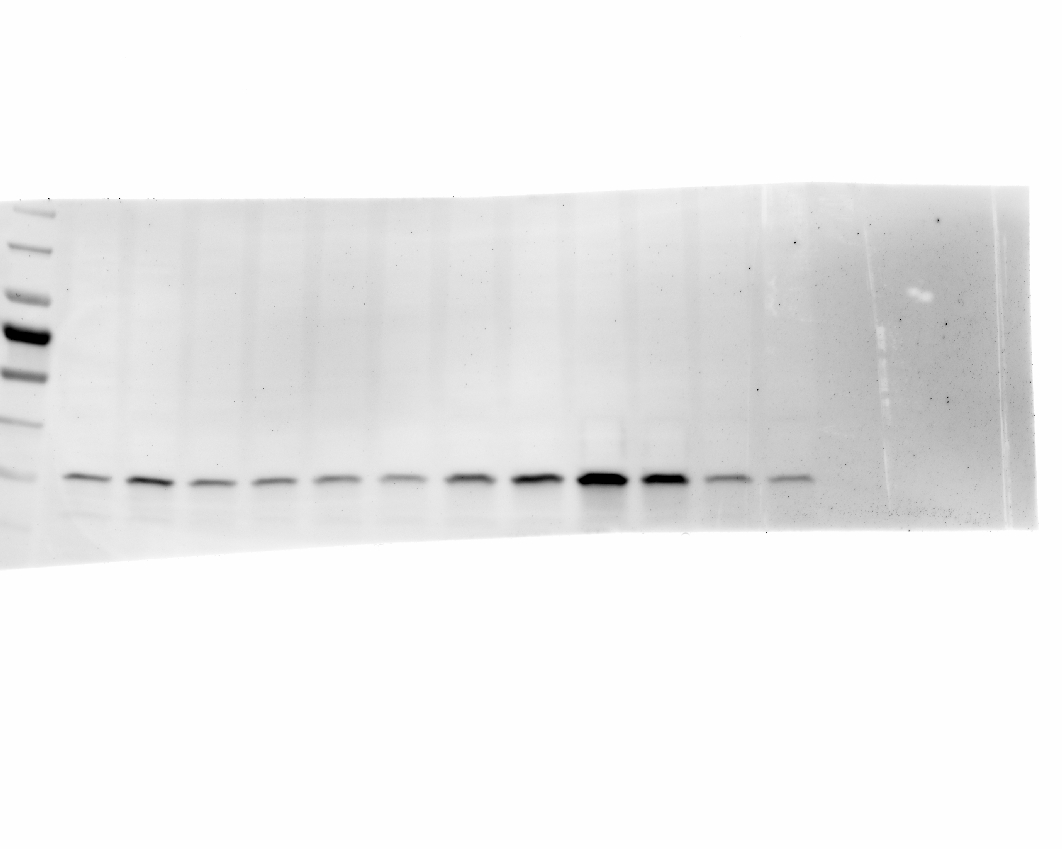

Supplement: Figure 4—source data 1. [file elife-85542-fig4-data1.zip › Figure 4 source data/Figure 4A/Figure 4A-pS6 raw data.jpg]

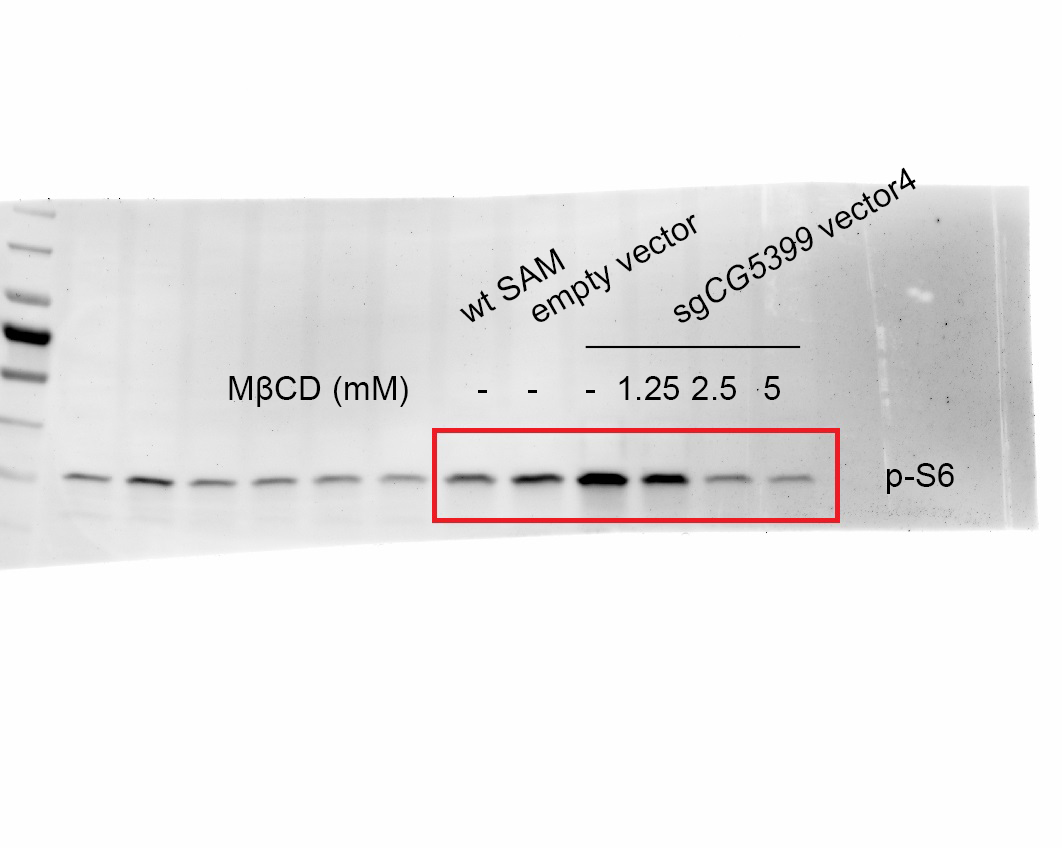

Supplement: Figure 4—source data 1. [file elife-85542-fig4-data1.zip › Figure 4 source data/Figure 4A/Figure 4A-pS6.tif]

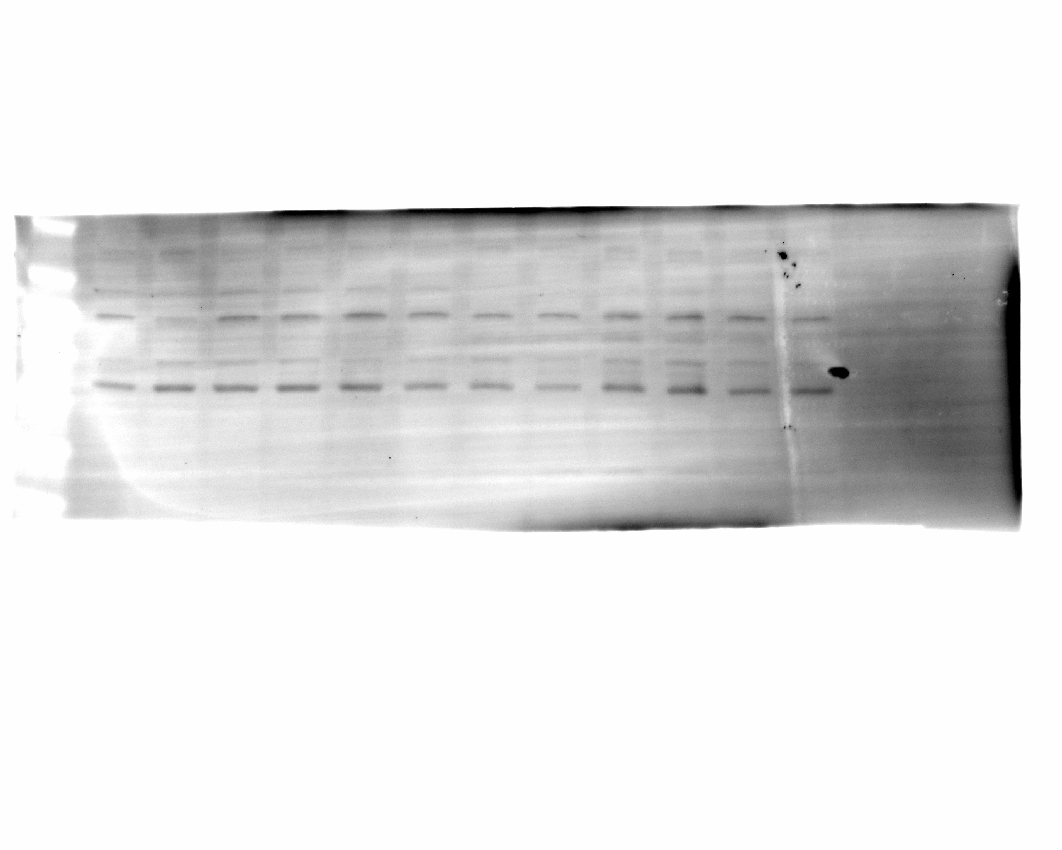

Supplement: Figure 4—source data 1. [file elife-85542-fig4-data1.zip › Figure 4 source data/Figure 4A/Figure 4A-total Akt raw data.jpg]

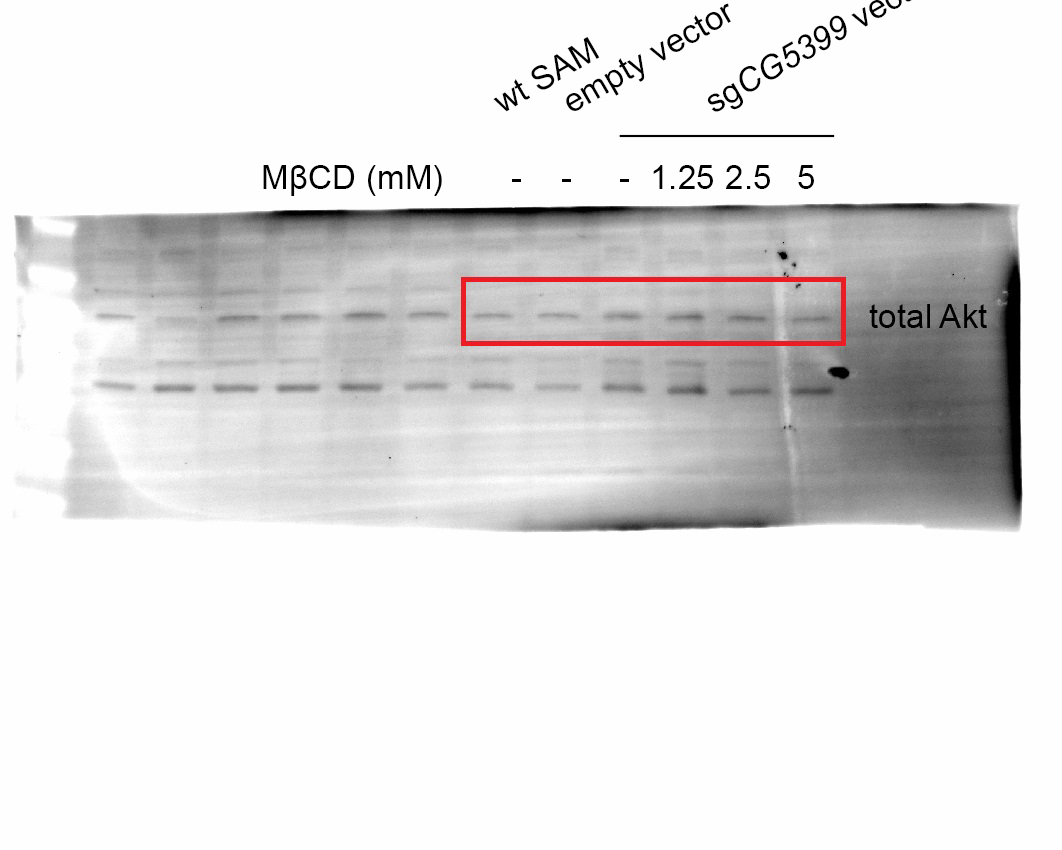

Supplement: Figure 4—source data 1. [file elife-85542-fig4-data1.zip › Figure 4 source data/Figure 4A/Figure 4A-total Akt.tif]

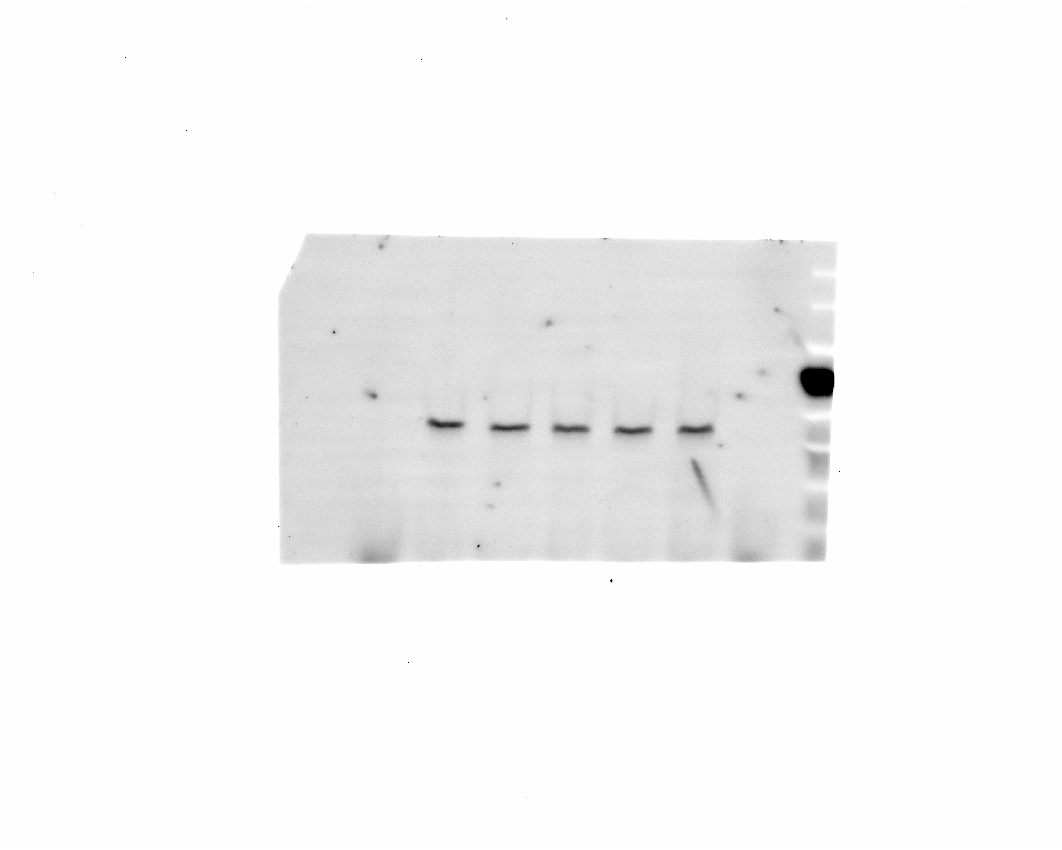

Supplement: Figure 4—source data 1. [file elife-85542-fig4-data1.zip › Figure 4 source data/Figure 4B/Figure 4B-actin for pAkt blot raw data.jpg]

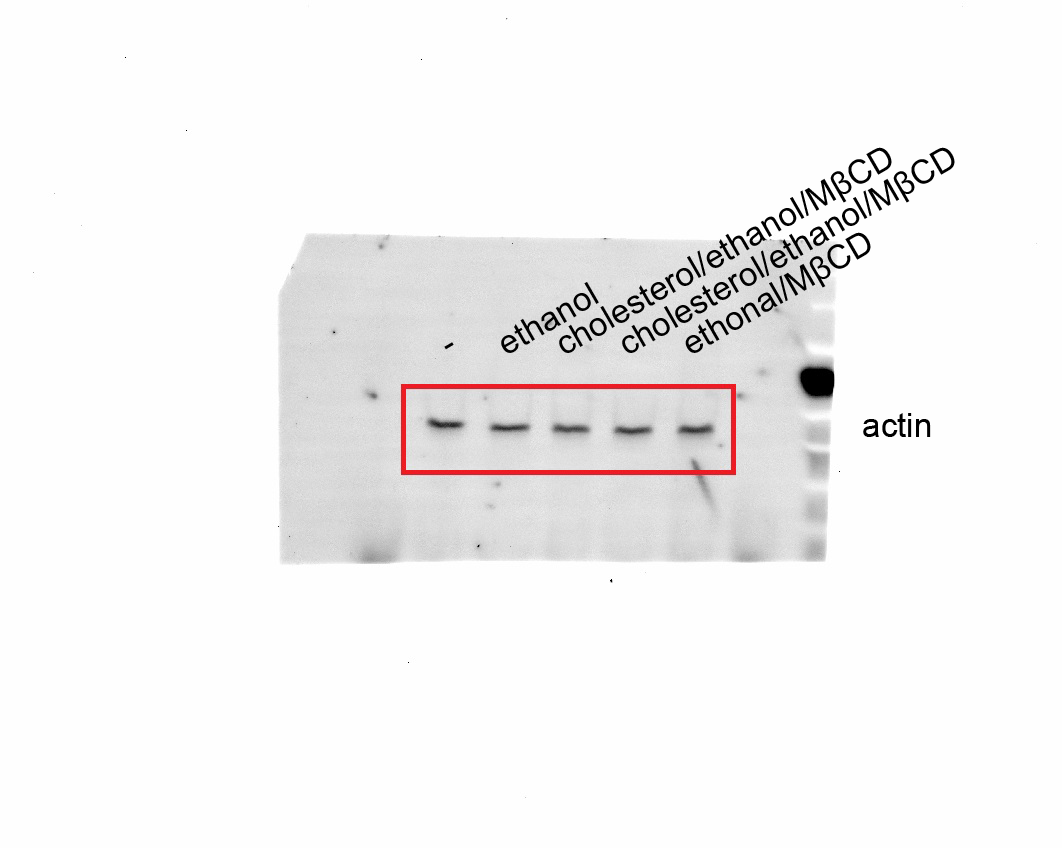

Supplement: Figure 4—source data 1. [file elife-85542-fig4-data1.zip › Figure 4 source data/Figure 4B/Figure 4B-actin for pAkt blot.tif]

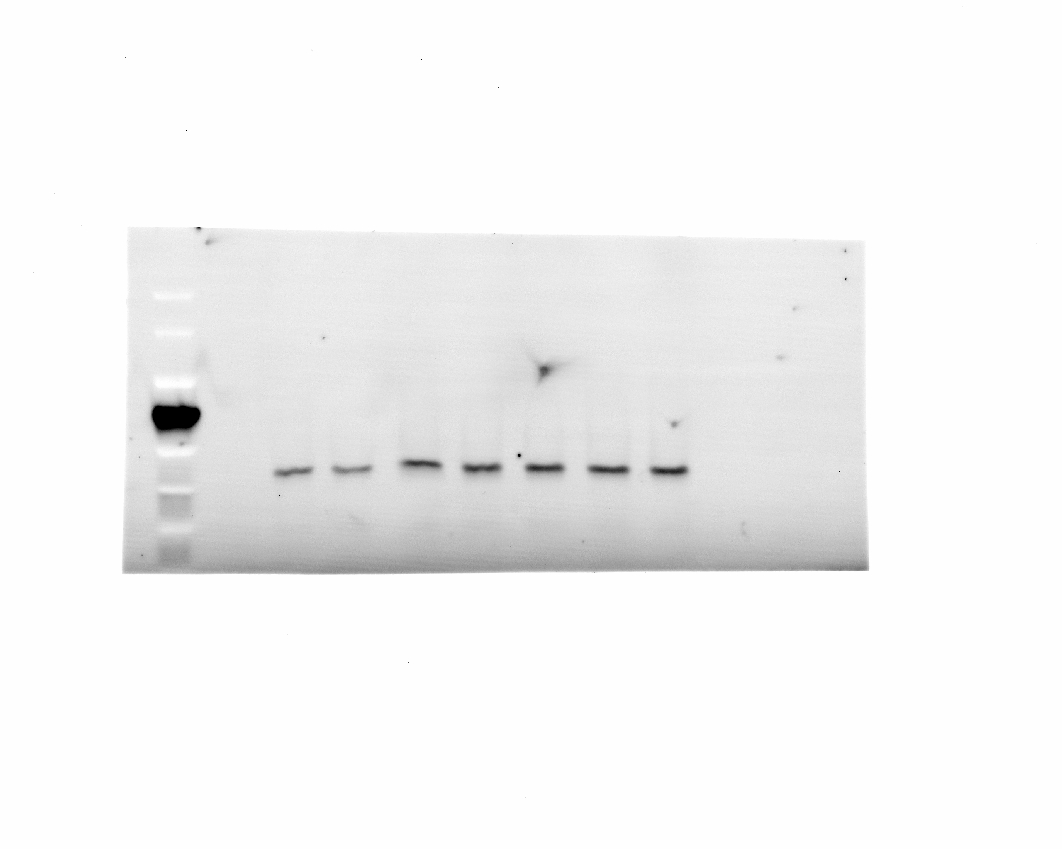

Supplement: Figure 4—source data 1. [file elife-85542-fig4-data1.zip › Figure 4 source data/Figure 4B/Figure 4B-actin for pInR blot raw data.jpg]

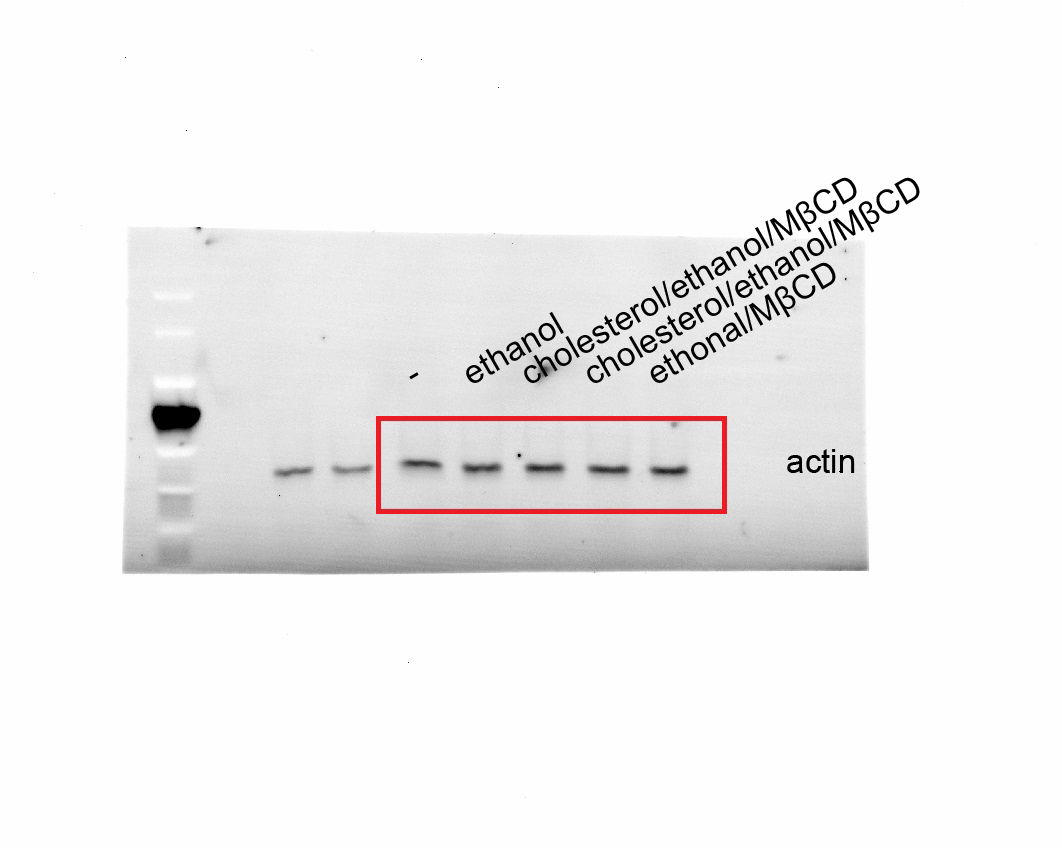

Supplement: Figure 4—source data 1. [file elife-85542-fig4-data1.zip › Figure 4 source data/Figure 4B/Figure 4B-actin for pInR blot.tif]

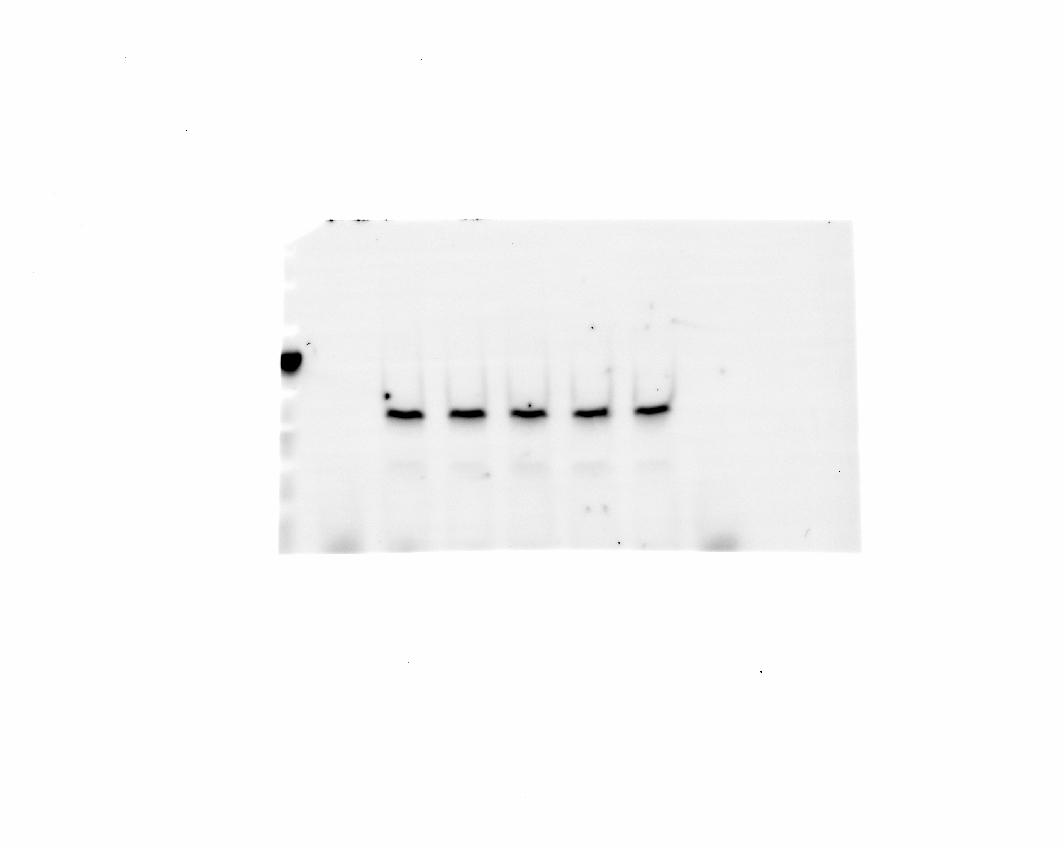

Supplement: Figure 4—source data 1. [file elife-85542-fig4-data1.zip › Figure 4 source data/Figure 4B/Figure 4B-actin for pS6 blot raw data.jpg]

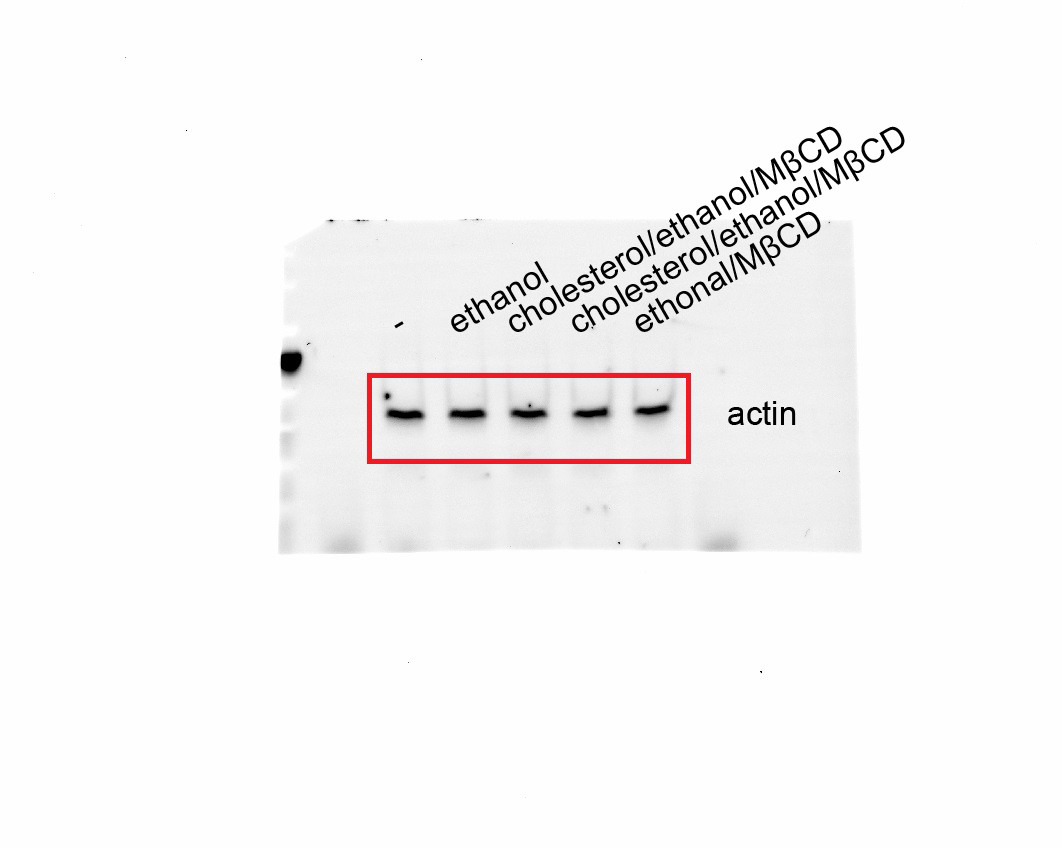

Supplement: Figure 4—source data 1. [file elife-85542-fig4-data1.zip › Figure 4 source data/Figure 4B/Figure 4B-actin for pS6 blot.tif]

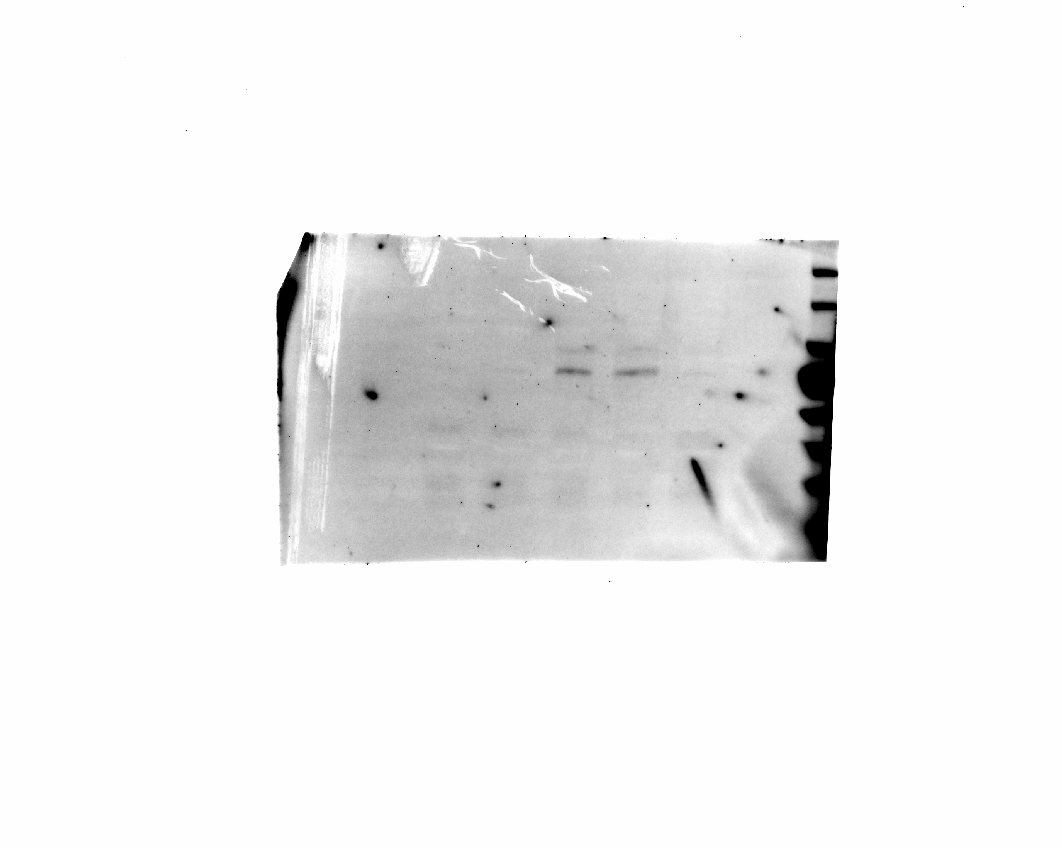

Supplement: Figure 4—source data 1. [file elife-85542-fig4-data1.zip › Figure 4 source data/Figure 4B/Figure 4B-pAkt raw data.jpg]

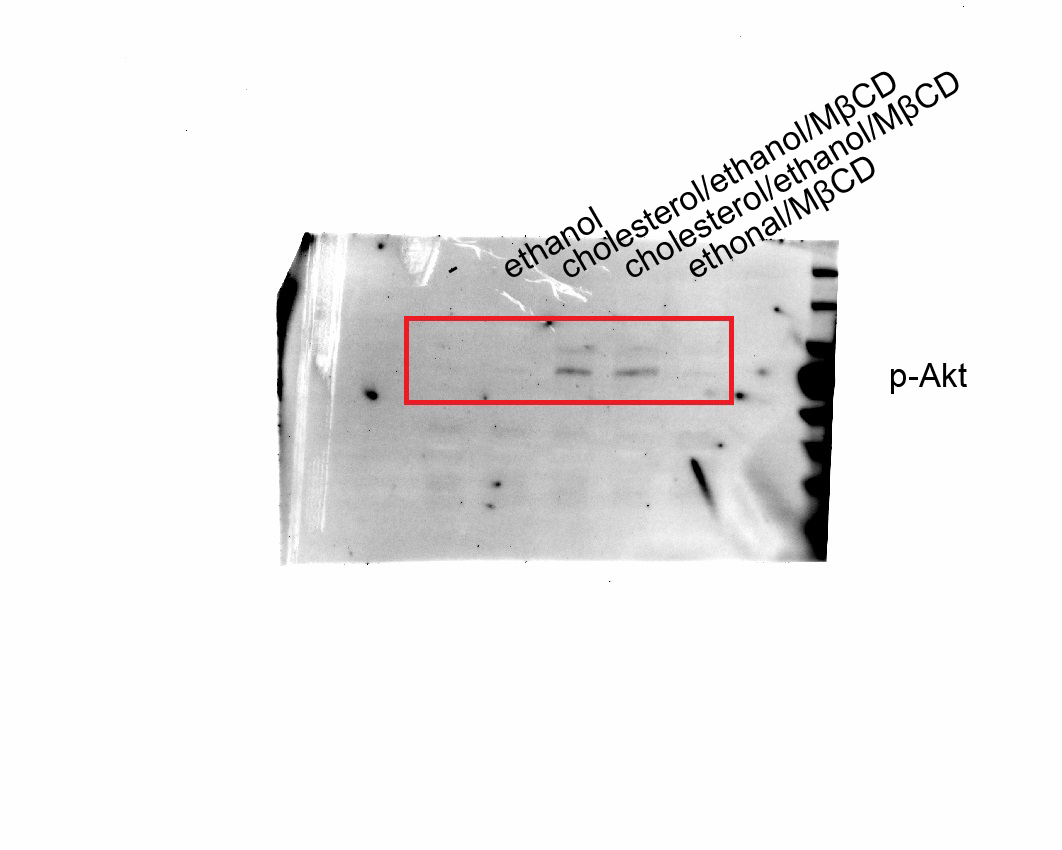

Supplement: Figure 4—source data 1. [file elife-85542-fig4-data1.zip › Figure 4 source data/Figure 4B/Figure 4B-pAkt.tif]
