# Supplementary figures and images for: Pooled genome-wide CRISPR activation screening for rapamycin resistance genes in Drosophila cells (part 2 of 2)
Source: eLife. 2023 Apr 20;12:e85542. doi: 10.7554/eLife.85542 (PMC10118385; doi:10.7554/eLife.85542)

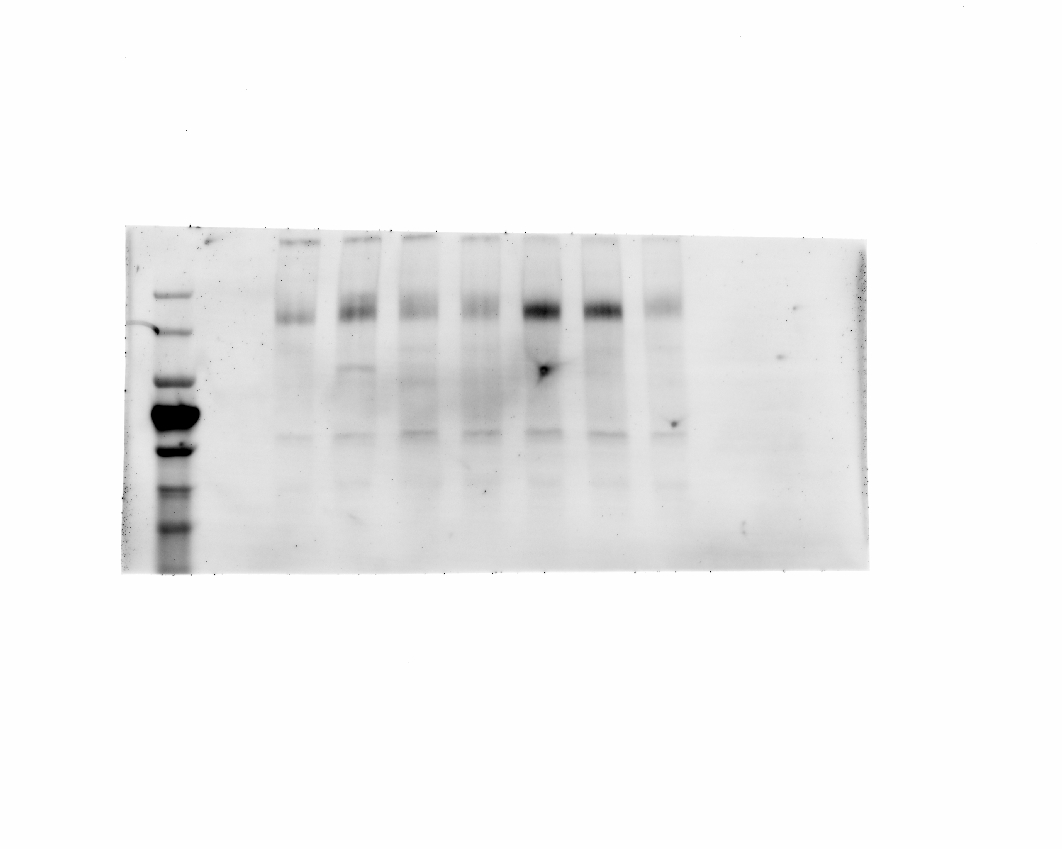

Supplement: Figure 4—source data 1. [file elife-85542-fig4-data1.zip › Figure 4 source data/Figure 4B/Figure 4B-pInR raw data.jpg]

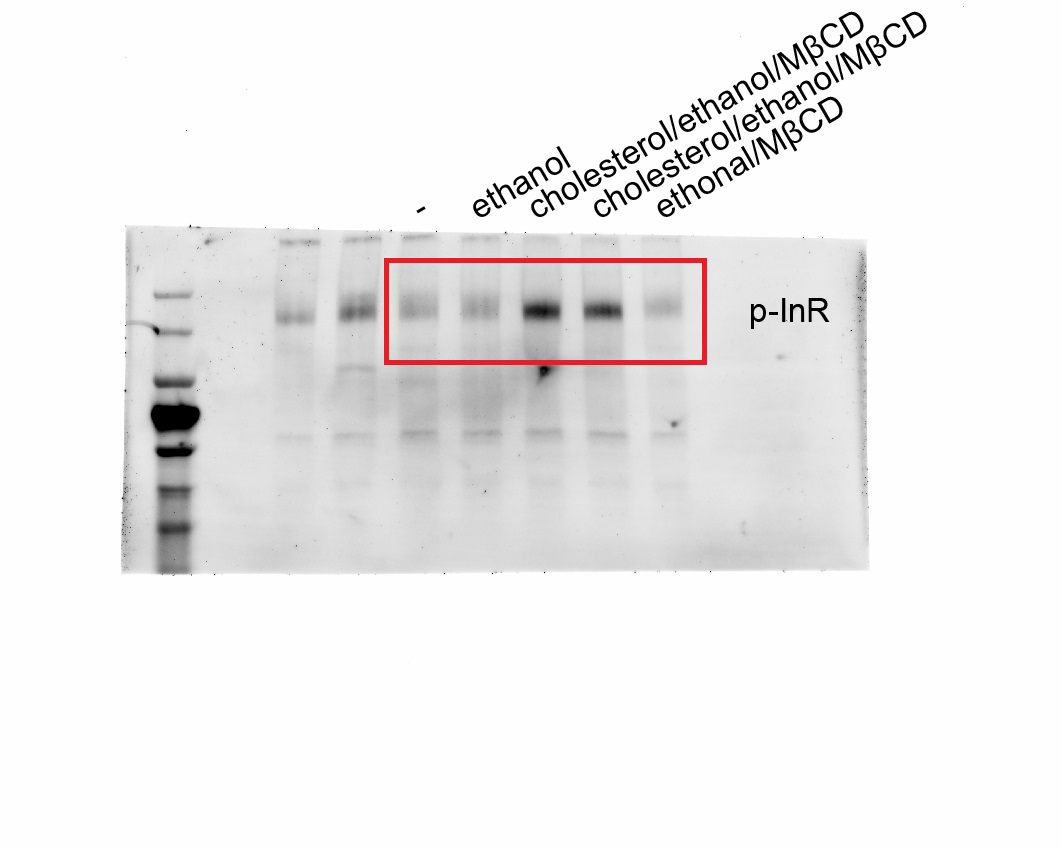

Supplement: Figure 4—source data 1. [file elife-85542-fig4-data1.zip › Figure 4 source data/Figure 4B/Figure 4B-pInR.tif]

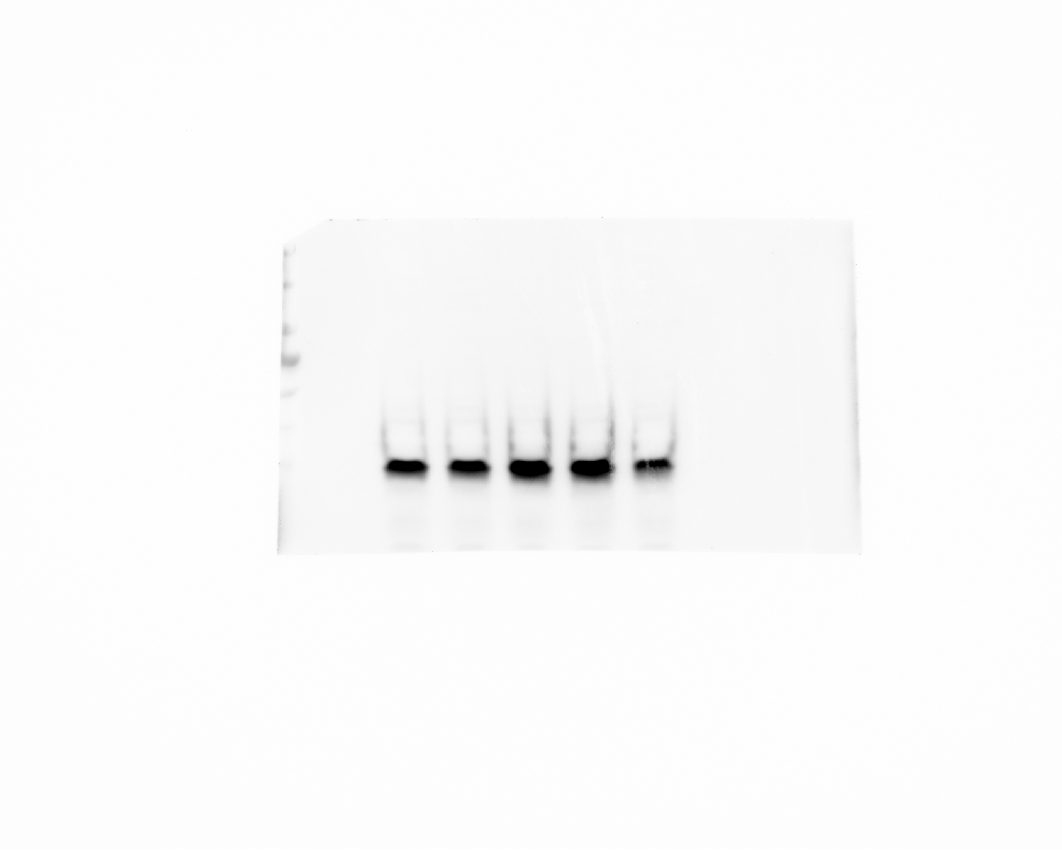

Supplement: Figure 4—source data 1. [file elife-85542-fig4-data1.zip › Figure 4 source data/Figure 4B/Figure 4B-pS6 raw data.jpg]

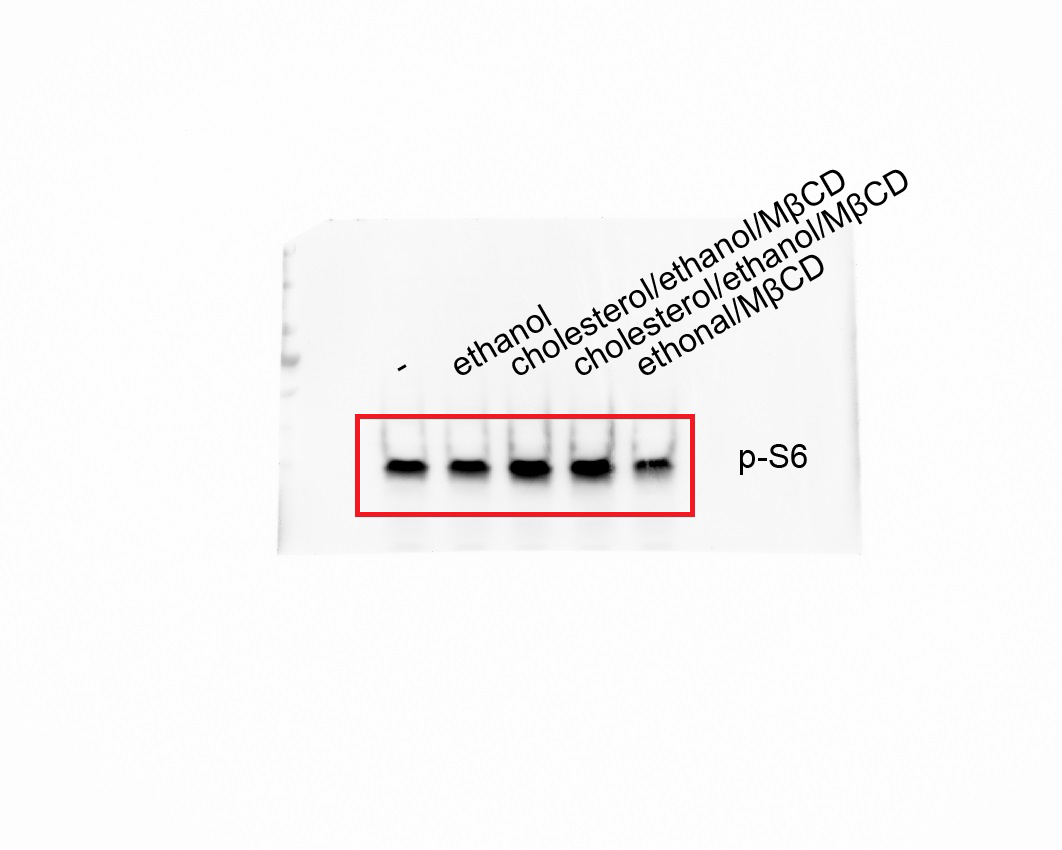

Supplement: Figure 4—source data 1. [file elife-85542-fig4-data1.zip › Figure 4 source data/Figure 4B/Figure 4B-pS6.tif]

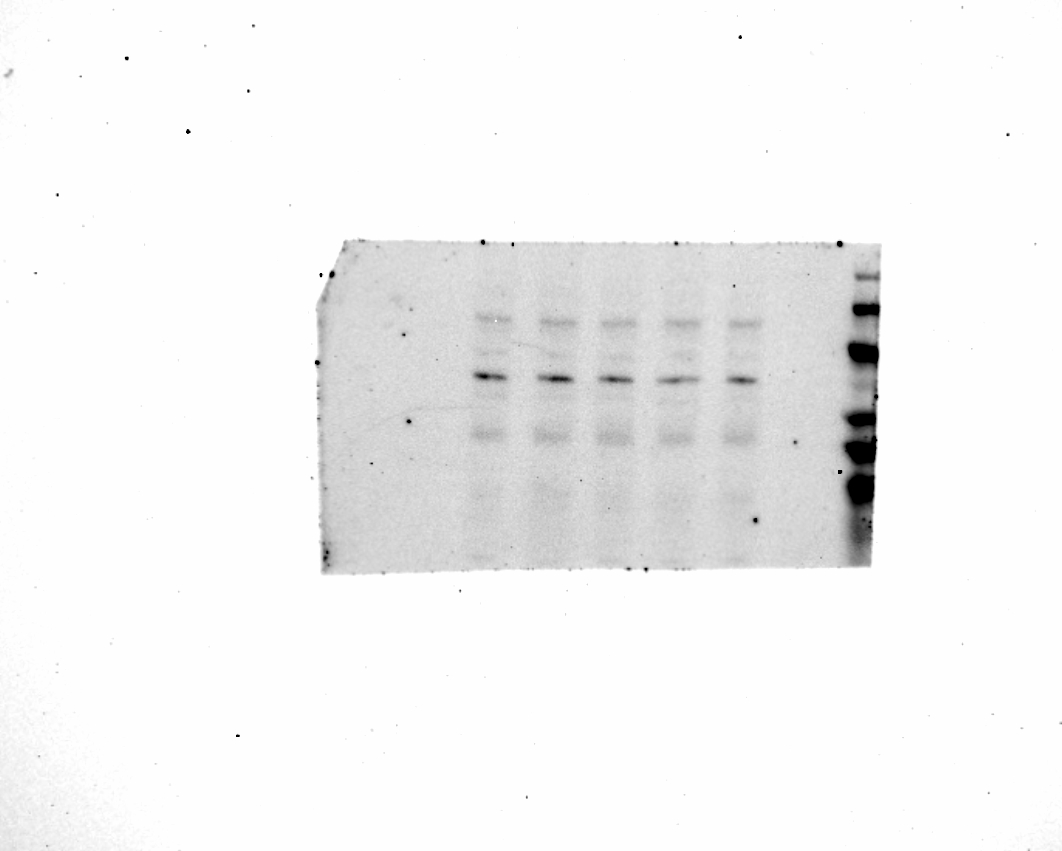

Supplement: Figure 4—source data 1. [file elife-85542-fig4-data1.zip › Figure 4 source data/Figure 4B/Figure 4B-total Akt raw data.jpg]

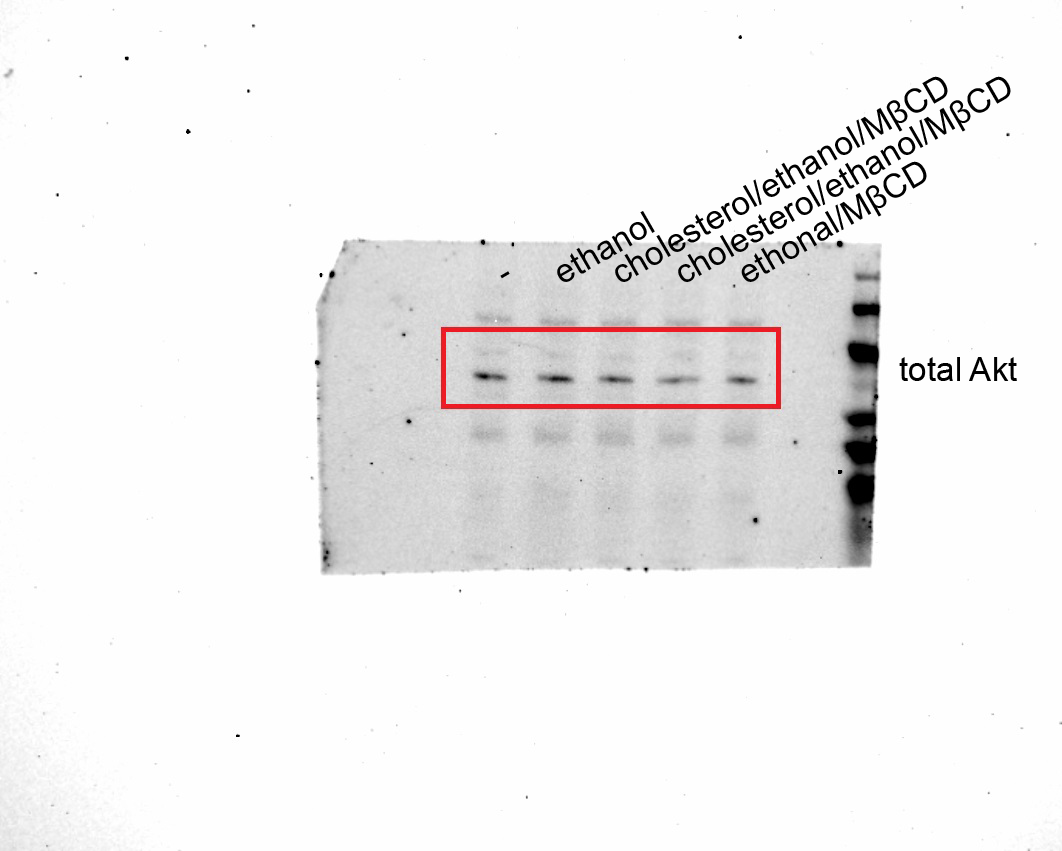

Supplement: Figure 4—source data 1. [file elife-85542-fig4-data1.zip › Figure 4 source data/Figure 4B/Figure 4B-total Akt.tif]

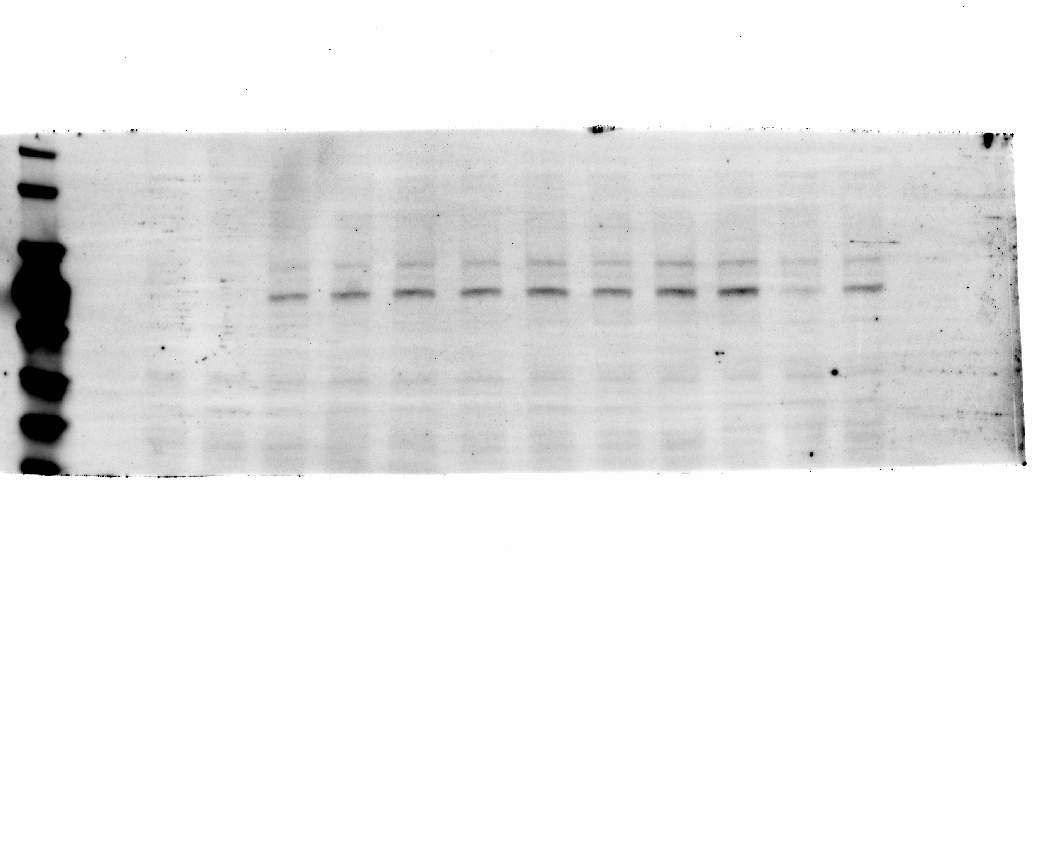

Supplement: Figure 4—source data 1. [file elife-85542-fig4-data1.zip › Figure 4 source data/Figure 4C/Figure 4C- pAkt for dsFlo raw data.jpg]

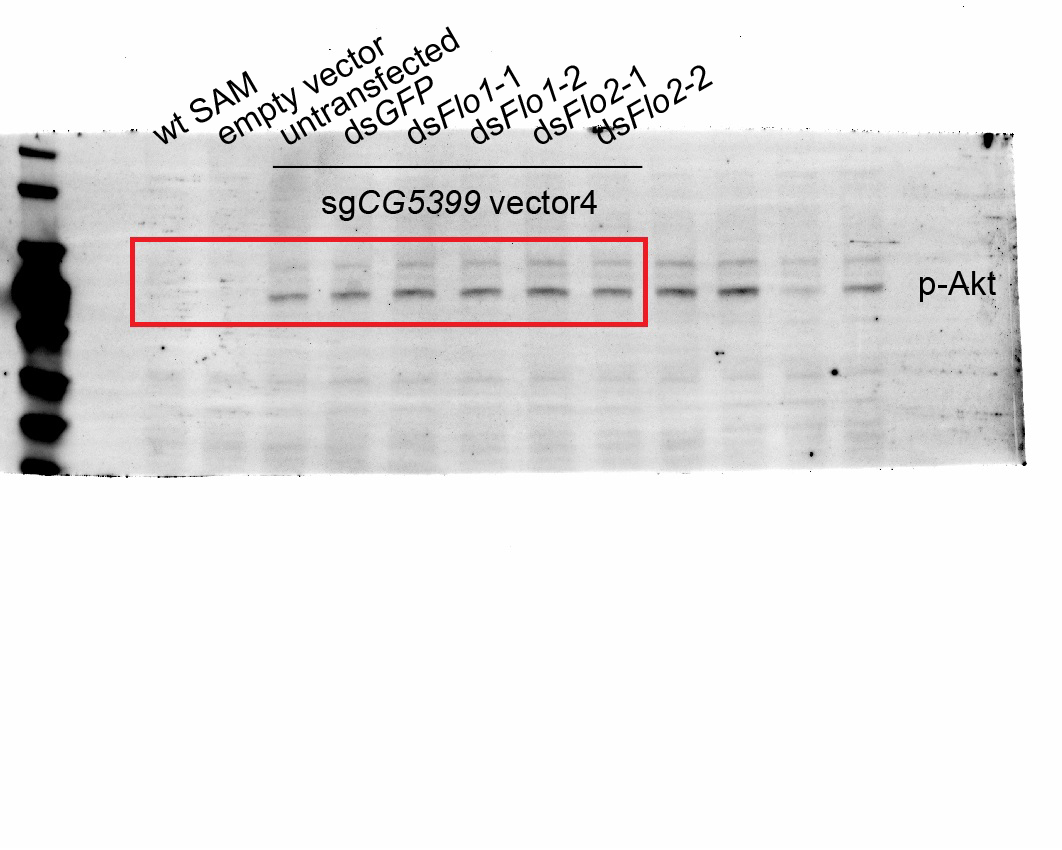

Supplement: Figure 4—source data 1. [file elife-85542-fig4-data1.zip › Figure 4 source data/Figure 4C/Figure 4C- pAkt for dsFlo.tif]

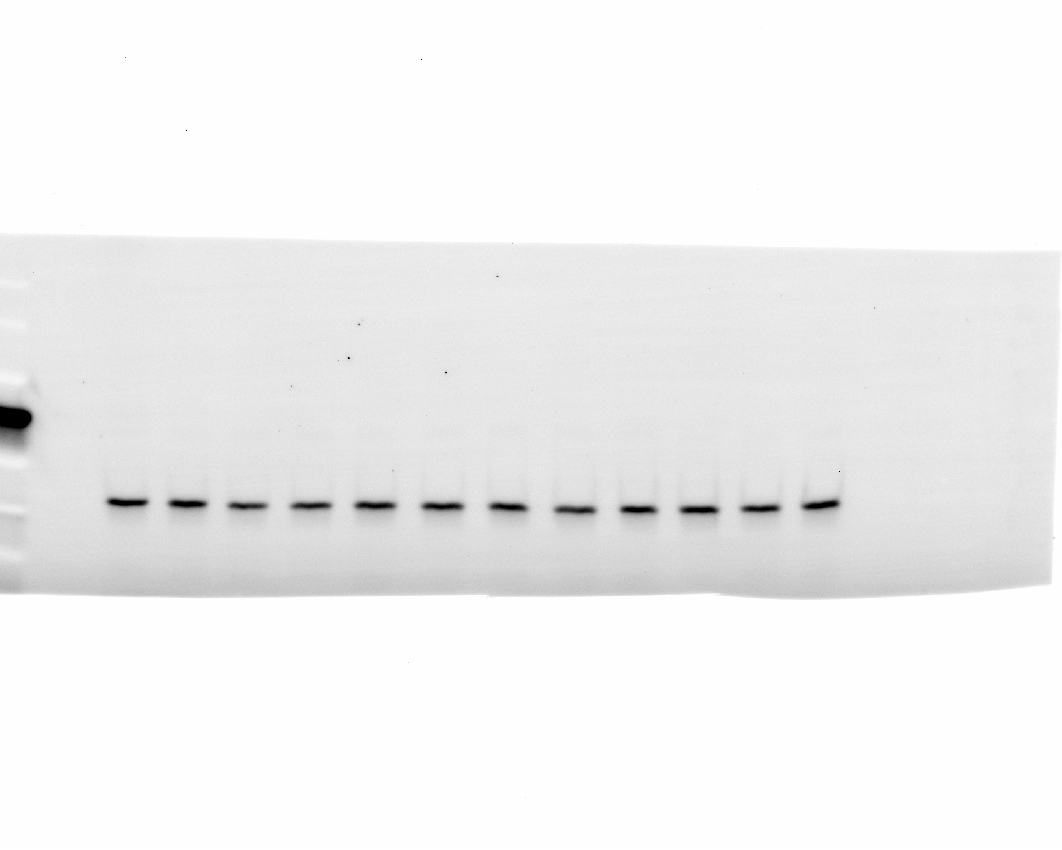

Supplement: Figure 4—source data 1. [file elife-85542-fig4-data1.zip › Figure 4 source data/Figure 4C/Figure 4C-actin for dsChc raw data.jpg]

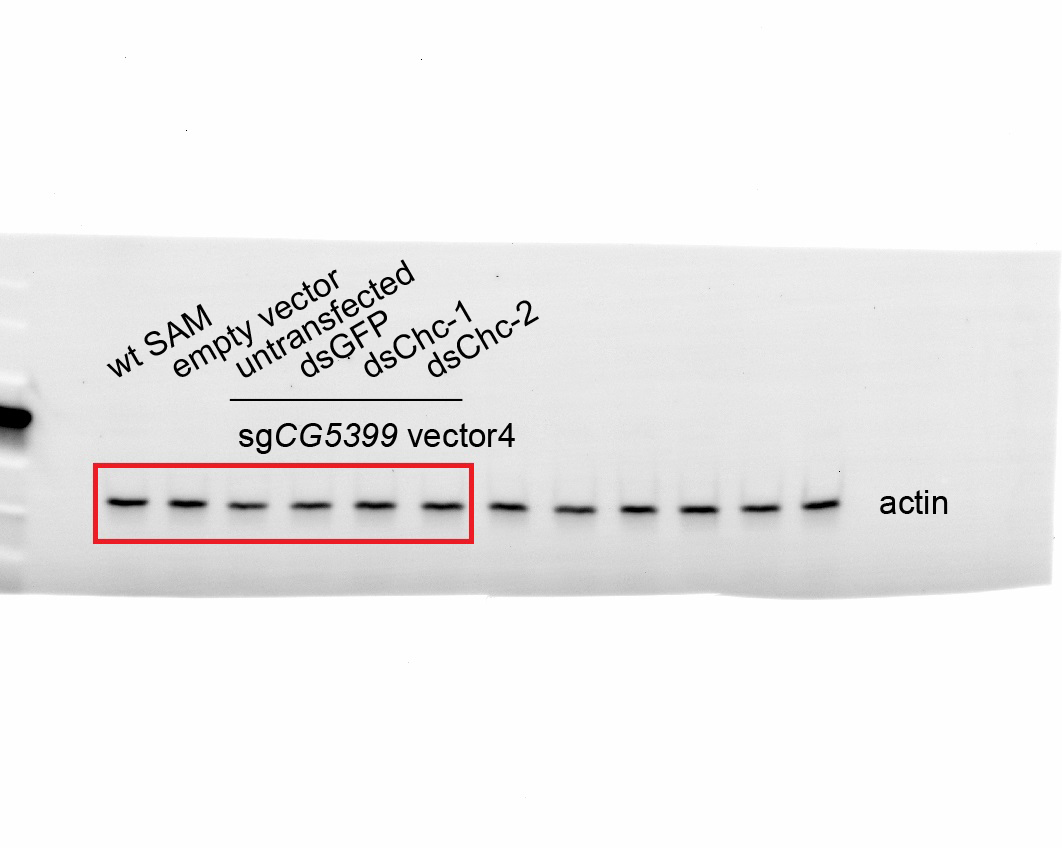

Supplement: Figure 4—source data 1. [file elife-85542-fig4-data1.zip › Figure 4 source data/Figure 4C/Figure 4C-actin for dsChc.tif]

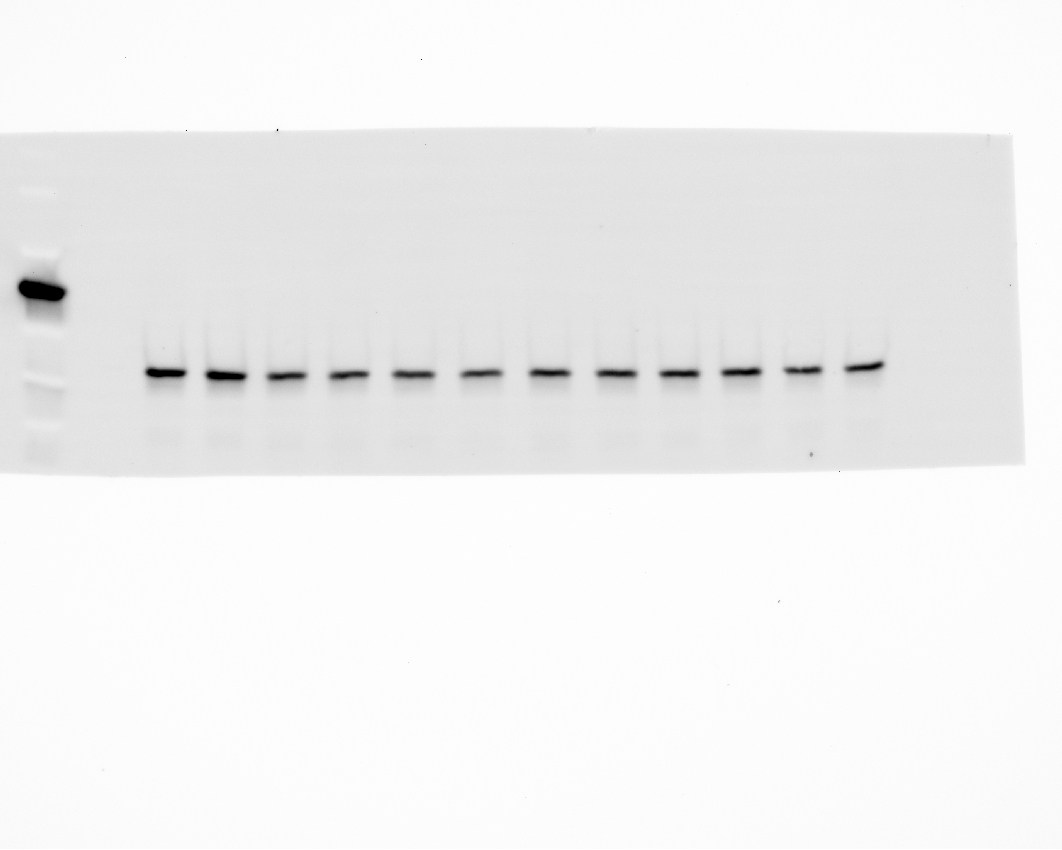

Supplement: Figure 4—source data 1. [file elife-85542-fig4-data1.zip › Figure 4 source data/Figure 4C/Figure 4C-actin for dsFlo raw data.jpg]

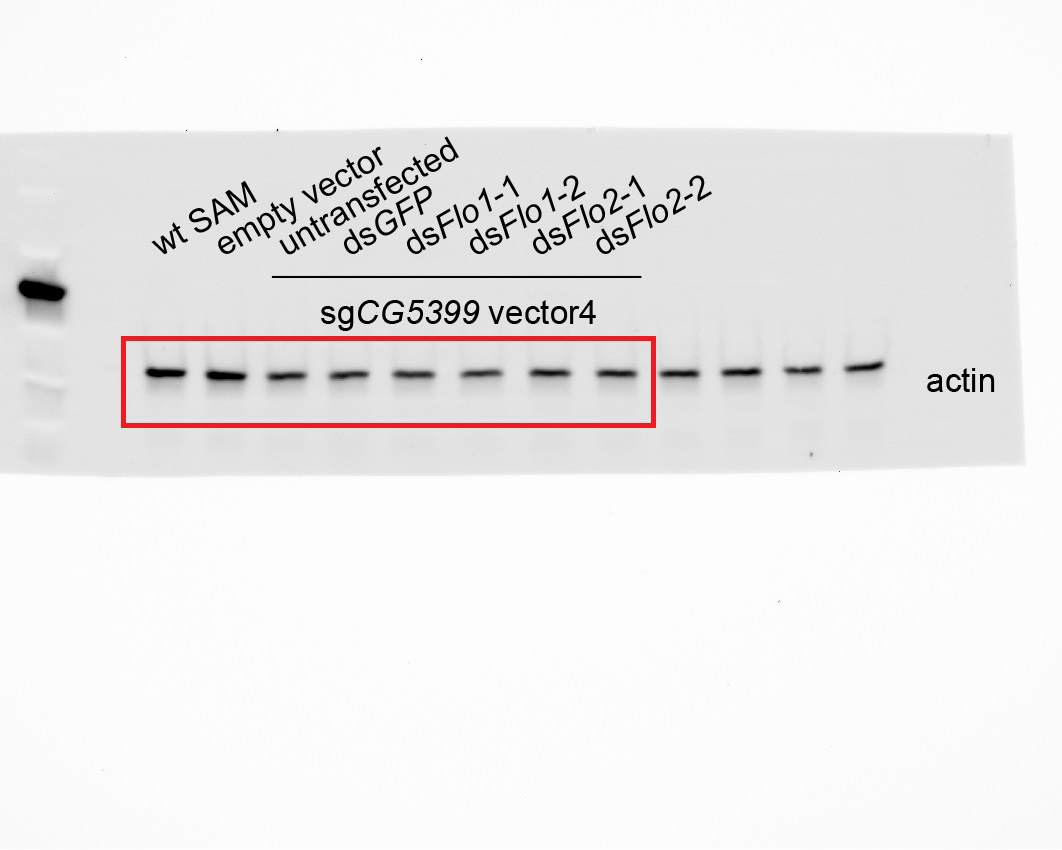

Supplement: Figure 4—source data 1. [file elife-85542-fig4-data1.zip › Figure 4 source data/Figure 4C/Figure 4C-actin for dsFlo.tif]

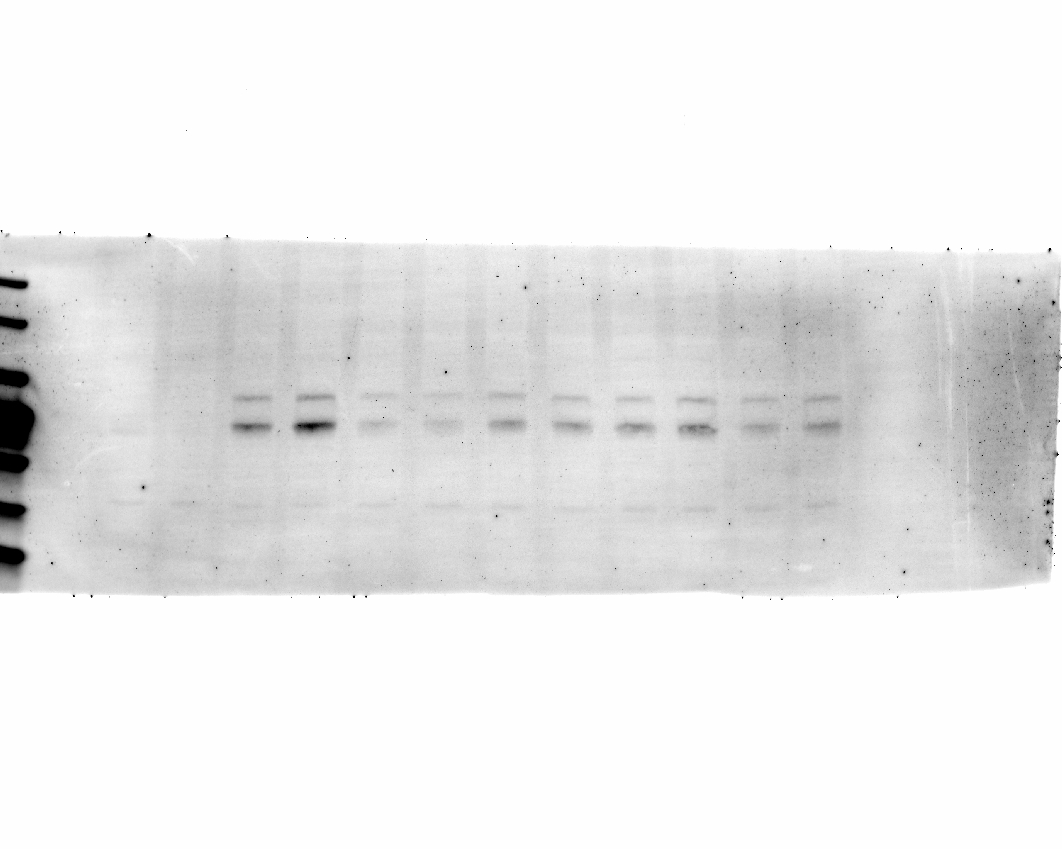

Supplement: Figure 4—source data 1. [file elife-85542-fig4-data1.zip › Figure 4 source data/Figure 4C/Figure 4C-pAkt dsChc raw data.jpg]

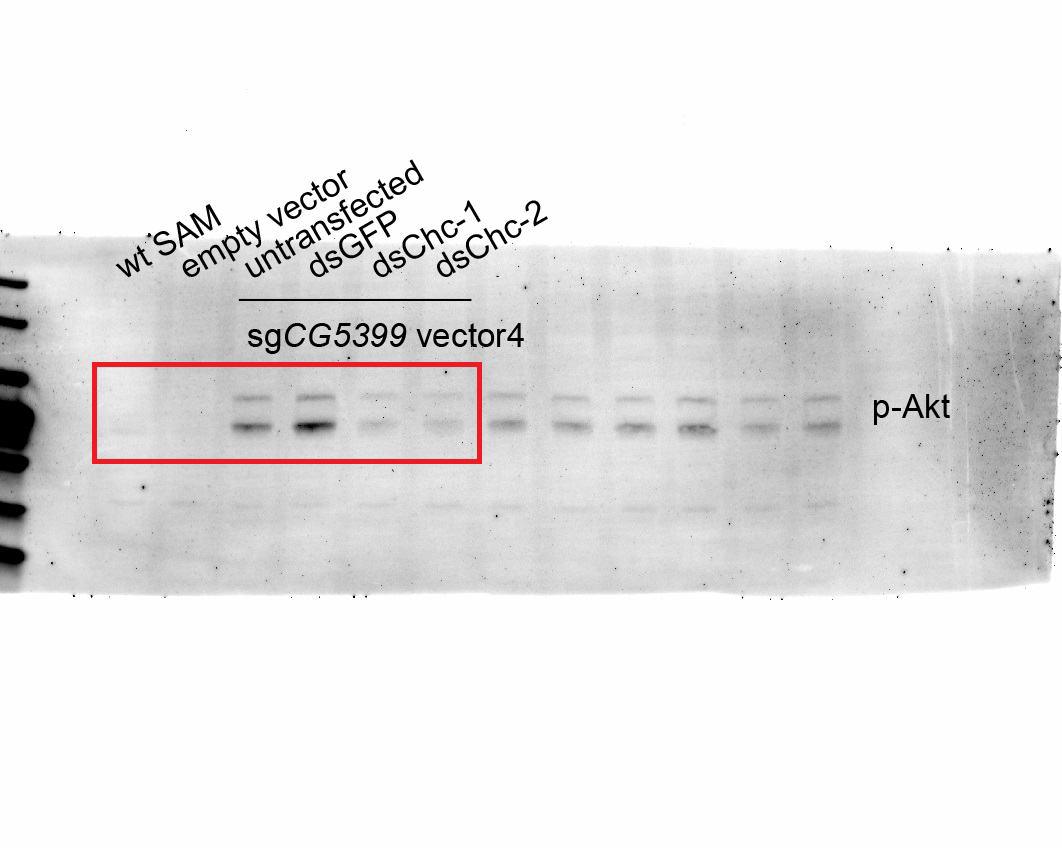

Supplement: Figure 4—source data 1. [file elife-85542-fig4-data1.zip › Figure 4 source data/Figure 4C/Figure 4C-pAkt dsChc.tif]

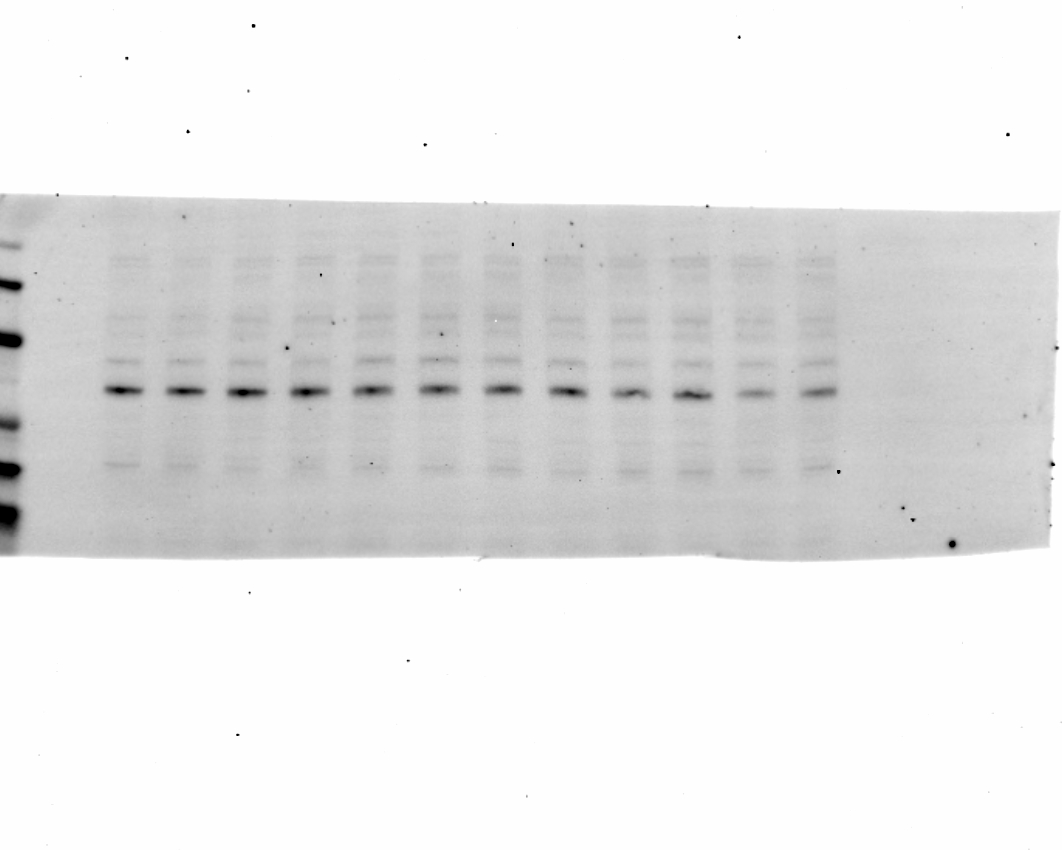

Supplement: Figure 4—source data 1. [file elife-85542-fig4-data1.zip › Figure 4 source data/Figure 4C/Figure 4C-total Akt for dsChc raw data.jpg]

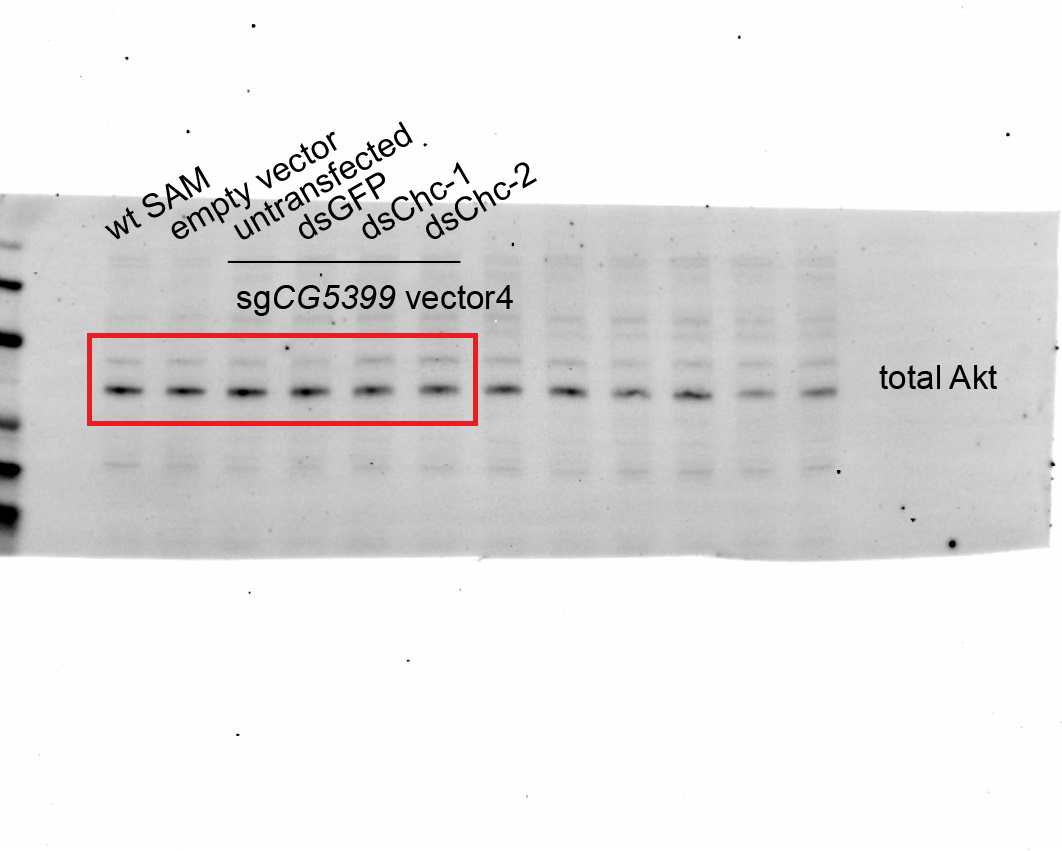

Supplement: Figure 4—source data 1. [file elife-85542-fig4-data1.zip › Figure 4 source data/Figure 4C/Figure 4C-total Akt for dsChc.tif]

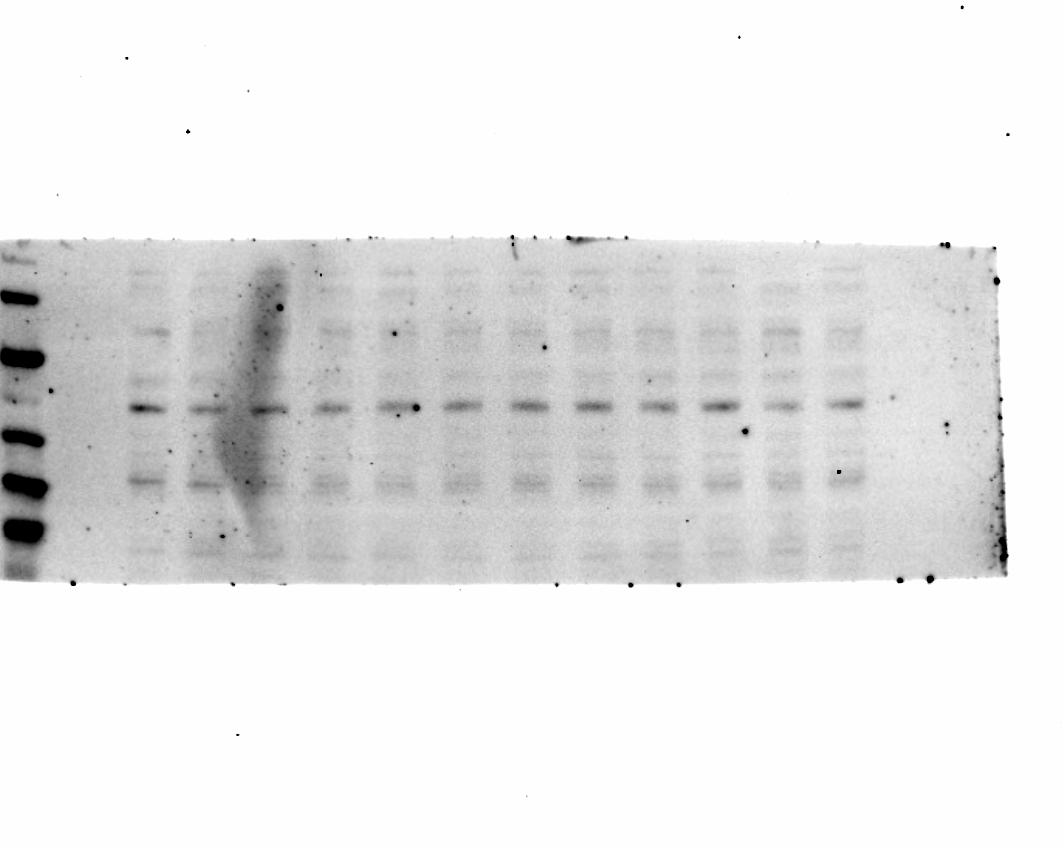

Supplement: Figure 4—source data 1. [file elife-85542-fig4-data1.zip › Figure 4 source data/Figure 4C/Figure 4C-total Akt for dsFlo raw data.jpg]

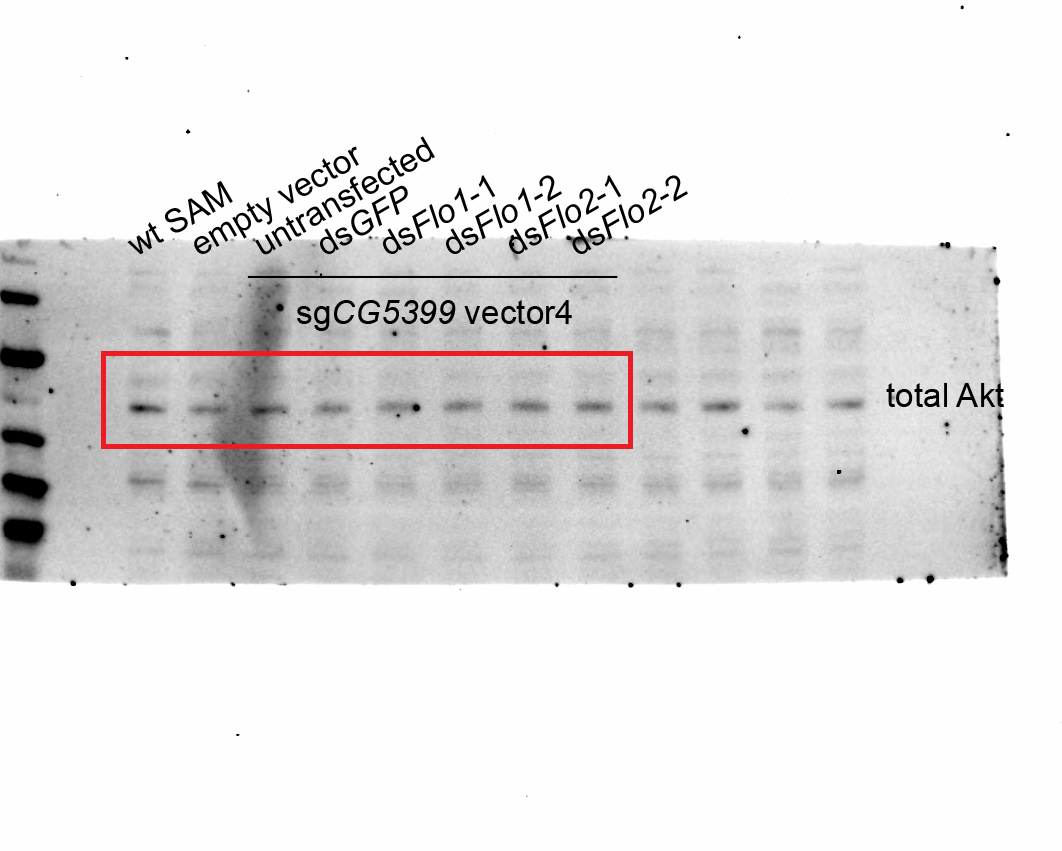

Supplement: Figure 4—source data 1. [file elife-85542-fig4-data1.zip › Figure 4 source data/Figure 4C/Figure 4C-total Akt for dsFlo.tif]

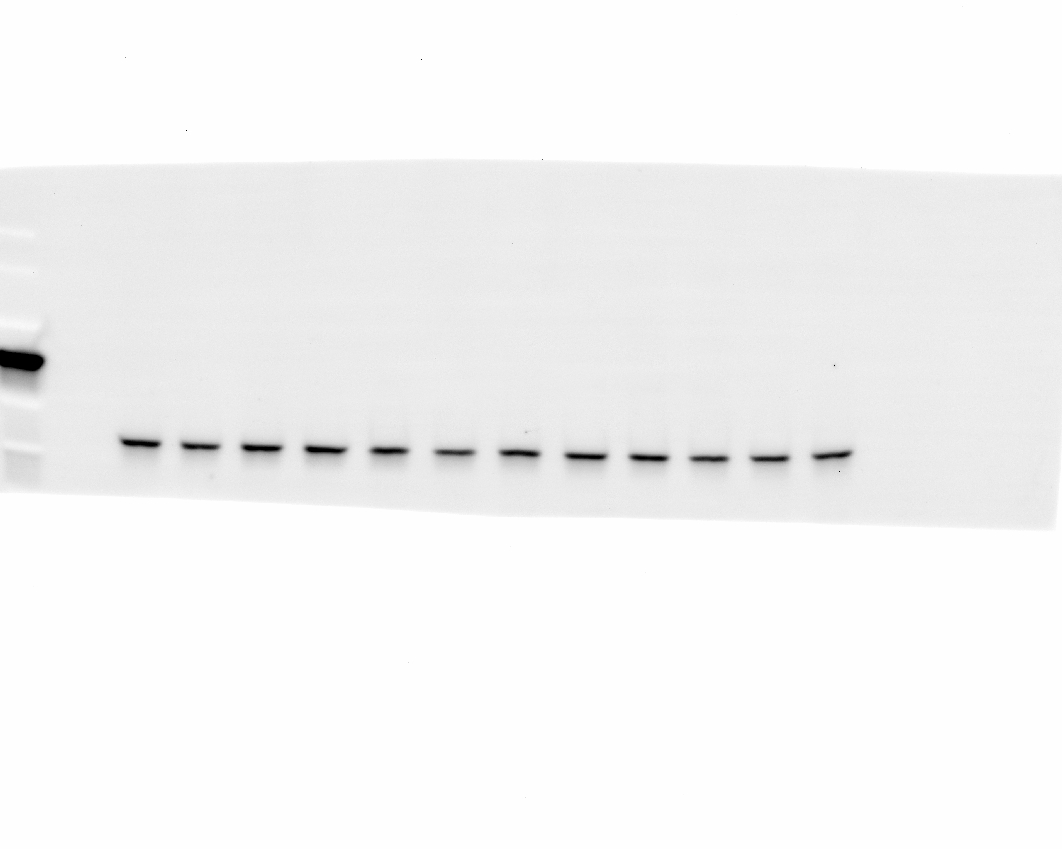

Supplement: Figure 4—source data 1. [file elife-85542-fig4-data1.zip › Figure 4 source data/Figure 4D/Figure 4D-actin raw data.jpg]

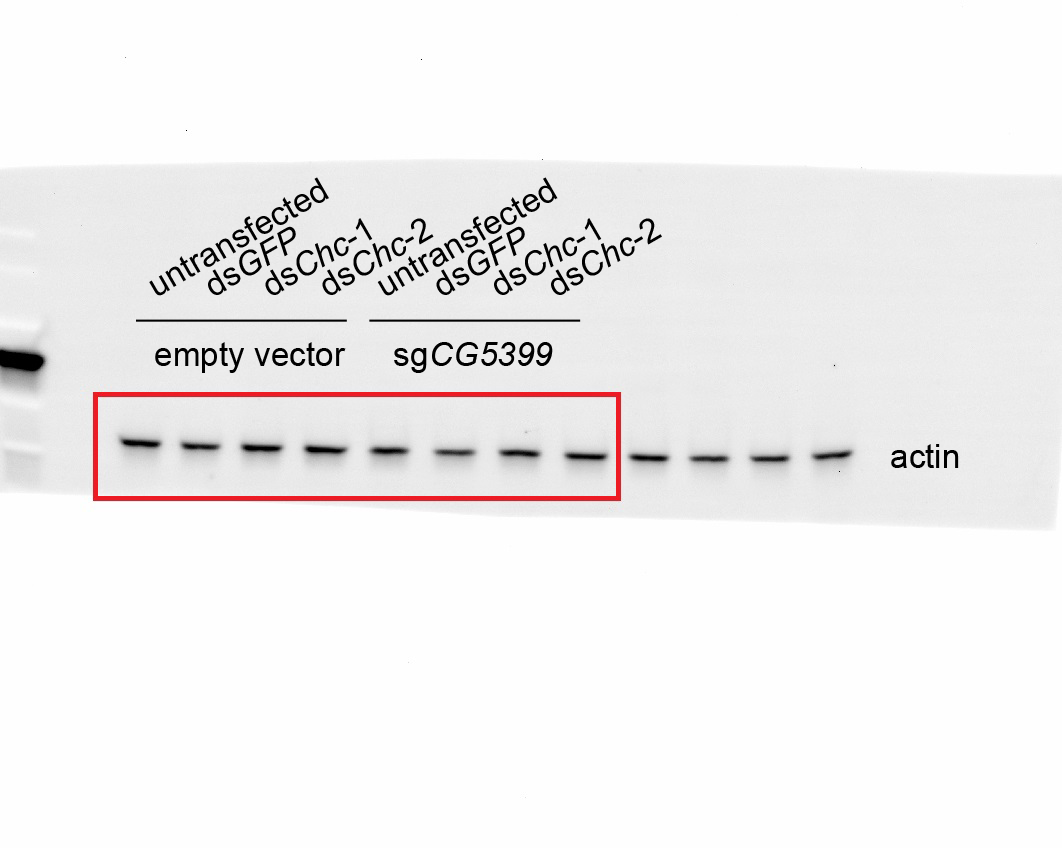

Supplement: Figure 4—source data 1. [file elife-85542-fig4-data1.zip › Figure 4 source data/Figure 4D/Figure 4D-actin.tif]

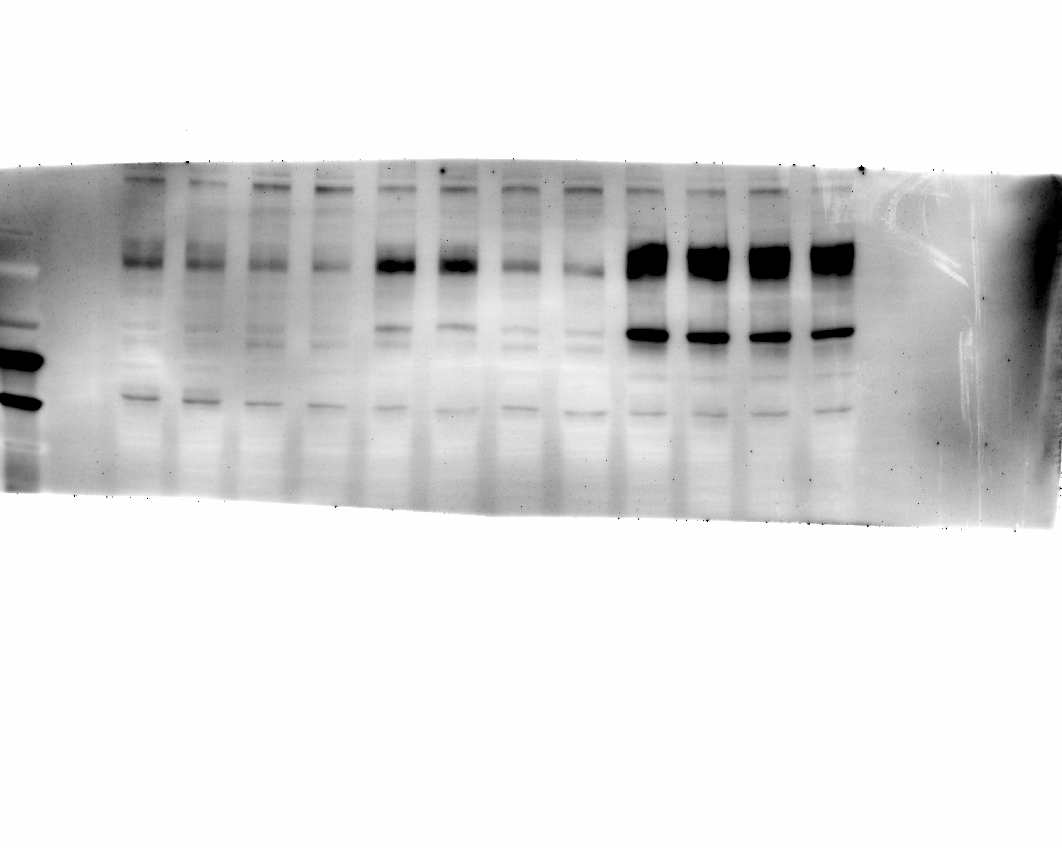

Supplement: Figure 4—source data 1. [file elife-85542-fig4-data1.zip › Figure 4 source data/Figure 4D/Figure 4D-pInR raw data.jpg]

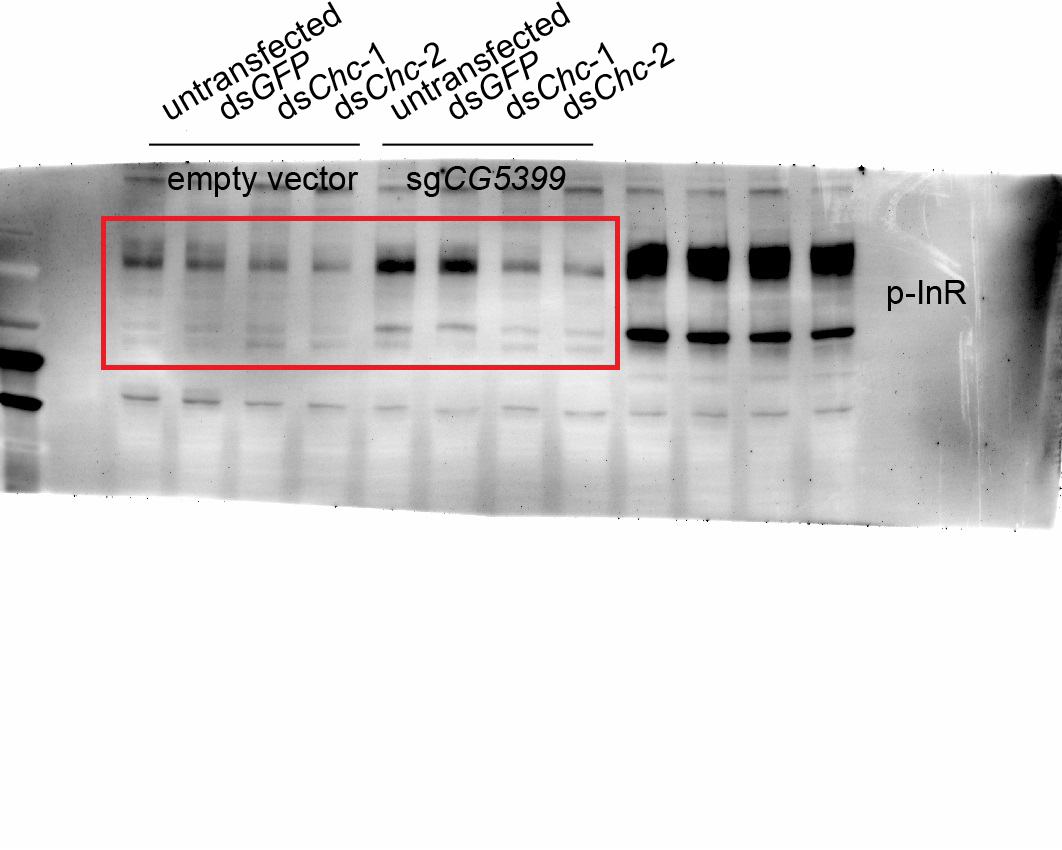

Supplement: Figure 4—source data 1. [file elife-85542-fig4-data1.zip › Figure 4 source data/Figure 4D/Figure 4D-pInR.tif]

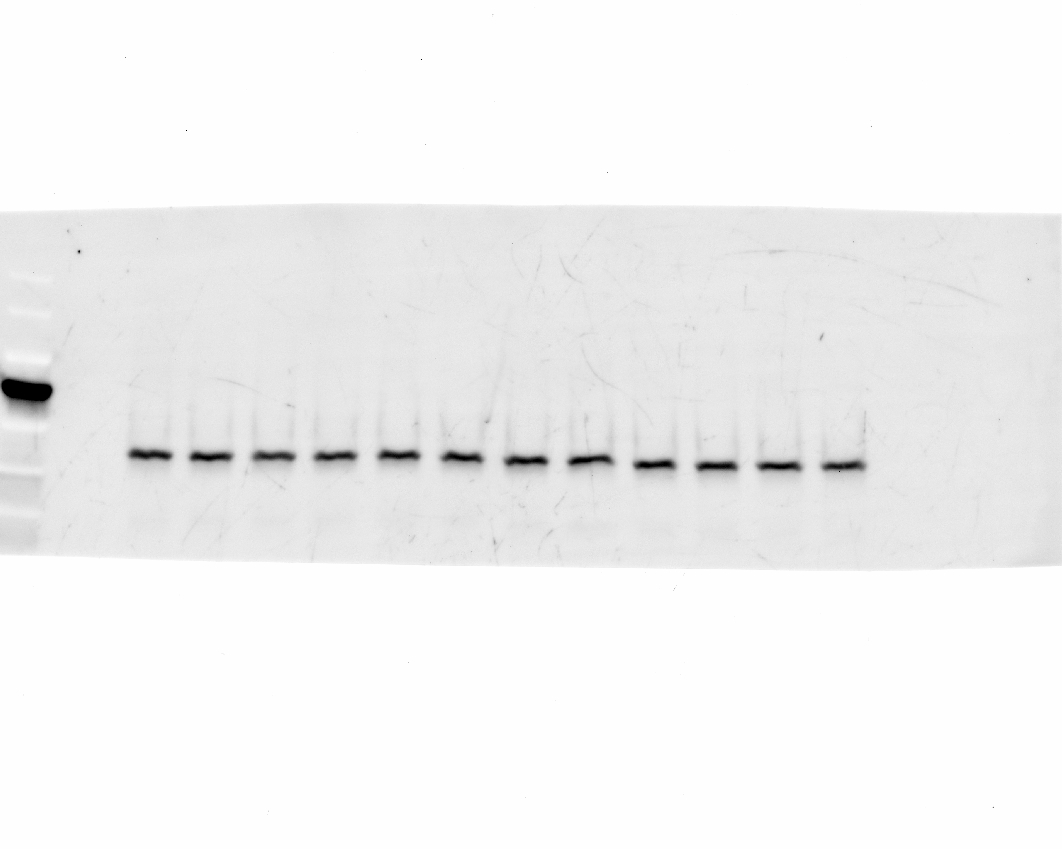

Supplement: Figure 4—source data 1. [file elife-85542-fig4-data1.zip › Figure 4 source data/Figure 4E/Figure 4E-actin raw data.jpg]

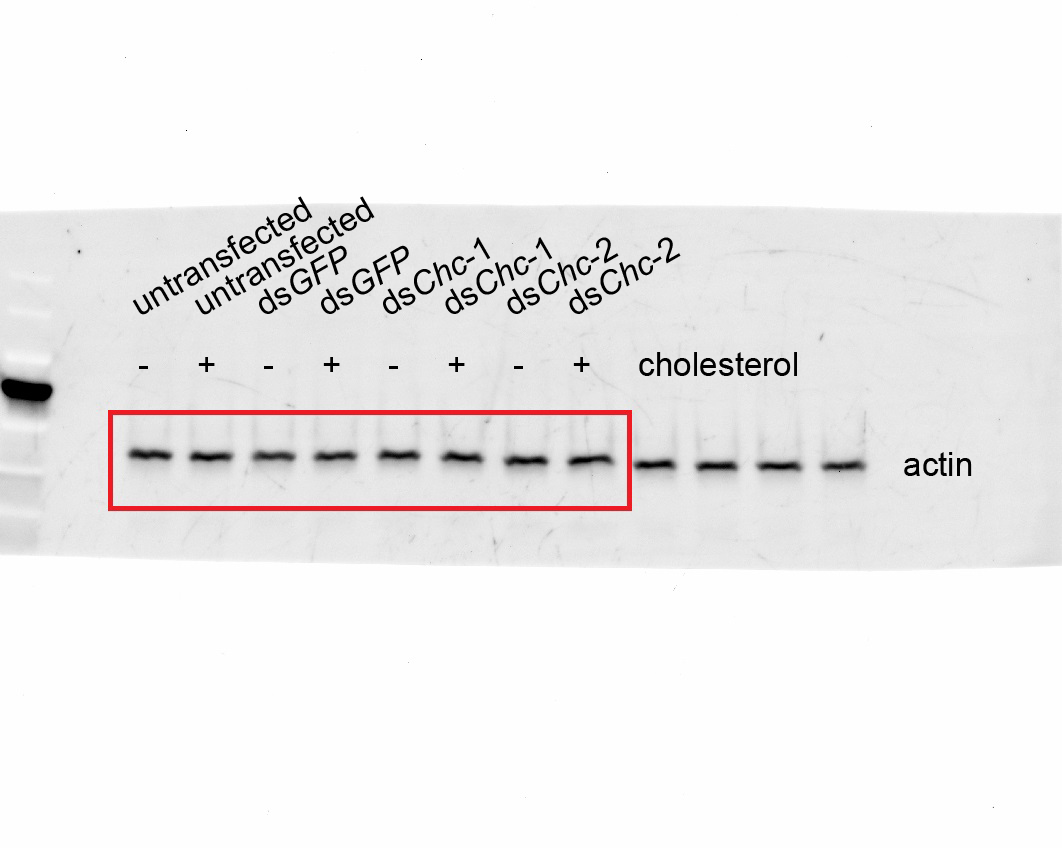

Supplement: Figure 4—source data 1. [file elife-85542-fig4-data1.zip › Figure 4 source data/Figure 4E/Figure 4E-actin.tif]

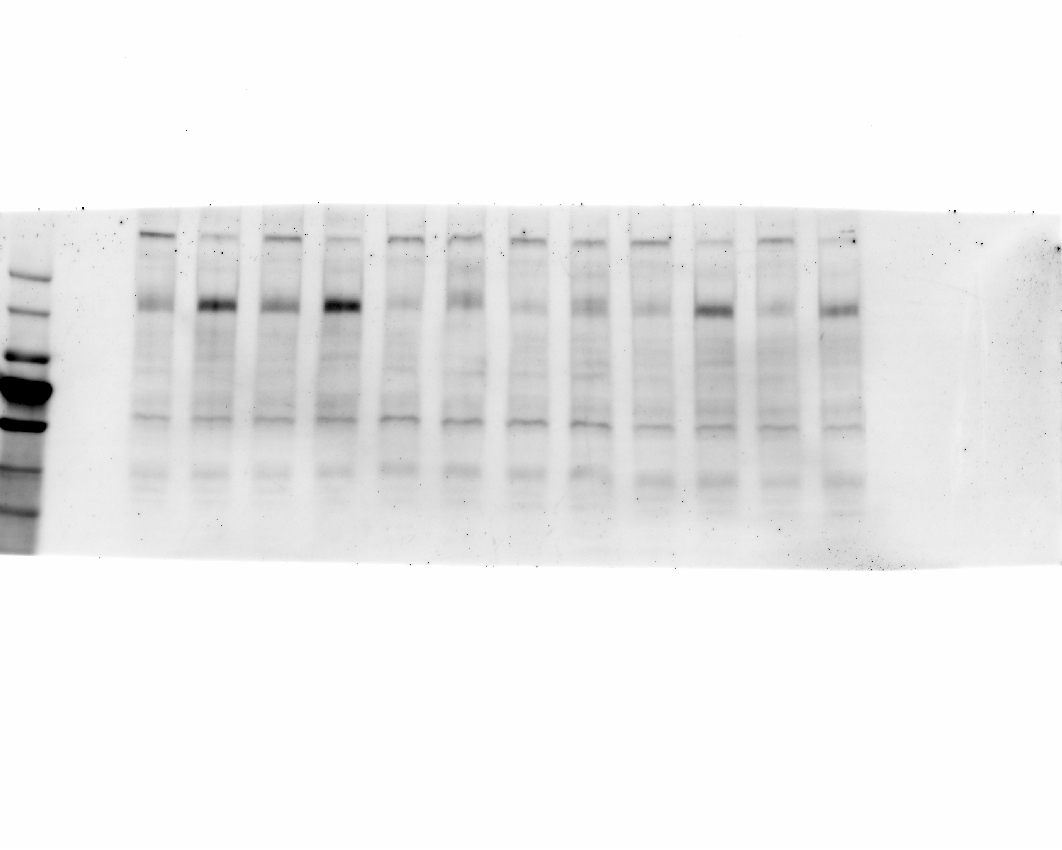

Supplement: Figure 4—source data 1. [file elife-85542-fig4-data1.zip › Figure 4 source data/Figure 4E/Figure 4E-pInR raw data.jpg]

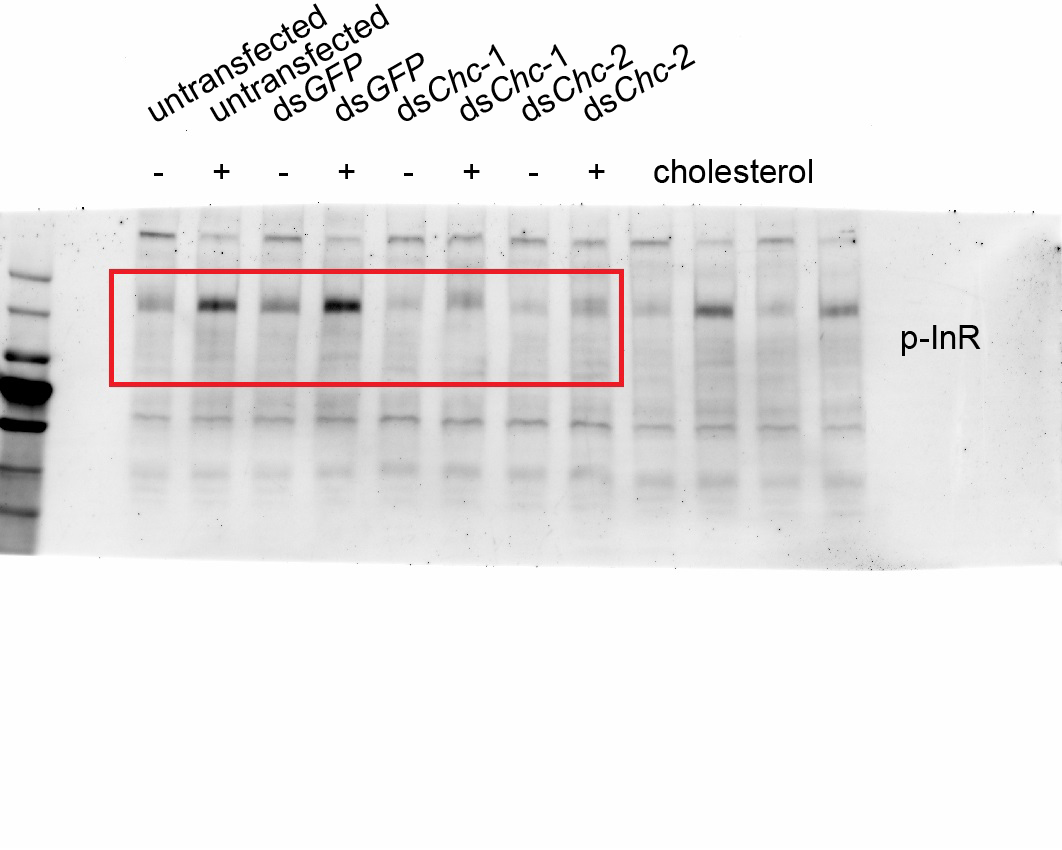

Supplement: Figure 4—source data 1. [file elife-85542-fig4-data1.zip › Figure 4 source data/Figure 4E/Figure 4E-pInR.tif]

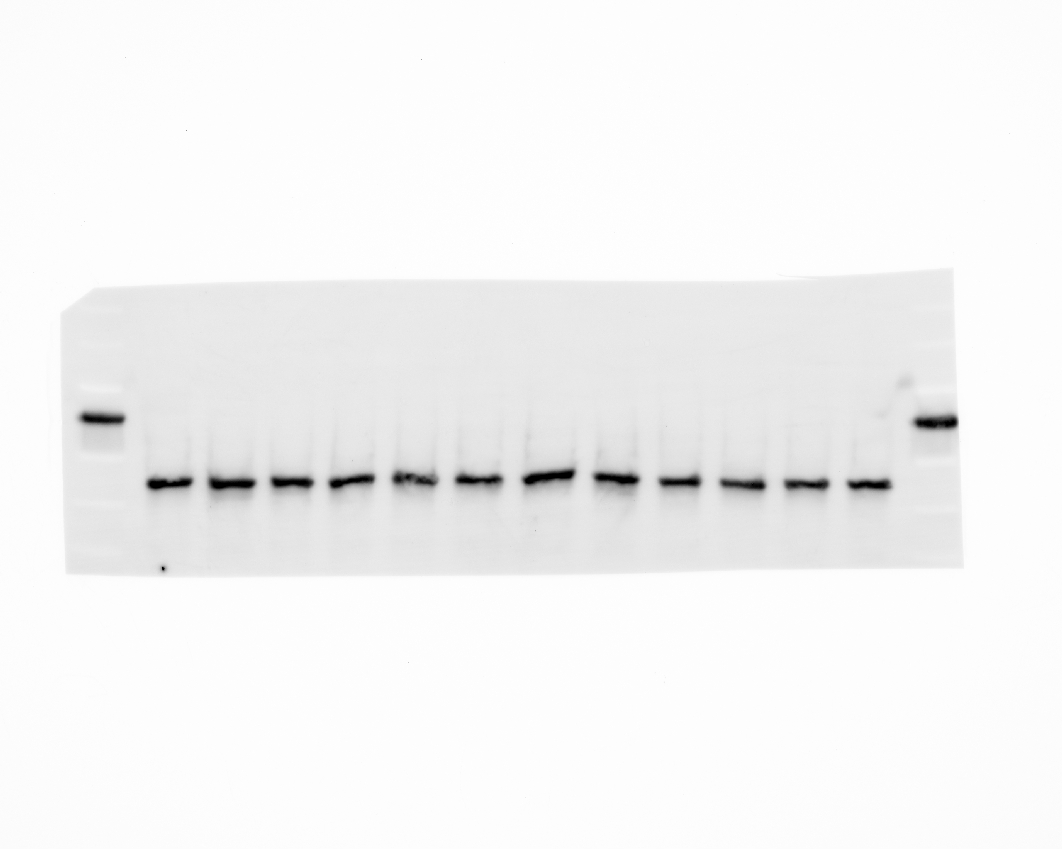

Supplement: Figure 4—figure supplement 1—source data 1. [file elife-85542-fig4-figsupp1-data1.zip › Figure 4-figure supplement 1 source data/Figure 4-figure supplement1C/S5C-actin raw data.jpg]

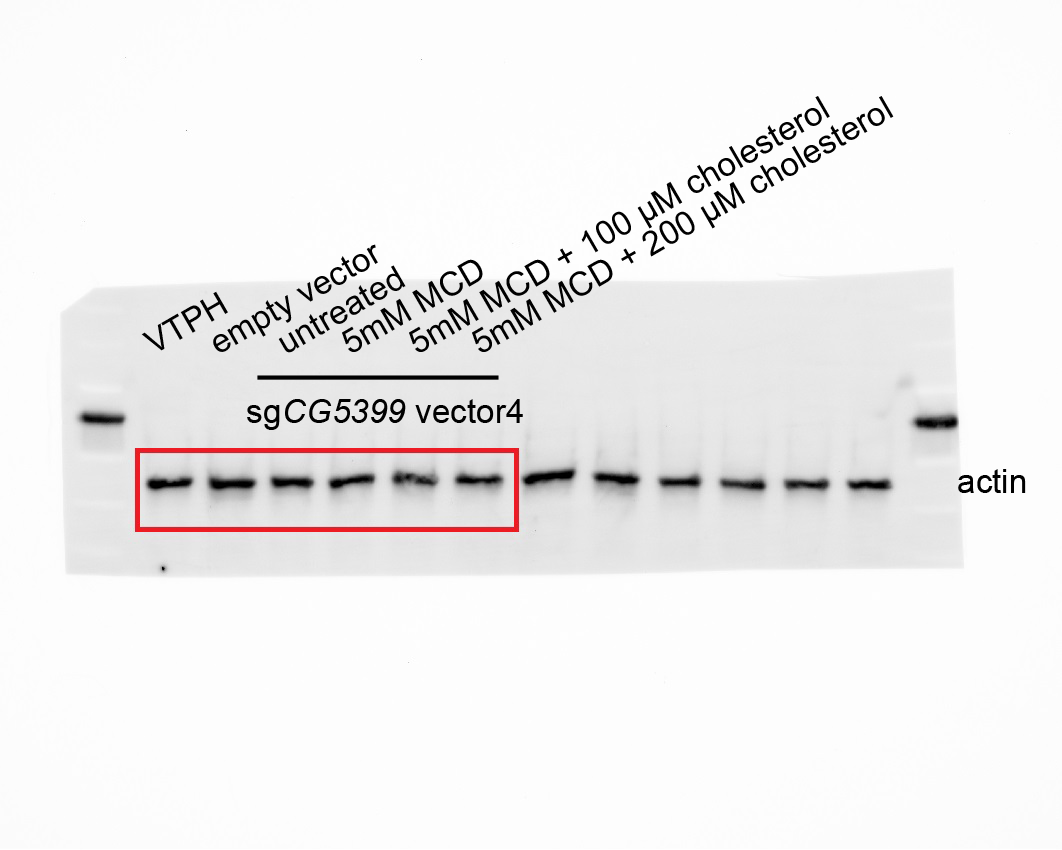

Supplement: Figure 4—figure supplement 1—source data 1. [file elife-85542-fig4-figsupp1-data1.zip › Figure 4-figure supplement 1 source data/Figure 4-figure supplement1C/S5C-actin.tif]

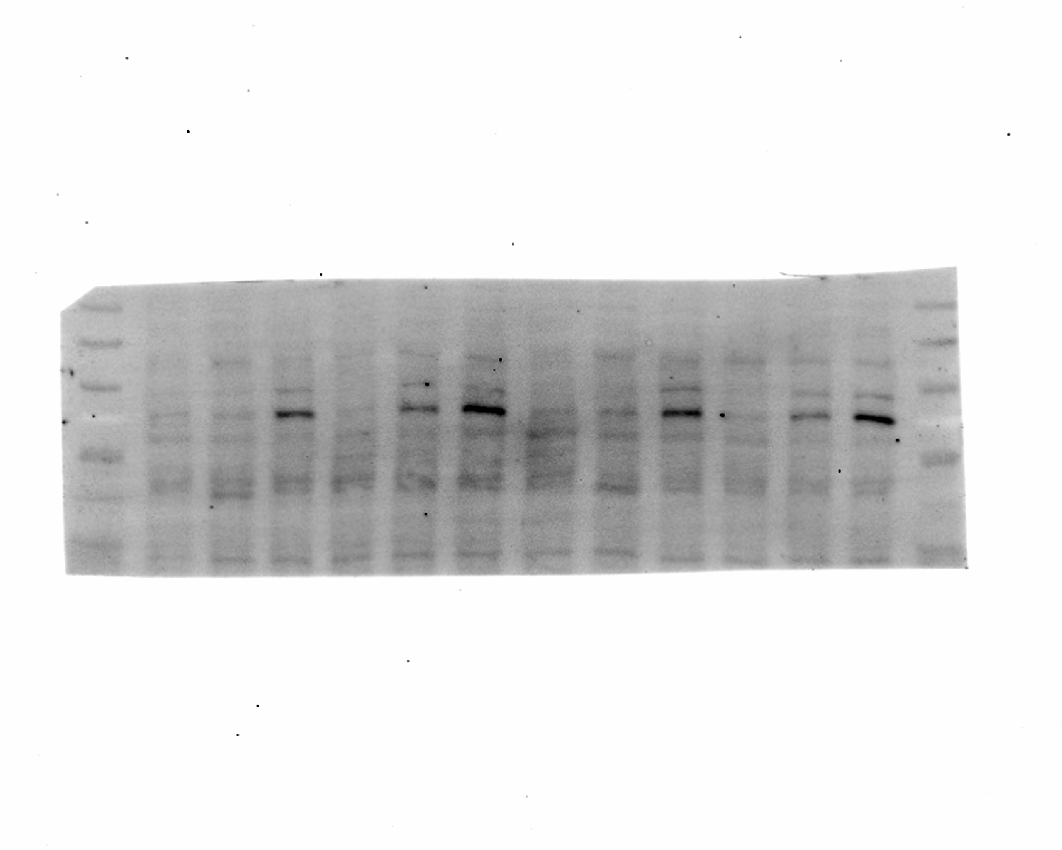

Supplement: Figure 4—figure supplement 1—source data 1. [file elife-85542-fig4-figsupp1-data1.zip › Figure 4-figure supplement 1 source data/Figure 4-figure supplement1C/S5C-pAkt raw data.jpg]

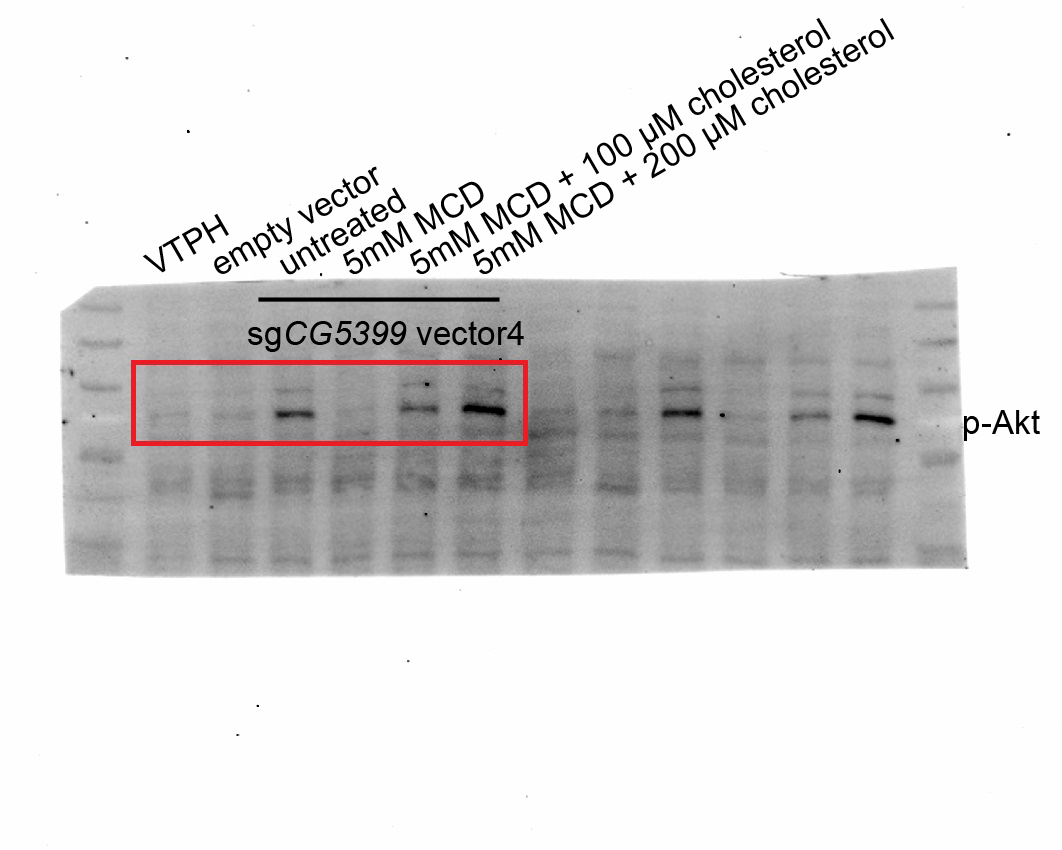

Supplement: Figure 4—figure supplement 1—source data 1. [file elife-85542-fig4-figsupp1-data1.zip › Figure 4-figure supplement 1 source data/Figure 4-figure supplement1C/S5C-pAkt.tif]

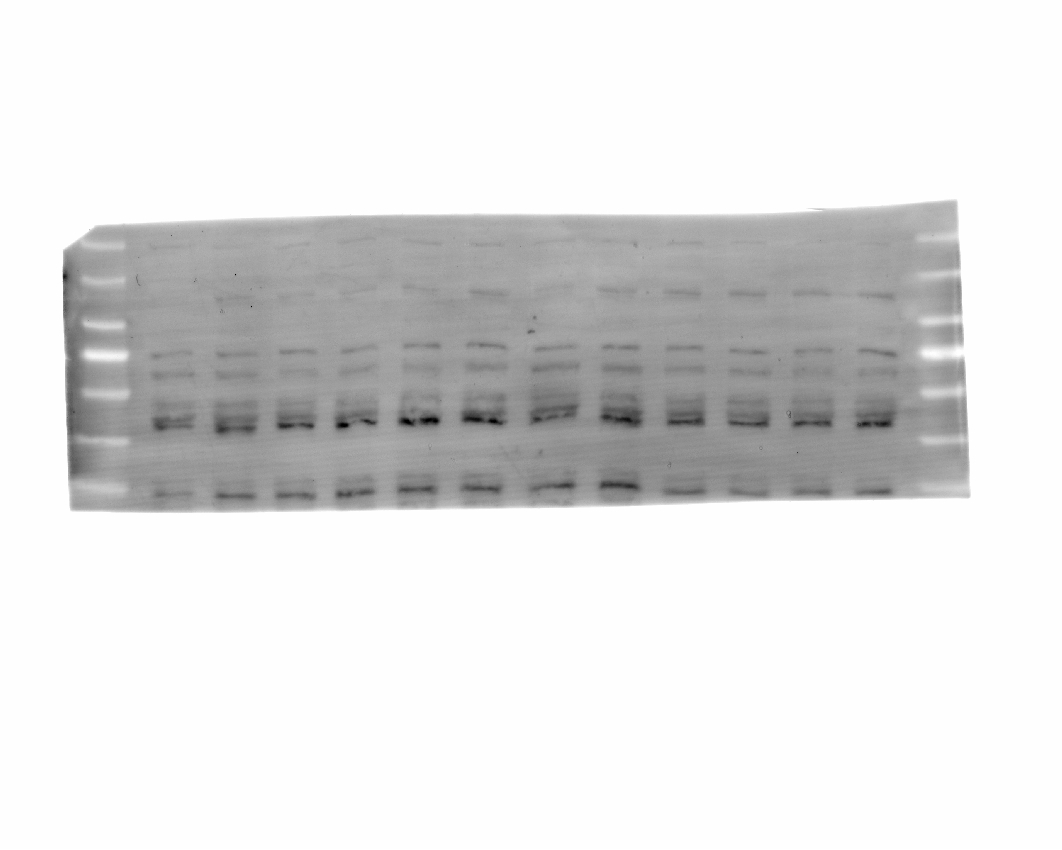

Supplement: Figure 4—figure supplement 1—source data 1. [file elife-85542-fig4-figsupp1-data1.zip › Figure 4-figure supplement 1 source data/Figure 4-figure supplement1C/S5C-total Akt raw data.jpg]

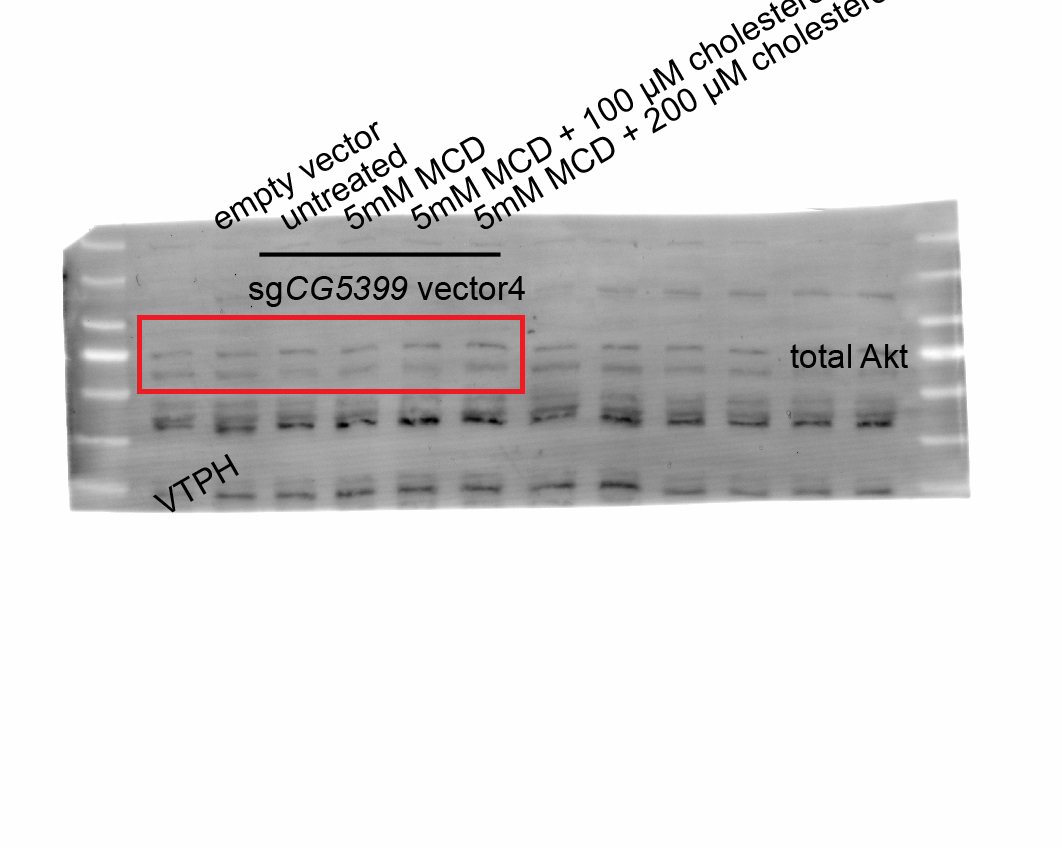

Supplement: Figure 4—figure supplement 1—source data 1. [file elife-85542-fig4-figsupp1-data1.zip › Figure 4-figure supplement 1 source data/Figure 4-figure supplement1C/S5C-total Akt.tif]

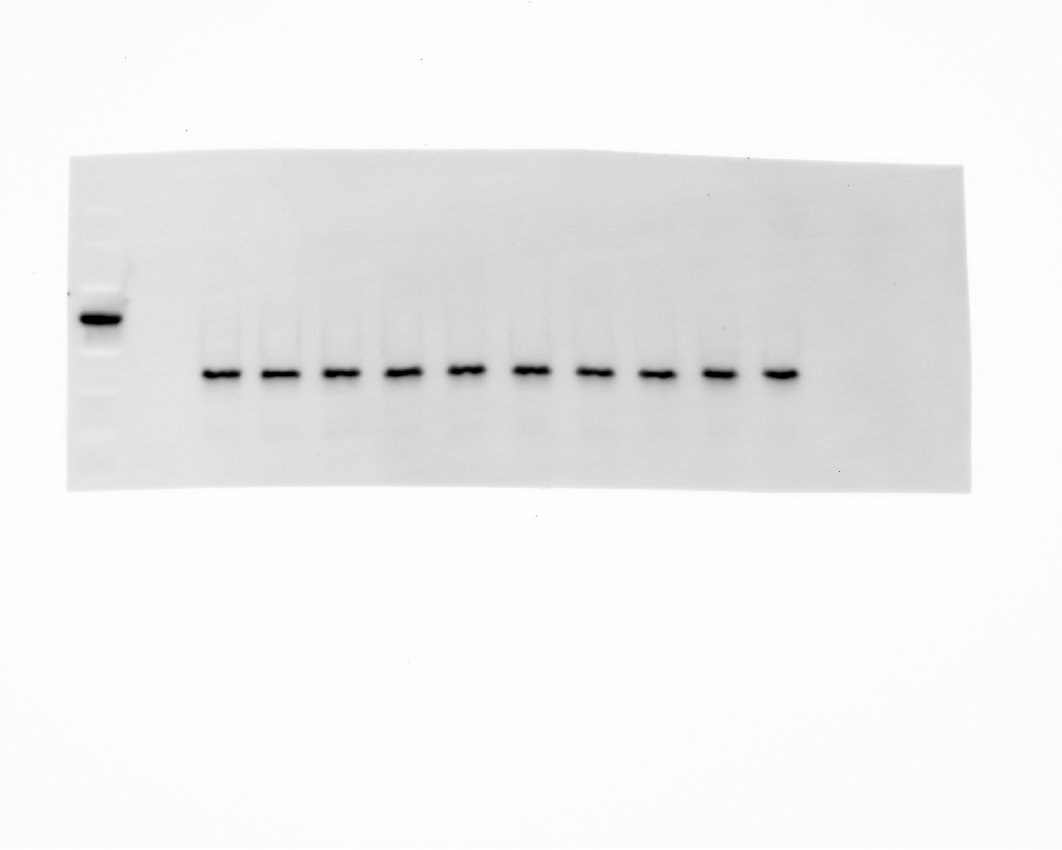

Supplement: Figure 4—figure supplement 1—source data 1. [file elife-85542-fig4-figsupp1-data1.zip › Figure 4-figure supplement 1 source data/Figure 4-figure supplement1D/S5D-actin for pAkt blot raw data.jpg]

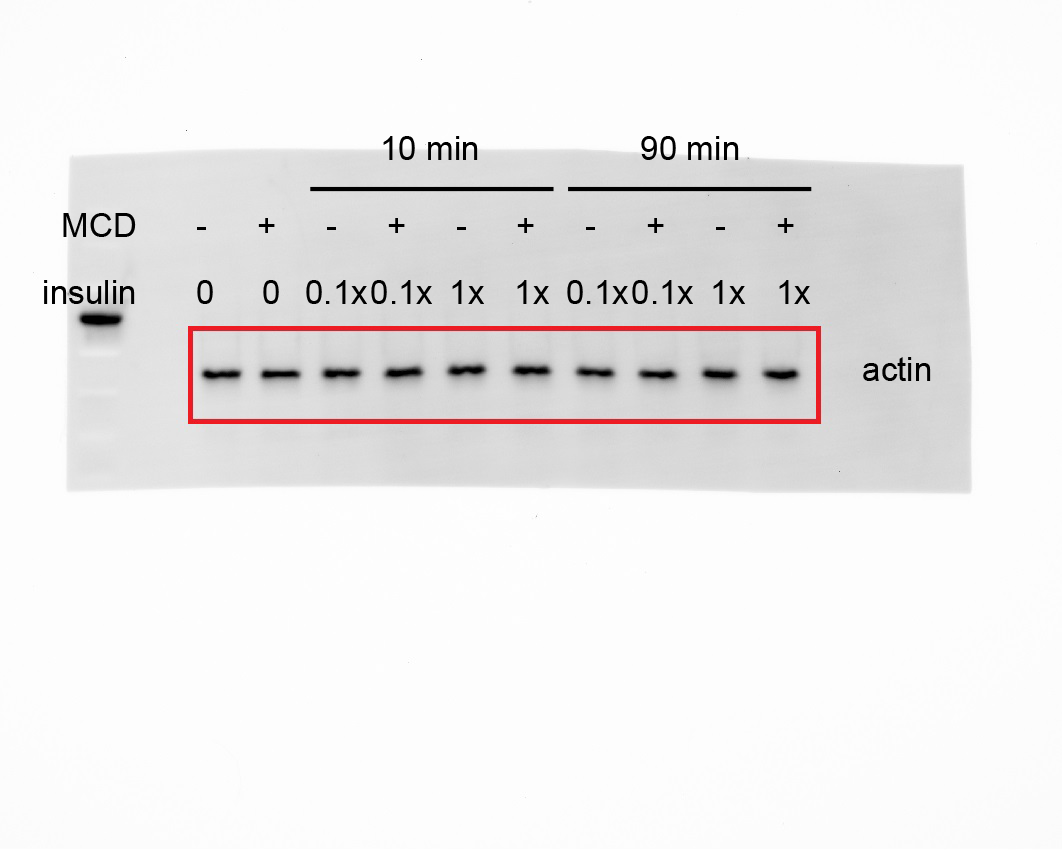

Supplement: Figure 4—figure supplement 1—source data 1. [file elife-85542-fig4-figsupp1-data1.zip › Figure 4-figure supplement 1 source data/Figure 4-figure supplement1D/S5D-actin for pAkt blot.tif]

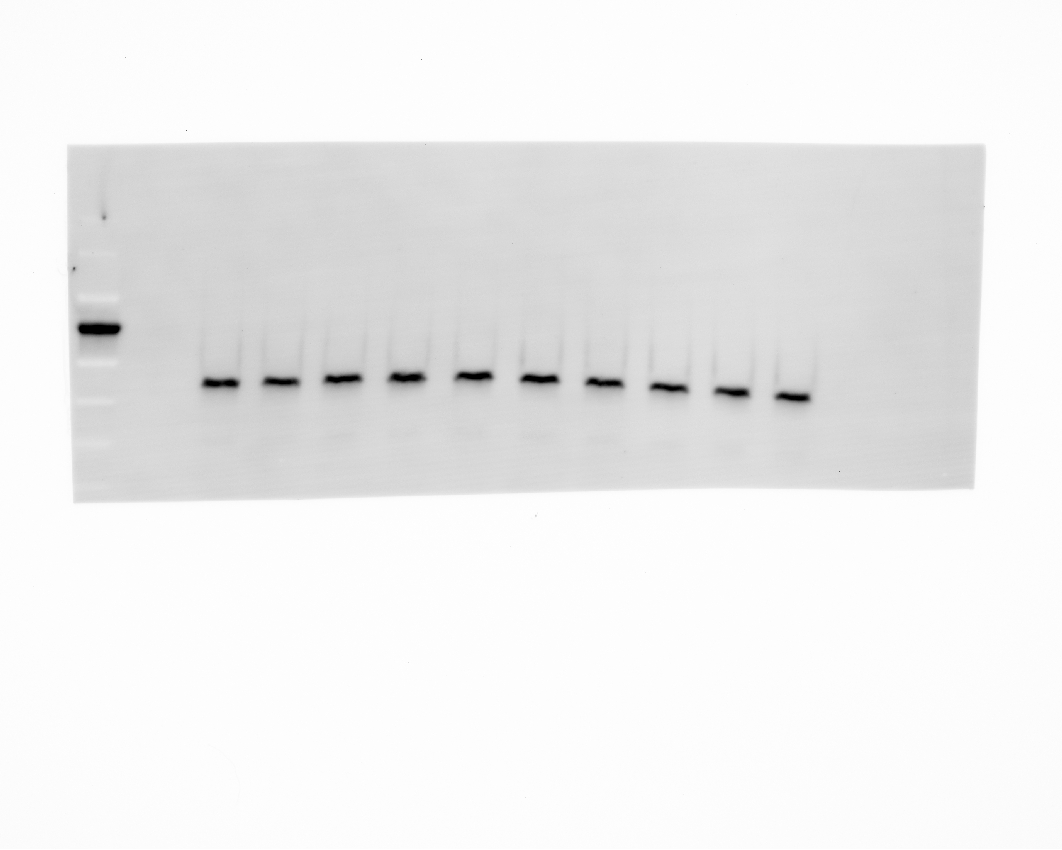

Supplement: Figure 4—figure supplement 1—source data 1. [file elife-85542-fig4-figsupp1-data1.zip › Figure 4-figure supplement 1 source data/Figure 4-figure supplement1D/S5D-actin for pInR blot raw data.jpg]

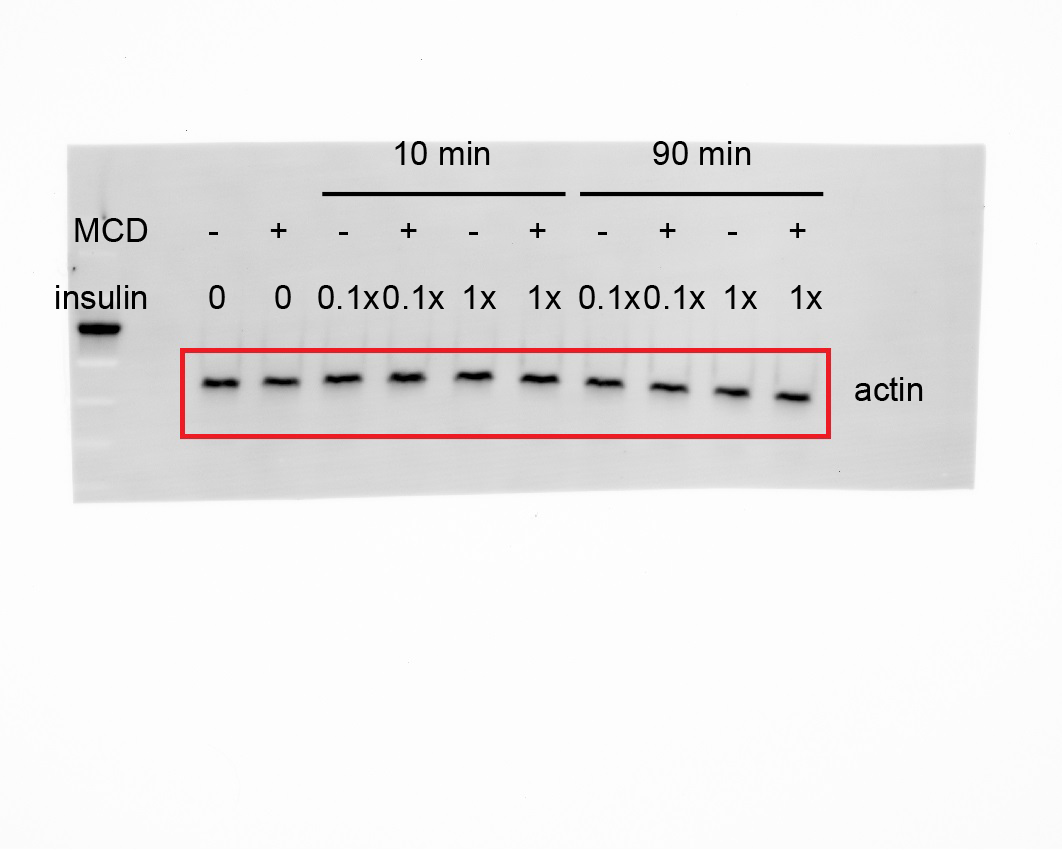

Supplement: Figure 4—figure supplement 1—source data 1. [file elife-85542-fig4-figsupp1-data1.zip › Figure 4-figure supplement 1 source data/Figure 4-figure supplement1D/S5D-actin for pInR blot.tif]

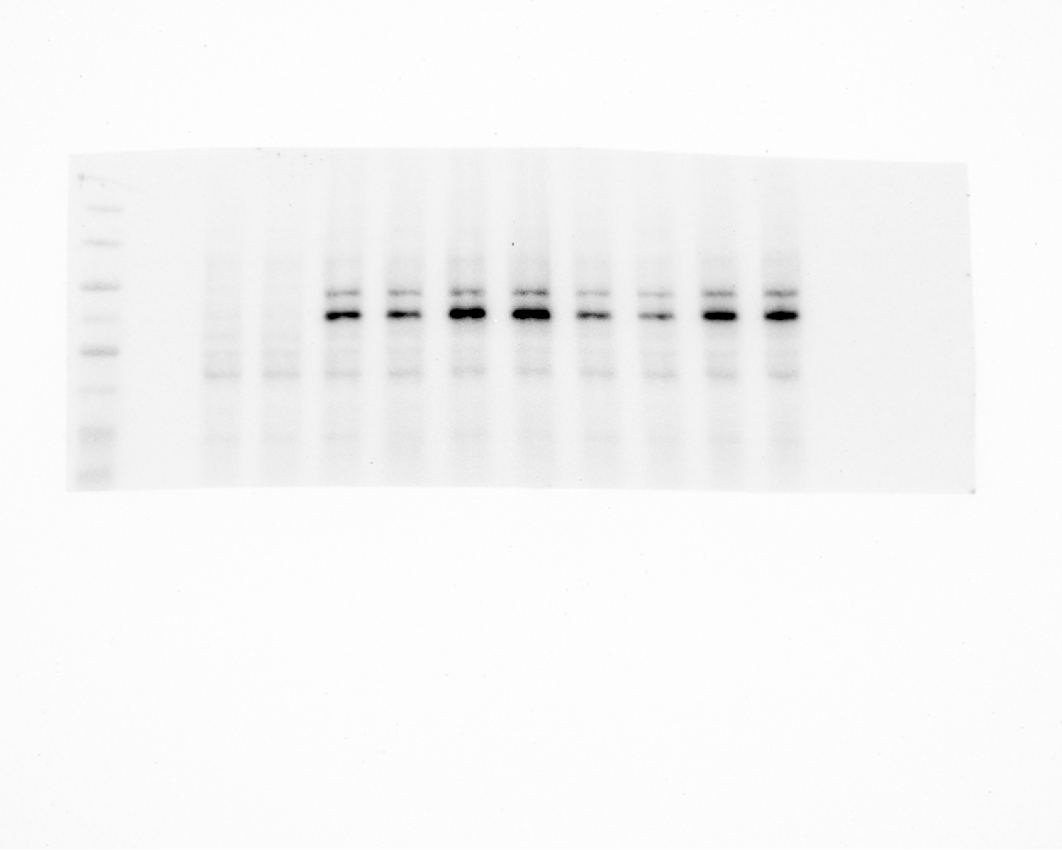

Supplement: Figure 4—figure supplement 1—source data 1. [file elife-85542-fig4-figsupp1-data1.zip › Figure 4-figure supplement 1 source data/Figure 4-figure supplement1D/S5D-pAkt raw data.jpg]

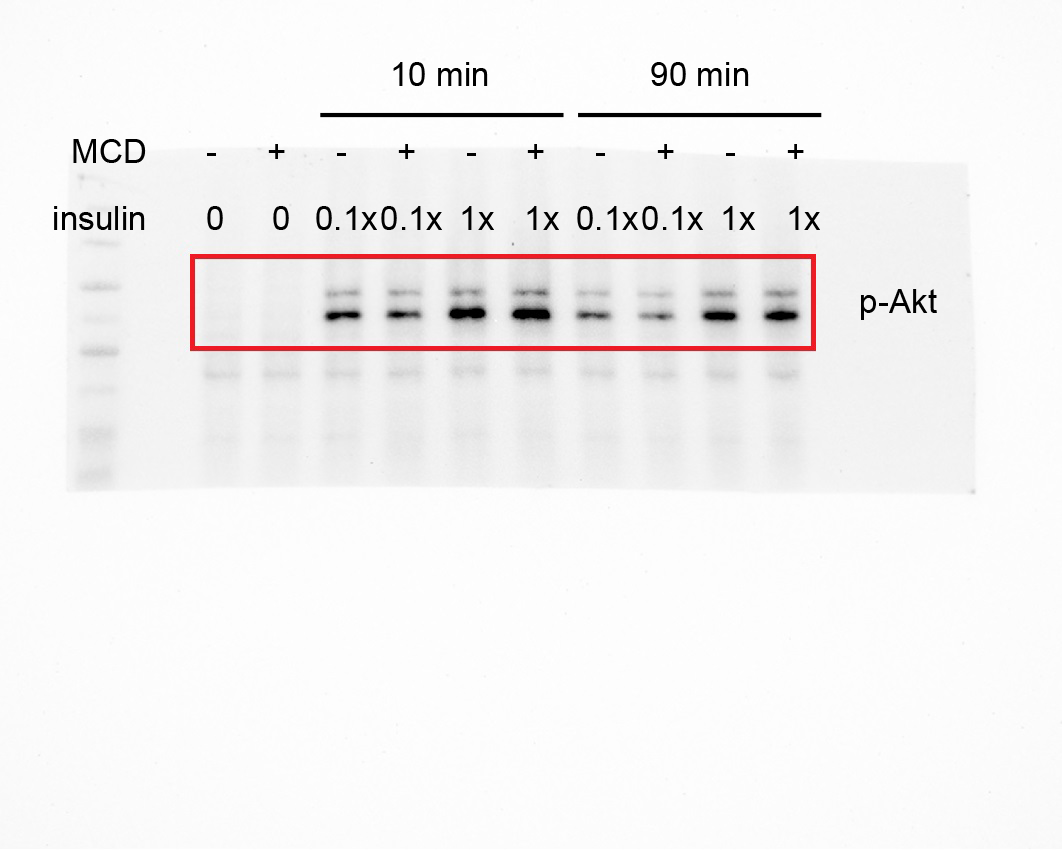

Supplement: Figure 4—figure supplement 1—source data 1. [file elife-85542-fig4-figsupp1-data1.zip › Figure 4-figure supplement 1 source data/Figure 4-figure supplement1D/S5D-pAkt.tif]

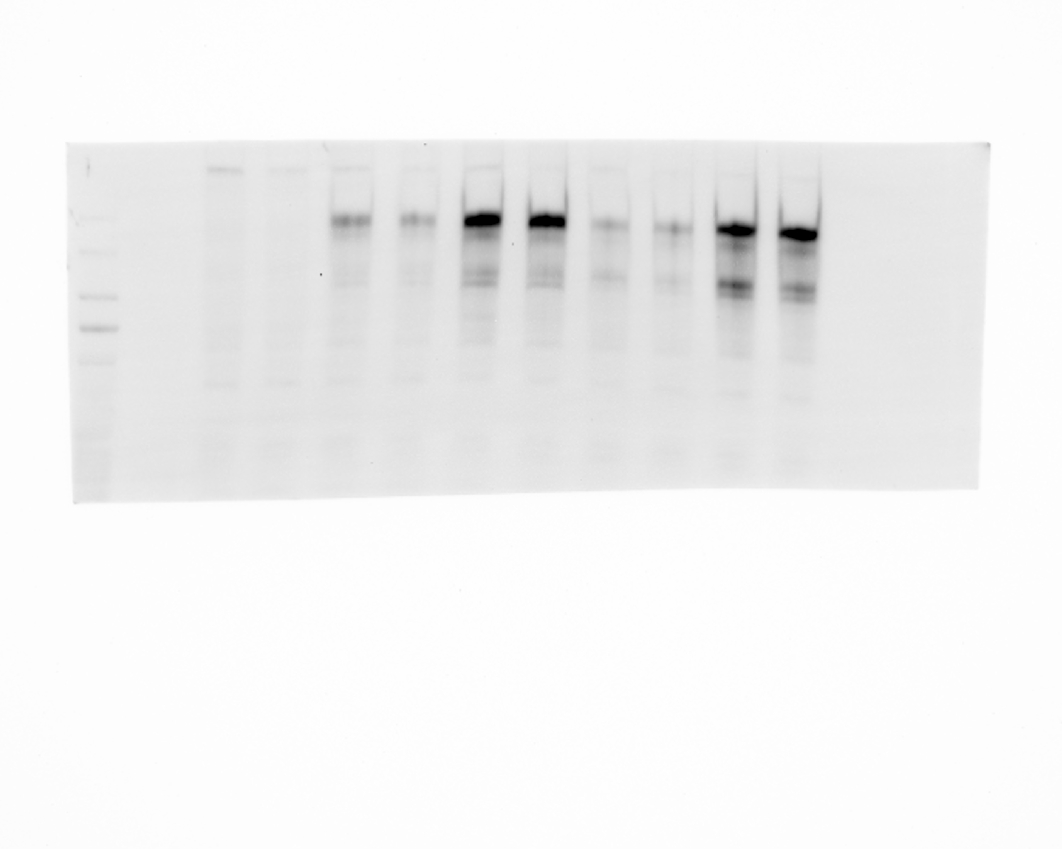

Supplement: Figure 4—figure supplement 1—source data 1. [file elife-85542-fig4-figsupp1-data1.zip › Figure 4-figure supplement 1 source data/Figure 4-figure supplement1D/S5D-pInR raw data.jpg]

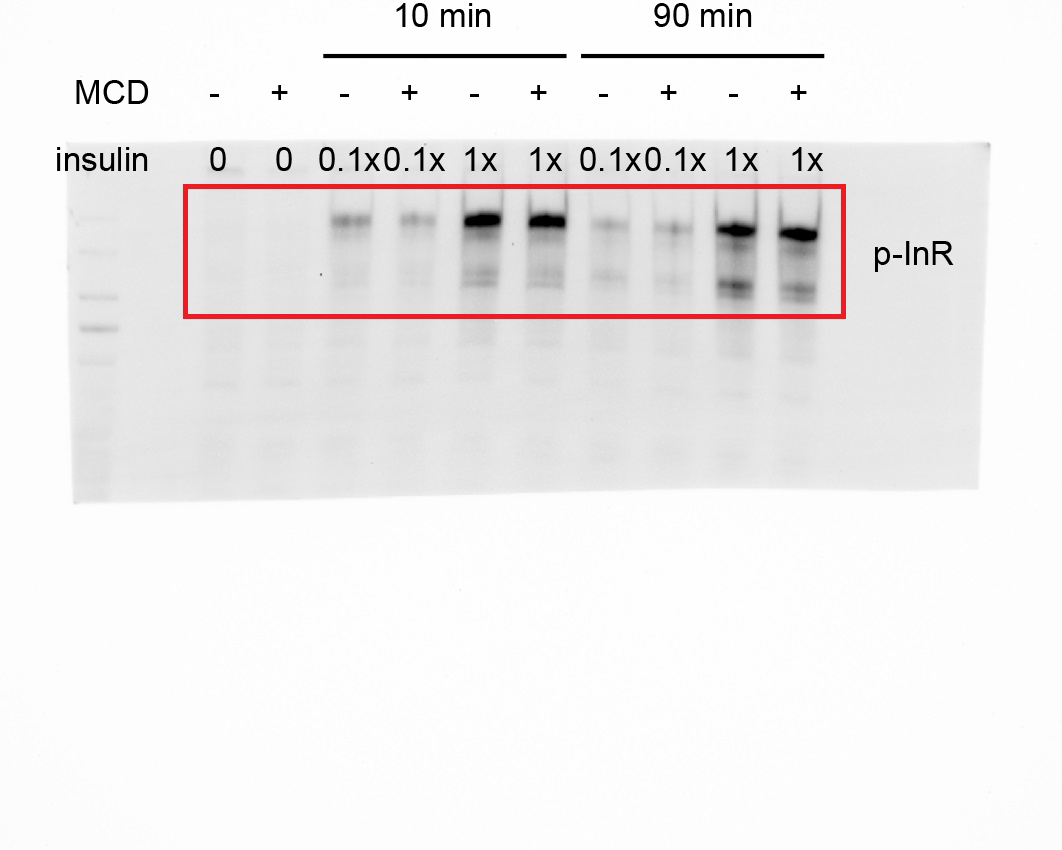

Supplement: Figure 4—figure supplement 1—source data 1. [file elife-85542-fig4-figsupp1-data1.zip › Figure 4-figure supplement 1 source data/Figure 4-figure supplement1D/S5D-pInR.tif]
