# Supplementary material for: Pooled genome-wide CRISPR activation screening for rapamycin resistance genes in Drosophila cells
Source: eLife. 2023 Apr 20;12:e85542. doi: 10.7554/eLife.85542 (PMC10118385; doi:10.7554/eLife.85542)
Supplement: Supplementary file 1. [file elife-85542-supp1.docx]

Supplementary file 1. sgRNA vectors used in this study.

| vector | description | 1^st^ sgRNA sequence | 2^nd^ sgRNA sequence |
| --- | --- | --- | --- |
| sg*Jon25Biii* | Single sgRNA vector | GAAATCAAAGAAACTCTAGG |  |
| sg*Sdr* | Single sgRNA vector | AGAATGAAAATCAAAGGTGG |  |
| sg*CG9877* | Dual-sgRNA vector | ATATTGTGTGGATATTGTAT | CTAATACGAACGCGATATTT |
| sg*CG8468* vector 1 | Dual-sgRNA vector | ACACTGCTAGAACGTGTTGT | TCCGTCGCTACTCCATAGTG |
| sg*CG8468* vector 2 | Dual-sgRNA vector | CGTATACGACACAGTGTTCT | ACACTGCTAGAACGTGTTGT |
| sg*CG8468* vector 3 | Dual-sgRNA vector | GATGAAGGTGCTATTTGGTA | CTTCGGATTTGTTGGTTACA |
| sg*CG8468* vector 4 | Dual-sgRNA vector | CTTCGGATTTGTTGGTTACA | CGTATACGACACAGTGTTCT |
| sg*CG8468* vector 5 | Dual-sgRNA vector | TCCGTCGCTACTCCATAGTG | TGTTGGCAGTATAAACCGCG |
| sg*CG8468* vector 6 | Dual-sgRNA vector | TGTTGGCAGTATAAACCGCG | GATGAAGGTGCTATTTGGTA |
| sg*CG5399* vector 1 | Dual-sgRNA vector | TTGCAGGTAAGACAACCTAT | AAAAGATACAAGCCTATAAA |
| sg*CG5399* vector 2 | Dual-sgRNA vector | TGGAATGACTAATCCCCACG | TGGACACCGAATCTATTTCT |
| sg*CG5399* vector 3 | Dual-sgRNA vector | GTTTGTTTATACCTTTCAAG | TGGAATGACTAATCCCCACG |
| sg*CG5399* vector 4 | Dual-sgRNA vector | TGGACACCGAATCTATTTCT | GCCAGGCGCTCCACAAGTGC |
| sg*CG5399* vector 5 | Dual-sgRNA vector | AAAAGATACAAGCCTATAAA | GTTTGTTTATACCTTTCAAG |
| sg*CG5399* vector 6 | Dual-sgRNA vector | GCCAGGCGCTCCACAAGTGC | TTGCAGGTAAGACAACCTAT |
| sg*CG9932* vector 1 | Dual-sgRNA vector | GCCGAAGTCGCACGAACAAC | GAGCTGTTGGGTGTGCGGTT |
| sg*CG9932* vector 2 | Dual-sgRNA vector | GAGCTGTTGGGTGTGCGGTT | CTTCTTCGCTCTCTTAAGCG |
| sg*CG9932* vector 3 | Dual-sgRNA vector | AACGGCCGGTGCTAACAGAG | GCCGAAGTCGCACGAACAAC |
| sg*CG9932* vector 4 | Dual-sgRNA vector | TGCCACCGACTGGTTGGCGT | AACGGCCGGTGCTAACAGAG |
| sg*CG9932* vector 5 | Dual-sgRNA vector | CTTCTTCGCTCTCTTAAGCG | AGCGCGAGAGCAAGCGAACG |
| sg*CG9932* vector 6 | Dual-sgRNA vector | AGCGCGAGAGCAAGCGAACG | TGCCACCGACTGGTTGGCGT |
