# Supplementary material for: Pooled genome-wide CRISPR activation screening for rapamycin resistance genes in Drosophila cells
Source: eLife. 2023 Apr 20;12:e85542. doi: 10.7554/eLife.85542 (PMC10118385; doi:10.7554/eLife.85542)
Supplement: Supplementary file 2. [file elife-85542-supp2.docx]

Supplementary file 2. PCR primers used in this study.

| primer | description | Sequence (5'-3') |
| --- | --- | --- |
| Focused-NGS-1F | 1^st^ round PCR of NGS library for focused screen | CCTATTTTCAATTTAACGTCG |
| Focused-NGS-1R | 1^st^ round PCR of NGS library for focused screen | CTAGCCTTATTTTAACTTGC |
| Focused-NGS-2F | 2^nd^ round PCR of NGS library for focused screen | CCTACACGACGCTCTTCCGATCT-(N)_n_-(B)_6_-CCTATTTTCAATTTAACGTCG |
| Focused-NGS-2R | 2^nd^ round PCR of NGS library for focused screen | CTAGCCTTATTTTAACTTGC |
| Focused-NGS-3F | 3^rd^ round PCR of NGS library for focused screen | AATGATACGGCGACCACCGAGATCTACACTCTTTCCCTACACGACGCTCTTCCGATCT |
| Focused-NGS-3R | 3^rd^ round PCR of NGS library for focused screen | CAAGCAGAAGACGGCATACGAGATCTAGCCTTATTTTAACTTGC |
| WGS-NGS-1F | 1^st^ round PCR of NGS library for genome-wide screen | CCTATTTTCAATTTAACGTCG |
| WGS-NGS-1R | 1^st^ round PCR of NGS library for genome-wide screen | ATATGCTTTATTGACAGAAAATTTGATG |
| WGS-NGS-2F | 2^nd^ round PCR of NGS library for genome-wide screen | CCTACACGACGCTCTTCCGATCT-(N)_n_-(B)_6_-CCTATTTTCAATTTAACGTCG |
| WGS-NGS-2R | 2^nd^ round PCR of NGS library for genome-wide screen | ATATGCTTTATTGACAGAAAATTTGATG |
| WGS-NGS-3F | 3^rd^ round PCR of NGS library for genome-wide screen | AATGATACGGCGACCACCGAGATCTACACTCTTTCCCTACACGACGCTCTTCCGATCT |
| WGS-NGS-3R | 3^rd^ round PCR of NGS library for genome-wide screen | CAAGCAGAAGACGGCATACGAGATATATGCTTTATTGACAGAAAATTTGATG |
| q*Jon25Biii*-F | qPCR primer for *Jon25Biii* | CAAGCTGGTGGGAGTTAGCA |
| q*Jon25Biii*-R | qPCR primer for *Jon25Biii* | GGTCACGGATCCAGTCCAAG |
| q*Sdr*-F | qPCR primer for *Sdr* | CGGCTACTTCCAGACGCTAC |
| q*Sdr*-F | qPCR primer for *Sdr* | TGGCCACCAGTGAAGAAGA |
| q*CG9877*-F | qPCR primer for *CG9877* | GCGAGCTGCTATCGTGTTTG |
| q*CG9877*-R | qPCR primer for *CG9877* | CCTCCGAAGCCACCATATCC |
| q*CG13538*-F | qPCR primer for *CG13538* | GTTGGCCGATGAATTTGAAACG |
| q*CG13538*-R | qPCR primer for *CG13538* | ATCAGCCGATTGCGATACTTC |
| q*CG8468*-F | qPCR primer for *CG8468* | CTGATGGCAGAGTTCGGTGTG |
| q*CG8468*-R | qPCR primer for *CG8468* | CATGGCACTAACAAAGGGTCC |
| q*CG5399*-F | qPCR primer for *CG5399* | AGTGCGATCATCTGCCTGG |
| q*CG5399*-R | qPCR primer for *CG5399* | TTGTGGCGTTGTTTGGGCT |
| q*CG9932*-F | qPCR primer for *CG9932* | TTGCCGTTGTGAACAGCGT |
| q*CG9932*-R | qPCR primer for *CG9932* | CGCTTGATCTTGCATTGCGG |
