# Supplementary material for: Pooled genome-wide CRISPR activation screening for rapamycin resistance genes in Drosophila cells
Source: eLife. 2023 Apr 20;12:e85542. doi: 10.7554/eLife.85542 (PMC10118385; doi:10.7554/eLife.85542)
Supplement: Supplementary file 3. [file elife-85542-supp3.docx]

Supplementary file 3. dsRNAs used in this study.

| dsRNA | dsRNA sequence |
| --- | --- |
| ds*GFP* | AAGTTCATCTGCACCACCGGCAAGCTGCCCGTGCCCTGGCCCACCCTCGTGACCACCCTGACCTACGGCGTGCAGTGCTTCAGCCGCTACCCCGACCACATGAAGCAGCACGACTTCTTCAAGTCCGCCATGCCCGAAGGCTACGTCCAGGAGCGCACCATCTTCTTCAAGGGCGACGGCAACTACAAGACCCGCGCCGAGGTGAAGTTCGAGGGCGACACCCTGGTGAACCGCATCGAGCTGAAGGGCATCGACTTCAAGGAGGACGGCAACATCCTGGGGCACAAGCTGGAGTACAACTACAACAGCCACAACGTCTATATCATGGCCGACAAGCAGAAGAACGGCATCAAGGTGAACTTCAAGATCCGCCACAACATCGAGGACGGCAGCGTGCAGCTCGCCGACCACTACCAGCAGAACACCCCCATCGGCGACGGCCCCGTGCTGCTGCCCGACAACCACTACCTGAGCACCCAGTCCGCCCTGAGCAAAGA |
| ds*CG5399*-1 | ATGTGCAATAGTGCGATCATCTGCCTGGCAATCGTGGCCGCCGTGGCCTCCATCTCTCAGGTTTCGGCCCAAGCCCAAACAACGCCACAATCCTGCGCCCAAGCCACCGGCATTAGTTTTAACCAGACCGCT |
| ds*CG5399*-2 | TTTAGTGGTCAGTGGGTCGAGTTGGCCCGCAATCCCGCCTCAAATGTCTCCTGCATTTCCCTTAACGTGGCTTTCTGGAACAACAACAATTACATGCTGGTCAATGTCAGCCATTCAAGCAATGTCGCCTCCACTTTGGTGGACGTCTATGAAACTGCGAATATCACGCTGGTGGAGAACAACCTCAGCGGATACAATGTCACCCTGGTGAATGGCCAGAAGCAGGAGTACGTCTTCGTGAAGTTGCTTAGCTTTGTGAACTCCACCTATCTGGTTGGATGCACCTACACCAATGCTACCAACACATCAACCAGTGCTGGTTTTATTCTGGGTAGGTCCACCTATACTCCGGAAGGCGTAAGGATTGCCAACAATGATGCCTCCGTGTCCTTCAGCAATTTCCAGAACAACACCTACGGCAATGTCACTCAGTTGGA |
| ds*Pi3K92E*-1 | CCTACCGCGGATGACACGCCTCTGCATTGTGATCTTCGAGGTGACCAAGATGTCGCGCTCGAAGAAATCGTCCAACAATAAGGACATCGCTCTGAAAGACGTCCCGTACAACAAAAACCCGCTGGCCTGGGTCAACACAACAATTTTCGACCACAAGGACATCCTGCGCACCGGTCGCCATACGCTGTACACGTGGACGTATGCTGACGACATTCAGTCGGTTGAGGTTTTCCATCCGCTGGGCACCATCGAACCCAATCCGCGCAAAGAGGAATGTGCGCTGGTGGACCTCACATTCCTCAGCAGCGGAACCGGAACGGTGCGGTATCCCAGCGAGGAGGTCGTGTTGCAGTACGCTGCGGATCGAGAGCAGGTCAATCGACTGCAACGGCAACTGGCAGGGCCAGAAAAGCCGATCAAAGAACTGAAGGAGCTTATGGCCAATTATACAGGACTGGATAAGATCTATGAGATGGTTGACCAGGACCGCAATGCCATT |
| ds*Pi3K92E*-2 | AGAGATTGGCGTGCTGATTTAAATGGAAAACTTTGAAAACAACAATCTGTTGATAAGGTAACCAGCGAAATGCGGGACAACTAGCCTCCGTAGCCTGAAAGAGGCCATTGAAAAGTCTAATTAGGAGGAGCTGGTGATCCTCAAGATCCGAGGCACCAGATCCAAAATCCGAACTTGAAGATCAAATAGTCATGAACATGATGGACAACCGGGCGTTGGCCTACGTGGCCCACCAGCCCAAGTATGAGACACCGCCGGAAGAAGCGGAGCCGCCCTGCATGCGCTTTTCGGTTAACCTGTGGAAAAACGAGATGCTGAACTGGGTGGACCTAATCTGCCTGTTGCCCAATGGATTCCTGCTGGAGCTCAGGGTCAATCCGGCCAACACCATCCAGGTAATCAAGGTGGAGATGGTCAACCAGGCCAAACAGATGCCACTGGGCTATGTGATCAAAGAGGCCTGCGAGTACCAGGTGTACGGCATCTCGACCTTCAACATCGAACCTTACACCGACGAAACGAAGCGACTCAGTGAGGTC |
| ds*InR*-1 | GCCATGGAAAATGACCTGCCAGCCACAACGCCTACCAAGAAAATATCAGATCCTTTAGCAGGCGACTGTAAGTGCGTGGAGGGTTCGAAGAAGACTAGCAGTCAGGAATACGATGATCGTAAAGTTCAAGCGGGCATGGAGTTTGAGAACGCGTTGCAAAACTTTATATTTGTTCCAAACATTCGGAAAAGCAAGAATGGATCGTCTGACAAATCAGACGGAGCGGAAGGTGCAGCTCTCGATTCTAATGCTATTCCAAATGGAGGAGCTACTAACCCTTCACGTAGAAGGAGAGACGTTGCGCTCGAGCCAGAGCTCGACGATGTAGAGGGCAGTGTACTTCTACGCCATGTGCGCTCCATCACAGACGATACCGATGCATTTTTCGAAAAGGACGACGAAAATACCTATAAAGACGAAGAAGACTTGTCCTCCAACAAACAATTCTATGAGGTGTTTGCCAAGGAATTGCCACCAAATCAAACACATTTTGTCTTTGAAAAACTGCGCCACTTCAC |
| ds*InR*-2 | GGAACAGCCGTTCTCAATGTCACATTACAATCAGTGGGAGCAAACTCCGCTATGCTGAACGTCACGACAAAAGTTGAAATAGGAGAGCCCCAAAAGCCGAGCAATGCTACAATTGTTTTTAAGGATCCGCGCGCCTTCATCGGTTTCGTGTTTTATCATATGATCGATCCGTACGGGAACTCAACTAAAAGCAGTGACGATCCATGCGATGATCGCTGGAAGGTTAGCTCTCCGGAAAAGAGCGGGGTCATGGTATTAAGCAATTTGATTCCGTACACTAACTACTCCTACTACGTTCGGACCATGGCTATATCCTCGGAATTGACAAACGCGGAGAGCGACGTGAAGAACTTTAGGACGAATCCCGGACGACCGTCAAAGGTTACGGAGGTGGTAGCAA |
| ds*Pvr*-1 | TCGGGTCATTACAACGTTCAGGAATATGCCAATCGCACGATCCAAATGACCGCGAACTTTGAGGGATTTCCGACGCCCTCCTTCAGTTGGTTCAAACCCGATGGCACCGAGGTGCGACAATCGGAGAATAACTTCAAGATTCTCTCCACGGAATTGAGCACAATGCTCCAGGTGCTGAACGCCCAATTGCAGGACAGCGGCACGTATGTCCTCCGTGGATCCAATTCCTTCGGCGTCGTTCAGCGGGAGTACAACGTCAGTGTGATGGACGCACCGGCGCTGAAGATGTCGGACGCCTATGTCCAGGTGGGATCCGTGGCGCGACTGGAGTGCACAGTACGCTCCTATCCGCCGGCTATCGTGACCTTCTT |
| ds*Pvr*-2 | CAGCAGCCGGGAGATAGACTTGTACGTCCACGATCCCTCTGCTCCTCAGTGGACAAACGGCGGACAGGAGGGTCACTCGAAAATAAAGCGCAAACTAAGCCAAACGCTGGAGCTGGAGTGTGCCTCCACAGCGGTTCCCGTGGCAATTGTGCGTTGGTTTAAGGACGACAAGGAAGTGACCGAATCAAAGCTCAGGCACATCATTGAAAAGGAATCCAAGCTGCTGATCACTCACCTGTATCCCGGAGATGAAGGCGTCTACAAGTGTGTGGTGGAGAACCGATTGGACAGAATCGAACGCTCCTTCACGGTAGTGATATCAGATCTGCCCGGCATTAGCATGGCCTGGGTGTGGTTCGGTGTGATACTATTCCTCATCCTGATCGGTCTGTGCGTCTTCCTCGCCGTGCGCTACCAGAAGGAGCACAAGCGGCATCTGGCCCTTAAGGCAGCCGGATTGGCCAACTTCGAGGAGGGCGCCGTGGGACACATCAATCCCGATCTGAC |
| ds*Flo1*-1 | TACCTGAGGTCATTGGGTATGGCCCGCACGGCGGAGGTGAAGCGCGATGCCCGCATTGGCGAGGCTGAGGCCCGAGCGGAGGCCCACATTAAGGAGGCCATTGCCGAGGAGCAACGCATGGCCGCACGCTTCCTCAACGATACCGATATTGCCAAGGCCCAGCGCGACTTTGAGCTGAAGAAGGCAGCATACGATGTGGAAGTGCAGACCAAAAAGGCCGAGGCCGAGATGGCCTACGAGCTGCAAGCGGCCAAGACCAAGCAGCGCATCAAGGAGGAGCAGATGCAGGTGAAGGTGATCGAGCGCACGCAGGAGATTGCCGTCCAGGAGCAGGAGATCATGCGCCGCGAGCGAGAGCTGGAGGCCACCATCCGCCGACCGGCCGAGGCGGAGAAGTTCCGCATGGAGAAATTGGCCGAGGCCAACAAGCAGCGCGTGGTCATGGAAGCCGAG |
| ds*Flo1*-2 | CCAGGTAGAGAGTCCTTGCGTGTACACCAGCCAAGGAGTGCCCATCTCGGTGACAGGCATTGCACAGGTGAAGGTCCAGGGTCAGAACGAGGACATGCTGCTGACCGCCTGTGAGCAGTTCTTGGGCAAATCAGAGGCAGAGATCAACCACATCGCCTTGGTCACCCTGGAGGGGCATCAGCGTGCCATCATGGGTTCGATGACCGTGGAGGAGATCTACAAGGACCGCAAGAAGTTCAGCAAGCAGGTGTTTGAGGTGGCCTCCAGCGATTTGGCCAACATGGGAATAACCGTGGTTTCCTACACCATC |
| ds*Flo2*-1 | TGCTGTGGATCGACCAAGAAGCGCACGATTGTGGGCGGCTGGGCGTGGGCGTGGTGGCTGGTAACCGATGTCCAGCGACTGTCCCTCAATGTGATGACCCTGAATCCGATGTGCGAGAATGTGGAAACGTCGCAAGGTGTTCCGCTAACGGTGACCGGAGTGGCTCAATGCAAGAT |
| ds*Flo2*-2 | GCGAGGTGGCCGCACCGGACGTGGGTCGTATGGGCATCGAGATTCTCTCGTTTACGATCAAGGACGTCTACGATGATGTGCAGTACCTGGCCTCGTTGGGCAAGGCCCAGACCGCCGTGGTCAAGCGGGATGCAGATGCCGGCGTGGCGGAGGCCAATCGAGATGCCGGTATCCGTGAGGCGGAGTGCGAAAAGAGCGCCATGGATGTGAAATACTCGACGGACACGAAAATCGAGGACAACACCAGGATGTACAAGCTGCAGAAGGCCAATTTCGATCAGGAGATCAACACGGCCAAGGCCGAATCGCAGTTGGCCTACGAGCTGCAGGCAGCCAAGATCCGCCAGCGCATCCGTAACGAGGAGATTCAGATCGAGGTGGTGGAGCGACGCAAGCAGATCGAGATTGAGTCGCAGGAAGTGCAGCGCAAGGATCGCGAGCTCACTGGCACAGTCAAGCTGCCCGCCGAGGCCGAGGCCTTCCGCCTCCAGACCCTTGCGCAGGCCA |
| ds*Chc*-1 | TGTGCGAAAGTTCAACAAGCTCTTTACAGCCGGCCAGTATGCTGAAGCGGCTAAAGTTGCTGCCCTGGCACCCAAGGCCATTCTGCGTACGCCACAGACGATCCAGCGTTTCCAACAGGTGCAGACACCAGCTGGCTCCACGACTCCGCCGCTGCTGCAATACTTTGGCATTCTCCTCGACCAGGGCAAGCTGAACAAGTTCGAGTCTCTCGAGCTGTGCCGTCCCGTCTTGCTGCAGGGCAAGAAGCAGCTGTGCGAGAAGTGGCTGAAGGAGGAGAAGTTGGAATGCAGCGAGGAGTTGGGTGATCTGGTCAAGGCCTCCGATCTTACACTTGCCCTGTCCATCTATCTGCGCGCAAATGTGCCCAACAAGGTTATCCAATGCTTTGCTGAGACTGGGCAGTTCCAGAAGATTGTACTCTACGCCAAGAAGGTCAACTATACGCCCGATTACGTGTTCCTGCTGCGCTCCGTGATGCGAAGCAACCCGGAGCAAGGAGCTGGTTTCGCCTCTATG |
| ds*Chc*-2 | CCCGAACGGGTGAAGAACTTCTTGAAGGAGGCCAAGCTGACGGATCAGCTACCATTAATTATTGTTTGTGATCGTTTTGATTTCGTGCACGACTTGGTGCTTTACCTGTATCGTAACAATCTGCAGAAGTACATTGAGATCTATGTGCAGAAAGTGAATCCATCCCGCTTGCCAGTGGTAGTGGGTGGTCTTCTTGATGTTGATTGCAGTGAGGATATAATTAAAAATCTAATTCTCGTGGTCAAGGGACAATTCTCAACCGACGAACTGGTCGAGGAGGTCGAGAAGCGCAACCGTCTCAAGCTTCTCCTTCCCTGGCTGGAGTCCCGAGTTCACGAGGGCTGCGTCGAGCCAGCCACCCACAACGCGTTGGCCAAGATCTACATTGACTCGAACAACAATCCCGAGAGATATCTTAAGGAGAATCAGTACTACGATAGCCGTGTGGTCGGTCGCTACTGCGAGAAGCGGGATCCCCATTTGGCGTGTGTCGCCTACGAGCGTGGATTGTG |
